# Identification of Genes and Genomic Islands Correlated with High Pathogenicity in *Streptococcus suis* Using Whole Genome Tilling Microarrays

**DOI:** 10.1371/journal.pone.0017987

**Published:** 2011-03-30

**Authors:** Xiao Zheng, Han Zheng, Ruiting Lan, Changyun Ye, Yiting Wang, Ji Zhang, Huaiqi Jing, Chen Chen, Mariela Segura, Marcelo Gottschalk, Jianguo Xu

**Affiliations:** 1 State Key Laboratory for Infectious Disease Prevention and Control, Changping, Beijing, China; 2 National Institute for Communicable Disease Control and Prevention, Changping, Beijing, China; 3 School of Biotechnology and Biomolecular Sciences, University of New South Wales, Sydney, New South Wales, Australia; 4 Faculty of Veterinary Medicine, University of Montréal, St-Hyacinthe, Québec, Canada; University of California, San Francisco, University of California, Berkeley, and the Children's Hospital Oakland Research Institute, United States of America

## Abstract

*Streptococcus suis* is an important zoonotic pathogen that can cause meningitis and sepsis in both pigs and humans. Infections in humans have been sporadic worldwide but two severe outbreaks occurred in China in recent years, while infections in pigs are a major problem in the swine industry. Some *S. suis* strains are more pathogenic than others with 2 sequence types (ST), ST1 and ST7, being well recognized as highly pathogenic. We analyzed 31 isolates from 23 serotypes and 25 STs by NimbleGen tiling microarray using the genome of a high pathogenicity (HP) ST1 strain, GZ1, as reference and a new algorithm to detect gene content difference. The number of genes absent in a strain ranged from 49 to 225 with a total of 632 genes absent in at least one strain, while 1346 genes were found to be invariably present in all strains as the core genome of *S. suis*, accounting for 68% of the GZ1 genome. The majority of genes are located in chromosomal blocks with two or more contiguous genes. Sixty two blocks are absent in two or more strains and defined as regions of difference (RDs), among which 26 are putative genomic islands (GIs). Clustering and statistical analyses revealed that 8 RDs including 6 putative GIs and 21 genes within these RDs are significantly associated with HP. Three RDs encode known virulence related factors including the extracellular factor, the capsular polysaccharide and a SrtF pilus. The strains were divided into 5 groups based on population genetic analysis of multilocus sequence typing data and the distribution of the RDs among the groups revealed gain and loss of RDs in different groups. Our study elucidated the gene content diversity of *S. suis* and identified genes that potentially promote HP.

## Introduction

*Streptococcus suis* is an important zoonotic pathogen. *S. suis* infections in pigs lead to a wide range of clinical forms from severe clinical disease, such as meningitis, septicemia and bronchopneumonia, to subclinical infections [1], [2], [3]. In humans, *S. suis* can cause meningitis and sepsis in persons exposed to pigs and/or pork-derived products [4], [5]. To date, 35 serotypes, designated as 1 to 34 and ½, have been described for *S. suis* [6], among which serotype 2 is most frequently associated with disease in pigs and humans in most countries. Multilocus sequence typing (MLST) has classified *S. suis* into more than 181 sequence types (STs). The majority of human isolates of serotype 2 belong to the ST1 clonal complex [7], although recently ST27 complex was reported to be prevalent in human infections in Thailand [8]. ST1 clonal complex with 12 known member STs was shown to have a higher degree of virulence than other STs [7]. ST7 (all serotype 2) of ST1 clonal complex caused two large outbreaks in China in 1998 and 2005 respectively [9], [10]. Both outbreaks had caused significant mortality as a result of developing *Streptococcus* toxic shock-like syndrome [11].

There are more than 20 proposed virulence factors contributing to the pathogenesis of *S. suis* infections [12]. These factors include the capsular polysaccharide (CPS), muramidase-release protein (Mrp), extracellular factor (EF) and suilysin. CPS is the only proven critical virulence factor of *S. suis*. Unencapsulated isogenic mutants were shown to be avirulent in pig and mouse models of infection [13], [14]. CPS facilitates the survival of the organism in the bloodstream. The sialic acid component located in the terminal position of CPS is likely to be responsible for the antiphagocytosis [15]. Despite the lack of evidence that Mrp, EF and suilysin play a critical role in virulence, a positive association was observed between the presence of these factors and virulence in European and Asian strains [12]. In addition, suilysin appears to be toxic to not only epithelial and endothelial cells but also monocytes and neutrophils, suggesting a role in the immune evasion of the host [16], [17], [18]. Based on differences in the presence of known or putative virulence factors, the epidemiology of infections and animal model studies, *S. suis* isolates have been categorized into groups with different levels of pathogenicity [19], [20]. The categorizations are based on different criteria in different studies and thus present a large degree of uncertainties [21]. However, the strains which have caused severe outbreaks or sporadic invasive human infections are well recognized as highly pathogenic [12]. ST1 is a classical high pathogenicity (HP) ST while ST7 differing from ST1 by a single base in one of the 7 MLST genes is referred to as an epidemic strain [12], [20]. In this study only strains from these two STs are treated as highly pathogenic. These strains characteristically produce high levels of cytokines and chemokines in systemic infection, leading to serious invasive disease [12], [20].

There are 6 fully or partially sequenced *S. suis* genomes published [19], [20], [22]. All belong to ST1 complex with 3 each from ST1 (P1/7, BM407 and GZ1) and ST7 (SC84, 98HAH33 and 05ZYH33). However, the two ST7 genomes, 98HAH33 and 05ZYH33, reported by Chen *et al.* [19] have been shown to contain numerous sequencing errors [22]. Comparison of P1/7, BM407 and SC84 revealed high similarity among these genomes with predominantly small nucleotide differences as a result of point mutations and recombinations. The only large genomic island identified and present in both BM407 and SC84 but absent in P1/7 is a putative pathogenicity island composed of integrative conjugative elements and transposons carrying genes for drug resistance [22]. The other ST1 genome from strain GZ1 was compared with a partially sequenced ST25 genome (89–1591) available at the time revealing 132 genomic islands including 5 pathogenicity islands [20].

In this study, we used NimbleGen (NimbleGen Systems, Madison, WI) microarray based comparative genome resequencing (CGR) technology to conduct a comprehensive genomic comparison of 31 *S. suis* strains of 23 serotypes from different clinical sources using genome sequence of a HP strain, GZ1, as the reference to gain a deeper insight into the species diversity, genome variation and virulence. We chose NimbleGen microarray CGR as it does not only detect gene presence/absence, but potentially can also detect sequence variation in comparison to traditional microarray technology. We found that the *S. suis* genome is highly diverse and identified genes and genomic islands predominantly or exclusively distributed in highly pathogenic strains.

## Materials and Methods

### Bacterial strains

Thirty one *S. suis* strains [6], [9], [23], [24], [25] including 6 human isolates were used in this study (Table 1). The sample included 22 serotype reference strains [6], [23], [24], [25]. These strains represent 25 STs of the 181 known STs in *S. suis*. Strain GZ1 was used as a reference for comparative hybridization. Nine strains were from ST1 clonal complex.

**Table 1:** Strains used for comparative analysis.

Strain	Sero-group	Sequence Type	Country	Source	Virulence#	No. of genes absent
LN1	2	ST1$	China	Patient	HP	49
GZ2	2	ST7$	China	Patient	HP	50
P1/7	2	ST1$	England	Diseased pig	HP	50
GX1	2	ST1$	China	Patient	HP	52
31533	2	ST1$	France	Diseased pig	HP	51
SC22	2	ST7$	China	Patient	HP	50
SC84	2	ST7$	China	Patient	HP	51
13730	14	ST6$	Netherland	Patient		91
11611	2	ST11$	Germany	Unknown		131
89-1591	2	ST25	Canada	Diseased pig		210
2651	1/2	ST56	Netherland	Diseased pig		185
4961	3	ST35	Denmark	Diseased pig		224
6407	4	ST54	Denmark	Diseased pig		191
11538	5	ST53	Denmark	Diseased pig		184
2524	6	ST55	Denmark	Diseased pig		255
14636	8	ST87	Denmark	Diseased pig		188
22083	9	ST82	Denmark	Diseased pig		230
4417	10	ST78	Denmark	Diseased pig		248
12814	11	ST91	Denmark	Diseased pig		243
8830	12	ST74	Denmark	Diseased pig		244
10581	13	ST71	Denmark	Diseased pig		244
NCTC1046	15	ST81	Netherland	Diseased pig		213
2726	16	ST73	Denmark	Diseased pig		214
93A	17	ST76	Canada	Healthy pig		182
NT77	18	ST79	Canada	Healthy pig		230
42A	19	ST76	Canada	Healthy pig		164
14A	21	Unknown	Canada	Healthy pig		168
89-2479	23	ST80	Canada	Diseased pig		185
89-3576-3	25	ST69	Canada	Diseased pig		244
89-590	28	ST75	Canada	Diseased pig		197
92-1400	30	ST77	Canada	Diseased pig		189

# HP: highly pathogenic.

$ Strains belong to ST1 clonal complex.

### *S. suis* customized tiling array set

The *S. suis* tiling array set was designed based on the genome sequence of strain GZ1 (GenBank accession number CP000837). Customized tilling arrays were generated by using 29-mer oligonucleotide probes spaced every 7 bases across the reference genome on both strands. The GZ1 genome size is 2,038,034 bp and thus each sample required a set of two microarrays, each consisting of about 380,000 probes. The oligonucleotide probes of the microarray were synthesized *in situ* on glass surface using the Maskless Array Synthesis (MAS) technology (NimbleGen Systems, Madison, WI) in a random layout as previously described [26], [27].

### DNA fragmentation, labeling and hybridization

Two µg of DNA from each strain were dissolved to 80 µl MilliQ water and sonicated to 500–2000 bp by digital sonifier (Branson Corporation, Danbury, CT). The fragmented genomic DNA was labeled with 5′-dye labeled random nonamer (Cy3 for sample strains and Cy5 for reference strain GZ1) (TriLink BioTechnologies Inc., San Diego, CA). The reaction contained 100 units of Klenow fragment (NEB) and dNTP mix (6 mM each dATP, dGTP, dCTP, and dTTP respectively) and incubated for 2 h at 37°C. Reactions were terminated with 0.5 M EDTA (pH 8.0), and labeled samples were precipitated by NaCl and isopropanol, and stored at −20°C protected from light before hybridization.

Prior to hybridization, labeled control and test samples were dissolved in water and quantified using Nanodrop (NanoDrop Technologies, Wilmington, DE). Five µg each of test and reference samples were mixed and hybridized with the tiling arrays in NimbleGen hybridization buffer with a MAUI apparatus for 16 h at 42°C. After hybridization, microarrays were washed at room temperature with NimbleGen Wash buffers (NimbleGen Array Reuse Kit) according to the manufacturer's protocol. The washed arrays were then spun dry and scanned at 5 µm resolution using the Genepix 4000B scanner (Axon Instruments, Union City, CA).

### Microarray data acquisition and analysis

Images were extracted and processed using NimbleScan v2.4 software and analyzed using the NimbleGen commercial pipeline. The analysis results supplied contained hybridization signal intensities, reference to test signal ratio of each probe (1 if both hybridized with the same intensity, >1 if test probe contains mutation or absent, and <1 if more copies of test probe than reference probe) and probe hybridization calls (1 if positive, 0 if the ratio is not significantly different from 1) in GFF file format which enabled visually examination of potential mutations using NimbleGen SignalMap software and also text file format.

In order to determine the presence/absence of a gene, we developed a threshold based algorithm to identify absent genes for each test strain based on the probe hybridization calls. We first computed the percentage of positive calls (PPC) in a gene which equals to the percentage of positive call probes in the total number of tilling probes for a gene. As the smallest gene is around 110 bp and the average gene size is 908 bp, the smallest and average numbers of probes are 15 and 129 respectively. A higher PPC indicates a higher probability of the absence of the gene and vice versa.

We then used genome sequenced strains to determine the PPC cutoff values depending on the level of divergence of a test strain from the reference strain. Two genome sequenced strains, P1/7 and 89-1591, were included in the test strains. The former is closely related to GZ1 while the latter is distantly related [20]. Genes with a PPC value above zero were selected and the presence/absence of a gene in P1/7 or 89-1591 was verified by BLASTn searches. A gene with no BLAST hits or with homologous sequences covering no more than 20% of its reference length in GZ1 was determined as absent, otherwise the gene was deemed present. By comparing the PPC values and gene absence based on BLAST searches, we derived an optimal PPC value that minimizes the number of genes being called present but in fact they are absent and vice versa. This PPC value was used as a cutoff for other test strains. A P1/7 based PPC cutoff and the 89-1591 based PPC cutoff were derived to apply to ST1 clonal complex strains and non-ST1 clonal complex respectively.

### Phylogenetic analysis

The presence or absence of a gene was coded as binary data with gene presence as 1 and absence as 0. Hierarchical clustering on microarray data was conducted using Cluster 3.0, and trees were represented using TreeView [28]. Allelic sequences of the strains were obtained from *S. suis* MLST website (http://ssuis.mlst.net), concatenated and aligned using the CLUSTALW program. Neighbour joining trees were constructed using MEGA software version 4.1 [29] based on Kimura 2 parameter model.

### Bioinformatic analysis

The genome sequence data of *S. suis* P1/7 and 89-1591 used in this study were obtained from the Wellcome Trust Sanger Institute (ftp://ftp.sanger.ac.uk/pub4/pathogens/ss) and the DOE Joint Genome Institute (http://genome.jgi-psf.org/strsu/strsu.home). The genome of *S. suis* strain GZ1 used was sequenced by our laboratory and an early version of the current release in the NCBI database was used [20]. Comparison of the genomic sequences was facilitated by using the Artemis Comparison Tool (ACT) [30]. Distributional bioinformatics tool SMART [31] was used in the prediction and analysis of protein domains and motifs. IslandViewer [32] was used to predict genomic islands. Restriction/Modification systems were analyzed using REBASE [33]. STRUCTURE version 2.2 [34], which implements a Bayesian approach for deducing population structure from multi-locus data, was used to analyze the population clustering of an isolate, assuming that each isolate has derived all of its ancestry from only one population. The number of populations, *K*, was determined under the “admixture” model and in each simulation run, the Markov Chain Monte Carlo (MCMC) simulation of 30,000 iterations approximated the posterior probability of *K*, following a burn-in of 10,000 iterations. After multiple runs on each *K* assumed, the value that generated the highest posterior probability was used as the number of possible populations. The assignment of an isolate to a particular population was done under the linkage model.

### Array data deposition

MIAME compliant array data from this study have been deposited in the Gene Expression Omnibus database (GEO, http://www.ncbi.nlm.nih.gov/geo/) under GEO series accession number GSE26052.

## Results

### Overall hybridisation intensities from genome tiling microarrays

**Figure 1:**
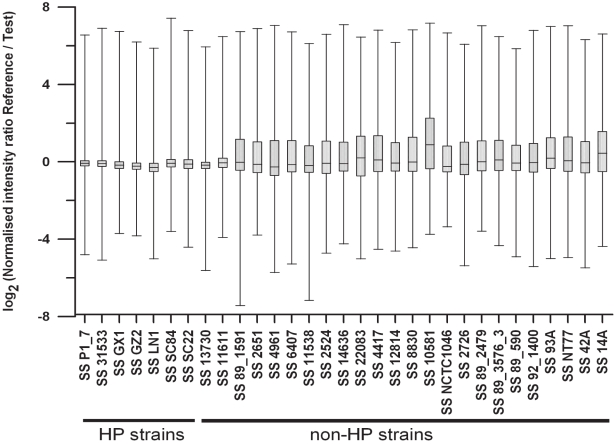
Box-Whisker Plot illustration on the distribution of hybridization intensities of the 760,000 probes for the 31 test *S. suis* strains. The signal ratios were plotted against each strain. The x-axis represents the microarray data of individual strains, while the y-axis represents the log_2_ transformed feature signal ratio. The box is defined by the lower and upper quartiles, and the line in the center of the box is the median. Whiskers represent the rest of the distribution, with caps at the ends of each box indicating the extreme values (minimum and maximum). The strain name corresponding to each microarray is shown at the bottom of the plot.

Thirty one strains were analyzed by CGR using GZ1 genome sequence as reference consisting of 760,000 tilling probes. The characteristics of the hybridization signal ratios for the 31 strains are shown in Figure 1 as a box plot. The signal ratio of the reference probe intensity over the test probe intensity is expected to be 1 or 0 if log_2_ transformed when there is no difference between the two probes. The log_2_ ratio shifts above 0 if mutation is present in test probe or absence of the test probe and below 0 if test genome contains more copies of the target region. The ranges of signal intensities across the strains are similar. The 25 to 75 percentiles are smaller for the closely related strains (ST1 clonal complex strains) while those for the more divergent strains are much bigger, consistent with expectations of higher level of single nucleotide polymorphism (SNP) difference and gene content difference. The majority of the strains have a median around zero. The majority of the strains also showed a higher positive signal above the median, consistent with absence of a gene or divergent sequences. Visual inspection of the signal intensities across the genome using the NimbleGen SignalMap software found that ST1 clonal complex strains showed few differences to the reference strain GZ1, whereas strains from the other STs displayed many differences. As array measurements for repetitive genetic elements are much less reliable than for unique sequences [35], repetitive elements such as IS and transposases were excluded in subsequent genomic comparisons.

### Detection of SNPs and gene content polymorphisms using tiling microarray

We first determined whether the hybridization data were sufficiently sensitive to detect SNPs by examining the hybridization signals of MLST sequence variation of the test strains since all strains used have been typed by MLST [7]. For three ST7 strains (SC22, SC84 and GZ2), single nucleotide changes in housekeeping gene *thyA* were detected by the tiling microarray in all cases. For the ST6 strain 13730, a single nucleotide change in *thyA* was also detected. However, for the ST11 strain 11611, a single nucleotide change in *aroA* was not detected. We further used the SNPs between the GZ1 and P1/7 genomes to determine the sensitivity of SNP detection. We identified 301 SNPs located at least 100 bp apart between the 2 genomes (we excluded closely located SNPs), among which, 102 were called as positive by Nimblegen algorithm, detecting only 33.9% of the SNPs. However 230 SNPs had at least one probe with a signal ratio of 2 or greater, suggesting that up to 76.4% SNPs can be potentially detected, although these SNPs will require resequencing for confirmation. In contrast, for the more distantly related strains, the MLST SNPs were not detected consistently. According to MLST data, there are 37 to 113 SNPs among these strains. However, none was called positive. These results indicated that it is not feasible to detect SNPs in these highly diverse strains. Therefore we did not analyze the hybridization data for SNP variation for the 31 strains tested.

**Figure 2:**
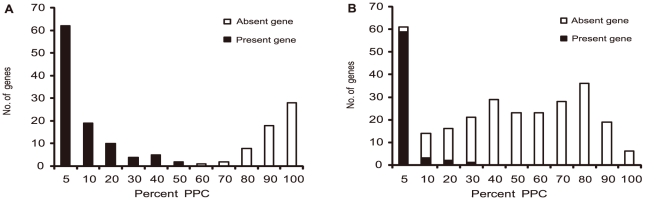
Distribution of percentage positive calls (PPC) for determination of cut-off values of calling a gene present or absent. (A) Histogram for P1/7 PPC values. (B) Histogram for 89-1591 PPC values. The actual presence (black-coded) or absence (white-coded) of a gene was determined by genome comparison between P1/7 (A) and 89-1591 (B) with GZ1 respectively. The cutoff PPC value minimizes the number of genes present being called absent.

We then determined the validity of using the tiling microarray data to predict gene presence or absence by comparing mapping data with genome sequence data using the two genome sequenced strains, P1/7 and 89-1591 and derived a PPC value to determine the presence or absence of a gene. There were 159 and 278 genes from P1/7 and 89-1591 hybridizations with a PPC above 0% respectively. By BLASTn searches against respective genome sequences, 57 of the 159 genes in P1/7 and 207 of the 278 genes in 89-1591 were determined to be absence in P1/7 and 89-1591. We further found that the true absent genes have a higher PPC value, as can be seen in Figure 2 of the distribution of the PPC values which was plotted in 10% PPC intervals and 5% intervals for those less than or equal to 10%. Interestingly the PPC distribution is different for the two strains. The P1/7 PPCs have a U shape distribution while the 89-1591 PPCs more evenly distributed except for the 0–5% PPC class. Using this data we can find that for P1/7, a PPC of 50% as cutoff identified the 57 absent genes correctly with 0% false positives or false negatives. For 89-1591, a PPC cutoff of 8% correctly identified the 207 genes as absence but also identified another 6 “present” genes as absent with a false detection rate of 3% (6/213). Thus, we conclude that a PPC of 50% for P1/7 and 8% for 89-1591 respectively as the threshold value is optimal to distinguish the absence of a gene. Notably there is a tradeoff between the rejection of false positives and the retention of true absence in determining the threshold value for 89-1591 since its genome is more divergent from the reference genome. Therefore ST1 complex strains were analyzed using the 50% PPC cut-off as GZ1 belongs to ST1 while other strains were analyzed using the 8% PPC cut-off.

### The core genome of *S. suis* and the accessory genome of the reference strain GZ1

**Figure 3:**
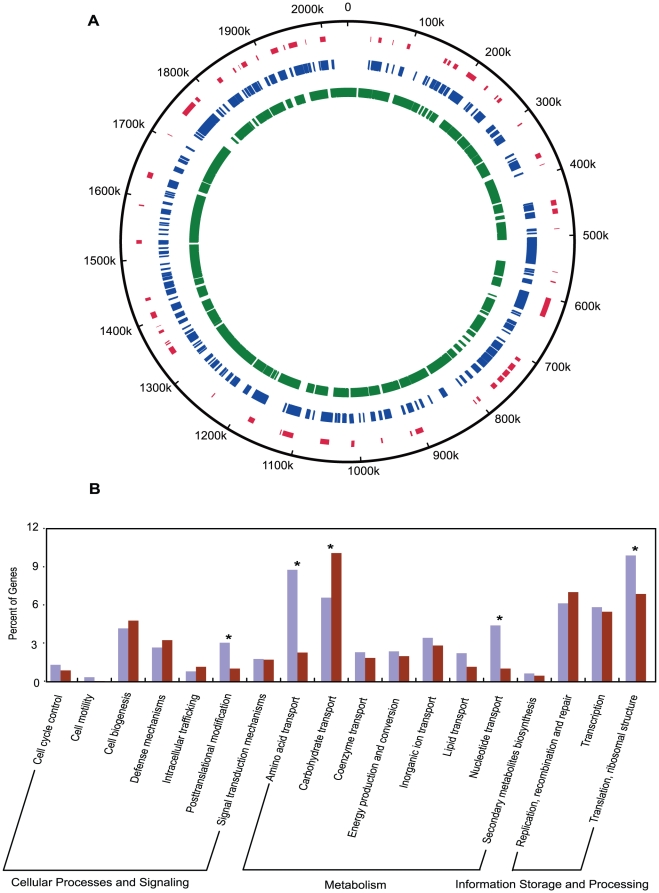
Distribution and functional categories comparison of core genes and accessory genes in GZ1 genome. (A) Circular representation of the distribution of core and accessory genes and regions of difference on the reference GZ1 genome. The outmost circle shows the scale in kilobases. The inner circles (from outside to inside) represent features: regions of difference, accessory genes and core genes. (B) Distribution of core and accessory genes in different functional categories. For each category the percentage of core and accessory genes were shown in columns in pairs with the core genes on the left (purple) and the accessory genes on the right (dark red). The star above the columns indicates that there is a significant difference in the distribution of core and accessory genes in the given functional category (*p*<0.05).

The number of genes absent in a strain ranged from 49 to 225 with a total of 632 genes absent in at least one strain, while 1346 genes are invariably present in all strains (Table S1). We define the former as the accessory genome of GZ1 and the latter as the core genome of *S. suis* respectively (Figure 3A) and the core genes account for 68% of the GZ1 genome. The ST1 clonal complex strains have far fewer genes absent as expected, ranging from 49 to 91 genes.

The core genes and accessory genes were further categorized into cluster of orthologous groups gene functional categories and their distribution is shown in Figure 3B. Four categories, amino acid transport, nucleotide transport, translation and ribosomal structure and posttranslational modifications, were significantly over-represented by core genes while carbohydrate transport was the only category significantly over-represented by the accessory genes. The latter phenomenon is likely to be a reflection of adaptation to oral and respiratory tract environment rich in glycoproteins and murein polysaccharides which previously was proposed to contribute to the pathogenic potential of pathogenic streptococci [36], [37]. Known virulence determinants and immunogenic proteins are present in both core and accessory genome, with 23 (1.7%) in the former and 15 (2.4%) in the latter (Table S2).

The majority (365) of the accessory genes consisted of two or more contiguous genes in blocks ranging from 0.5 to 46 kbp in size, among which, 62 were absent in two or more strains while 17 were absent in only one strain (Table S1, Table S3). We defined the former as regions of difference (RDs) which were analyzed in more detail below. Genes in RDs often displayed heterogeneous patterns of presence or absence with the exception of 14 RDs (RD5, RD7, RD9, RD13, RD20, RD26, RD28, RD33, RD34, RD35, RD46, RD48, RD58, and RD62). RDs were scattered around the GZ1 genome (Figure 3A). Five RDs (RD1, RD4, RD29, RD32, and RD45) were found to contain prophage remnants, indicating that they have been acquired in the reference genome by horizontal transfer. We analyzed the RDs for structural features of genomic islands (GIs) including sequence composition bias, tRNA genes as insertion site, direct repeats flanking the RD and integrases [38] and found 26 of the 62 RDs contain sufficient features to be putative GIs (Table S4).

In addition to blocks of variable genes, 93 singular genes were identified to be individually absent in two or more of our test strains. We defined these genes as singular variable genes (SVG, Table S5) which were analyzed in more detail below. Notably, several genes encoding important virulence determinants such as suilysin (SSGZ1_1246), Sao (SSGZ1_1217), RevS (SSGZ1_1897) and Mrp (SSGZ1_0743) were in the SVG set. Lastly, 101 genes were absent in only one test strain and were not subjected to further comparative analysis (Table S1). Of note, several pathogenesis related proteins: dipeptidyl aminopeptidase IV (SSGZ1_0184), DNAse (SSGZ1_1784), cell wall hydrolase/autolysin (SSGZ1_1146), surface associated subtilisin-like serine protease (SSGZ1_1797), and CovR regulator (SSGZ1_1561) belonged to this set (Table S2).

### Clustering and statistical analyses reveal RDs correlated with high pathogenicity

**Figure 4:**
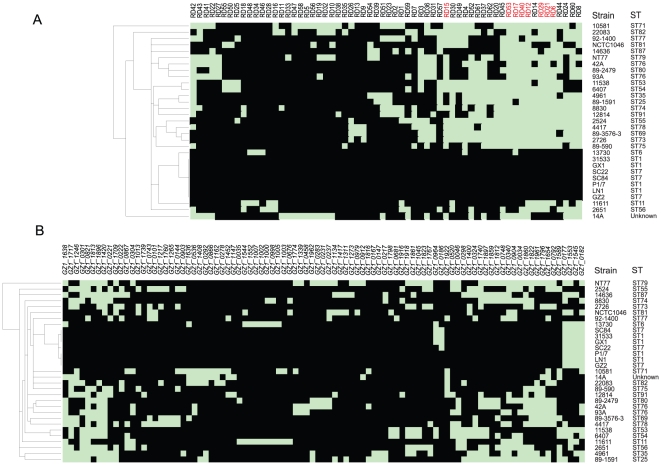
Clustering of variable genes and regions detected by tiling microarray of 31 *S. suis* strains. (A) Clustering of regions of difference (RDs) and *S. suis* strains based on the presence or absence of RDs in the 31 test strains. For each individual strain, the presence of a RD is represented by a black box, whereas the absence of a RD corresponds to a cyan box. The dendrogram constructed by average linkage hierarchical cluster analysis shows the relationships of the 31 strains. The strain names and sequence types are presented on the right side. RD numbers are given at the top and red text indicates high pathogenicity associated RDs. (B) Clustering based on the presence or absence of singular variable genes (SVGs). Color coding is the same as in (A).

To determine whether a RD is more likely to be associated with HP, we conservatively use ST1 and ST7 strains as the HP group [20] which consisted of 7 strains and grouped the remaining 24 strains as non-HP group. As genes in RDs may be variably present among the strains, we first applied the rule that as long as one gene is absent in a strain the RD was treated as absence to identify all RDs potentially correlated with HP. We applied hierarchical clustering to RD patterns after this recoding (Figure 4A). RD based clustering showed that HP strains (ST1 and ST7) were grouped together and were most close to the reference genome. The remaining strains displayed the most polymorphisms in RD content. The RD clustering is consistent with the division of the strains into HP and non-HP groups (Figure 4A).

We identified 12 RDs (RD6, RD8, RD12, RD14, RD15, RD17, RD21, RD29, RD40, RD45, RD53 and RD60) preferentially present in HP strains. The differential distribution of RDs was tested using Fisher's exact test and all were statistically significant with a *p* value of 0.0008 or less (we used *p*≤0.05 as the significant level before Bonferroni correction for multiple tests for 62 tests). To further pinpoint genes within a block which are associated with HP, we tested each gene individually using p value of 0.00052 as cutoff after Bonferroni correction for multiple tests (*p*≤0.05, 97 tests). Twenty-one genes from 8 RDs were found to be significant. Interestingly, for 4 RDs (RD8, RD14, RD45, and RD60) no genes reached the significance cutoff, although all 4 RDs contain genes with uncorrected p value of *p*≤0.01. Therefore we treat only 8 RDs as HP associated (Table 2).

**Table 2:** Regions of difference associated with high pathogenicity.

Region of difference	HP# strains	Non-HP strains	P value$	Annotated functions	GZ1 locus tags	References
RD6	7/7	3/24	4.56×10^−5^	Extracellular factor; Putative RTX family exoprotein	SSGZ1_0164–SSGZ1_0166	Vecht *et al.* [39]
RD12	7/7	1/24	3.04×10^−6^	MerR regulator; Hypothetical protein	SSGZ1_0342–SSGZ1_0346	
RD15&	7/7	4/24	1.25×10^−4^	SrtF pilus	SSGZ1_0420–SSGZ1_0424	Takamatsu *et al.* [40]
RD17&	7/7	3/24	4.56×10^−5^	Capsular polysaccharide synthesis	SSGZ1_0555–SSGZ1_0577	Charland et al. [13]; Smith *et al.* [14]
RD21&	7/7	2/24	1.37×10^−5^	HsdR; Transposase; HsdM; HsdS; Hypothetical protein	SSGZ1_0682–SSGZ1_0690	
RD29&	7/7	4/24	1.25×10^−4^	Abortive bacteriophage resistance protein	SSGZ1_0848–SSGZ1_0849	
RD40&	7/7	3/24	4.56×10^−5^	Type I restriction-modification system	SSGZ1_1285–SSGZ1_1288	
RD53&	7/7	3/24	4.56×10^−5^	Hydrolase family protein; Nicotinamide mononucleotide transporter; Regulator; ATP-dependent Clp protein; CtsR regulator	SSGZ1_1787–SSGZ1_1791	

# HP: highly pathogenic. All other strains are treated as non-HP.

$ P value based on Fisher's exact test.

& These RDs are putative genomic islands. See text and Table S4 for details.

We also applied clustering and statistical tests to SVGs. It is less apparent of the clustering of SVGs (Figure 4B). Three genes were found to be associated with the HP group but the p value was not significant after correcting for multiple tests. Of note, genes encoding the 2 known virulence factors, suilysin and Mrp, were not significantly associated with HP.

### Functional significance of RDs and SVGs associated with HP strains

Among the 8 HP associated RDs identified above, two, RD6 and RD17, encoding extracellular factor and serotype 2 capsular polysaccharide respectively, are known virulence factors of *S. suis* [14], [39] while another, RD15, encoding a *srtF* pilus cluster, has recently been reported to be preferentially distributed in ST1 clonal complex strains [40].

RD53 is interesting. It contains 5 genes (SSGZ1_1787–SSGZ1_1791) encoding a hypothetical protein, a nicotinamide mononucleotide transporter, a transcriptional regulator, an ATP-dependent Clp protease and a CtsR transcriptional regulator, and is homologous to a region (SGO1995–SGO1999) in *Streptococcus gordonii* genome [41], with amino acid sequence identity ranging from 54% to 72%, suggesting that RD53 may have been acquired from *S. gordonii* by lateral gene transfer. However since *S. gordonii* is a commensal of oral and respiratory tract, the genes are less likely to be virulence factors *per se*. Further bioinformatics analysis revealed that RD53 might be associated with virulence. The first three genes may facilitate the utilization of extracellular V factors (NADP, NAD and NMN). Of the 2 remaining genes (SSGZ_1790 and SSGZ_1791) in RD53, *SSGZ1_1790* encodes a ClpC protein [42] as it contained two nucleotide binding domains (AAA-1, AAA-2) and two Clp_N domains involved in protein binding, as well as the presence of specific signature sequences while *SSGZ1_1791* encodes a conserved heat shock protein regulator, CtsR of Gram-positive bacteria [43], [44]. ClpC protein could affect virulence as it contributes to stress tolerance and virulence in *Streptococcus pneumoniae* [45] and host cell invasion in *Listeria monocytogenes* [46]. Based on the CGR data, *clpC* and *ctsR* were present in 11 and 14 strains respectively. Further PCR and sequencing analyses showed that both *clpC* and *ctsR* are present in all the strains studied and for those strains whose *clpC* and *ctsR* were absent by CGR analysis, the *clpC* and *ctsR* sequences were very divergent with only 76% and 80% identity at nucleotide level respectively (data not shown). These results suggest that only the first 3 genes of RD53 are uniquely associated with HP and the divergent *clpC* and *ctsR* genes were brought in during the acquisition of the first 3 genes. The homologous *clpC*/*ctsR* flanking regions may have mediated the genetic exchange of the first 3 genes of RD53.

Two RDs are restriction-modification related. RD40 encodes an *Eco*EI type I restriction-modification system [47] while RD21 encodes another type I restriction-modification system and two hypothetical proteins. Restriction-modification systems including both Types I and II have been proposed to play a role in virulence in other species including *Helicobacter pylori, Neisseria meningitides* and *Yersinia pseudotuberculosis* [48], [49], [50].

The remaining 2 RDs (RD12 and RD29) have functions unlikely to be related to virulence or have no known functions to determine their potential role in virulence. RD12 encodes a MerR family regulatory protein (SSGZ1_0342) and four hypothetical proteins (SSGZ1_0343 to SSGZ1_0346). Interestingly, BLAST search revealed that the latter shared homology with a set of four genes (STRSA0001_1199–STRSA0001_1202) in *Streptococcus salivarius* strain SK126, with amino acid sequence identity ranging from 11% to 56%. Further analysis showed that SSGZ1_0343 may encode a dynamin-superfamily protein as it contained a Dynamin_N domain [51]. RD29 is phage-related and contains a gene encoding an abortive phage infection resistance protein [52].

### Phylogenetic relationship based on variable gene content and evolutionary dynamics of regions of difference

**Figure 5:**
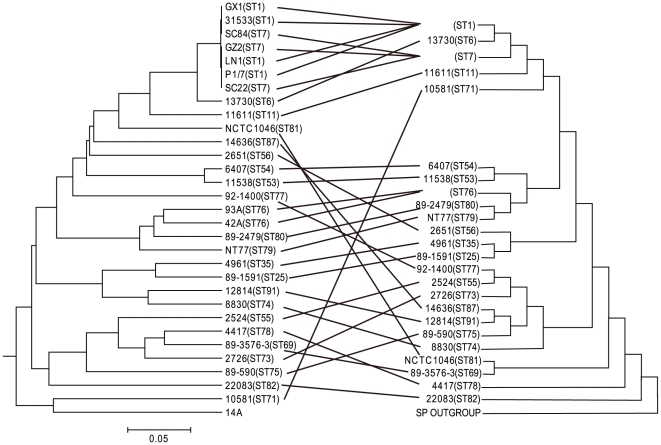
Comparison of phylogenetic relationships based on microarray data and multi-locus sequence typing (MLST) data. Dendrogram on the left was based on microarray data for the presence and absence of genes using UPGMA while phylogenic tree on the right in mirrored image was based on the concatenated sequences of 7 MLST alleles constructed using the neighbour joining method. The branches showing similar groupings were connected by lines. MLST based tree was rooted using a *Streptococcus pneumonia* strain marked as SP as an outgroup. Microarray data based tree was unrooted.

We used the presence and absence of genes to construct a dendrogram using Unweighted Pair Group Method with Arithmetic Mean (UPGMA) (Figure 5 left). The relationships based on microarray data was compared with that based on 7 housekeeping genes as the strains used in this study have been typed previously using MLST except for one strain (14A) from which only 6 housekeeping gene sequences were obtainable [7]. The housekeeping gene tree was constructed using the neighbour joining method and *S. pneumoniae* was used as an outgroup (Figure 5 right). Comparison of the two trees shows that the closely related strains based on MLST were largely grouped together by the microarray data, including strains belonging to 3 clonal complexes: ST1, ST25/ST35 and ST53/ST54. Another 2 sets of strains, 2524 (ST55) and 92-1400 (ST77), and 93A (ST76), 42A (ST76), NT77 (ST79) and 89-2479 (ST80) are also consistent between the two trees. However 89-3576-3 (ST69), 2726 (ST73) and 4417 (ST78) were grouped together in the microarray data tree but were separated on different branches on the MLST tree. Conversely 14636 (ST87) and 12814 (ST91) grouped together on the MLST tree are on separate branches on the microarray data tree.

However, there is no consistency in major branching orders between microarray data and MLST trees. A possible explanation is that the MLST tree has been affected by recombination. A previous study by King *et al.* [7] showed that *S. suis* has relatively high levels of recombination. We used their MLST data to determine the relative contribution of recombination using the method of Feil *et al.* [53]. Among the 10 clonal complexes (STs differing by one allele) identified by King *et al.* [7], 21 allelic changes contained a single base mutational difference while 14 has 2 to 25 base differences which were attributed to recombination. Thus recombination contributes 40% (14/35) to the diversification of clonal complexes. However when combined, the number of base changes introduced by recombination is much larger than that introduced by mutation. The placement of a strain on the tree is thus much more heavily affected by recombination.

**Figure 6:**
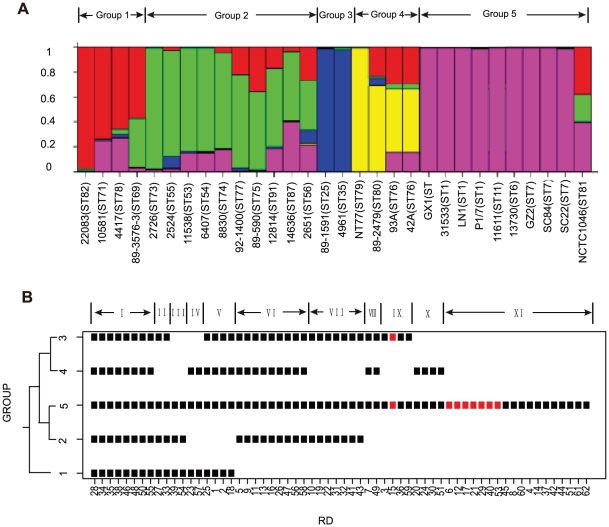
Population structure and distribution of regions of difference (RDs) among subpopulations. (A) Division of the 31 strains into subpopulations (groups) based on STRUCTURE analysis of multilocus sequence typing data. The 5 subpopulations (groups) are colour coded. Group numbering and strain details are shown above and below the population strip respectively. Each isolate has been allocated to a subpopulation. Imports from another subpopulation are shown in its respective subpopulation colour. Y-axis is the proportion of subpopulation allocation for a strain. (B) Distribution of RDs among the subpopulations (groups). On the left is a phylogeny of the 5 groups, based on neighbour joining tree of representative strains without imports from each group shown in (A). On the right is the plot of the distribution of RDs. The distribution was grouped into 11 patterns as shown at the top. Each square represents a RD present in greater than 75% of the strains in that group. Squares marked red were RDs associated with high pathogenicity strains as identified by cluster and statistical analysis.

To overcome the adverse effect of recombination on the MLST tree and to provide a phylogenetic framework to determine the gain or loss of the RDs, we used population genetic analysis as done previously [54] to divide the *S. suis* strains into subpopulations. STRUCTURE analysis of the MLST data of the 7 house keeping genes for these strains previously published by King *et al.* [7] found 5 subpopulations/groups with group 5 containing the HP strains of ST1 clonal complex (Figure 6A). It also revealed that many strains contained sequences from other groups, which is consistent with the high level of recombination seen in clonal complexes as shown above. We then used the strains that showed no recombinant ancestry to construct a neighbour joining tree to establish the evolutionary relationships of these groups (Figure 6B). The tree shows that group 1 is the ancestral group with groups 2 to 5 divided into 2 lineages evolved in parallel.

We next calculated the percentage of presence of an RD in each group and mapped the RDs onto the group tree (Figure 6B). An RD is shown as present if the RD is present in 75% or more of the strains in a given group. The distribution of RDs can be categorized into 11 patterns. Pattern I is present in all groups and therefore the loss of these RDs occurred sporadically in differently lineages. Patterns II to V show RDs that are present in the earliest group (group 1) but deleted in one or more other groups. Patterns VI and VII are RDs gained after diverging from group 1 but pattern VII RDs have been lost in group 4. Pattern VIII is also likely to have been gained by the common ancestor of groups 2 to 5 with independent loss in group 2. Patterns IX and X could be independent gains or losses as these are present in one of the 2 member groups in each lineage (groups 2/5 and groups 3/4). Pattern XI RDs were apparently gained by group 5. Majority of the group 5 RDs are associated with HP.

## Discussion

In this study we used NimbleGen tiling microarray to determine the gene content variation of *S. suis* using the HP strain GZ1 genome as reference and a selection of 31 test strains including 7 HP strains from ST1 clonal complex. We show that of the 1978 GZ1 genes studied, 1346 (68%) are invariably present in all strains as the core genome of the species. The number of core genes in *S. suis* is similar to that observed for other *Streptococcus* species but much larger than the number of genes (858) shared by *S. suis* with its two close species *S. pneumoniae* and *S. thermophilus* [55]. There is a wide variation of the presence of the accessory genes among the strains tested ranging from 49 (2.5%) to 255 (12.9%) genes absent. As expected, strains more closely related to GZ1 vary less and verse versa. Therefore if other strains have a similar genome size to GZ1, more accessory genes will be discovered from these strains when their genomes are sequenced. The *S. suis* pan genome is potentially very large.

Microarray based comparative genomic studies commonly use PCR products or gene specific oligonucleotides as detection probes. We used NimbleGen tiling microarray to detect gene content difference. The advantage of this technology is its potential to detect both deletions and single base changes with tiling probes covering the entire genome. Tiling microarrays have been widely used as the first step of mutation mapping for genome resequencing [35], [56], [57], [58], [59] and have been used for gene deletion detection [60], [61]. However it had not been used for direct detection of gene content difference. We developed a new algorithm to analyze tiling microarrays for the determination of presence or absence of a gene, which was made feasible by using two genomes one of which is closely related to the reference while the other is more distantly related. The cut-off for calling a gene present or absent was dependent upon the percentage of probes being hybridized with a high signal ratio, which we see as a limitation of the NimbleGen tiling microarray approach for comparative genome content analysis.

Previous studies have identified various genes which either directly contribute to virulence or act as virulence markers based on functional studies [12], [62], differential distribution between pathogenic and less or non-pathogenic strains [20], [63] or genome comparisons [19]. This study performed a systemic analysis of GZ1 genes for their correlation with HP using a set of strains well recognized for their high pathogenicity. We used clustering and statistical analyses of the microarray data to find 8 RDs and 21 genes within these RDs that are significantly associated with HP. The validity of this approach is demonstrated by finding 3 RDs that encode known high pathogenicity related virulence factors including the extracellular factor, serotype 2 capsular polysaccharide and a SrtF pilus. We also found one RD encoding genes that may facilitate the utilization of extracellular V factors (NADP, NAD and NMN) which may contribute to virulence. However, there are other genes which show only an association. Previous studies suggest that HP may not be determined by a single gene [2], [12]. Our analysis confirms this notion and the HP associated genes may additively contribute to the higher virulence of some *S. suis* strains. Variably presence of these genes explains the varied pathogenic potential of the diverse *S. suis* strains isolated from pigs. However, many human isolates are from serotype 2. It would be interesting to determine the presence of the HP associated genes in serotype 2 strains from other lineages to shed light on the association of this serotype with virulence.

However, there are 2 caveats in the determination of association of RDs with high pathogenicity. Firstly the strains in the HP group are phylogenetically closely related as all were from ST1 complex. Genes gained by the HP group in its most recent common ancestor will show the highest association but may have no role in pathogenesis, i.e. these genes are indirectly associated with virulence through common descent. For example, since all strains in the HP group are serotype 2 it is not surprising that RD17 encoding serotype 2 capsular polysaccharide was found to be highly associated with HP, although in this case the capsule is known to be important to virulence. However, 6 (RD15, RD17, RD21, RD29, RD40 and RD53) of the 8 HP associated RDs are putative GIs, suggesting that these RDs were at least initially mobile and acquired from another source. Two RDs (RD6 and RD17) encode known virulence factors and 4 (RD12, RD15, RD29 and RD53) may play a role in virulence although their role in virulence require experimental confirmation. Secondly, it is debatable how to assign a strain to an HP group for the determination of an association. We did not assign to the HP group the serotype 14 strain 13730 of ST6 analyzed by CGR in this study. However, serotype 14 strains have been increasingly isolated from human infections [64], [65], [66], [67]. Kerdsin *et al.* [65] typed 12 serotype 14 isolates from Thailand by MLST and found them to be either ST105 (11 isolates) or ST127 (1 isolate), both of which belong to the ST1 clonal complex. Thus serotype 14 strains from ST1 complex can also be regarded as highly pathogenic. We reanalyzed the association of RDs with HP by assigning our serotype 14 ST6 strain to the HP group together with the 7 ST1 and ST7 strains. An additional 4 RDs (RD8, RD14, RD45, and RD60) were found to be statistically significantly associated with HP. RD8 contains a gene (SSGZ1_0196) encoding a protein with “parallel beta-helix repeat” domains. RD14 has no predictable functions except for a DNA/RNA helicase of superfamily II (SSGZ1_0412) while RD45 contains remnants of a phage. RD60 encodes a *srtBCD* pilus gene cluster and has been shown to be preferentially distributed in ST1 complex strains together with RD15 as described above [40]. Three singular genes (SSGZ1_0821, SSGZ1_1420 and SSGZ1_1896, encoding a hypothetical protein, a putative membrane protein and a CAAX amino terminal protease family protein respectively) were also found to be significantly associated with HP.

We found that the genes gained or lost are mostly grouped together with two or more genes as chromosomal blocks which are likely to have been gained by lateral gene transfer. Twenty-six of the 62 RDs are putative GIs, further supporting this notion. One block (RD53) was clearly imported from outside of the species. There are also many singularly absent genes. Since there is high level of recombination in housekeeping genes as demonstrated in our STRUCTURE analysis, many of these genes may be obtained by hitchhiking due to homologous recombination of their flanking genes. Many such events have been observed in the comparison of *Vibrio cholerae* genomes [68], a species with high level of recombination.

As the recombination rate has not been quantified previously, we used MLST data from King *et al.* [7] to determine the contribution of recombination to the diversification of *S. suis* clonal complexes. We found that the relative contribution of recombination to mutation is 1∶1.5. As recombination brings in far more base changes to an allele than mutation which adversely affects the inference of true phylogenetic relationships, we used a population genetic approach to divide the strains into 5 groups and established the evolutionary relationships of these groups. The distribution of the RDs along the phylogenetic tree revealed gain and loss of RDs in different lineages.

In conclusion, our study elucidated the gene content evolution of *S. suis*, identified genes that potentially promote high pathogenicity and divided the *S. suis* population into 5 groups, providing significant insight into the evolution of pathogenicity.

## Supporting Information

Table S1 **Distribution of GZ1 genes in 31 test strains identified by microarray.**
(DOC)

Table S2 **Distribution of virulence related factors or immunogenic proteins in core or accessory genome of** ***Streptococcus suis*** **GZ1.**
(DOC)

Table S3 **Summary of genomic RDs and their distribution in 31 test strains according to their pathogenic capacity.**
(DOC)

Table S4 **Characteristics of putative genomic islands.** GI features for 26 regions of difference (RDs) are described.
(DOC)

Table S5 **Summary of SVG and their distribution in 31 test strains according to their pathogenic capacity.**
(DOC)

## References

[1] Chanter N, Jones PW, Alexander TJ (1993). Meningitis in pigs caused by *Streptococcus suis*–a speculative review.. Vet Microbiol.

[2] Gottschalk M, Segura M (2000). The pathogenesis of the meningitis caused by *Streptococcus suis*: the unresolved questions.. Vet Microbiol.

[3] Reams RY, Glickman LT, Harrington DD, Thacker HL, Bowersock TL (1994). *Streptococcus suis* infection in swine: a retrospective study of 256 cases. Part II. Clinical signs, gross and microscopic lesions, and coexisting microorganisms.. J Vet Diagn Invest.

[4] Arends JP, Zanen HC (1988). Meningitis caused by *Streptococcus suis* in humans.. Rev Infect Dis.

[5] Kopic J, Paradzik MT, Pandak N (2002). *Streptococcus suis* infection as a cause of severe illness: 2 cases from Croatia.. Scand J Infect Dis.

[6] Higgins R, Gottschalk M, Boudreau M, Lebrun A, Henrichsen J (1995). Description of six new capsular types (29-34) of *Streptococcus suis*.. J Vet Diagn Invest.

[7] King SJ, Leigh JA, Heath PJ, Luque I, Tarradas C (2002). Development of a multilocus sequence typing scheme for the pig pathogen *Streptococcus suis*: identification of virulent clones and potential capsular serotype exchange.. J Clin Microbiol.

[8] Takamatsu D, Wongsawan K, Osaki M, Nishino H, Ishiji T (2008). *Streptococcus suis* in humans, Thailand.. Emerg Infect Dis.

[9] Ye C, Bai X, Zhang J, Jing H, Zheng H (2008). Spread of *Streptococcus suis* sequence type 7, China.. Emerg Infect Dis.

[10] Ye C, Zhu X, Jing H, Du H, Segura M (2006). *Streptococcus suis* sequence type 7 outbreak, Sichuan, China.. Emerg Infect Dis.

[11] Yu H, Jing H, Chen Z, Zheng H, Zhu X (2006). Human *Streptococcus suis* outbreak, Sichuan, China.. Emerg Infect Dis.

[12] Gottschalk M, Segura M, Xu J (2007). *Streptococcus suis* infections in humans: the Chinese experience and the situation in North America.. Anim Health Res Rev.

[13] Charland N, Harel J, Kobisch M, Lacasse S, Gottschalk M (1998). *Streptococcus suis* serotype 2 mutants deficient in capsular expression.. Microbiology.

[14] Smith HE, Damman M, van der Velde J, Wagenaar F, Wisselink HJ (1999). Identification and characterization of the cps locus of *Streptococcus suis* serotype 2: the capsule protects against phagocytosis and is an important virulence factor.. Infect Immun.

[15] Charland N, Kobisch M, Martineau-Doize B, Jacques M, Gottschalk M (1996). Role of capsular sialic acid in virulence and resistance to phagocytosis of *Streptococcus suis* capsular type 2.. FEMS Immunol Med Microbiol.

[16] Chabot-Roy G, Willson P, Segura M, Lacouture S, Gottschalk M (2006). Phagocytosis and killing of *Streptococcus suis* by porcine neutrophils.. Microb Pathog.

[17] Lalonde M, Segura M, Lacouture S, Gottschalk M (2000). Interactions between *Streptococcus suis* serotype 2 and different epithelial cell lines.. Microbiology.

[18] Segura M, Gottschalk M (2002). *Streptococcus suis* interactions with the murine macrophage cell line J774: adhesion and cytotoxicity.. Infect Immun.

[19] Chen C, Tang J, Dong W, Wang C, Feng Y (2007). A glimpse of streptococcal toxic shock syndrome from comparative genomics of *S. suis* 2 Chinese isolates.. PLoS One.

[20] Ye C, Zheng H, Zhang J, Jing H, Wang L (2009). Clinical, experimental, and genomic differences between intermediately pathogenic, highly pathogenic, and epidemic *Streptococcus suis*.. J Infect Dis.

[21] Gottschalk M, Higgins R, Quessy S (1999). Dilemma of the virulence of *Strptococcus suis* strains.. J Clin Microbiol.

[22] Holden MT, Hauser H, Sanders M, Ngo TH, Cherevach I (2009). Rapid evolution of virulence and drug resistance in the emerging zoonotic pathogen *Streptococcus suis*.. PLoS One.

[23] Gottschalk M, Higgins R, Jacques M, Beaudoin M, Henrichsen J (1991). Characterization of six new capsular types (23 through 28) of *Streptococcus suis*.. J Clin Microbiol.

[24] Gottschalk M, Higgins R, Jacques M, Mittal KR, Henrichsen J (1989). Description of 14 new capsular types of *Streptococcus suis*.. J Clin Microbiol.

[25] Perch B, Pedersen KB, Henrichsen J (1983). Serology of capsulated streptococci pathogenic for pigs: six new serotypes of *Streptococcus suis*.. J Clin Microbiol.

[26] Nuwaysir EF, Huang W, Albert TJ, Singh J, Nuwaysir K (2002). Gene expression analysis using oligonucleotide arrays produced by maskless photolithography.. Genome Res.

[27] Singh-Gasson S, Green RD, Yue Y, Nelson C, Blattner F (1999). Maskless fabrication of light-directed oligonucleotide microarrays using a digital micromirror array.. Nat Biotechnol.

[28] Eisen MB, Spellman PT, Brown PO, Botstein D (1998). Cluster analysis and display of genome-wide expression patterns.. Proc Natl Acad Sci U S A.

[29] Tamura K, Dudley J, Nei M, Kumar S (2007). MEGA4: Molecular Evolutionary Genetics Analysis (MEGA) software version 4.0.. Mol Biol Evol.

[30] Carver TJ, Rutherford KM, Berriman M, Rajandream MA, Barrell BG (2005). ACT: the Artemis Comparison Tool.. Bioinformatics.

[31] Letunic I, Doerks T, Bork P (2009). SMART 6: recent updates and new developments.. Nucleic Acids Res.

[32] Langille MG, Brinkman FS (2009). IslandViewer: an integrated interface for computational identification and visualization of genomic islands.. Bioinformatics.

[33] Roberts RJ, Vincze T, Posfai J, Macelis D (2010). REBASE–a database for DNA restriction and modification: enzymes, genes and genomes.. Nucleic Acids Res.

[34] Falush D, Stephens M, Pritchard JK (2003). Inference of population structure using multilocus genotype data: linked loci and correlated allele frequencies.. Genetics.

[35] Zhang W, Qi W, Albert TJ, Motiwala AS, Alland D (2006). Probing genomic diversity and evolution of *Escherichia coli* O157 by single nucleotide polymorphisms.. Genome Res.

[36] Mitchell TJ (2003). The pathogenesis of streptococcal infections: from tooth decay to meningitis.. Nat Rev Microbiol.

[37] Tettelin H, Nelson KE, Paulsen IT, Eisen JA, Read TD (2001). Complete genome sequence of a virulent isolate of *Streptococcus pneumoniae*.. Science.

[38] Langille MG, Hsiao WW, Brinkman FS (2010). Detecting genomic islands using bioinformatics approaches.. Nat Rev Microbiol.

[39] Vecht U, Wisselink HJ, Jellema ML, Smith HE (1991). Identification of two proteins associated with virulence of *Streptococcus suis* type 2.. Infect Immun.

[40] Takamatsu D, Nishino H, Ishiji T, Ishii J, Osaki M (2009). Genetic organization and preferential distribution of putative pilus gene clusters in *Streptococcus suis*.. Vet Microbiol.

[41] Vickerman MM, Iobst S, Jesionowski AM, Gill SR (2007). Genome-wide transcriptional changes in *Streptococcus gordonii* in response to competence signaling peptide.. J Bacteriol.

[42] Frees D, Savijoki K, Varmanen P, Ingmer H (2007). Clp ATPases and ClpP proteolytic complexes regulate vital biological processes in low GC, Gram-positive bacteria.. Mol Microbiol.

[43] Derre I, Rapoport G, Msadek T (1999). CtsR, a novel regulator of stress and heat shock response, controls clp and molecular chaperone gene expression in gram-positive bacteria.. Mol Microbiol.

[44] Nair S, Derre I, Msadek T, Gaillot O, Berche P (2000). CtsR controls class III heat shock gene expression in the human pathogen *Listeria monocytogenes*.. Mol Microbiol.

[45] Ibrahim YM, Kerr AR, Silva NA, Mitchell TJ (2005). Contribution of the ATP-dependent protease ClpCP to the autolysis and virulence of *Streptococcus pneumoniae*.. Infect Immun.

[46] Nair S, Milohanic E, Berche P (2000). ClpC ATPase is required for cell adhesion and invasion of *Listeria monocytogenes*.. Infect Immun.

[47] Murray NE, Daniel AS, Cowan GM, Sharp PM (1993). Conservation of motifs within the unusually variable polypeptide sequences of type I restriction and modification enzymes.. Mol Microbiol.

[48] Ando T, Wassenaar TM, Peek RM, Aras RA, Tschumi AI (2002). A *Helicobacter pylori* restriction endonuclease-replacing gene, hrgA, is associated with gastric cancer in Asian strains.. Cancer Res.

[49] Bart A, Pannekoek Y, Dankert J, van der Ende A (2001). NmeSI restriction-modification system identified by representational difference analysis of a hypervirulent *Neisseria meningitidis* strain.. Infect Immun.

[50] Pouillot F, Fayolle C, Carniel E (2007). A putative DNA adenine methyltransferase is involved in *Yersinia pseudotuberculosis* pathogenicity.. Microbiology.

[51] Praefcke GJ, McMahon HT (2004). The dynamin superfamily: universal membrane tubulation and fission molecules?. Nat Rev Mol Cell Biol.

[52] Labrie SJ, Samson JE Moineau S Bacteriophage resistance mechanisms.. Nat Rev Microbiol.

[53] Feil EJ, Smith JM, Enright MC, Spratt BG (2000). Estimating recombinational parameters in *Streptococcus pneumoniae* from multilocus sequence typing data.. Genetics.

[54] Tay CY, Mitchell H, Dong Q, Goh KL, Dawes IW (2009). Population structure of *Helicobacter pylori* among ethnic groups in Malaysia: recent acquisition of the bacterium by the Malay population.. BMC Microbiol.

[55] Lefebure T, Stanhope MJ (2007). Evolution of the core and pan-genome of *Streptococcus*: positive selection, recombination, and genome composition.. Genome Biol.

[56] Albert TJ, Dailidiene D, Dailide G, Norton JE, Kalia A (2005). Mutation discovery in bacterial genomes: metronidazole resistance in *Helicobacter pylori*.. Nat Methods.

[57] Beres SB, Richter EW, Nagiec MJ, Sumby P, Porcella SF (2006). Molecular genetic anatomy of inter- and intraserotype variation in the human bacterial pathogen group A *Streptococcus*.. Proc Natl Acad Sci U S A.

[58] Jackson SA, Mammel MK, Patel IR, Mays T, Albert TJ (2007). Interrogating genomic diversity of *E. coli* O157:H7 using DNA tiling arrays.. Forensic Sci Int.

[59] Maharjan RP, Gu C, Reeves PR, Sintchenko V, Gilbert GL (2008). Genome-wide analysis of single nucleotide polymorphisms in *Bordetella pertussis* using comparative genomic sequencing.. Res Microbiol.

[60] Leung AS, Tran V, Wu Z, Yu X, Alexander DC (2008). Novel genome polymorphisms in BCG vaccine strains and impact on efficacy.. BMC Genomics.

[61] Urban AE, Korbel JO, Selzer R, Richmond T, Hacker A (2006). High-resolution mapping of DNA copy alterations in human chromosome 22 using high-density tiling oligonucleotide arrays.. Proc Natl Acad Sci U S A.

[62] Baums CG, Valentin-Weigand P (2009). Surface-associated and secreted factors of *Streptococcus suis* in epidemiology, pathogenesis and vaccine development.. Anim Health Res Rev.

[63] Jiang H, Fan HJ, Lu CP (2009). Identification and distribution of putative virulent genes in strains of *Streptococcus suis* serotype 2.. Vet Microbiol.

[64] Haleis A, Alfa M, Gottschalk M, Bernard K, Ronald A (2009). Meningitis caused by *Streptococcus suis* serotype 14, North America.. Emerg Infect Dis.

[65] Kerdsin A, Oishi K, Sripakdee S, Boonkerd N, Polwichai P (2009). Clonal dissemination of human isolates of *Streptococcus suis* serotype 14 in Thailand.. J Med Microbiol.

[66] Mai NT, Hoa NT, Nga TV, Linh le D, Chau TT (2008). *Streptococcus suis* meningitis in adults in Vietnam.. Clin Infect Dis.

[67] Poggenborg R, Gaini S, Kjaeldgaard P, Christensen JJ (2008). *Streptococcus suis*: meningitis, spondylodiscitis and bacteraemia with a serotype 14 strain.. Scand J Infect Dis.

[68] Feng L, Reeves PR, Lan R, Ren Y, Gao C (2008). A recalibrated molecular clock and independent origins for the cholera pandemic clones.. PLoS One.

