# Supplementary material for: Identification of Genes and Genomic Islands Correlated with High Pathogenicity in Streptococcus suis Using Whole Genome Tilling Microarrays
Source: PLoS One. 2011 Mar 30;6(3):e17987. doi: 10.1371/journal.pone.0017987 (PMC3068143; doi:10.1371/journal.pone.0017987)
Supplement: Table S1 — Distribution of GZ1 genes in 31 test strains identified by microarray. (DOC) [file pone.0017987.s001.doc]

**Table S1 Presence/absence of GZ1 genes in 31 test strains identified by microarray.**

| **Gene** | | | | | **89-1591** | | **GX1** | | **11611** | | **31533** | | | **GZ2** | | **LN1** | | **P1/7** | | **SC84** | | **SC22** | | | **2651** | | **4961** | | **6407** | | **11538** | | **2524** | | | **14636** | | **22083** | | **4417** | | **12814** | | **8830** | | **10581** | | | **13730** | | **NCTC1046** | | **2726** | | **93A** | | **NT77** | | | **42A** | | **14A** | | **89-2479** | | **89-3576-3** | | **89-590** | **92-1400** | **Annotation** |
| --- | --- | --- | --- | --- | --- | --- | --- | --- | --- | --- | --- | --- | --- | --- | --- | --- | --- | --- | --- | --- | --- | --- | --- | --- | --- | --- | --- | --- | --- | --- | --- | --- | --- | --- | --- | --- | --- | --- | --- | --- | --- | --- | --- | --- | --- | --- | --- | --- | --- | --- | --- | --- | --- | --- | --- | --- | --- | --- | --- | --- | --- | --- | --- | --- | --- | --- | --- | --- | --- | --- |
| **SSGZ1_0001** | | | | | + | | + | | + | | + | | | + | | + | | + | | + | | + | | | + | | + | | + | | + | | + | | | + | | + | | + | | + | | + | | + | | | + | | + | | + | | + | | + | | | + | | + | | + | | + | | + | + | **chromosomal replication initiator protein,DnaA** |
| **SSGZ1_0002** | | | | | + | | + | | + | | + | | | + | | + | | + | | + | | + | | | + | | + | | + | | + | | + | | | + | | + | | + | | + | | + | | + | | | + | | + | | + | | + | | + | | | + | | + | | + | | + | | + | + | **DNA polymerase III, beta subunit** |
| **SSGZ1_0003** | | | | | + | | + | | + | | + | | | + | | + | | + | | + | | + | | | + | | + | | + | | + | | + | | | + | | + | | + | | + | | + | | + | | | + | | + | | + | | + | | + | | | + | | + | | + | | + | | + | + | **similar to putative transcription regulator** |
| **SSGZ1_0004** | | | | | + | | + | | + | | + | | | + | | + | | + | | + | | + | | | + | | + | | + | | + | | + | | | + | | + | | + | | + | | + | | + | | | + | | + | | + | | + | | + | | | + | | + | | + | | + | | + | + | **hypothetical protein** |
| **SSGZ1_0005** | | | | | + | | + | | + | | + | | | + | | + | | + | | + | | + | | | + | | + | | + | | + | | + | | | + | | + | | + | | + | | + | | + | | | + | | + | | + | | + | | + | | | + | | + | | + | | + | | + | + | **Helix-turn-helix motif** |
| **SSGZ1_0006** | | | | | + | | + | | + | | + | | | + | | + | | + | | + | | + | | | + | | + | | + | | + | | + | | | + | | + | | + | | + | | + | | + | | | + | | + | | + | | + | | + | | | + | | + | | + | | + | | + | + | **GTP-binding protein** |
| **SSGZ1_0007** | | | | | + | | + | | + | | + | | | + | | + | | + | | + | | + | | | + | | + | | + | | + | | + | | | + | | + | | + | | + | | + | | + | | | + | | + | | + | | + | | + | | | + | | + | | + | | + | | + | + | **Peptidyl-tRNA hydrolase** |
| **SSGZ1_0008** | | | | | + | | + | | + | | + | | | + | | + | | + | | + | | + | | | + | | + | | + | | + | | + | | | + | | + | | + | | + | | + | | + | | | + | | + | | + | | + | | + | | | + | | + | | + | | + | | + | + | **Transcription-repair coupling factor** |
| **SSGZ1_0009** | | | | | + | | + | | + | | + | | | + | | + | | + | | + | | + | | | + | | + | | + | | + | | + | | | + | | + | | + | | + | | + | | + | | | + | | + | | + | | + | | + | | | + | | + | | + | | + | | + | + | **RNA-binding S4** |
| **SSGZ1_0010** | | | | | + | | + | | + | | + | | | + | | + | | + | | + | | + | | | + | | + | | + | | + | | + | | | + | | + | | + | | + | | + | | + | | | + | | + | | + | | + | | + | | | + | | + | | + | | + | | + | + | **Septum formation initiator** |
| **SSGZ1_0011** | | | | | + | | + | | + | | + | | | + | | + | | + | | + | | + | | | + | | + | | + | | + | | + | | | + | | + | | + | | + | | + | | + | | | + | | + | | + | | + | | + | | | + | | + | | + | | + | | + | + | **hypothetical protein** |
| **SSGZ1_0012** | | | | | + | | + | | + | | + | | | + | | + | | + | | + | | + | | | + | | + | | + | | + | | + | | | + | | + | | + | | + | | + | | + | | | + | | + | | + | | + | | + | | | + | | + | | + | | + | | + | + | **beta-lactamase-like protein** |
| **SSGZ1_0013** | | | | | + | | + | | + | | + | | | + | | + | | + | | + | | + | | | + | | + | | + | | + | | + | | | + | | + | | + | | + | | + | | + | | | + | | + | | + | | + | | + | | | + | | + | | + | | + | | + | + | **PP-loop** |
| **SSGZ1_0014** | | | | | + | | + | | + | | + | | | + | | + | | + | | + | | + | | | + | | + | | + | | + | | + | | | + | | + | | + | | + | | + | | + | | | + | | + | | + | | + | | + | | | + | | + | | + | | + | | + | + | **Hypoxanthine phosphoribosyl transferase** |
| **SSGZ1_0015** | | | | | + | | + | | + | | + | | | + | | + | | + | | + | | + | | | + | | + | | + | | + | | + | | | + | | + | | + | | + | | + | | + | | | + | | + | | + | | + | | + | | | + | | + | | + | | + | | + | + | **Peptidase M41, FtsH** |
| **SSGZ1_0016** | | | | | + | | + | | + | | + | | | + | | + | | + | | + | | + | | | + | | + | | + | | + | | + | | | + | | + | | + | | + | | + | | + | | | + | | + | | + | | + | | + | | | + | | + | | + | | + | | + | + | **tRNA-Ser** |
| **SSGZ1_0017** | | | | | + | | + | | + | | + | | | + | | + | | + | | + | | + | | | + | | + | | + | | + | | + | | | + | | + | | + | | + | | + | | + | | | + | | + | | + | | + | | + | | | + | | + | | + | | + | | + | + | **putative cell shape-determining protein MreC** |
| **SSGZ1_0018** | | | | | + | | + | | + | | + | | | + | | + | | + | | + | | + | | | + | | + | | + | | + | | + | | | + | | + | | + | | + | | + | | + | | | + | | + | | + | | + | | + | | | + | | + | | + | | + | | + | + | **rod shape-determining protein MreD** |
| **SSGZ1_0019** | | | | | + | | + | | + | | + | | | + | | + | | + | | + | | + | | | + | | + | | + | | + | | + | | | + | | + | | + | | + | | + | | + | | | + | | + | | + | | + | | + | | | + | | + | | + | | + | | + | + | **CHAP** |
| **SSGZ1_0020** | | | | | + | | + | | + | | + | | | + | | + | | + | | + | | + | | | + | | + | | + | | + | | + | | | + | | + | | + | | + | | + | | + | | | + | | + | | + | | + | | + | | | + | | + | | + | | + | | + | + | **Ribose-phosphate pyrophosphokinase** |
| **SSGZ1_0021** | | | | | + | | + | | + | | + | | | + | | + | | + | | + | | + | | | + | | + | | + | | + | | + | | | + | | + | | + | | + | | + | | + | | | + | | + | | + | | + | | + | | | + | | + | | + | | + | | + | + | **aromatic amino acid aminotransferase** |
| **SSGZ1_0022** | | | | | + | | + | | + | | + | | | + | | + | | + | | + | | + | | | + | | + | | + | | + | | + | | | + | | + | | + | | + | | + | | + | | | + | | + | | + | | + | | + | | | + | | + | | + | | + | | + | + | **Recombination protein O** |
| **SSGZ1_0023** | | | | | + | | + | | + | | + | | | + | | + | | + | | + | | + | | | + | | + | | + | | + | | + | | | + | | + | | + | | + | | + | | + | | | + | | + | | + | | + | | + | | | + | | + | | + | | + | | + | + | **Fatty acid synthesis plsX protein** |
| **SSGZ1_0024** | | | | | + | | + | | + | | + | | | + | | + | | + | | + | | + | | | + | | + | | + | | + | | + | | | + | | + | | + | | + | | + | | + | | | + | | + | | + | | + | | + | | | + | | + | | + | | + | | + | + | **similar to acyl carrier protein** |
| **SSGZ1_0025** | | | | | + | | + | | + | | + | | | + | | + | | + | | + | | + | | | + | | + | | + | | + | | + | | | + | | + | | + | | + | | + | | + | | | + | | + | | + | | + | | + | | | + | | + | | + | | + | | + | + | **SAICAR synthetase** |
| **SSGZ1_0026** | | | | | + | | + | | + | | + | | | + | | + | | + | | + | | + | | | + | | + | | + | | + | | + | | | + | | + | | + | | + | | + | | + | | | + | | + | | + | | + | | + | | | + | | + | | + | | + | | + | + | **Phosphoribosylformylglycinamidine synthase,** |
| **SSGZ1_0027** | | | | | + | | + | | + | | + | | | + | | + | | + | | + | | + | | | + | | + | | + | | + | | + | | | + | | + | | + | | + | | + | | + | | | + | | + | | + | | + | | + | | | + | | + | | + | | + | | + | + | **Amidophosphoribosyl transferase** |
| **SSGZ1_0028** | | | | | + | | + | | + | | + | | | + | | + | | + | | + | | + | | | + | | + | | + | | + | | + | | | + | | + | | + | | + | | + | | + | | | + | | + | | + | | + | | + | | | + | | + | | + | | + | | + | + | **Phosphoribosylformylglycinamidine cyclo-ligase** |
| **SSGZ1_0029** | | | | | + | | + | | + | | + | | | + | | + | | + | | + | | + | | | - | | + | | + | | + | | + | | | + | | + | | + | | + | | + | | + | | | + | | + | | + | | + | | + | | | + | | + | | + | | + | | + | + | **phosphoribosyl glycinamide transformylase-N** |
| **SSGZ1_0030** | | | | | + | | + | | + | | + | | | + | | + | | + | | + | | + | | | + | | + | | + | | + | | + | | | + | | + | | + | | + | | + | | + | | | + | | + | | + | | + | | + | | | + | | + | | + | | + | | + | + | **Bifunctional** |
| **SSGZ1_0031** | | | | | + | | + | | + | | + | | | + | | + | | + | | + | | + | | | + | | + | | + | | + | | + | | | + | | + | | + | | + | | + | | + | | | + | | + | | + | | + | | + | | | + | | + | | + | | + | | + | + | **Phosphoribosylglycinamide synthetase** |
| **SSGZ1_0032** | | | | | + | | + | | + | | + | | | + | | + | | + | | + | | + | | | + | | + | | + | | + | | + | | | + | | + | | + | | + | | + | | + | | | + | | + | | + | | + | | + | | | + | | + | | + | | + | | + | + | **1-(5-Phosphoribosyl)-5-amino-4-imidazole-** |
| **SSGZ1_0033** | | | | | + | | + | | + | | + | | | + | | + | | + | | + | | + | | | + | | + | | + | | + | | + | | | + | | + | | + | | + | | + | | + | | | + | | + | | + | | + | | + | | | + | | + | | + | | + | | + | + | **Phosphoribosylaminoimidazole carboxylase, ATPase** |
| **SSGZ1_0034** | | | | | - | | + | | - | | + | | | + | | + | | + | | + | | + | | | - | | - | | - | | + | | - | | | + | | + | | + | | + | | - | | + | | | + | | + | | + | | - | | - | | | + | | + | | - | | + | | + | + | **hypothetical protein** |
| **SSGZ1_0035** | | | | | + | | + | | - | | + | | | + | | + | | + | | + | | + | | | + | | + | | + | | + | | + | | | + | | + | | + | | + | | + | | + | | | + | | + | | + | | + | | + | | | + | | + | | - | | + | | + | + | **hypothetical protein** |
| **SSGZ1_0036** | | | | | + | | + | | + | | + | | | + | | + | | + | | + | | + | | | + | | + | | + | | + | | + | | | + | | + | | + | | + | | + | | + | | | + | | + | | + | | + | | + | | | + | | + | | + | | + | | + | + | **Adenylosuccinate lyase** |
| **SSGZ1_0037** | | | | | + | | + | | + | | + | | | + | | + | | + | | + | | + | | | + | | + | | + | | + | | + | | | + | | + | | + | | + | | + | | + | | | + | | + | | + | | + | | + | | | + | | + | | + | | + | | + | + | **hypothetical protein** |
| **SSGZ1_0038** | | | | | + | | + | | + | | + | | | + | | + | | + | | + | | + | | | + | | + | | + | | + | | + | | | + | | + | | + | | + | | + | | - | | | + | | + | | + | | + | | + | | | + | | + | | + | | + | | + | + | **Amino acid transporter** |
| **SSGZ1_0039** | | | | | + | | + | | - | | + | | | + | | + | | + | | + | | + | | | - | | + | | + | | + | | - | | | + | | + | | + | | - | | - | | - | | | + | | + | | + | | + | | + | | | + | | + | | + | | + | | + | - | **Abortive infection protein** |
| **SSGZ1_0040** | | | | | + | | + | | - | | + | | | + | | + | | + | | + | | + | | | - | | + | | + | | + | | - | | | + | | + | | + | | - | | - | | + | | | + | | + | | + | | + | | + | | | + | | + | | + | | + | | + | - | **hypothetical protein** |
| **SSGZ1_0041** | | | | | + | | + | | + | | + | | | + | | + | | + | | + | | + | | | + | | + | | + | | + | | + | | | + | | + | | + | | + | | + | | + | | | + | | + | | + | | + | | + | | | + | | + | | + | | + | | + | + | **ABC-type multidrug transport system ATPase** |
| **SSGZ1_0042** | | | | | + | | + | | + | | + | | | + | | + | | + | | + | | + | | | + | | + | | + | | + | | + | | | - | | + | | + | | - | | - | | - | | | + | | + | | + | | + | | + | | | + | | + | | + | | + | | + | - | **hypothetical protein** |
| **SSGZ1_0043** | | | | | + | | + | | + | | + | | | + | | + | | + | | + | | + | | | + | | + | | + | | + | | + | | | - | | + | | + | | - | | - | | - | | | + | | + | | + | | + | | + | | | + | | + | | + | | + | | + | - | **ABC transporter,ATP binding protein** |
| **SSGZ1_0044** | | | | | + | | + | | + | | + | | | + | | + | | + | | + | | + | | | + | | + | | + | | + | | + | | | + | | + | | + | | + | | - | | + | | | + | | + | | + | | + | | + | | | + | | + | | + | | + | | + | - | **hypothetical protein** |
| **SSGZ1_0045** | | | | | + | | + | | + | | + | | | + | | + | | + | | + | | + | | | + | | + | | + | | + | | + | | | + | | + | | + | | + | | + | | + | | | + | | + | | + | | + | | + | | | + | | + | | + | | + | | + | + | **Holliday junction DNA helicase RuvB** |
| **SSGZ1_0046** | | | | | + | | + | | - | | + | | | + | | + | | + | | + | | + | | | - | | + | | - | | - | | - | | | + | | + | | - | | + | | + | | + | | | + | | + | | + | | - | | - | | | - | | + | | - | | + | | + | + | **GCN5-related N-acetyltransferase** |
| **SSGZ1_0047** | | | | | + | | + | | + | | + | | | + | | + | | + | | + | | + | | | + | | + | | + | | + | | + | | | + | | + | | + | | + | | + | | + | | | + | | + | | + | | + | | + | | | + | | + | | + | | + | | + | + | **HAD-superfamily hydrolase, subfamily IA, variant** |
| **SSGZ1_0048** | | | | | + | | + | | + | | + | | | + | | + | | + | | + | | + | | | + | | + | | + | | + | | + | | | + | | + | | + | | + | | + | | + | | | + | | + | | + | | + | | + | | | + | | + | | + | | + | | + | + | **putative transcriptional regulator** |
| **SSGZ1_0049** | | | | | + | | + | | + | | + | | | + | | + | | + | | + | | + | | | + | | + | | + | | + | | + | | | + | | - | | + | | + | | + | | + | | | + | | + | | + | | + | | + | | | + | | + | | + | | + | | + | + | **transposase IS116/IS110/IS902** |
| **SSGZ1_0050** | | | | | + | | + | | + | | + | | | + | | + | | + | | + | | + | | | + | | - | | + | | + | | - | | | + | | + | | + | | - | | - | | + | | | + | | + | | + | | + | | + | | | + | | + | | + | | - | | + | + | **Transposase, IS4** |
| **SSGZ1_0051** | | | | | + | | + | | + | | + | | | + | | + | | + | | + | | + | | | + | | + | | + | | + | | + | | | + | | + | | + | | + | | + | | + | | | + | | + | | + | | + | | + | | | + | | + | | + | | + | | + | + | **putative dihydrofolate synthetase** |
| **SSGZ1_0052** | | | | | + | | + | | + | | + | | | + | | + | | + | | + | | + | | | + | | + | | + | | + | | + | | | + | | + | | + | | + | | + | | + | | | + | | + | | + | | + | | + | | | + | | + | | + | | + | | + | + | **hypothetical protein** |
| **SSGZ1_0053** | | | | | + | | + | | + | | + | | | + | | + | | + | | + | | + | | | + | | + | | + | | + | | + | | | + | | + | | + | | + | | + | | + | | | + | | + | | + | | + | | + | | | + | | + | | + | | + | | + | + | **Heme/copper-type cytochrome/quinol oxidase,** |
| **SSGZ1_0054** | | | | | + | | + | | + | | + | | | + | | + | | + | | + | | + | | | + | | + | | + | | + | | + | | | + | | + | | + | | + | | + | | + | | | + | | + | | + | | + | | + | | | + | | + | | + | | + | | + | + | **hypothetical protein** |
| **SSGZ1_0055** | | | | | + | | + | | + | | + | | | + | | + | | + | | + | | + | | | + | | + | | + | | + | | + | | | + | | + | | + | | + | | + | | + | | | + | | + | | + | | + | | + | | | + | | + | | + | | + | | + | + | **putative mismatch repair protein HexB** |
| **SSGZ1_0056** | | | | | + | | + | | + | | + | | | + | | + | | + | | + | | + | | | + | | + | | + | | + | | + | | | + | | + | | + | | + | | + | | + | | | + | | + | | + | | + | | + | | | + | | + | | + | | + | | + | + | **Holliday junction DNA helicase RuvA** |
| **SSGZ1_0057** | | | | | + | | + | | + | | + | | | + | | + | | + | | + | | + | | | + | | + | | + | | + | | + | | | + | | + | | + | | + | | + | | + | | | + | | + | | + | | + | | + | | | + | | + | | + | | + | | + | + | **DNA-3-methyladenine glycosylase I** |
| **SSGZ1_0058** | | | | | + | | + | | + | | + | | | + | | + | | + | | + | | + | | | + | | + | | + | | + | | + | | | + | | + | | + | | + | | + | | + | | | + | | + | | + | | + | | + | | | + | | + | | + | | + | | + | + | **putative competence and damage inducible protein** |
| **SSGZ1_0059** | | | | | + | | + | | + | | + | | | + | | + | | + | | + | | + | | | + | | + | | + | | + | | + | | | + | | + | | + | | + | | + | | + | | | + | | + | | + | | + | | + | | | + | | + | | + | | + | | + | + | **homologous recombination factor RecA** |
| **SSGZ1_0060** | | | | | + | | + | | + | | + | | | + | | + | | + | | + | | + | | | + | | + | | + | | + | | + | | | + | | + | | + | | + | | + | | + | | | + | | + | | + | | + | | + | | | + | | + | | + | | + | | + | + | **putative Arsenate reductase** |
| **SSGZ1_0061** | | | | | + | | + | | + | | + | | | + | | + | | + | | + | | + | | | + | | + | | + | | + | | + | | | + | | + | | + | | + | | + | | + | | | + | | + | | + | | + | | + | | | + | | + | | + | | + | | + | + | **protein of unknown function DUF965** |
| **SSGZ1_0062** | | | | | + | | + | | + | | + | | | + | | + | | + | | + | | + | | | + | | + | | + | | + | | + | | | + | | + | | + | | + | | + | | + | | | + | | + | | + | | + | | + | | | + | | + | | + | | + | | + | + | **Resolvase, holliday junction-type, YqgF-like** |
| **SSGZ1_0063** | | | | | + | | + | | + | | + | | | + | | + | | + | | + | | + | | | + | | + | | + | | + | | + | | | + | | + | | + | | + | | + | | + | | | + | | + | | + | | + | | + | | | + | | + | | + | | + | | + | + | **Protein of unknown function DUF1292** |
| **SSGZ1_0064** | | | | | + | | + | | + | | + | | | + | | + | | + | | + | | + | | | + | | + | | - | | - | | - | | | - | | + | | - | | + | | + | | + | | | + | | + | | - | | - | | - | | | - | | + | | - | | - | | + | + | **protein of unknown function DUF925** |
| **SSGZ1_0065** | | | | | + | | + | | + | | + | | | + | | + | | + | | + | | + | | | + | | + | | - | | - | | - | | | - | | + | | + | | + | | + | | + | | | + | | + | | + | | - | | - | | | - | | + | | - | | + | | + | + | **competence-specific global transcription** |
| **SSGZ1_0066** | | | | | + | | + | | + | | + | | | + | | + | | + | | + | | + | | | + | | + | | + | | + | | + | | | + | | + | | + | | + | | + | | + | | | + | | + | | + | | + | | + | | | + | | + | | + | | + | | + | + | **ribosomal protein S10** |
| **SSGZ1_0067** | | | | | + | | + | | + | | + | | | + | | + | | + | | + | | + | | | + | | + | | + | | + | | + | | | + | | + | | + | | + | | + | | + | | | + | | + | | + | | + | | + | | | + | | + | | + | | + | | + | + | **'ribosomal protein L3** |
| **SSGZ1_0068** | | | | | + | | + | | + | | + | | | + | | + | | + | | + | | + | | | + | | + | | + | | + | | + | | | + | | + | | + | | + | | + | | + | | | + | | + | | + | | + | | + | | | + | | + | | + | | + | | + | + | **ribosomal protein L4** |
| **SSGZ1_0069** | | | | | + | | + | | + | | + | | | + | | + | | + | | + | | + | | | + | | + | | + | | + | | + | | | + | | + | | + | | + | | + | | + | | | + | | + | | + | | + | | + | | | + | | + | | + | | + | | + | + | **ribosomal protein L23** |
| **SSGZ1_0070** | | | | | + | | + | | + | | + | | | + | | + | | + | | + | | + | | | + | | + | | + | | + | | + | | | + | | + | | + | | + | | + | | + | | | + | | + | | + | | + | | + | | | + | | + | | + | | + | | + | + | **'ribosomal protein L2** |
| **SSGZ1_0071** | | | | | + | | + | | + | | + | | | + | | + | | + | | + | | + | | | + | | + | | + | | + | | + | | | + | | + | | + | | + | | + | | + | | | + | | + | | + | | + | | + | | | + | | + | | + | | + | | + | + | **'ribosomal protein S19** |
| **SSGZ1_0072** | | | | | + | | + | | + | | + | | | + | | + | | + | | + | | + | | | + | | + | | + | | + | | + | | | + | | + | | + | | + | | + | | + | | | + | | + | | + | | + | | + | | | + | | + | | + | | + | | + | + | **ribosomal protein L22** |
| **SSGZ1_0073** | | | | | + | | + | | + | | + | | | + | | + | | + | | + | | + | | | + | | + | | + | | + | | + | | | + | | + | | + | | + | | + | | + | | | + | | + | | + | | + | | + | | | + | | + | | + | | + | | + | + | **ribosomal protein S3** |
| **SSGZ1_0074** | | | | | + | | + | | + | | + | | | + | | + | | + | | + | | + | | | + | | + | | + | | + | | + | | | + | | + | | + | | + | | + | | + | | | + | | + | | + | | + | | + | | | + | | + | | + | | + | | + | + | **ribosomal protein L16** |
| **SSGZ1_0075** | | | | | + | | + | | + | | + | | | + | | + | | + | | + | | + | | | + | | + | | + | | + | | + | | | + | | + | | + | | + | | + | | + | | | + | | + | | + | | + | | + | | | + | | + | | + | | + | | + | + | **50s ribosomal protein L29** |
| **SSGZ1_0076** | | | | | + | | + | | + | | + | | | + | | + | | + | | + | | + | | | + | | + | | + | | + | | + | | | + | | + | | + | | + | | + | | + | | | + | | + | | + | | + | | + | | | + | | + | | + | | + | | + | + | **ribosomal protein S17** |
| **SSGZ1_0077** | | | | | + | | + | | + | | + | | | + | | + | | + | | + | | + | | | + | | + | | + | | + | | + | | | + | | + | | + | | + | | + | | + | | | + | | + | | + | | + | | + | | | + | | + | | + | | + | | + | + | **ribosomal protein L14** |
| **SSGZ1_0078** | | | | | + | | + | | + | | + | | | + | | + | | + | | + | | + | | | + | | + | | + | | + | | + | | | + | | + | | + | | + | | + | | + | | | + | | + | | + | | + | | + | | | + | | + | | + | | + | | + | + | **ribosomal protein L24** |
| **SSGZ1_0079** | | | | | + | | + | | + | | + | | | + | | + | | + | | + | | + | | | + | | + | | + | | + | | + | | | + | | + | | + | | + | | + | | + | | | + | | + | | + | | + | | + | | | + | | + | | + | | + | | + | + | **ribosomal protein L5** |
| **SSGZ1_0080** | | | | | + | | + | | + | | + | | | + | | + | | + | | + | | + | | | + | | + | | + | | + | | + | | | + | | + | | + | | + | | + | | + | | | + | | + | | + | | + | | + | | | + | | + | | + | | + | | + | + | **ribosomal protein S14** |
| **SSGZ1_0081** | | | | | + | | + | | + | | + | | | + | | + | | + | | + | | + | | | + | | + | | + | | + | | + | | | + | | + | | + | | + | | + | | + | | | + | | + | | + | | + | | + | | | + | | + | | + | | + | | + | + | **ribosomal protein S8** |
| **SSGZ1_0082** | | | | | + | | + | | + | | + | | | + | | + | | + | | + | | + | | | + | | + | | + | | + | | + | | | + | | + | | + | | + | | + | | + | | | + | | + | | + | | + | | + | | | + | | + | | + | | + | | + | + | **hypothetical protein** |
| **SSGZ1_0083** | | | | | + | | + | | + | | + | | | + | | + | | + | | + | | + | | | + | | + | | + | | + | | + | | | + | | + | | + | | + | | + | | + | | | + | | + | | + | | + | | + | | | + | | + | | + | | + | | + | + | **ribosomal protein L6** |
| **SSGZ1_0084** | | | | | + | | + | | + | | + | | | + | | + | | + | | + | | + | | | + | | + | | + | | + | | + | | | + | | + | | + | | + | | + | | + | | | + | | + | | + | | + | | + | | | + | | + | | + | | + | | + | + | **ribosomal protein L18** |
| **SSGZ1_0085** | | | | | + | | + | | + | | + | | | + | | + | | + | | + | | + | | | + | | + | | + | | + | | + | | | + | | + | | + | | + | | + | | + | | | + | | + | | + | | + | | + | | | + | | + | | + | | + | | + | + | **ribosomal protein S5** |
| **SSGZ1_0086** | | | | | + | | + | | + | | + | | | + | | + | | + | | + | | + | | | + | | + | | - | | + | | + | | | + | | + | | + | | + | | + | | + | | | + | | + | | + | | + | | + | | | + | | + | | + | | + | | + | + | **ribosomal protein L30** |
| **SSGZ1_0087** | | | | | + | | + | | + | | + | | | + | | + | | + | | + | | + | | | + | | + | | + | | + | | + | | | + | | + | | + | | + | | + | | + | | | + | | + | | + | | + | | + | | | + | | + | | + | | + | | + | + | **ribosomal protein L15** |
| **SSGZ1_0088** | | | | | + | | + | | + | | + | | | + | | + | | + | | + | | + | | | + | | + | | + | | + | | + | | | + | | + | | + | | + | | + | | + | | | + | | + | | + | | + | | + | | | + | | + | | + | | + | | + | + | **putative preprotein translocase SecY protein** |
| **SSGZ1_0089** | | | | | + | | + | | + | | + | | | + | | + | | + | | + | | + | | | + | | + | | + | | + | | + | | | + | | + | | + | | + | | + | | + | | | + | | + | | + | | + | | + | | | + | | + | | + | | + | | + | + | **adenylate kinase** |
| **SSGZ1_0090** | | | | | + | | + | | + | | + | | | + | | + | | + | | + | | + | | | + | | + | | + | | + | | + | | | + | | + | | + | | + | | + | | + | | | + | | + | | + | | + | | + | | | + | | + | | + | | + | | + | + | **Translation initiation factor IF-1** |
| **SSGZ1_0091** | | | | | + | | + | | + | | + | | | + | | + | | + | | + | | + | | | + | | + | | + | | + | | + | | | + | | + | | + | | + | | + | | + | | | + | | + | | + | | + | | + | | | + | | + | | + | | + | | + | + | **ribosomal protein S13** |
| **SSGZ1_0092** | | | | | + | | + | | + | | + | | | + | | + | | + | | + | | + | | | + | | + | | + | | + | | + | | | + | | + | | + | | + | | + | | + | | | + | | + | | + | | + | | + | | | + | | + | | + | | + | | + | + | **ribosomal protein S11** |
| **SSGZ1_0093** | | | | | + | | + | | + | | + | | | + | | + | | + | | + | | + | | | + | | + | | + | | + | | + | | | + | | + | | + | | + | | + | | + | | | + | | + | | + | | + | | + | | | + | | + | | + | | + | | + | + | **DNA-directed RNA polymerase alpha subunit** |
| **SSGZ1_0094** | | | | | + | | + | | + | | + | | | + | | + | | + | | + | | + | | | + | | + | | + | | + | | + | | | + | | + | | + | | + | | + | | + | | | + | | + | | + | | + | | + | | | + | | + | | + | | + | | + | + | **tRNA-Leu** |
| **SSGZ1_0095** | | | | | - | | + | | + | | + | | | + | | + | | + | | + | | + | | | + | | + | | - | | - | | - | | | + | | + | | - | | + | | + | | + | | | - | | - | | - | | + | | + | | | + | | + | | + | | - | | - | - | **Integrase, catalytic core, phage** |
| **SSGZ1_0096** | | | | | - | | + | | + | | + | | | + | | + | | + | | + | | + | | | + | | + | | - | | - | | - | | | + | | + | | - | | + | | + | | - | | | - | | - | | - | | + | | + | | | + | | + | | + | | - | | - | - | **hypothetical protein** |
| **SSGZ1_0097** | | | | | - | | + | | + | | + | | | + | | + | | + | | + | | + | | | + | | + | | + | | - | | - | | | + | | - | | - | | + | | + | | - | | | - | | - | | - | | + | | + | | | + | | + | | + | | - | | - | - | **Replication initiation factor** |
| **SSGZ1_0098** | | | | | - | | + | | + | | + | | | + | | + | | + | | + | | + | | | + | | + | | + | | - | | - | | | + | | - | | - | | + | | + | | - | | | - | | - | | - | | + | | + | | | + | | + | | + | | - | | - | - | **hypothetical protein** |
| **SSGZ1_0099** | | | | | - | | + | | + | | + | | | + | | + | | + | | + | | + | | | + | | - | | - | | - | | - | | | + | | - | | - | | + | | - | | - | | | - | | + | | - | | + | | + | | | - | | + | | + | | - | | - | - | **Cell divisionFtsK/SpoIIIE** |
| **SSGZ1_0100** | | | | | - | | + | | + | | + | | | + | | + | | + | | + | | + | | | + | | - | | + | | - | | - | | | + | | - | | - | | + | | - | | - | | | - | | - | | - | | + | | + | | | + | | + | | + | | - | | - | - | **hypothetical protein** |
| **SSGZ1_0101** | | | | | + | | + | | + | | + | | | + | | + | | + | | + | | + | | | + | | + | | + | | + | | - | | | + | | + | | - | | + | | + | | + | | | + | | - | | + | | + | | + | | | + | | + | | + | | + | | + | - | **hypothetical protein** |
| **SSGZ1_0102** | | | | | + | | + | | + | | + | | | + | | + | | + | | + | | + | | | + | | + | | + | | + | | + | | | + | | + | | + | | + | | + | | + | | | + | | + | | + | | + | | + | | | + | | + | | + | | + | | + | + | **hypothetical protein** |
| **SSGZ1_0103** | | | | | + | | + | | + | | + | | | + | | + | | + | | + | | + | | | + | | + | | + | | + | | + | | | + | | + | | + | | + | | + | | + | | | + | | + | | + | | + | | + | | | + | | + | | + | | + | | + | + | **hypothetical protein** |
| **SSGZ1_0104** | | | | | + | | + | | + | | + | | | + | | + | | + | | + | | + | | | + | | + | | + | | + | | + | | | + | | + | | + | | + | | + | | + | | | + | | + | | + | | + | | + | | | + | | + | | + | | + | | + | + | **hypothetical protein** |
| **SSGZ1_0105** | | | | | + | | + | | + | | + | | | + | | + | | + | | + | | + | | | + | | + | | + | | + | | + | | | + | | + | | + | | + | | + | | + | | | + | | + | | + | | + | | + | | | + | | + | | + | | + | | + | + | **adcR, transcriptional repressor for** |
| **SSGZ1_0106** | | | | | + | | + | | + | | + | | | + | | + | | + | | + | | + | | | + | | + | | + | | + | | + | | | + | | + | | + | | + | | + | | + | | | + | | + | | + | | + | | + | | | + | | + | | + | | + | | + | + | **adcC, ABC transporter ATP-binding domain - Zinc** |
| **SSGZ1_0107** | | | | | + | | + | | + | | + | | | + | | + | | + | | + | | + | | | + | | + | | + | | + | | + | | | + | | + | | + | | + | | + | | + | | | + | | + | | + | | + | | + | | | + | | + | | + | | + | | + | + | **adcB, ABC transporter membrane-spanning permease** |
| **SSGZ1_0108** | | | | | + | | + | | + | | + | | | + | | + | | + | | + | | + | | | + | | + | | + | | + | | + | | | + | | + | | + | | + | | + | | + | | | + | | + | | + | | + | | + | | | + | | + | | + | | + | | + | + | **zinc ABC transporter, zinc-binding lipoprotein** |
| **SSGZ1_0109** | | | | | + | | + | | + | | + | | | + | | + | | + | | + | | + | | | + | | + | | + | | + | | + | | | + | | + | | + | | + | | + | | + | | | + | | + | | + | | + | | + | | | + | | + | | + | | + | | + | + | **transcriptional repressor CopY** |
| **SSGZ1_0110** | | | | | + | | + | | + | | + | | | + | | + | | + | | + | | + | | | + | | + | | + | | + | | + | | | + | | + | | + | | + | | + | | + | | | + | | + | | + | | + | | + | | | + | | + | | + | | + | | + | + | **hypothetical protein** |
| **SSGZ1_0111** | | | | | + | | + | | + | | + | | | + | | + | | + | | + | | + | | | + | | + | | + | | + | | + | | | + | | + | | + | | + | | + | | + | | | + | | + | | + | | + | | + | | | + | | + | | + | | + | | + | + | **Heavy metal transport/detoxification protein** |
| **SSGZ1_0112** | | | | | + | | + | | + | | + | | | + | | + | | + | | + | | + | | | + | | + | | + | | + | | + | | | + | | + | | + | | + | | + | | + | | | + | | + | | + | | + | | + | | | + | | + | | + | | + | | + | + | **Histidine triad (HIT) protein** |
| **SSGZ1_0113** | | | | | + | | + | | + | | + | | | + | | + | | + | | + | | + | | | + | | + | | + | | + | | + | | | + | | + | | + | | + | | + | | + | | | + | | + | | + | | + | | + | | | + | | + | | + | | + | | + | + | **Tyrosyl-tRNA synthetase** |
| **SSGZ1_0114** | | | | | + | | + | | + | | + | | | + | | + | | + | | + | | + | | | + | | + | | + | | + | | + | | | + | | + | | + | | + | | + | | + | | | + | | + | | + | | + | | + | | | + | | + | | + | | + | | + | + | **Glycosyl transferase, family** |
| **SSGZ1_0115** | | | | | + | | + | | + | | + | | | + | | + | | + | | + | | + | | | + | | + | | + | | + | | + | | | + | | + | | + | | + | | + | | + | | | + | | + | | + | | + | | + | | | + | | + | | + | | + | | + | + | **DNA-directed RNA polymerase, beta subunit** |
| **SSGZ1_0116** | | | | | + | | + | | + | | + | | | + | | + | | + | | + | | + | | | + | | + | | + | | + | | + | | | + | | + | | + | | + | | + | | + | | | + | | + | | + | | + | | + | | | + | | + | | + | | + | | + | + | **DNA-directed RNA polymerase, beta subunit** |
| **SSGZ1_0117** | | | | | + | | + | | + | | + | | | + | | + | | + | | + | | + | | | + | | + | | + | | + | | + | | | + | | + | | + | | + | | + | | + | | | + | | + | | + | | + | | + | | | + | | + | | + | | + | | + | + | **Protein of unknown function DUF1033** |
| **SSGZ1_0118** | | | | | + | | + | | + | | + | | | + | | + | | + | | + | | + | | | + | | + | | + | | + | | + | | | + | | + | | + | | + | | + | | + | | | + | | + | | + | | + | | + | | | + | | + | | + | | + | | + | + | **hypothetical protein** |
| **SSGZ1_0119** | | | | | + | | + | | + | | + | | | + | | + | | + | | + | | + | | | + | | + | | + | | + | | + | | | + | | + | | + | | + | | + | | + | | | + | | + | | + | | + | | + | | | + | | + | | + | | + | | + | + | **Peptidase U61, LD-carboxypeptidase A** |
| **SSGZ1_0120** | | | | | + | | + | | + | | + | | | + | | + | | + | | + | | + | | | + | | + | | + | | + | | + | | | + | | + | | + | | + | | + | | + | | | + | | + | | + | | + | | + | | | + | | + | | + | | + | | + | + | **type II secretion system protein E** |
| **SSGZ1_0121** | | | | | + | | + | | + | | + | | | + | | + | | + | | + | | + | | | + | | + | | + | | + | | + | | | + | | + | | + | | + | | + | | + | | | + | | + | | + | | + | | + | | | + | | + | | + | | + | | + | + | **type II secretion system protein F** |
| **SSGZ1_0122** | | | | | + | | + | | + | | + | | | + | | + | | + | | + | | + | | | + | | + | | + | | + | | + | | | + | | + | | + | | + | | + | | + | | | + | | + | | + | | + | | + | | | + | | + | | + | | + | | + | + | **competence protein** |
| **SSGZ1_0123** | | | | | + | | + | | + | | + | | | + | | + | | + | | + | | + | | | + | | + | | + | | + | | + | | | - | | + | | + | | + | | + | | + | | | + | | + | | + | | + | | + | | | + | | + | | + | | + | | + | + | **Type II secretory pathway, pseudopilin PulG** |
| **SSGZ1_0124** | | | | | + | | + | | + | | + | | | + | | + | | + | | + | | + | | | + | | + | | + | | + | | + | | | + | | + | | + | | + | | + | | + | | | + | | + | | + | | + | | + | | | + | | + | | + | | + | | + | + | **Type II secretory pathway, pseudopilin PulG** |
| **SSGZ1_0125** | | | | | + | | + | | + | | + | | | + | | + | | + | | + | | + | | | + | | + | | + | | + | | + | | | + | | + | | + | | + | | + | | + | | | + | | + | | + | | + | | + | | | + | | + | | + | | + | | + | + | **competence protein ComGF** |
| **SSGZ1_0126** | | | | | + | | + | | + | | + | | | + | | + | | + | | + | | + | | | + | | + | | + | | + | | + | | | + | | + | | + | | + | | + | | + | | | + | | + | | + | | + | | + | | | + | | + | | + | | + | | + | + | **conserved hypothetical protein** |
| **SSGZ1_0127** | | | | | + | | + | | + | | + | | | + | | + | | + | | + | | + | | | + | | + | | + | | + | | + | | | + | | + | | + | | + | | + | | + | | | + | | + | | + | | + | | + | | | + | | + | | + | | + | | + | + | **Adenine-specific DNA methylase** |
| **SSGZ1_0128** | | | | | + | | + | | + | | + | | | + | | + | | + | | + | | + | | | + | | + | | + | | + | | + | | | + | | + | | + | | + | | + | | + | | | + | | + | | + | | + | | + | | | + | | + | | + | | + | | + | + | **acetate kinase** |
| **SSGZ1_0129** | | | | | + | | + | | + | | + | | | + | | + | | + | | + | | + | | | + | | + | | + | | + | | + | | | + | | + | | + | | + | | + | | + | | | + | | + | | + | | + | | + | | | + | | + | | + | | + | | + | + | **Accessory gene regulator B** |
| **SSGZ1_0130** | | | | | + | | + | | + | | + | | | + | | + | | + | | + | | + | | | + | | + | | + | | + | | + | | | + | | + | | + | | + | | + | | + | | | + | | + | | + | | + | | + | | | + | | + | | + | | + | | + | + | **putative folylpolyglutamate synthetase** |
| **SSGZ1_0131** | | | | | + | | + | | + | | + | | | + | | + | | + | | + | | + | | | + | | + | | + | | + | | + | | | + | | + | | + | | + | | + | | + | | | + | | + | | + | | + | | + | | | + | | + | | + | | + | | + | + | **glutamyl aminopeptidase PepA** |
| **SSGZ1_0132** | | | | | + | | + | | + | | + | | | + | | + | | + | | + | | + | | | + | | + | | + | | + | | + | | | + | | + | | + | | + | | + | | + | | | + | | + | | + | | + | | + | | | + | | + | | + | | + | | + | + | **conserved hypothetical protein** |
| **SSGZ1_0133** | | | | | + | | + | | + | | + | | | + | | + | | + | | + | | + | | | + | | + | | + | | + | | + | | | + | | + | | + | | + | | + | | + | | | + | | + | | + | | + | | + | | | + | | + | | + | | + | | + | + | **Thioredoxin-related protein** |
| **SSGZ1_0134** | | | | | + | | + | | + | | + | | | + | | + | | + | | + | | + | | | + | | + | | + | | + | | + | | | + | | + | | + | | + | | + | | + | | | + | | + | | + | | + | | + | | | + | | + | | + | | + | | + | + | **inosine-5-phosphate dehydrogenase** |
| **SSGZ1_0135** | | | | | + | | + | | + | | + | | | + | | + | | + | | + | | + | | | + | | + | | + | | + | | + | | | + | | + | | + | | + | | + | | + | | | + | | + | | + | | + | | + | | | + | | + | | + | | + | | + | + | **phenylalanyl-tRNA synthetase homolog** |
| **SSGZ1_0136** | | | | | + | | + | | + | | + | | | + | | + | | + | | + | | + | | | + | | + | | + | | + | | + | | | + | | + | | + | | + | | + | | + | | | + | | + | | + | | + | | + | | | + | | + | | + | | + | | + | + | **conserved hypothetical protein** |
| **SSGZ1_0137** | | | | | + | | + | | + | | + | | | + | | + | | + | | + | | + | | | + | | + | | + | | + | | + | | | + | | + | | + | | + | | + | | + | | | + | | + | | + | | + | | + | | | + | | + | | + | | + | | + | + | **Single-stranded DNA-binding protein** |
| **SSGZ1_0138** | | | | | + | | + | | + | | + | | | + | | + | | + | | + | | + | | | + | | + | | + | | + | | + | | | + | | + | | + | | + | | + | | + | | | + | | + | | + | | + | | + | | | + | | + | | + | | + | | + | + | **hypothetical protein** |
| **SSGZ1_0139** | | | | | + | | + | | + | | + | | | + | | + | | + | | + | | + | | | + | | + | | + | | + | | + | | | + | | + | | + | | + | | + | | + | | | + | | + | | + | | + | | + | | | + | | + | | + | | + | | + | + | **Chaperonin Cpn10** |
| **SSGZ1_0140** | | | | | + | | + | | + | | + | | | + | | + | | + | | + | | + | | | + | | + | | + | | + | | + | | | + | | + | | + | | + | | + | | + | | | + | | + | | + | | + | | + | | | + | | + | | + | | + | | + | + | **GroEL** |
| **SSGZ1_0141** | | | | | + | | + | | + | | + | | | + | | + | | + | | + | | + | | | + | | + | | + | | + | | + | | | + | | + | | + | | + | | + | | + | | | + | | + | | + | | + | | + | | | + | | + | | + | | + | | + | + | **ribosomal protein S12** |
| **SSGZ1_0142** | | | | | + | | + | | + | | + | | | + | | + | | + | | + | | + | | | + | | + | | + | | + | | + | | | + | | + | | + | | + | | + | | + | | | + | | + | | + | | + | | + | | | + | | + | | + | | + | | + | + | **ribosomal protein S7** |
| **SSGZ1_0143** | | | | | + | | + | | + | | + | | | + | | + | | + | | + | | + | | | + | | + | | + | | + | | + | | | + | | + | | + | | + | | + | | + | | | + | | + | | + | | + | | + | | | + | | + | | + | | + | | + | + | **translation elongation factor G:Small** |
| **SSGZ1_0144** | | | | | + | | + | | + | | + | | | + | | + | | + | | + | | + | | | + | | + | | + | | + | | - | | | + | | + | | - | | + | | + | | - | | | + | | + | | - | | + | | + | | | + | | + | | + | | - | | - | + | **Endothelin-converting enzyme 1** |
| **SSGZ1_0145** | | | | | + | | + | | + | | + | | | + | | + | | + | | + | | + | | | + | | + | | + | | + | | + | | | + | | + | | + | | + | | + | | + | | | + | | + | | + | | + | | + | | | + | | + | | + | | + | | + | + | **glyceraldehyde-3-phosphate dehydrogenase** |
| **SSGZ1_0146** | | | | | + | | + | | + | | + | | | + | | + | | + | | + | | + | | | + | | + | | + | | + | | + | | | + | | + | | + | | + | | + | | + | | | + | | + | | + | | + | | + | | | + | | + | | + | | + | | + | + | **phosphoglycerate kinase** |
| **SSGZ1_0147** | | | | | - | | + | | + | | + | | | + | | + | | + | | + | | + | | | + | | - | | + | | + | | + | | | + | | + | | + | | + | | + | | + | | | + | | + | | + | | + | | + | | | + | | + | | + | | + | | + | + | **Hypothetical protein** |
| **SSGZ1_0148** | | | | | + | | + | | + | | + | | | + | | + | | + | | + | | + | | | + | | + | | + | | + | | + | | | + | | + | | + | | + | | + | | + | | | + | | + | | + | | + | | + | | | + | | + | | + | | + | | + | + | **transcriptional regulator, glutamine synthetase** |
| **SSGZ1_0149** | | | | | + | | + | | + | | + | | | + | | + | | + | | + | | + | | | + | | + | | + | | + | | + | | | + | | + | | + | | + | | + | | + | | | + | | + | | + | | + | | + | | | + | | + | | + | | + | | + | + | **glutamine synthetase type 1** |
| **SSGZ1_0150** | | | | | + | | + | | + | | + | | | + | | + | | + | | + | | + | | | + | | + | | + | | + | | + | | | + | | + | | + | | + | | + | | + | | | + | | + | | + | | + | | + | | | + | | + | | + | | + | | + | + | **metallo-beta-lactamase superfamily protein** |
| **SSGZ1_0151** | | | | | + | | + | | + | | + | | | + | | + | | + | | + | | + | | | + | | + | | + | | + | | + | | | + | | + | | + | | + | | + | | + | | | + | | + | | + | | + | | + | | | + | | + | | + | | + | | + | + | **Uncharacterized conserved small protein** |
| **SSGZ1_0152** | | | | | + | | + | | + | | + | | | + | | + | | + | | + | | + | | | + | | + | | + | | + | | + | | | + | | + | | + | | + | | + | | + | | | + | | + | | + | | + | | + | | | + | | + | | + | | + | | + | + | **Peptidase M22, glycoprotease** |
| **SSGZ1_0153** | | | | | + | | + | | + | | + | | | + | | + | | + | | + | | + | | | + | | + | | + | | + | | + | | | + | | + | | + | | + | | + | | + | | | + | | + | | + | | + | | + | | | + | | + | | + | | + | | + | + | **Ribosomal-protein-alanine acetyltransferase** |
| **SSGZ1_0154** | | | | | + | | + | | + | | + | | | + | | + | | + | | + | | + | | | + | | + | | + | | + | | + | | | + | | + | | + | | + | | + | | + | | | + | | + | | + | | + | | + | | | + | | + | | + | | + | | + | + | **glycoprotease family protein** |
| **SSGZ1_0155** | | | | | + | | + | | + | | + | | | + | | + | | + | | + | | + | | | + | | + | | + | | + | | + | | | + | | + | | + | | + | | + | | + | | | + | | + | | + | | + | | + | | | + | | + | | + | | + | | + | + | **transcriptional regulator, AraC family** |
| **SSGZ1_0156** | | | | | + | | + | | + | | + | | | + | | + | | + | | + | | + | | | + | | + | | + | | + | | + | | | + | | + | | + | | + | | + | | - | | | + | | + | | + | | + | | + | | | + | | + | | + | | + | | + | + | **putative sugar ABC transporter, sugar-binding** |
| **SSGZ1_0157** | | | | | + | | + | | + | | + | | | + | | + | | + | | + | | + | | | + | | + | | + | | + | | + | | | + | | + | | + | | + | | + | | - | | | + | | + | | + | | + | | + | | | + | | - | | + | | + | | + | + | **Binding-protein-dependent transport systems** |
| **SSGZ1_0158** | | | | | + | | + | | + | | + | | | + | | + | | + | | + | | + | | | + | | + | | + | | + | | + | | | + | | + | | + | | + | | + | | - | | | + | | + | | + | | + | | + | | | + | | - | | + | | + | | + | + | **Binding-protein-dependent transport systems** |
| **SSGZ1_0159** | | | | | + | | + | | + | | + | | | + | | + | | + | | + | | + | | | + | | + | | + | | + | | + | | | + | | + | | + | | + | | + | | - | | | + | | + | | + | | + | | + | | | + | | - | | + | | + | | + | + | **Glycoside hydrolase, clan GH-D** |
| **SSGZ1_0160** | | | | | + | | + | | + | | + | | | + | | + | | + | | + | | + | | | + | | + | | + | | + | | + | | | + | | + | | + | | + | | + | | + | | | + | | + | | + | | + | | + | | | + | | + | | + | | + | | + | + | **putative integral membrane protein, possible** |
| **SSGZ1_0161** | | | | | + | | + | | + | | + | | | + | | + | | + | | + | | + | | | + | | + | | + | | + | | + | | | + | | + | | + | | + | | + | | + | | | + | | + | | + | | + | | + | | | + | | + | | + | | + | | + | + | **Branched-chain amino acid transport** |
| **SSGZ1_0162** | | | | | + | | + | | + | | + | | | + | | + | | + | | + | | + | | | + | | + | | + | | + | | + | | | + | | + | | + | | + | | + | | + | | | + | | + | | + | | + | | + | | | + | | + | | + | | + | | + | + | **Predicted membrane protein** |
| **SSGZ1_0163** | | | | | + | | + | | + | | + | | | + | | + | | + | | + | | + | | | + | | + | | + | | + | | + | | | + | | + | | + | | + | | + | | + | | | + | | + | | + | | + | | + | | | + | | + | | + | | + | | + | + | **hypothetical protein** |
| **SSGZ1_0164** | | | | | - | | + | | - | | + | | | + | | + | | + | | + | | + | | | - | | - | | - | | - | | - | | | - | | - | | - | | - | | - | | - | | | + | | - | | - | | - | | - | | | + | | + | | - | | - | | - | - | **EF** |
| **SSGZ1_0165** | | | | | - | | + | | + | | + | | | + | | + | | + | | + | | + | | | + | | - | | + | | + | | + | | | - | | - | | - | | - | | - | | - | | | + | | + | | - | | + | | + | | | + | | + | | + | | - | | - | - | **large variant extracellular factor,C-terminal** |
| **SSGZ1_0166** | | | | | - | | + | | + | | + | | | + | | + | | + | | + | | + | | | + | | - | | + | | + | | + | | | - | | - | | - | | - | | - | | - | | | + | | + | | - | | + | | + | | | + | | + | | + | | - | | - | - | **Putative RTX family exoprotein A gene** |
| **SSGZ1_0167** | | | | | - | | + | | + | | + | | | + | | + | | + | | + | | + | | | + | | - | | + | | + | | + | | | + | | + | | + | | + | | + | | + | | | + | | + | | + | | + | | + | | | + | | + | | + | | + | | + | + | **PIG-X/PBN1** |
| **SSGZ1_0168** | | | | | + | | + | | + | | + | | | + | | + | | + | | + | | + | | | + | | + | | + | | + | | + | | | + | | + | | + | | + | | + | | + | | | + | | + | | + | | + | | + | | | + | | + | | + | | + | | + | + | **ABC transporter** |
| **SSGZ1_0169** | | | | | + | | + | | + | | + | | | + | | + | | + | | + | | + | | | + | | + | | + | | + | | + | | | + | | + | | + | | + | | + | | + | | | + | | + | | + | | + | | + | | | + | | + | | + | | + | | + | + | **Predicted flavoprotein** |
| **SSGZ1_0170** | | | | | + | | + | | + | | + | | | + | | + | | + | | + | | + | | | + | | + | | + | | + | | + | | | + | | + | | + | | + | | + | | + | | | + | | + | | + | | + | | + | | | + | | + | | + | | + | | + | + | **Beta-glucosidase** |
| **SSGZ1_0171** | | | | | + | | + | | + | | + | | | + | | + | | + | | + | | + | | | + | | + | | + | | + | | + | | | + | | + | | + | | + | | + | | + | | | + | | + | | + | | + | | + | | | + | | + | | + | | + | | + | + | **conserved hypothetical protein** |
| **SSGZ1_0172** | | | | | + | | + | | + | | + | | | + | | + | | + | | + | | + | | | + | | + | | + | | + | | - | | | + | | - | | - | | - | | - | | + | | | + | | + | | + | | + | | + | | | + | | + | | + | | + | | - | - | **Transcriptional antiterminator** |
| **SSGZ1_0173** | | | | | + | | + | | + | | + | | | + | | + | | + | | + | | + | | | + | | + | | + | | + | | - | | | + | | - | | - | | - | | - | | + | | | + | | + | | + | | + | | + | | | + | | + | | + | | + | | - | - | **Phosphotransferase system,** |
| **SSGZ1_0174** | | | | | + | | + | | + | | + | | | + | | + | | + | | + | | + | | | + | | + | | + | | + | | - | | | + | | - | | - | | - | | - | | + | | | + | | + | | + | | + | | + | | | + | | + | | + | | + | | - | - | **Putative sugar-specific permease, SgaT/UlaA** |
| **SSGZ1_0175** | | | | | + | | + | | + | | + | | | + | | + | | + | | + | | + | | | + | | + | | + | | + | | - | | | + | | - | | - | | - | | - | | + | | | + | | + | | + | | + | | + | | | + | | + | | + | | + | | - | - | **Transketolase, N terminal** |
| **SSGZ1_0176** | | | | | + | | + | | + | | + | | | + | | + | | + | | + | | + | | | + | | + | | + | | + | | - | | | + | | - | | - | | - | | - | | + | | | + | | + | | + | | + | | + | | | + | | + | | + | | + | | - | - | **Transketolase, C terminal** |
| **SSGZ1_0177** | | | | | - | | - | | + | | - | | | - | | - | | - | | - | | - | | | + | | + | | - | | - | | + | | | - | | + | | - | | + | | - | | + | | | - | | - | | + | | + | | - | | | + | | + | | + | | + | | + | + | **GCN5-related N-acetyltransferase** |
| **SSGZ1_0178** | | | | | + | | + | | + | | + | | | + | | + | | + | | + | | + | | | + | | + | | + | | + | | + | | | + | | + | | + | | + | | + | | + | | | + | | + | | + | | + | | + | | | + | | + | | + | | + | | + | + | **Glycerophosphoryl diester phosphodiesterase** |
| **SSGZ1_0179** | | | | | + | | + | | + | | + | | | + | | + | | + | | + | | + | | | + | | + | | + | | + | | + | | | + | | + | | + | | + | | + | | + | | | + | | + | | + | | + | | + | | | + | | + | | + | | + | | + | + | **Glycerophosphoryl diester phosphodiesterase** |
| **SSGZ1_0180** | | | | | + | | + | | + | | + | | | + | | + | | + | | + | | + | | | + | | + | | + | | + | | + | | | + | | + | | + | | + | | + | | + | | | + | | + | | + | | + | | + | | | + | | + | | + | | + | | + | + | **Conserved hypothetical protein** |
| **SSGZ1_0181** | | | | | + | | + | | + | | + | | | + | | + | | + | | + | | + | | | + | | + | | + | | + | | + | | | + | | + | | + | | + | | + | | + | | | + | | + | | + | | + | | + | | | + | | + | | + | | + | | + | + | **Sugar isomerase (SIS)** |
| **SSGZ1_0182** | | | | | - | | - | | + | | - | | | - | | - | | - | | - | | - | | | + | | - | | - | | - | | + | | | + | | + | | + | | + | | - | | - | | | - | | - | | + | | - | | - | | | - | | + | | + | | + | | + | + | **Surface protein from Gram-positive cocci, anchor** |
| **SSGZ1_0183** | | | | | + | | + | | + | | + | | | + | | + | | + | | + | | + | | | + | | - | | + | | + | | - | | | + | | + | | + | | - | | - | | + | | | - | | + | | - | | + | | + | | | + | | + | | + | | - | | + | + | **Transposase,IS4** |
| **SSGZ1_0184** | | | | | + | | + | | + | | + | | | + | | + | | + | | + | | + | | | + | | + | | + | | + | | + | | | + | | + | | + | | + | | + | | + | | | - | | + | | + | | + | | + | | | + | | + | | + | | + | | + | + | **dipeptidyl aminopeptidase IV** |
| **SSGZ1_0185** | | | | | + | | + | | + | | + | | | + | | + | | + | | + | | + | | | + | | + | | + | | + | | + | | | + | | + | | + | | + | | + | | + | | | + | | + | | + | | + | | + | | | + | | + | | + | | + | | + | + | **Methyl-accepting chemotaxis protein** |
| **SSGZ1_0186** | | | | | + | | - | | + | | - | | | + | | + | | + | | - | | - | | | + | | + | | + | | + | | + | | | + | | + | | + | | + | | + | | + | | | + | | + | | + | | + | | + | | | + | | + | | + | | + | | + | + | **Beta-lactamase** |
| **SSGZ1_0187** | | | | | + | | + | | + | | + | | | + | | + | | + | | + | | + | | | + | | + | | + | | + | | + | | | + | | + | | + | | + | | + | | + | | | + | | + | | + | | + | | + | | | + | | + | | + | | + | | + | + | **'Formate acetyltransferase** |
| **SSGZ1_0188** | | | | | + | | + | | + | | + | | | + | | + | | + | | + | | + | | | + | | + | | + | | + | | + | | | + | | + | | + | | + | | + | | + | | | + | | + | | + | | + | | + | | | + | | + | | + | | + | | + | - | **DNA-directed DNA polymerase** |
| **SSGZ1_0189** | | | | | + | | + | | + | | + | | | + | | + | | + | | + | | + | | | + | | + | | + | | + | | + | | | + | | + | | + | | + | | + | | + | | | + | | + | | + | | + | | + | | | + | | + | | + | | + | | + | + | **Transcriptional regulator, Rrf2** |
| **SSGZ1_0190** | | | | | + | | + | | + | | + | | | + | | + | | + | | + | | + | | | + | | + | | + | | + | | + | | | + | | + | | + | | + | | + | | + | | | + | | + | | + | | + | | + | | | + | | + | | + | | + | | + | + | **NAD(P)-binding Rossmann-like Domain** |
| **SSGZ1_0191** | | | | | + | | + | | + | | + | | | + | | + | | + | | + | | + | | | + | | + | | + | | + | | + | | | + | | + | | + | | + | | + | | + | | | + | | + | | + | | + | | + | | | + | | + | | + | | + | | + | + | **similar to Fructose-1-phosphate kinase and** |
| **SSGZ1_0192** | | | | | + | | + | | + | | + | | | + | | + | | + | | + | | + | | | + | | + | | + | | + | | - | | | + | | + | | - | | + | | + | | + | | | + | | - | | + | | + | | + | | | + | | + | | + | | - | | + | - | **Helix-hairpin-helix motif:HhH-GPD** |
| **SSGZ1_0193** | | | | | + | | + | | - | | + | | | + | | + | | + | | + | | + | | | - | | + | | + | | + | | - | | | + | | + | | - | | - | | - | | + | | | + | | - | | + | | + | | + | | | + | | + | | + | | - | | - | - | **ROK** |
| **SSGZ1_0194** | | | | | + | | + | | - | | + | | | + | | + | | + | | + | | + | | | - | | + | | + | | + | | - | | | + | | - | | - | | - | | - | | - | | | + | | - | | - | | + | | + | | | + | | - | | + | | - | | - | - | **PTS lactose/cellobiose IIC component** |
| **SSGZ1_0195** | | | | | + | | + | | - | | + | | | + | | + | | + | | + | | + | | | - | | + | | + | | + | | - | | | + | | + | | - | | - | | - | | + | | | + | | - | | - | | + | | + | | | + | | + | | + | | - | | - | - | **conserved hypothetical protein** |
| **SSGZ1_0196** | | | | | - | | + | | - | | + | | | + | | + | | + | | + | | + | | | - | | - | | + | | + | | - | | | + | | - | | - | | - | | - | | - | | | + | | - | | - | | - | | - | | | + | | + | | - | | - | | - | - | **Parallel beta-helix repeat** |
| **SSGZ1_0197** | | | | | + | | + | | - | | + | | | + | | + | | + | | + | | + | | | - | | + | | + | | + | | - | | | + | | + | | - | | - | | - | | + | | | + | | - | | + | | + | | + | | | + | | + | | + | | + | | + | - | **hypothetical protein** |
| **SSGZ1_0198** | | | | | + | | + | | - | | + | | | + | | + | | + | | + | | + | | | - | | + | | + | | + | | - | | | + | | - | | - | | - | | - | | - | | | + | | - | | - | | + | | + | | | + | | + | | + | | - | | - | - | **Binding-protein-dependent transport systems** |
| **SSGZ1_0199** | | | | | + | | + | | - | | + | | | + | | + | | + | | + | | + | | | - | | + | | + | | + | | - | | | + | | + | | - | | - | | - | | + | | | + | | - | | - | | + | | + | | | + | | + | | + | | - | | - | - | **taurine ABC transporter, ATP-binding protein** |
| **SSGZ1_0200** | | | | | + | | + | | - | | + | | | + | | + | | + | | + | | + | | | - | | + | | + | | + | | - | | | + | | - | | - | | - | | - | | - | | | + | | - | | - | | + | | + | | | + | | - | | + | | - | | - | - | **NLPA lipoprotein** |
| **SSGZ1_0201** | | | | | + | | + | | - | | + | | | + | | + | | + | | + | | + | | | - | | + | | + | | + | | - | | | + | | + | | - | | - | | - | | + | | | + | | - | | + | | + | | + | | | + | | + | | + | | + | | - | - | **Glucosamine/galactosamine-6-phosphate isomerase** |
| **SSGZ1_0202** | | | | | + | | + | | - | | + | | | + | | + | | + | | + | | + | | | - | | + | | + | | + | | - | | | + | | - | | - | | - | | - | | - | | | + | | - | | - | | + | | + | | | + | | - | | + | | - | | - | - | **cation-transporting ATPase** |
| **SSGZ1_0203** | | | | | + | | + | | - | | + | | | + | | + | | + | | + | | + | | | - | | + | | + | | + | | - | | | + | | - | | - | | - | | - | | - | | | + | | - | | - | | + | | + | | | + | | + | | + | | - | | - | - | **E1-E2 ATPase-associated region** |
| **SSGZ1_0204** | | | | | + | | + | | + | | + | | | + | | + | | + | | + | | + | | | + | | + | | + | | + | | + | | | + | | + | | + | | + | | + | | + | | | + | | - | | + | | + | | + | | | + | | + | | + | | + | | + | + | **Copper-exporting ATPase** |
| **SSGZ1_0205** | | | | | + | | + | | + | | + | | | + | | + | | + | | + | | + | | | + | | + | | + | | + | | + | | | + | | + | | + | | + | | + | | + | | | + | | + | | + | | + | | + | | | + | | + | | + | | + | | + | + | **Helicase RecD/TraA** |
| **SSGZ1_0206** | | | | | + | | + | | + | | + | | | + | | + | | + | | + | | + | | | + | | + | | + | | + | | + | | | + | | + | | + | | + | | + | | + | | | + | | + | | + | | + | | + | | | + | | + | | + | | + | | + | + | **Signal peptidase I** |
| **SSGZ1_0207** | | | | | + | | + | | + | | + | | | + | | + | | + | | + | | + | | | + | | + | | + | | + | | + | | | + | | + | | + | | + | | + | | + | | | + | | + | | + | | + | | + | | | + | | + | | + | | + | | + | + | **Ribonuclease HIII** |
| **SSGZ1_0208** | | | | | + | | + | | + | | + | | | + | | + | | + | | + | | + | | | + | | + | | + | | + | | + | | | + | | + | | + | | + | | + | | + | | | + | | + | | + | | + | | + | | | + | | + | | + | | + | | + | + | **IgG binding protein** |
| **SSGZ1_0209** | | | | | + | | + | | + | | + | | | + | | + | | + | | + | | + | | | + | | + | | + | | + | | + | | | + | | + | | + | | + | | + | | + | | | + | | + | | + | | + | | + | | | + | | + | | + | | + | | + | + | **Peptidoglycan-binding LysM** |
| **SSGZ1_0210** | | | | | + | | + | | + | | + | | | + | | + | | + | | + | | + | | | + | | + | | + | | + | | + | | | + | | + | | + | | + | | + | | + | | | + | | + | | + | | + | | + | | | + | | + | | + | | + | | + | + | **dextran glucosidase DexS** |
| **SSGZ1_0211** | | | | | + | | + | | + | | + | | | + | | + | | + | | + | | + | | | + | | + | | + | | + | | + | | | + | | + | | + | | + | | + | | + | | | + | | + | | + | | + | | + | | | + | | + | | + | | + | | + | + | **dextran glucosidase DexS** |
| **SSGZ1_0212** | | | | | + | | + | | + | | + | | | + | | + | | + | | + | | + | | | + | | + | | + | | + | | + | | | + | | + | | + | | + | | + | | + | | | + | | + | | + | | + | | + | | | + | | + | | + | | + | | + | + | **'PTS system, trehalose-specific IIBC component /** |
| **SSGZ1_0213** | | | | | + | | + | | + | | + | | | + | | + | | + | | + | | + | | | + | | + | | + | | + | | + | | | + | | + | | + | | + | | + | | + | | | + | | + | | + | | + | | + | | | + | | + | | + | | + | | + | + | **regulatory protein, GntR** |
| **SSGZ1_0214** | | | | | + | | + | | + | | + | | | + | | + | | + | | + | | + | | | + | | + | | + | | + | | + | | | + | | + | | + | | + | | + | | + | | | + | | + | | + | | + | | + | | | + | | + | | + | | + | | + | + | **Cell division protein ZapA-like** |
| **SSGZ1_0215** | | | | | + | | + | | + | | + | | | + | | + | | + | | + | | + | | | + | | + | | + | | + | | + | | | + | | + | | + | | + | | + | | + | | | + | | + | | + | | + | | + | | | + | | + | | + | | + | | + | + | **Colicin V production protein** |
| **SSGZ1_0216** | | | | | + | | + | | + | | + | | | + | | + | | + | | + | | + | | | + | | + | | + | | + | | + | | | + | | + | | + | | + | | + | | + | | | + | | + | | + | | + | | + | | | + | | + | | + | | + | | + | + | **DNA structure-specific ATPase involved in** |
| **SSGZ1_0217** | | | | | - | | + | | + | | + | | | + | | + | | + | | + | | + | | | + | | - | | + | | + | | + | | | - | | + | | + | | + | | - | | + | | | + | | + | | + | | + | | + | | | + | | + | | + | | + | | + | + | **acetyltransferase, GNAT family protein** |
| **SSGZ1_0218** | | | | | + | | + | | + | | + | | | + | | + | | + | | + | | + | | | + | | + | | + | | + | | + | | | + | | + | | + | | + | | + | | + | | | + | | + | | + | | + | | + | | | + | | + | | + | | + | | + | + | **Thioredoxin** |
| **SSGZ1_0219** | | | | | + | | + | | + | | + | | | + | | + | | + | | + | | + | | | + | | + | | + | | + | | + | | | + | | + | | + | | + | | + | | + | | | + | | + | | + | | + | | + | | | + | | + | | + | | + | | + | + | **AMP-dependent synthetase and ligase** |
| **SSGZ1_0220** | | | | | + | | + | | + | | + | | | + | | + | | + | | + | | + | | | + | | + | | + | | + | | + | | | + | | + | | + | | + | | + | | + | | | + | | + | | + | | + | | + | | | + | | + | | + | | + | | + | + | **Peptide methionine sulfoxide reductase** |
| **SSGZ1_0221** | | | | | - | | + | | + | | + | | | + | | + | | + | | + | | + | | | + | | - | | + | | + | | - | | | - | | + | | + | | - | | - | | + | | | + | | + | | + | | + | | + | | | + | | + | | + | | + | | - | - | **Transcriptional activator** |
| **SSGZ1_0222** | | | | | + | | + | | + | | + | | | + | | + | | + | | + | | + | | | + | | + | | + | | + | | - | | | - | | - | | + | | + | | + | | + | | | + | | + | | - | | + | | + | | | + | | + | | + | | + | | - | - | **Bacteriocin-associated integral membrane** |
| **SSGZ1_0223** | | | | | + | | + | | + | | + | | | + | | + | | + | | + | | + | | | + | | + | | + | | + | | + | | | + | | + | | + | | + | | + | | + | | | + | | + | | + | | + | | + | | | + | | + | | + | | + | | + | + | **Bacteriocin-associated integral membrane** |
| **SSGZ1_0224** | | | | | + | | + | | + | | + | | | + | | + | | + | | + | | + | | | + | | + | | + | | + | | + | | | + | | + | | + | | + | | + | | + | | | + | | + | | + | | + | | + | | | + | | + | | + | | + | | + | + | **ABC transporter , ATP-binding protein** |
| **SSGZ1_0225** | | | | | + | | + | | + | | + | | | + | | + | | + | | + | | + | | | + | | + | | + | | + | | + | | | + | | + | | + | | + | | + | | + | | | + | | + | | + | | + | | + | | | + | | + | | + | | + | | + | + | **Sodium:alanine symporter** |
| **SSGZ1_0226** | | | | | + | | + | | + | | + | | | + | | + | | + | | + | | + | | | + | | + | | + | | + | | + | | | + | | + | | + | | + | | + | | + | | | + | | + | | + | | + | | + | | | + | | + | | + | | + | | + | + | **MscS Mechanosensitive ion channel** |
| **SSGZ1_0227** | | | | | + | | + | | + | | + | | | + | | + | | + | | + | | + | | | + | | + | | + | | + | | + | | | + | | + | | + | | + | | + | | + | | | + | | + | | + | | + | | + | | | + | | + | | + | | + | | + | + | **Predicted ATPase involved in replication** |
| **SSGZ1_0228** | | | | | + | | + | | + | | + | | | + | | + | | + | | + | | + | | | + | | + | | + | | + | | + | | | + | | + | | + | | + | | + | | + | | | + | | + | | + | | + | | + | | | + | | + | | + | | + | | + | + | **NAD(P)H-dependent glutamate dehydrogenase** |
| **SSGZ1_0229** | | | | | + | | + | | + | | + | | | + | | + | | + | | + | | + | | | + | | + | | + | | + | | + | | | + | | + | | + | | + | | + | | + | | | + | | + | | + | | + | | + | | | + | | + | | + | | + | | + | + | **dihydroorotate dehydrogenase** |
| **SSGZ1_0230** | | | | | + | | + | | + | | + | | | + | | + | | + | | + | | + | | | + | | + | | + | | + | | + | | | + | | + | | + | | + | | + | | + | | | + | | + | | + | | + | | + | | | + | | + | | + | | + | | + | + | **hypothetical protein** |
| **SSGZ1_0231** | | | | | + | | + | | + | | + | | | + | | + | | + | | + | | + | | | + | | + | | + | | + | | + | | | + | | + | | + | | + | | + | | + | | | + | | + | | + | | + | | - | | | - | | + | | - | | + | | + | + | **Predicted hydrolase of the HAD superfamily** |
| **SSGZ1_0232** | | | | | + | | + | | + | | + | | | + | | + | | + | | + | | + | | | + | | + | | + | | + | | + | | | + | | + | | + | | + | | + | | + | | | + | | + | | + | | + | | + | | | + | | + | | + | | + | | + | + | **ABC-type uncharacterized transport system,** |
| **SSGZ1_0233** | | | | | + | | + | | + | | + | | | + | | + | | + | | + | | + | | | + | | + | | + | | + | | + | | | + | | + | | + | | + | | + | | + | | | + | | + | | + | | + | | + | | | + | | + | | + | | + | | + | + | **ABC-type uncharacterized transport system,** |
| **SSGZ1_0234** | | | | | + | | + | | + | | + | | | + | | + | | + | | + | | + | | | + | | + | | + | | + | | + | | | + | | + | | + | | + | | + | | + | | | + | | + | | + | | + | | + | | | + | | + | | + | | + | | + | + | **ABC transporter,ATP-binding protein** |
| **SSGZ1_0235** | | | | | + | | + | | + | | + | | | + | | + | | + | | + | | + | | | + | | + | | + | | + | | + | | | + | | + | | + | | + | | + | | + | | | + | | + | | + | | + | | + | | | + | | + | | + | | + | | + | + | **regulatory protein, TetR** |
| **SSGZ1_0236** | | | | | + | | + | | + | | + | | | + | | + | | + | | + | | + | | | + | | + | | + | | + | | + | | | + | | + | | + | | + | | + | | + | | | + | | + | | + | | + | | + | | | + | | + | | + | | + | | + | + | **putative membrane protein** |
| **SSGZ1_0237** | | | | | + | | + | | + | | + | | | + | | + | | + | | + | | + | | | + | | + | | + | | + | | + | | | + | | + | | + | | + | | + | | + | | | + | | - | | + | | + | | - | | | - | | + | | - | | + | | + | - | **hypothetical protein** |
| **SSGZ1_0238** | | | | | + | | + | | + | | + | | | + | | + | | + | | + | | + | | | + | | + | | + | | + | | + | | | + | | + | | + | | + | | + | | + | | | + | | + | | + | | + | | + | | | + | | + | | + | | + | | + | + | **hypothetical protein** |
| **SSGZ1_0239** | | | | | + | | + | | + | | + | | | + | | + | | + | | + | | + | | | + | | + | | + | | + | | + | | | + | | + | | + | | + | | + | | + | | | + | | + | | + | | + | | + | | | + | | + | | + | | + | | + | + | **Predicted membrane protein** |
| **SSGZ1_0240** | | | | | + | | + | | + | | + | | | + | | + | | + | | + | | + | | | + | | + | | + | | + | | + | | | + | | + | | + | | + | | + | | + | | | + | | + | | + | | + | | + | | | + | | + | | + | | + | | + | + | **Predicted transcriptional regulator** |
| **SSGZ1_0241** | | | | | + | | + | | + | | + | | | + | | + | | + | | + | | + | | | + | | + | | + | | + | | + | | | + | | + | | - | | + | | + | | + | | | + | | + | | - | | + | | + | | | + | | + | | + | | - | | + | + | **LrgA** |
| **SSGZ1_0242** | | | | | + | | + | | + | | + | | | + | | + | | + | | + | | + | | | + | | + | | + | | + | | + | | | + | | + | | - | | + | | + | | + | | | + | | + | | - | | + | | + | | | + | | + | | + | | - | | + | + | **LrgB** |
| **SSGZ1_0243** | | | | | + | | + | | + | | + | | | + | | + | | + | | + | | + | | | + | | + | | + | | + | | + | | | + | | + | | - | | + | | + | | + | | | + | | + | | - | | + | | + | | | + | | + | | + | | - | | + | + | **Formate/nitrite transporter** |
| **SSGZ1_0244** | | | | | + | | + | | + | | + | | | + | | + | | + | | + | | + | | | + | | + | | + | | + | | + | | | + | | + | | + | | + | | + | | + | | | + | | + | | + | | + | | + | | | + | | + | | + | | + | | + | + | **glycerol uptake facilitator protein, putative** |
| **SSGZ1_0245** | | | | | + | | + | | + | | + | | | + | | + | | + | | + | | + | | | + | | + | | + | | + | | + | | | + | | + | | + | | + | | + | | + | | | + | | + | | + | | + | | - | | | + | | + | | + | | + | | + | + | **ATPase** |
| **SSGZ1_0246** | | | | | + | | + | | + | | + | | | + | | + | | + | | + | | + | | | + | | + | | + | | + | | + | | | + | | + | | + | | + | | + | | + | | | + | | + | | + | | + | | + | | | + | | + | | + | | + | | + | + | **NUDIX hydrolase** |
| **SSGZ1_0247** | | | | | + | | + | | + | | + | | | + | | + | | + | | + | | + | | | + | | + | | + | | + | | + | | | + | | + | | + | | + | | + | | + | | | + | | + | | + | | + | | + | | | + | | + | | + | | + | | + | + | **hypothetical protein** |
| **SSGZ1_0248** | | | | | + | | + | | + | | + | | | + | | + | | + | | + | | + | | | + | | + | | + | | + | | + | | | + | | + | | + | | + | | + | | - | | | + | | - | | + | | + | | - | | | + | | - | | + | | + | | + | + | **Translation initiation factor 2 (IF-2; GTPase)** |
| **SSGZ1_0249** | | | | | + | | + | | + | | + | | | + | | + | | + | | + | | + | | | + | | + | | + | | + | | + | | | + | | - | | + | | + | | + | | - | | | + | | + | | + | | + | | - | | | + | | + | | + | | + | | + | + | **Methyl-accepting chemotaxis protein** |
| **SSGZ1_0250** | | | | | + | | + | | + | | + | | | + | | + | | + | | + | | + | | | + | | + | | + | | + | | + | | | + | | - | | + | | + | | + | | - | | | + | | + | | + | | + | | - | | | + | | - | | + | | + | | + | + | **Methyl-accepting chemotaxis protein** |
| **SSGZ1_0251** | | | | | + | | + | | + | | + | | | + | | + | | + | | + | | + | | | + | | + | | + | | + | | + | | | + | | + | | + | | + | | + | | + | | | + | | + | | + | | + | | + | | | + | | + | | + | | + | | + | + | **hypothetical protein** |
| **SSGZ1_0252** | | | | | + | | + | | + | | + | | | + | | + | | + | | + | | + | | | + | | + | | + | | + | | + | | | + | | + | | + | | + | | + | | + | | | + | | + | | + | | + | | + | | | + | | + | | + | | + | | + | + | **50S ribosomal protein L33** |
| **SSGZ1_0253** | | | | | + | | + | | + | | + | | | + | | + | | + | | + | | + | | | + | | + | | + | | + | | + | | | + | | + | | + | | + | | + | | + | | | + | | + | | + | | + | | + | | | + | | + | | + | | + | | + | + | **Ribosomal protein L32** |
| **SSGZ1_0254** | | | | | + | | + | | + | | + | | | + | | + | | + | | + | | + | | | + | | + | | + | | + | | + | | | + | | + | | + | | + | | + | | + | | | + | | + | | + | | + | | + | | | + | | + | | + | | + | | + | + | **Histidyl-tRNA synthetase, class IIa** |
| **SSGZ1_0255** | | | | | + | | + | | + | | + | | | + | | + | | + | | + | | + | | | + | | + | | + | | + | | + | | | + | | + | | + | | + | | + | | + | | | + | | + | | + | | + | | + | | | + | | + | | + | | + | | + | + | **Zinc-containing alcohol dehydrogenase** |
| **SSGZ1_0256** | | | | | + | | + | | + | | + | | | + | | + | | + | | + | | + | | | + | | + | | + | | + | | + | | | + | | + | | + | | + | | + | | + | | | + | | + | | + | | + | | + | | | + | | + | | + | | + | | + | + | **hypothetical protein** |
| **SSGZ1_0257** | | | | | + | | + | | + | | + | | | + | | + | | + | | + | | + | | | + | | + | | + | | + | | + | | | + | | + | | + | | + | | + | | + | | | + | | + | | + | | + | | + | | | + | | + | | + | | + | | + | + | **Iron-containing alcohol dehydrogenase** |
| **SSGZ1_0258** | | | | | + | | + | | + | | + | | | + | | + | | + | | + | | + | | | + | | + | | + | | + | | + | | | + | | + | | + | | + | | + | | + | | | + | | + | | + | | + | | + | | | + | | + | | + | | + | | + | + | **Threonine synthase** |
| **SSGZ1_0259** | | | | | + | | + | | + | | + | | | + | | + | | + | | + | | + | | | + | | + | | + | | + | | + | | | + | | + | | + | | + | | + | | + | | | + | | + | | + | | + | | + | | | + | | + | | + | | + | | + | + | **ABC transporter, ATP-binding protein** |
| **SSGZ1_0260** | | | | | + | | + | | + | | + | | | + | | + | | + | | + | | + | | | + | | + | | + | | + | | + | | | + | | + | | + | | + | | + | | + | | | + | | + | | + | | + | | + | | | + | | + | | + | | + | | + | + | **ABC transporter, transmembrane region** |
| **SSGZ1_0261** | | | | | + | | + | | + | | + | | | + | | + | | + | | + | | + | | | + | | + | | + | | + | | + | | | + | | + | | + | | + | | + | | + | | | + | | + | | + | | + | | + | | | + | | + | | + | | + | | + | + | **hypothetical protein** |
| **SSGZ1_0262** | | | | | + | | + | | + | | + | | | + | | + | | + | | + | | + | | | + | | + | | + | | + | | + | | | + | | + | | + | | + | | + | | + | | | + | | + | | + | | + | | + | | | + | | + | | + | | + | | + | + | **ABC-type cobalt transport system, permease** |
| **SSGZ1_0263** | | | | | + | | + | | + | | + | | | + | | + | | + | | + | | + | | | + | | + | | + | | + | | + | | | + | | + | | + | | + | | + | | + | | | + | | + | | + | | + | | + | | | + | | + | | + | | + | | + | + | **ABC transporter component protein** |
| **SSGZ1_0264** | | | | | + | | + | | + | | + | | | + | | + | | + | | + | | + | | | + | | + | | + | | + | | + | | | + | | + | | + | | + | | + | | + | | | + | | + | | + | | + | | + | | | + | | + | | + | | + | | + | + | **Multi antimicrobial extrusion protein MatE** |
| **SSGZ1_0265** | | | | | + | | + | | + | | + | | | + | | + | | + | | + | | + | | | + | | + | | + | | + | | + | | | + | | + | | + | | + | | + | | + | | | + | | + | | + | | + | | + | | | + | | + | | + | | + | | + | + | **Mevalonate kinase** |
| **SSGZ1_0266** | | | | | + | | + | | + | | + | | | + | | + | | + | | + | | + | | | + | | + | | + | | + | | + | | | + | | + | | + | | + | | + | | + | | | + | | + | | + | | + | | + | | | + | | + | | + | | + | | + | + | **Diphosphomevalonate decarboxylase** |
| **SSGZ1_0267** | | | | | + | | + | | + | | + | | | + | | + | | + | | + | | + | | | + | | + | | + | | + | | + | | | + | | + | | + | | + | | + | | + | | | + | | + | | + | | + | | + | | | + | | + | | + | | + | | + | + | **Gram positive phosphomevalonate kinase** |
| **SSGZ1_0268** | | | | | + | | + | | + | | + | | | + | | + | | + | | + | | + | | | + | | + | | + | | + | | + | | | + | | + | | + | | + | | + | | + | | | + | | + | | + | | + | | + | | | + | | + | | + | | + | | + | + | **Isopentenyl-diphosphate delta-isomerase** |
| **SSGZ1_0269** | | | | | + | | + | | + | | + | | | + | | + | | + | | + | | + | | | + | | + | | + | | + | | + | | | + | | - | | + | | + | | + | | + | | | + | | + | | + | | + | | + | | | + | | + | | + | | + | | + | + | **ABC transporter,transmembrane region** |
| **SSGZ1_0270** | | | | | + | | + | | + | | + | | | + | | + | | + | | + | | + | | | + | | + | | + | | + | | + | | | + | | - | | + | | + | | + | | + | | | + | | + | | + | | + | | + | | | + | | + | | + | | + | | + | + | **ABC transporter, permease protein** |
| **SSGZ1_0271** | | | | | + | | + | | + | | + | | | + | | + | | + | | + | | + | | | + | | + | | + | | + | | + | | | + | | + | | + | | + | | + | | + | | | + | | + | | + | | + | | + | | | + | | + | | + | | + | | + | + | **ACT domain-containing protein** |
| **SSGZ1_0272** | | | | | + | | + | | + | | + | | | + | | + | | + | | + | | + | | | + | | + | | + | | + | | + | | | + | | + | | + | | + | | + | | + | | | + | | + | | + | | + | | + | | | + | | + | | + | | + | | + | + | **hypothetical protein** |
| **SSGZ1_0273** | | | | | + | | + | | + | | + | | | + | | + | | + | | + | | + | | | - | | + | | + | | + | | + | | | + | | + | | + | | + | | + | | + | | | + | | + | | + | | + | | + | | | + | | + | | + | | + | | + | + | **putative phosphoglycerate mutase** |
| **SSGZ1_0274** | | | | | + | | + | | + | | + | | | + | | + | | + | | + | | + | | | + | | + | | + | | + | | + | | | + | | + | | + | | + | | + | | + | | | + | | + | | + | | + | | + | | | + | | + | | + | | + | | + | + | **heat shock protein regulator** |
| **SSGZ1_0275** | | | | | + | | + | | + | | + | | | + | | + | | + | | + | | + | | | + | | + | | + | | + | | + | | | + | | + | | + | | + | | + | | + | | | + | | + | | + | | + | | + | | | + | | + | | + | | + | | + | + | **GrpE protein** |
| **SSGZ1_0276** | | | | | + | | + | | + | | + | | | + | | + | | + | | + | | + | | | + | | + | | + | | + | | + | | | + | | + | | + | | + | | + | | + | | | + | | + | | + | | + | | + | | | + | | + | | + | | + | | + | + | **Heat shock protein Hsp70** |
| **SSGZ1_0277** | | | | | + | | + | | + | | + | | | + | | + | | + | | + | | + | | | + | | + | | + | | + | | + | | | + | | + | | + | | + | | + | | + | | | + | | + | | + | | + | | + | | | + | | + | | + | | + | | + | + | **Chaperone DnaJ** |
| **SSGZ1_0278** | | | | | + | | + | | + | | + | | | + | | + | | + | | + | | + | | | + | | + | | + | | + | | + | | | + | | - | | + | | + | | + | | - | | | + | | + | | + | | + | | + | | | + | | + | | + | | + | | + | + | **Lipoate-protein ligase A** |
| **SSGZ1_0279** | | | | | + | | + | | + | | + | | | + | | + | | + | | + | | + | | | + | | + | | + | | + | | + | | | + | | + | | + | | + | | - | | + | | | + | | + | | + | | + | | + | | | + | | + | | + | | + | | + | + | **Amidase** |
| **SSGZ1_0280** | | | | | + | | + | | + | | + | | | + | | + | | + | | + | | + | | | + | | + | | + | | + | | + | | | + | | + | | + | | + | | + | | + | | | + | | + | | + | | + | | + | | | + | | + | | + | | + | | + | + | **extracellular solute-binding protein, family 3** |
| **SSGZ1_0281** | | | | | + | | + | | + | | + | | | + | | + | | + | | + | | + | | | + | | + | | + | | + | | + | | | + | | + | | + | | + | | + | | + | | | + | | + | | + | | + | | + | | | + | | + | | + | | + | | + | + | **Amino acid ABC transporter, permease protein** |
| **SSGZ1_0282** | | | | | + | | + | | + | | + | | | + | | + | | + | | + | | + | | | + | | + | | + | | + | | + | | | + | | + | | + | | + | | + | | + | | | + | | + | | + | | + | | + | | | + | | + | | + | | + | | + | + | **Helix-turn-helix motif** |
| **SSGZ1_0283** | | | | | + | | + | | + | | + | | | + | | + | | + | | + | | + | | | + | | + | | + | | + | | + | | | + | | + | | + | | + | | + | | + | | | + | | + | | + | | + | | - | | | - | | + | | - | | + | | + | + | **hypothetical protein** |
| **SSGZ1_0284** | | | | | + | | + | | + | | + | | | + | | + | | + | | + | | + | | | + | | + | | + | | + | | + | | | + | | + | | + | | + | | + | | + | | | + | | + | | + | | + | | + | | | + | | + | | + | | + | | + | + | **Heavy metal-(Cd/Co/Hg/Pb/Zn)-translocating** |
| **SSGZ1_0285** | | | | | + | | + | | + | | + | | | + | | + | | + | | + | | + | | | + | | + | | + | | + | | + | | | + | | + | | + | | + | | + | | + | | | + | | + | | + | | + | | + | | | + | | + | | + | | + | | + | + | **Ferric-uptake regulator** |
| **SSGZ1_0286** | | | | | + | | + | | + | | + | | | + | | + | | + | | + | | + | | | + | | + | | + | | + | | + | | | + | | + | | + | | + | | + | | + | | | + | | + | | + | | + | | + | | | + | | + | | + | | + | | + | + | **tRNA pseudouridine synthase** |
| **SSGZ1_0287** | | | | | + | | + | | + | | + | | | + | | + | | + | | + | | + | | | + | | + | | + | | + | | + | | | + | | + | | + | | + | | + | | + | | | + | | + | | + | | + | | + | | | + | | + | | + | | + | | + | + | **Phosphomethylpyrimidine kinase** |
| **SSGZ1_0288** | | | | | + | | + | | + | | + | | | + | | + | | + | | + | | + | | | + | | + | | + | | + | | + | | | + | | + | | + | | + | | + | | + | | | + | | + | | + | | + | | + | | | + | | + | | + | | + | | + | + | **Predicted membrane protein** |
| **SSGZ1_0289** | | | | | + | | + | | + | | + | | | + | | + | | + | | + | | + | | | + | | + | | + | | + | | + | | | + | | + | | + | | + | | + | | + | | | + | | + | | + | | + | | + | | | + | | + | | + | | + | | + | + | **hypothetical protein** |
| **SSGZ1_0290** | | | | | + | | + | | + | | + | | | + | | + | | + | | + | | + | | | + | | + | | + | | + | | + | | | + | | + | | + | | + | | + | | + | | | + | | + | | + | | + | | + | | | + | | + | | + | | + | | + | + | **hypothetical protein** |
| **SSGZ1_0291** | | | | | + | | + | | + | | + | | | + | | + | | + | | + | | + | | | + | | + | | + | | + | | + | | | + | | + | | + | | + | | + | | + | | | + | | + | | + | | + | | + | | | + | | + | | + | | + | | + | + | **hypothetical protein** |
| **SSGZ1_0292** | | | | | - | | + | | + | | + | | | + | | + | | + | | + | | + | | | + | | - | | + | | + | | + | | | + | | - | | + | | + | | + | | - | | | + | | + | | + | | + | | + | | | + | | - | | + | | + | | + | + | **hypothetical protein** |
| **SSGZ1_0293** | | | | | + | | + | | + | | + | | | + | | + | | + | | + | | + | | | + | | + | | + | | + | | + | | | + | | + | | + | | + | | + | | + | | | + | | + | | + | | + | | + | | | + | | + | | + | | + | | + | + | **Predicted transcriptional regulator** |
| **SSGZ1_0294** | | | | | + | | + | | + | | + | | | + | | + | | + | | + | | + | | | + | | + | | + | | + | | + | | | + | | + | | + | | + | | + | | + | | | + | | + | | + | | + | | + | | | + | | + | | + | | + | | + | + | **Zinc-containing alcohol dehydrogenase** |
| **SSGZ1_0295** | | | | | + | | + | | + | | + | | | + | | + | | + | | + | | + | | | + | | + | | + | | + | | + | | | + | | + | | + | | + | | + | | + | | | + | | + | | + | | + | | + | | | + | | + | | + | | + | | + | + | **hydrolase** |
| **SSGZ1_0296** | | | | | + | | + | | + | | + | | | + | | + | | + | | + | | + | | | + | | + | | + | | + | | + | | | + | | - | | + | | + | | - | | + | | | + | | + | | + | | + | | + | | | + | | + | | + | | + | | + | + | **Short-chain dehydrogenase/reductase SDR** |
| **SSGZ1_0297** | | | | | + | | + | | + | | + | | | + | | + | | + | | + | | + | | | + | | + | | + | | + | | + | | | + | | - | | + | | + | | - | | + | | | + | | + | | + | | + | | + | | | + | | - | | + | | + | | + | + | **NADH:flavin oxidoreductase/NADH oxidase** |
| **SSGZ1_0298** | | | | | - | | + | | - | | + | | | + | | + | | + | | + | | + | | | - | | + | | - | | - | | + | | | - | | - | | + | | + | | + | | + | | | + | | + | | + | | + | | + | | | + | | + | | + | | + | | + | + | **hypothetical protein** |
| **SSGZ1_0299** | | | | | + | | + | | + | | + | | | + | | + | | + | | + | | + | | | + | | + | | + | | + | | + | | | + | | - | | + | | + | | + | | + | | | + | | + | | + | | + | | + | | | + | | + | | + | | + | | + | + | **regulatory protein, TetR** |
| **SSGZ1_0300** | | | | | + | | + | | + | | + | | | + | | + | | + | | + | | + | | | + | | + | | + | | + | | + | | | + | | + | | + | | + | | + | | + | | | + | | + | | + | | + | | + | | | + | | + | | + | | + | | + | + | **hypothetical protein** |
| **SSGZ1_0301** | | | | | - | | + | | - | | + | | | + | | + | | + | | + | | + | | | - | | - | | - | | - | | - | | | - | | - | | + | | - | | - | | + | | | + | | + | | + | | - | | - | | | + | | + | | + | | + | | + | + | **lipase** |
| **SSGZ1_0302** | | | | | + | | + | | + | | + | | | + | | + | | + | | + | | + | | | + | | + | | + | | + | | + | | | + | | - | | + | | + | | + | | + | | | + | | + | | + | | + | | + | | | + | | + | | + | | + | | + | + | **acetyl esterase, putative** |
| **SSGZ1_0303** | | | | | + | | + | | + | | + | | | + | | + | | + | | + | | + | | | + | | + | | + | | + | | + | | | + | | + | | + | | + | | + | | + | | | + | | + | | + | | + | | + | | | + | | + | | + | | + | | + | + | **Trigger factor** |
| **SSGZ1_0304** | | | | | + | | + | | - | | + | | | + | | + | | + | | + | | + | | | - | | + | | + | | + | | + | | | + | | + | | + | | + | | + | | - | | | + | | + | | + | | - | | - | | | - | | + | | + | | + | | + | + | **glycosyl transferase, group 2 family protein** |
| **SSGZ1_0305** | | | | | + | | + | | + | | + | | | + | | + | | + | | + | | + | | | + | | + | | + | | + | | + | | | + | | + | | + | | + | | + | | + | | | + | | + | | + | | + | | + | | | + | | + | | + | | + | | + | + | **Periplasmic solute binding protein** |
| **SSGZ1_0306** | | | | | + | | + | | + | | + | | | + | | + | | + | | + | | + | | | + | | + | | + | | + | | + | | | + | | + | | + | | + | | + | | + | | | + | | + | | + | | + | | + | | | + | | + | | + | | + | | + | + | **Streptococcal histidine triad protein** |
| **SSGZ1_0307** | | | | | + | | + | | + | | + | | | + | | + | | + | | + | | + | | | + | | + | | + | | + | | + | | | + | | + | | + | | + | | + | | + | | | + | | + | | + | | + | | + | | | + | | + | | + | | + | | + | + | **DNA-directed RNA polymerase delta subunit** |
| **SSGZ1_0308** | | | | | + | | + | | + | | + | | | + | | + | | + | | + | | + | | | + | | + | | + | | + | | + | | | + | | + | | + | | + | | + | | + | | | + | | + | | + | | + | | + | | | + | | + | | + | | + | | + | + | **tRNA-Leu** |
| **SSGZ1_0309** | | | | | + | | + | | + | | + | | | + | | + | | + | | + | | + | | | + | | + | | + | | + | | + | | | + | | + | | + | | + | | + | | + | | | + | | + | | + | | + | | + | | | + | | + | | + | | + | | + | + | **Fructose-bisphosphate aldolase** |
| **SSGZ1_0310** | | | | | + | | + | | + | | + | | | + | | + | | + | | + | | + | | | + | | + | | + | | + | | + | | | + | | + | | + | | + | | + | | + | | | + | | + | | + | | + | | + | | | + | | + | | + | | + | | + | + | **tRNA-Glu** |
| **SSGZ1_0311** | | | | | + | | + | | + | | + | | | + | | + | | + | | + | | + | | | + | | + | | + | | + | | + | | | + | | + | | + | | + | | + | | + | | | + | | + | | + | | + | | + | | | + | | + | | + | | + | | + | + | **ATP-dependent DNA helicase RecG** |
| **SSGZ1_0312** | | | | | + | | + | | + | | + | | | + | | + | | + | | + | | + | | | + | | + | | + | | + | | + | | | + | | + | | + | | + | | + | | + | | | + | | + | | + | | + | | + | | | + | | + | | + | | + | | + | + | **Asparaginase/glutaminase** |
| **SSGZ1_0313** | | | | | + | | + | | + | | + | | | + | | + | | + | | + | | + | | | + | | + | | + | | + | | + | | | + | | + | | + | | + | | + | | + | | | + | | + | | + | | + | | + | | | + | | + | | + | | + | | + | + | **HAD-superfamily hydrolase, subfamily IIB** |
| **SSGZ1_0314** | | | | | + | | + | | + | | + | | | + | | + | | + | | + | | + | | | + | | + | | + | | + | | + | | | + | | + | | + | | + | | + | | + | | | + | | + | | + | | + | | + | | | + | | + | | + | | + | | + | + | **hypothetical protein** |
| **SSGZ1_0315** | | | | | + | | + | | + | | + | | | + | | + | | + | | + | | + | | | + | | + | | + | | + | | + | | | + | | + | | + | | + | | + | | + | | | + | | + | | + | | + | | + | | | + | | + | | + | | + | | + | + | **UspA** |
| **SSGZ1_0316** | | | | | + | | + | | + | | + | | | + | | + | | + | | + | | + | | | + | | + | | + | | + | | + | | | + | | + | | + | | + | | + | | + | | | + | | + | | + | | + | | + | | | + | | + | | + | | + | | + | + | **Aminotransferase, class I and II** |
| **SSGZ1_0317** | | | | | + | | + | | + | | + | | | + | | + | | + | | + | | + | | | + | | + | | + | | + | | + | | | + | | + | | + | | + | | + | | + | | | + | | + | | + | | + | | + | | | + | | + | | + | | + | | + | + | **GTP-sensing transcriptional pleiotropic** |
| **SSGZ1_0318** | | | | | + | | + | | + | | + | | | + | | + | | + | | + | | + | | | + | | + | | + | | + | | + | | | + | | + | | + | | + | | + | | + | | | + | | + | | + | | + | | + | | | + | | + | | + | | + | | + | + | **Isochorismatase hydrolase** |
| **SSGZ1_0319** | | | | | + | | + | | + | | + | | | + | | + | | + | | + | | + | | | + | | + | | + | | + | | + | | | + | | + | | + | | + | | + | | + | | | + | | + | | + | | + | | + | | | + | | + | | + | | + | | + | + | **ribosomal protein L19** |
| **SSGZ1_0320** | | | | | + | | + | | + | | + | | | + | | + | | + | | + | | + | | | + | | + | | + | | + | | + | | | + | | + | | + | | + | | + | | + | | | + | | + | | + | | + | | + | | | + | | + | | + | | + | | + | + | **hypothetical protein** |
| **SSGZ1_0321** | | | | | + | | + | | + | | + | | | + | | + | | + | | + | | + | | | + | | + | | + | | + | | + | | | + | | + | | + | | + | | + | | + | | | + | | + | | + | | + | | + | | | + | | + | | + | | + | | + | + | **Glutamyl-tRNA(Gln) amidotransferase C subunit** |
| **SSGZ1_0322** | | | | | + | | + | | + | | + | | | + | | + | | + | | + | | + | | | + | | + | | + | | + | | + | | | + | | + | | + | | + | | + | | + | | | + | | + | | + | | + | | + | | | + | | + | | + | | + | | + | + | **Glutamyl-tRNA(Gln) amidotransferase A subunit** |
| **SSGZ1_0323** | | | | | + | | + | | + | | + | | | + | | + | | + | | + | | + | | | + | | + | | + | | + | | + | | | + | | + | | + | | + | | + | | + | | | + | | + | | + | | + | | + | | | + | | + | | + | | + | | + | + | **Glutamyl-tRNA(Gln) amidotransferase B subunit** |
| **SSGZ1_0324** | | | | | - | | + | | + | | + | | | + | | + | | + | | + | | + | | | + | | - | | - | | - | | - | | | - | | + | | - | | + | | + | | + | | | + | | + | | + | | + | | + | | | + | | + | | + | | - | | + | - | **deoxyguanosinetriphosphate triphosphohydrolase-related protein** |
| **SSGZ1_0325** | | | | | + | | + | | + | | + | | | + | | + | | + | | + | | + | | | + | | + | | + | | + | | + | | | + | | + | | + | | + | | + | | + | | | + | | + | | + | | + | | + | | | + | | + | | + | | + | | + | + | **LacI transcriptional regulator** |
| **SSGZ1_0326** | | | | | + | | + | | + | | + | | | + | | + | | + | | + | | + | | | + | | + | | + | | + | | + | | | + | | + | | + | | + | | + | | + | | | + | | + | | + | | + | | + | | | + | | + | | + | | + | | + | + | **Galactokinase** |
| **SSGZ1_0327** | | | | | + | | + | | + | | + | | | + | | + | | + | | + | | + | | | + | | + | | + | | + | | + | | | + | | + | | + | | + | | + | | + | | | + | | + | | + | | + | | + | | | + | | + | | + | | + | | + | + | **Galactose-1-phosphate uridylyltransferase 2** |
| **SSGZ1_0328** | | | | | + | | + | | + | | + | | | + | | + | | + | | + | | + | | | + | | + | | + | | + | | + | | | + | | + | | + | | + | | + | | + | | | + | | + | | + | | + | | + | | | + | | + | | + | | + | | + | + | **Permease of the drug/metabolite transporter** |
| **SSGZ1_0329** | | | | | + | | + | | + | | + | | | + | | + | | + | | + | | + | | | + | | + | | + | | + | | + | | | + | | + | | + | | + | | + | | + | | | + | | + | | + | | + | | + | | | + | | + | | + | | + | | + | + | **CutC** |
| **SSGZ1_0330** | | | | | + | | + | | + | | + | | | + | | + | | + | | + | | + | | | + | | + | | + | | + | | + | | | + | | + | | + | | + | | + | | + | | | + | | + | | + | | + | | + | | | + | | + | | + | | + | | + | + | **hypothetical protein** |
| **SSGZ1_0331** | | | | | + | | + | | + | | + | | | + | | + | | + | | + | | + | | | + | | + | | + | | + | | + | | | + | | + | | + | | + | | + | | + | | | + | | + | | + | | + | | + | | | + | | + | | + | | + | | + | + | **HAD-superfamily hydrolase** |
| **SSGZ1_0332** | | | | | + | | + | | + | | + | | | + | | + | | + | | + | | + | | | + | | + | | + | | + | | + | | | + | | + | | + | | + | | + | | + | | | + | | + | | + | | + | | + | | | + | | + | | + | | + | | + | + | **GTP-binding protein** |
| **SSGZ1_0333** | | | | | + | | + | | + | | + | | | + | | + | | + | | + | | + | | | + | | + | | + | | + | | + | | | + | | + | | + | | + | | + | | + | | | + | | + | | + | | + | | + | | | + | | + | | + | | + | | + | + | **Protein of unknown function UPF0044** |
| **SSGZ1_0334** | | | | | + | | + | | + | | + | | | + | | + | | + | | + | | + | | | + | | + | | + | | + | | + | | | + | | + | | + | | + | | + | | + | | | + | | + | | + | | + | | + | | | + | | + | | + | | + | | + | + | **Cytidyltransferase-related protein: Probable** |
| **SSGZ1_0335** | | | | | + | | + | | + | | + | | | + | | + | | + | | + | | + | | | + | | + | | + | | + | | + | | | + | | + | | + | | + | | + | | + | | | + | | + | | + | | + | | + | | | + | | + | | + | | + | | + | + | **Predicted HD superfamily hydrolase involved in** |
| **SSGZ1_0336** | | | | | + | | + | | + | | + | | | + | | + | | + | | + | | + | | | + | | + | | + | | + | | + | | | + | | + | | + | | + | | + | | + | | | + | | + | | + | | + | | + | | | + | | + | | + | | + | | + | + | **GCN5-related N-acetyltransferase** |
| **SSGZ1_0337** | | | | | + | | + | | + | | + | | | + | | + | | + | | + | | + | | | + | | + | | + | | + | | + | | | + | | + | | + | | + | | + | | + | | | + | | + | | + | | + | | + | | | + | | + | | + | | + | | + | + | **Isochorismatase hydrolase** |
| **SSGZ1_0338** | | | | | + | | + | | + | | + | | | + | | + | | + | | + | | + | | | + | | + | | + | | + | | + | | | + | | + | | + | | + | | + | | + | | | + | | + | | + | | + | | + | | | + | | + | | + | | + | | + | + | **Iojap-related protein** |
| **SSGZ1_0339** | | | | | + | | + | | + | | + | | | + | | + | | + | | + | | + | | | + | | + | | + | | + | | + | | | + | | + | | + | | + | | + | | + | | | + | | + | | + | | + | | + | | | + | | + | | + | | + | | + | + | **SAM-dependent methyltransferase** |
| **SSGZ1_0340** | | | | | - | | + | | - | | + | | | + | | + | | + | | + | | + | | | - | | - | | + | | + | | - | | | - | | + | | - | | - | | - | | + | | | + | | - | | + | | - | | - | | | + | | + | | - | | + | | + | - | **hypothetical protein** |
| **SSGZ1_0341** | | | | | + | | + | | + | | + | | | + | | + | | + | | + | | + | | | + | | + | | + | | + | | + | | | + | | + | | + | | + | | + | | + | | | + | | + | | + | | + | | + | | | + | | + | | + | | + | | + | + | **Predicted nucleotidyltransferase** |
| **SSGZ1_0342** | | | | | - | | + | | - | | + | | | + | | + | | + | | + | | + | | | - | | - | | - | | - | | - | | | + | | + | | + | | - | | - | | + | | | + | | - | | + | | - | | - | | | + | | + | | - | | + | | - | - | **putative transcriptional regulator** |
| **SSGZ1_0343** | | | | | - | | + | | - | | + | | | + | | + | | + | | + | | + | | | - | | - | | - | | - | | - | | | - | | - | | - | | - | | - | | - | | | + | | - | | - | | - | | - | | | - | | + | | - | | - | | - | - | **hypothetical protein** |
| **SSGZ1_0344** | | | | | - | | + | | - | | + | | | + | | + | | + | | + | | + | | | - | | - | | - | | - | | - | | | - | | - | | - | | - | | - | | - | | | + | | - | | - | | - | | - | | | - | | - | | - | | - | | - | - | **putative ATP-binding protein** |
| **SSGZ1_0345** | | | | | - | | + | | - | | + | | | + | | + | | + | | + | | + | | | - | | - | | - | | - | | - | | | - | | - | | - | | - | | - | | - | | | + | | - | | - | | - | | - | | | - | | + | | - | | - | | - | - | **hypothetical protein** |
| **SSGZ1_0346** | | | | | - | | + | | - | | + | | | + | | + | | + | | + | | + | | | - | | - | | - | | - | | - | | | - | | - | | - | | - | | - | | + | | | + | | - | | + | | - | | - | | | - | | + | | - | | - | | - | - | **hypothetical protein** |
| **SSGZ1_0347** | | | | | + | | + | | + | | + | | | + | | + | | + | | + | | + | | | + | | + | | + | | + | | + | | | + | | + | | + | | + | | + | | + | | | + | | + | | + | | + | | + | | | + | | + | | + | | + | | + | + | **Formylmethionine deformylase** |
| **SSGZ1_0348** | | | | | + | | + | | + | | + | | | + | | + | | + | | + | | + | | | + | | + | | + | | + | | + | | | + | | + | | + | | + | | + | | + | | | + | | + | | + | | + | | + | | | + | | + | | + | | + | | + | + | **AAA ATPase, central region** |
| **SSGZ1_0349** | | | | | + | | + | | + | | + | | | + | | + | | + | | + | | + | | | + | | + | | + | | + | | + | | | + | | + | | + | | + | | + | | + | | | + | | + | | + | | + | | + | | | + | | + | | + | | + | | + | + | **Glycoside hydrolase, family 77** |
| **SSGZ1_0350** | | | | | + | | + | | + | | + | | | + | | + | | + | | + | | + | | | + | | + | | + | | + | | + | | | + | | + | | + | | + | | + | | + | | | + | | + | | + | | + | | + | | | + | | + | | + | | + | | + | + | **Phosphorylase** |
| **SSGZ1_0351** | | | | | + | | + | | + | | + | | | + | | + | | + | | + | | + | | | + | | + | | + | | + | | + | | | + | | + | | + | | + | | + | | + | | | + | | + | | + | | + | | + | | | + | | + | | + | | + | | + | + | **Bacterial regulatory protein, GntR** |
| **SSGZ1_0352** | | | | | + | | + | | + | | + | | | + | | + | | + | | + | | + | | | + | | + | | + | | + | | + | | | + | | + | | + | | + | | + | | + | | | + | | + | | + | | + | | + | | | + | | + | | + | | + | | + | + | **phosphatase** |
| **SSGZ1_0353** | | | | | + | | + | | + | | + | | | + | | + | | + | | + | | + | | | + | | + | | + | | + | | + | | | + | | + | | + | | + | | + | | + | | | + | | + | | + | | + | | + | | | + | | + | | + | | + | | + | + | **Sugar-specific permease** |
| **SSGZ1_0354** | | | | | + | | + | | + | | + | | | + | | + | | + | | + | | + | | | + | | + | | + | | + | | + | | | + | | + | | - | | + | | + | | + | | | + | | + | | + | | + | | + | | | + | | + | | + | | - | | + | + | **hypothetical protein** |
| **SSGZ1_0355** | | | | | + | | + | | + | | + | | | + | | + | | + | | + | | + | | | + | | + | | + | | + | | + | | | + | | + | | - | | + | | + | | + | | | + | | + | | + | | + | | + | | | + | | + | | + | | - | | + | + | **Transcriptional regulator PadR-like protein** |
| **SSGZ1_0356** | | | | | + | | + | | + | | + | | | + | | + | | + | | + | | + | | | + | | + | | + | | + | | + | | | + | | + | | + | | + | | + | | + | | | + | | + | | + | | + | | + | | | + | | + | | + | | + | | + | + | **Protein of unknown function DUF28** |
| **SSGZ1_0357** | | | | | + | | + | | + | | + | | | + | | + | | + | | + | | + | | | + | | + | | + | | + | | + | | | + | | + | | + | | + | | + | | + | | | + | | + | | + | | + | | + | | | + | | + | | + | | + | | + | + | **Protein of unknown function DUF62** |
| **SSGZ1_0358** | | | | | + | | + | | + | | + | | | + | | + | | + | | + | | + | | | + | | + | | + | | + | | + | | | + | | + | | + | | + | | + | | + | | | + | | + | | + | | + | | + | | | + | | + | | + | | + | | + | + | **Predicted membrane protein** |
| **SSGZ1_0359** | | | | | + | | + | | + | | + | | | + | | + | | + | | + | | + | | | + | | + | | + | | + | | + | | | + | | + | | + | | + | | + | | + | | | + | | + | | + | | + | | + | | | + | | + | | + | | + | | + | + | **hypothetical protein** |
| **SSGZ1_0360** | | | | | + | | + | | + | | + | | | + | | + | | + | | + | | + | | | + | | + | | + | | + | | + | | | + | | + | | + | | + | | + | | + | | | + | | + | | + | | + | | + | | | + | | + | | + | | + | | + | + | **Rhodanese-like protein** |
| **SSGZ1_0361** | | | | | + | | + | | + | | + | | | + | | + | | + | | + | | + | | | + | | + | | + | | + | | + | | | + | | + | | + | | + | | + | | + | | | + | | + | | + | | + | | + | | | + | | + | | + | | + | | + | + | **similar to lactoylglutathione lyase** |
| **SSGZ1_0362** | | | | | + | | + | | + | | + | | | + | | + | | + | | + | | + | | | + | | + | | + | | + | | + | | | + | | + | | + | | + | | + | | + | | | + | | + | | + | | + | | + | | | + | | + | | + | | + | | + | + | **ABC transporter** |
| **SSGZ1_0363** | | | | | + | | + | | + | | + | | | + | | + | | + | | + | | + | | | + | | + | | + | | + | | + | | | + | | + | | + | | + | | + | | + | | | + | | + | | + | | + | | + | | | + | | + | | + | | + | | + | + | **Cobalt transport protein** |
| **SSGZ1_0364** | | | | | + | | + | | + | | + | | | + | | + | | + | | + | | + | | | + | | + | | + | | + | | + | | | + | | + | | + | | + | | + | | + | | | + | | + | | + | | + | | + | | | + | | + | | + | | + | | + | + | **Cold shock protein** |
| **SSGZ1_0365** | | | | | + | | + | | + | | + | | | + | | + | | + | | + | | + | | | + | | + | | + | | + | | + | | | + | | + | | + | | + | | + | | + | | | + | | + | | + | | + | | + | | | + | | + | | + | | + | | + | + | **Glucose inhibited cell division protein** |
| **SSGZ1_0366** | | | | | + | | + | | + | | + | | | + | | + | | + | | + | | + | | | + | | + | | + | | + | | + | | | + | | + | | + | | + | | + | | + | | | + | | + | | + | | + | | + | | | + | | + | | + | | + | | + | + | **Glycosyl transferase, family 51** |
| **SSGZ1_0367** | | | | | + | | + | | + | | + | | | + | | + | | + | | + | | + | | | + | | + | | + | | + | | + | | | + | | + | | + | | + | | + | | + | | | + | | + | | + | | + | | + | | | + | | + | | + | | + | | + | + | **Recombination protein U** |
| **SSGZ1_0368** | | | | | + | | + | | + | | + | | | + | | + | | + | | + | | + | | | + | | + | | + | | + | | + | | | + | | + | | + | | + | | + | | + | | | + | | + | | + | | + | | + | | | + | | + | | + | | + | | + | + | **Protein of unknown function DUF1273** |
| **SSGZ1_0369** | | | | | + | | + | | + | | + | | | + | | + | | + | | + | | + | | | + | | + | | + | | + | | + | | | + | | + | | + | | + | | + | | + | | | + | | + | | + | | + | | + | | | + | | + | | + | | + | | + | + | **DivIVA** |
| **SSGZ1_0370** | | | | | + | | + | | + | | + | | | + | | + | | + | | + | | + | | | + | | + | | + | | + | | + | | | + | | + | | + | | + | | + | | + | | | + | | + | | + | | + | | + | | | + | | + | | + | | + | | + | + | **hypothetical protein** |
| **SSGZ1_0371** | | | | | + | | + | | + | | + | | | + | | + | | + | | + | | + | | | + | | + | | + | | + | | + | | | + | | + | | + | | + | | + | | + | | | + | | + | | + | | + | | + | | | + | | + | | + | | + | | + | + | **Putative RNA methylase** |
| **SSGZ1_0372** | | | | | + | | + | | + | | + | | | + | | + | | + | | + | | + | | | + | | + | | + | | + | | + | | | + | | + | | + | | + | | + | | + | | | + | | + | | + | | + | | + | | | + | | + | | + | | + | | + | + | **hypothetical protein** |
| **SSGZ1_0373** | | | | | + | | + | | + | | + | | | + | | + | | + | | + | | + | | | + | | + | | + | | + | | + | | | + | | + | | + | | + | | + | | + | | | + | | + | | + | | + | | + | | | + | | + | | + | | + | | + | + | **LuxS protein** |
| **SSGZ1_0374** | | | | | + | | + | | + | | + | | | + | | + | | + | | + | | + | | | + | | + | | + | | + | | + | | | + | | + | | + | | + | | + | | + | | | + | | + | | + | | + | | + | | | + | | + | | + | | + | | + | + | **HDIG** |
| **SSGZ1_0375** | | | | | + | | + | | + | | + | | | + | | + | | + | | + | | + | | | + | | + | | + | | + | | + | | | + | | + | | + | | + | | + | | + | | | + | | + | | + | | + | | + | | | + | | + | | + | | + | | + | + | **Guanylate kinase** |
| **SSGZ1_0376** | | | | | + | | + | | + | | + | | | + | | + | | + | | + | | + | | | + | | + | | + | | + | | + | | | + | | + | | + | | + | | + | | + | | | + | | + | | + | | + | | + | | | + | | + | | + | | + | | + | + | **RNA polymerase, omega subunit** |
| **SSGZ1_0377** | | | | | + | | + | | + | | + | | | + | | + | | + | | + | | + | | | + | | + | | + | | + | | + | | | + | | + | | + | | + | | + | | + | | | + | | + | | + | | + | | + | | | + | | + | | + | | + | | + | + | **Primosomal protein N** |
| **SSGZ1_0378** | | | | | + | | + | | + | | + | | | + | | + | | + | | + | | + | | | + | | + | | + | | + | | + | | | + | | + | | + | | + | | + | | + | | | + | | + | | + | | + | | + | | | + | | + | | + | | + | | + | + | **Methionyl-tRNA formyltransferase** |
| **SSGZ1_0379** | | | | | + | | + | | + | | + | | | + | | + | | + | | + | | + | | | + | | + | | + | | + | | + | | | + | | + | | + | | + | | + | | + | | | + | | + | | + | | + | | + | | | + | | + | | + | | + | | + | + | **rRNA SAM-dependent methyltransferase** |
| **SSGZ1_0380** | | | | | + | | + | | + | | + | | | + | | + | | + | | + | | + | | | + | | + | | + | | + | | + | | | + | | + | | + | | + | | + | | + | | | + | | + | | + | | + | | + | | | + | | + | | + | | + | | + | + | **phosphatase 2C-like-protein** |
| **SSGZ1_0381** | | | | | + | | + | | + | | + | | | + | | + | | + | | + | | + | | | + | | + | | + | | + | | + | | | + | | + | | + | | + | | + | | + | | | + | | + | | + | | + | | + | | | + | | + | | + | | + | | + | + | **PASTA** |
| **SSGZ1_0382** | | | | | + | | + | | + | | + | | | + | | + | | + | | + | | + | | | + | | + | | + | | + | | + | | | + | | + | | + | | + | | + | | + | | | + | | + | | + | | + | | + | | | + | | + | | + | | + | | + | + | **Predicted membrane protein** |
| **SSGZ1_0383** | | | | | + | | + | | + | | + | | | + | | + | | + | | + | | + | | | + | | + | | + | | + | | + | | | + | | + | | + | | + | | + | | + | | | + | | + | | + | | + | | + | | | + | | + | | + | | + | | + | + | **ATPase-like protein** |
| **SSGZ1_0384** | | | | | + | | + | | + | | + | | | + | | + | | + | | + | | + | | | + | | + | | + | | + | | + | | | + | | + | | + | | + | | + | | + | | | + | | + | | + | | + | | + | | | + | | + | | + | | + | | + | + | **regulatory protein, LuxR: Response regulator** |
| **SSGZ1_0385** | | | | | + | | + | | + | | + | | | + | | + | | + | | + | | + | | | + | | + | | + | | + | | + | | | + | | + | | + | | + | | + | | + | | | + | | + | | + | | + | | + | | | + | | + | | + | | + | | + | + | **Cof protein: HAD-superfamily hydrolase,** |
| **SSGZ1_0386** | | | | | + | | + | | + | | + | | | + | | + | | + | | + | | + | | | + | | + | | + | | + | | + | | | + | | + | | + | | + | | + | | + | | | + | | + | | + | | + | | + | | | + | | + | | + | | + | | + | + | **RNA binding S1** |
| **SSGZ1_0387** | | | | | + | | + | | + | | + | | | + | | + | | + | | + | | + | | | + | | + | | + | | + | | + | | | + | | + | | + | | + | | + | | + | | | + | | + | | + | | + | | + | | | + | | + | | + | | + | | + | + | **O-acetylserine lyase** |
| **SSGZ1_0388** | | | | | + | | + | | + | | + | | | + | | + | | + | | + | | + | | | + | | + | | + | | + | | + | | | + | | + | | + | | + | | + | | + | | | + | | + | | + | | + | | + | | | + | | + | | + | | + | | + | + | **hypothetical protein** |
| **SSGZ1_0389** | | | | | + | | + | | + | | + | | | + | | + | | + | | + | | + | | | + | | + | | + | | + | | + | | | + | | + | | + | | + | | + | | + | | | + | | + | | + | | + | | + | | | + | | + | | + | | + | | + | + | **Helicase** |
| **SSGZ1_0390** | | | | | + | | + | | + | | + | | | + | | + | | + | | + | | + | | | + | | + | | + | | + | | + | | | + | | + | | + | | + | | + | | + | | | + | | + | | + | | + | | + | | | + | | + | | + | | + | | + | + | **putative late competence protein** |
| **SSGZ1_0391** | | | | | + | | + | | + | | + | | | + | | + | | + | | + | | + | | | + | | + | | + | | + | | + | | | + | | + | | + | | + | | + | | + | | | + | | + | | + | | + | | + | | | + | | + | | + | | + | | + | + | **tRNA-Pro** |
| **SSGZ1_0392** | | | | | + | | + | | + | | + | | | + | | + | | + | | + | | + | | | + | | + | | + | | + | | + | | | + | | + | | + | | + | | + | | + | | | + | | + | | + | | + | | + | | | + | | + | | + | | + | | + | + | **hypothetical protein** |
| **SSGZ1_0393** | | | | | + | | + | | + | | + | | | + | | + | | + | | + | | + | | | + | | + | | + | | + | | + | | | + | | + | | + | | + | | + | | + | | | + | | + | | + | | + | | + | | | + | | + | | + | | + | | + | + | **tRNA (uracil-5-)-methyltransferase** |
| **SSGZ1_0394** | | | | | + | | + | | + | | + | | | + | | + | | + | | + | | + | | | + | | + | | + | | + | | + | | | + | | + | | + | | + | | + | | + | | | + | | + | | + | | + | | + | | | + | | + | | + | | + | | + | + | **Regulatory protein RecX** |
| **SSGZ1_0395** | | | | | + | | + | | + | | + | | | + | | + | | + | | + | | + | | | + | | + | | + | | + | | + | | | + | | + | | + | | + | | + | | + | | | + | | + | | + | | + | | + | | | + | | + | | + | | + | | + | + | **Protein of unknown function DUF402** |
| **SSGZ1_0396** | | | | | + | | + | | + | | + | | | + | | + | | + | | + | | + | | | + | | + | | + | | + | | + | | | + | | + | | + | | + | | + | | + | | | + | | + | | + | | + | | + | | | + | | + | | + | | + | | + | + | **hypothetical protein** |
| **SSGZ1_0397** | | | | | + | | + | | + | | + | | | + | | + | | + | | + | | + | | | + | | + | | + | | + | | + | | | + | | + | | + | | + | | + | | + | | | + | | + | | + | | + | | + | | | + | | + | | + | | + | | + | + | **transcriptional regulator, GntR family** |
| **SSGZ1_0398** | | | | | + | | + | | + | | + | | | + | | + | | + | | + | | + | | | + | | + | | + | | + | | + | | | + | | + | | + | | + | | + | | + | | | + | | + | | + | | + | | + | | | + | | + | | + | | + | | + | + | **transcriptional regulator, GntR family** |
| **SSGZ1_0399** | | | | | + | | + | | + | | + | | | + | | + | | + | | + | | + | | | + | | + | | + | | + | | + | | | + | | + | | + | | + | | + | | + | | | + | | + | | + | | + | | + | | | + | | + | | + | | + | | + | + | **Beta-galactosidase** |
| **SSGZ1_0400** | | | | | + | | + | | + | | + | | | + | | + | | + | | + | | + | | | + | | + | | + | | + | | + | | | + | | + | | + | | + | | + | | + | | | + | | + | | + | | + | | + | | | + | | + | | + | | + | | + | + | **PTS system sorbose subfamily IIB component** |
| **SSGZ1_0401** | | | | | + | | + | | + | | + | | | + | | + | | + | | + | | + | | | + | | + | | + | | + | | + | | | + | | + | | + | | + | | + | | + | | | + | | + | | + | | + | | + | | | + | | + | | + | | + | | + | + | **sorbose-specific IIC subunit** |
| **SSGZ1_0402** | | | | | + | | + | | + | | + | | | + | | + | | + | | + | | + | | | + | | + | | + | | + | | + | | | + | | + | | + | | + | | + | | + | | | + | | + | | + | | + | | + | | | + | | + | | + | | + | | + | + | **PTS system mannose family IID component protein** |
| **SSGZ1_0403** | | | | | + | | + | | + | | + | | | + | | + | | + | | + | | + | | | + | | + | | + | | + | | + | | | + | | + | | + | | + | | + | | + | | | + | | + | | + | | + | | + | | | + | | + | | + | | + | | + | + | **PTS system fructose subfamily IIA component** |
| **SSGZ1_0404** | | | | | + | | + | | + | | + | | | + | | + | | + | | + | | + | | | + | | + | | + | | + | | + | | | + | | + | | + | | + | | + | | + | | | + | | + | | + | | + | | + | | | + | | + | | + | | + | | + | + | **Aldose 1-epimerase** |
| **SSGZ1_0405** | | | | | + | | + | | + | | + | | | + | | + | | + | | + | | + | | | + | | + | | + | | + | | + | | | + | | + | | + | | + | | + | | + | | | + | | + | | + | | + | | + | | | + | | + | | + | | + | | + | + | **hypothetical protein** |
| **SSGZ1_0406** | | | | | + | | + | | + | | + | | | + | | + | | + | | + | | + | | | + | | + | | + | | + | | + | | | + | | + | | + | | + | | + | | + | | | + | | + | | + | | + | | + | | | + | | + | | + | | + | | + | + | **Superfamily II helicase** |
| **SSGZ1_0407** | | | | | + | | + | | + | | + | | | + | | + | | + | | + | | + | | | + | | + | | + | | + | | + | | | + | | + | | + | | + | | + | | + | | | + | | + | | + | | + | | + | | | + | | + | | + | | + | | + | + | **dioxygenase** |
| **SSGZ1_0408** | | | | | + | | + | | + | | + | | | + | | + | | + | | + | | + | | | + | | + | | + | | + | | + | | | + | | + | | + | | - | | + | | + | | | + | | + | | + | | + | | + | | | + | | + | | + | | + | | + | + | **Shikimate kinase** |
| **SSGZ1_0409** | | | | | + | | + | | + | | + | | | + | | + | | + | | + | | + | | | + | | + | | + | | + | | + | | | + | | + | | + | | + | | + | | + | | | + | | + | | + | | + | | + | | | + | | + | | + | | + | | + | + | **Valyl-tRNA synthetase, class Ia** |
| **SSGZ1_0410** | | | | | - | | + | | - | | + | | | + | | + | | + | | + | | + | | | - | | - | | - | | + | | - | | | + | | + | | - | | - | | - | | + | | | + | | + | | + | | - | | - | | | + | | + | | - | | + | | + | - | **hypothetical protein** |
| **SSGZ1_0411** | | | | | - | | + | | - | | + | | | + | | + | | + | | + | | + | | | - | | - | | + | | + | | - | | | + | | + | | - | | + | | + | | - | | | + | | + | | - | | + | | + | | | + | | + | | + | | - | | + | + | **cell filamentation protein Fic-related protein** |
| **SSGZ1_0412** | | | | | - | | + | | - | | + | | | + | | + | | + | | + | | + | | | - | | - | | + | | + | | - | | | - | | + | | - | | - | | - | | - | | | + | | + | | - | | + | | + | | | + | | + | | + | | - | | - | + | **DNA or RNA helicases of superfamily II** |
| **SSGZ1_0413** | | | | | - | | + | | - | | + | | | + | | + | | + | | + | | + | | | - | | - | | + | | + | | - | | | - | | + | | - | | - | | - | | + | | | + | | + | | - | | + | | + | | | + | | + | | + | | - | | - | + | **hypothetical protein** |
| **SSGZ1_0414** | | | | | - | | + | | - | | + | | | + | | + | | + | | + | | + | | | - | | - | | + | | + | | - | | | - | | + | | - | | - | | - | | - | | | + | | + | | + | | + | | + | | | + | | + | | + | | - | | - | + | **transcriptional regulator** |
| **SSGZ1_0415** | | | | | + | | + | | + | | + | | | + | | + | | + | | + | | + | | | + | | + | | + | | + | | + | | | + | | + | | + | | + | | + | | + | | | + | | + | | + | | + | | + | | | + | | + | | + | | + | | + | + | **hypothetical protein** |
| **SSGZ1_0416** | | | | | + | | + | | + | | + | | | + | | + | | + | | + | | + | | | + | | + | | + | | + | | + | | | + | | + | | + | | + | | + | | + | | | + | | + | | + | | + | | + | | | + | | + | | + | | + | | + | + | **aspartate--ammonia ligase** |
| **SSGZ1_0417** | | | | | + | | + | | + | | + | | | + | | + | | + | | + | | + | | | + | | + | | + | | + | | + | | | + | | + | | + | | + | | + | | + | | | + | | + | | + | | + | | + | | | + | | + | | + | | + | | + | + | **GTP-binding protein TypA** |
| **SSGZ1_0418** | | | | | + | | + | | + | | + | | | + | | + | | + | | + | | + | | | + | | + | | + | | + | | + | | | + | | + | | + | | + | | + | | + | | | + | | + | | + | | + | | + | | | + | | + | | + | | + | | + | + | **hypothetical protein** |
| **SSGZ1_0419** | | | | | + | | + | | + | | + | | | + | | + | | + | | + | | + | | | + | | + | | + | | + | | + | | | + | | + | | + | | + | | + | | + | | | + | | + | | + | | + | | + | | | + | | + | | + | | + | | + | + | **hypothetical protein** |
| **SSGZ1_0420** | | | | | + | | + | | + | | + | | | + | | + | | + | | + | | + | | | + | | + | | + | | + | | - | | | + | | - | | - | | + | | + | | - | | | + | | + | | - | | + | | + | | | + | | - | | + | | - | | - | - | **signal peptidase IB** |
| **SSGZ1_0421** | | | | | + | | + | | - | | + | | | + | | + | | + | | + | | + | | | - | | + | | - | | - | | - | | | - | | - | | - | | + | | + | | - | | | + | | - | | - | | - | | - | | | - | | - | | - | | - | | - | - | **Ribonucleases G and E** |
| **SSGZ1_0422** | | | | | + | | + | | + | | + | | | + | | + | | + | | + | | + | | | + | | + | | - | | - | | - | | | + | | - | | - | | + | | + | | - | | | + | | - | | - | | - | | - | | | - | | - | | - | | - | | - | - | **Ribonucleases G and E** |
| **SSGZ1_0423** | | | | | + | | + | | + | | + | | | + | | + | | + | | + | | + | | | + | | + | | - | | + | | - | | | + | | - | | - | | + | | - | | - | | | + | | + | | - | | + | | + | | | + | | + | | + | | - | | - | - | **Surface protein from Gram-positive cocci** |
| **SSGZ1_0424** | | | | | + | | + | | + | | + | | | + | | + | | + | | + | | + | | | + | | + | | + | | + | | - | | | + | | - | | - | | + | | + | | + | | | + | | + | | + | | + | | + | | | + | | + | | + | | - | | - | - | **sortase** |
| **SSGZ1_0425** | | | | | + | | + | | + | | + | | | + | | + | | + | | + | | + | | | + | | + | | + | | + | | + | | | + | | + | | - | | + | | + | | + | | | + | | + | | + | | + | | + | | | + | | + | | + | | + | | + | + | **putative transposase** |
| **SSGZ1_0426** | | | | | + | | + | | + | | + | | | + | | + | | + | | + | | + | | | + | | + | | + | | + | | + | | | + | | + | | + | | + | | + | | + | | | + | | + | | + | | + | | + | | | + | | + | | + | | + | | + | + | **UDP-N-acetylmuramoylalanine-D-glutamate ligase** |
| **SSGZ1_0427** | | | | | + | | + | | + | | + | | | + | | + | | + | | + | | + | | | + | | + | | + | | + | | + | | | + | | + | | + | | + | | + | | + | | | + | | + | | + | | + | | + | | | + | | + | | + | | + | | + | + | **Undecaprenyldiphospho-muramoylpentapeptide** |
| **SSGZ1_0428** | | | | | + | | + | | + | | + | | | + | | + | | + | | + | | + | | | + | | + | | + | | + | | + | | | + | | + | | + | | + | | + | | + | | | + | | + | | + | | + | | + | | | + | | + | | + | | + | | + | + | **Cell division protein FtsQ** |
| **SSGZ1_0429** | | | | | + | | + | | + | | + | | | + | | + | | + | | + | | + | | | + | | + | | + | | + | | + | | | + | | + | | + | | + | | + | | + | | | + | | + | | + | | + | | + | | | + | | + | | + | | + | | + | + | **cell division protein FtsA** |
| **SSGZ1_0430** | | | | | + | | + | | + | | + | | | + | | + | | + | | + | | + | | | + | | + | | + | | + | | + | | | + | | + | | + | | + | | + | | + | | | + | | + | | + | | + | | + | | | + | | + | | + | | + | | + | + | **Cell division protein FtsZ** |
| **SSGZ1_0431** | | | | | + | | + | | + | | + | | | + | | + | | + | | + | | + | | | + | | + | | + | | + | | + | | | + | | + | | + | | + | | + | | + | | | + | | + | | + | | + | | + | | | + | | + | | + | | + | | + | + | **Predicted enzyme with a TIM-barrel fold** |
| **SSGZ1_0432** | | | | | + | | + | | + | | + | | | + | | + | | + | | + | | + | | | + | | + | | + | | + | | + | | | + | | + | | + | | + | | + | | + | | | + | | + | | + | | + | | + | | | + | | + | | + | | + | | + | + | **hypothetical protein** |
| **SSGZ1_0433** | | | | | + | | + | | + | | + | | | + | | + | | + | | + | | + | | | + | | + | | + | | + | | + | | | + | | + | | + | | + | | + | | + | | | + | | + | | + | | + | | + | | | + | | + | | + | | + | | + | + | **YGGT family protein** |
| **SSGZ1_0434** | | | | | + | | + | | + | | + | | | + | | + | | + | | + | | + | | | + | | + | | + | | + | | + | | | + | | + | | + | | + | | + | | + | | | + | | + | | + | | + | | + | | | + | | + | | + | | + | | + | + | **RNA-binding S4** |
| **SSGZ1_0435** | | | | | + | | + | | + | | + | | | + | | + | | + | | + | | + | | | + | | + | | + | | + | | + | | | + | | + | | + | | + | | + | | + | | | + | | + | | + | | + | | + | | | + | | + | | + | | + | | + | + | **DivIVA** |
| **SSGZ1_0436** | | | | | + | | + | | + | | + | | | + | | + | | + | | + | | + | | | + | | + | | + | | + | | + | | | + | | + | | + | | + | | + | | + | | | + | | + | | + | | + | | + | | | + | | + | | + | | + | | + | + | **GCN5-related N-acetyltransferase** |
| **SSGZ1_0437** | | | | | + | | + | | + | | + | | | + | | + | | + | | + | | + | | | + | | + | | + | | + | | + | | | + | | + | | + | | + | | + | | + | | | + | | + | | + | | + | | + | | | + | | + | | + | | + | | + | + | **Isoleucyl-tRNA synthetase, class Ia** |
| **SSGZ1_0438** | | | | | + | | + | | + | | + | | | + | | + | | + | | + | | + | | | + | | + | | + | | + | | + | | | + | | + | | + | | + | | + | | + | | | + | | + | | + | | + | | + | | | + | | + | | + | | + | | + | + | **hypothetical protein** |
| **SSGZ1_0439** | | | | | + | | + | | + | | + | | | + | | + | | + | | + | | + | | | + | | + | | + | | + | | + | | | + | | + | | + | | + | | + | | + | | | + | | + | | + | | + | | + | | | + | | + | | + | | + | | + | + | **putative hydrolase** |
| **SSGZ1_0440** | | | | | + | | + | | + | | + | | | + | | + | | + | | + | | + | | | + | | + | | + | | + | | + | | | + | | + | | + | | + | | + | | + | | | + | | + | | + | | + | | + | | | + | | + | | + | | + | | + | + | **UvrB/UvrC protein** |
| **SSGZ1_0441** | | | | | + | | + | | + | | + | | | + | | + | | + | | + | | + | | | + | | + | | + | | + | | + | | | + | | + | | + | | + | | + | | + | | | + | | + | | + | | + | | + | | | + | | + | | + | | + | | + | + | **hypothetical protein** |
| **SSGZ1_0442** | | | | | + | | + | | + | | + | | | + | | + | | + | | + | | + | | | + | | + | | + | | + | | + | | | + | | + | | + | | + | | + | | + | | | + | | + | | + | | + | | + | | | + | | + | | + | | + | | + | + | **permease protein** |
| **SSGZ1_0443** | | | | | + | | + | | + | | + | | | + | | + | | + | | + | | + | | | + | | + | | + | | + | | + | | | + | | + | | + | | + | | + | | + | | | + | | + | | + | | + | | + | | | + | | + | | + | | + | | + | + | **putative amino acid ABC transporter, ATP-binding** |
| **SSGZ1_0444** | | | | | + | | + | | + | | + | | | + | | + | | + | | + | | + | | | + | | + | | + | | + | | + | | | + | | + | | + | | + | | + | | + | | | + | | + | | + | | + | | + | | | + | | + | | + | | + | | + | + | **methylenetetrahydrofolate dehydrogenase** |
| **SSGZ1_0445** | | | | | + | | + | | - | | + | | | + | | + | | + | | + | | + | | | - | | + | | + | | + | | + | | | + | | - | | + | | + | | + | | - | | | + | | + | | + | | + | | + | | | + | | + | | + | | + | | + | + | **hypothetical protein** |
| **SSGZ1_0446** | | | | | + | | + | | - | | + | | | + | | + | | + | | + | | + | | | - | | + | | + | | + | | + | | | + | | + | | + | | + | | + | | + | | | + | | + | | + | | + | | + | | | + | | + | | + | | + | | + | + | **putative signal peptidase IB** |
| **SSGZ1_0447** | | | | | + | | + | | - | | + | | | + | | + | | + | | + | | + | | | - | | + | | + | | + | | + | | | + | | - | | + | | + | | + | | - | | | + | | + | | + | | + | | + | | | + | | + | | + | | + | | + | + | **hypothetical protein** |
| **SSGZ1_0448** | | | | | + | | + | | + | | + | | | + | | + | | + | | + | | + | | | + | | + | | + | | + | | + | | | + | | + | | + | | + | | + | | + | | | + | | + | | + | | + | | + | | | + | | + | | + | | + | | + | + | **Transposase, IS4** |
| **SSGZ1_0449** | | | | | + | | + | | + | | + | | | + | | + | | + | | + | | + | | | + | | + | | + | | + | | + | | | + | | - | | + | | + | | + | | + | | | + | | + | | + | | + | | + | | | + | | + | | + | | + | | + | + | **sortase-like protein/sortase A?** |
| **SSGZ1_0450** | | | | | + | | + | | + | | + | | | + | | + | | + | | + | | + | | | + | | + | | + | | + | | + | | | + | | + | | + | | + | | + | | + | | | + | | + | | + | | + | | + | | | + | | + | | + | | + | | + | + | **hypothetical protein** |
| **SSGZ1_0451** | | | | | + | | + | | + | | + | | | + | | + | | + | | + | | + | | | + | | + | | + | | + | | + | | | + | | + | | + | | + | | + | | + | | | + | | + | | + | | + | | + | | | + | | + | | + | | + | | + | + | **putative mercuric resisitant regulatory protein** |
| **SSGZ1_0452** | | | | | + | | + | | + | | + | | | + | | + | | + | | + | | + | | | + | | + | | + | | + | | + | | | + | | + | | + | | + | | + | | + | | | + | | + | | + | | + | | + | | | + | | + | | + | | + | | + | + | **hypothetical protein** |
| **SSGZ1_0453** | | | | | + | | + | | + | | + | | | + | | + | | + | | + | | + | | | + | | + | | + | | + | | + | | | + | | + | | + | | + | | + | | + | | | + | | + | | + | | + | | + | | | + | | + | | + | | + | | + | + | **Peptidase U32** |
| **SSGZ1_0454** | | | | | + | | + | | + | | + | | | + | | + | | + | | + | | + | | | + | | + | | + | | + | | + | | | + | | + | | + | | + | | + | | + | | | + | | + | | + | | + | | + | | | + | | + | | + | | + | | + | + | **Peptidase U32** |
| **SSGZ1_0455** | | | | | + | | + | | + | | + | | | + | | + | | + | | + | | + | | | + | | + | | + | | + | | + | | | + | | + | | + | | + | | + | | + | | | + | | + | | + | | + | | + | | | + | | + | | + | | + | | + | + | **hypothetical protein** |
| **SSGZ1_0456** | | | | | + | | + | | + | | + | | | + | | + | | + | | + | | + | | | + | | + | | + | | + | | + | | | + | | + | | + | | + | | + | | + | | | + | | + | | + | | + | | + | | | + | | + | | + | | + | | + | + | **hypothetical protein** |
| **SSGZ1_0457** | | | | | + | | + | | + | | + | | | + | | + | | + | | + | | + | | | + | | + | | + | | + | | + | | | + | | + | | + | | + | | + | | + | | | + | | + | | + | | + | | + | | | + | | + | | + | | + | | + | + | **hypothetical protein** |
| **SSGZ1_0458** | | | | | + | | + | | + | | + | | | + | | + | | + | | + | | + | | | + | | + | | + | | + | | + | | | + | | + | | + | | + | | + | | + | | | + | | + | | + | | - | | + | | | - | | + | | + | | + | | + | + | **biotin synthase (BioY family protein), putative** |
| **SSGZ1_0459** | | | | | + | | + | | + | | + | | | + | | + | | + | | + | | + | | | + | | + | | + | | + | | + | | | + | | + | | + | | + | | + | | + | | | + | | + | | + | | + | | + | | | + | | + | | + | | + | | + | + | **hypothetical protein** |
| **SSGZ1_0460** | | | | | + | | + | | + | | + | | | + | | + | | + | | + | | + | | | + | | + | | + | | + | | + | | | + | | + | | + | | + | | + | | + | | | + | | + | | + | | + | | + | | | + | | + | | + | | + | | + | + | **glutathione reductase** |
| **SSGZ1_0461** | | | | | + | | + | | + | | + | | | + | | + | | + | | + | | + | | | + | | + | | + | | + | | + | | | + | | + | | + | | + | | + | | + | | | + | | + | | + | | + | | + | | | + | | + | | + | | + | | + | + | **Membrane-fusion protein** |
| **SSGZ1_0462** | | | | | + | | + | | + | | + | | | + | | + | | + | | + | | + | | | + | | + | | + | | + | | + | | | + | | + | | + | | + | | + | | + | | | + | | + | | + | | + | | + | | | + | | + | | + | | + | | + | + | **ABC transporter** |
| **SSGZ1_0463** | | | | | + | | + | | + | | + | | | + | | + | | + | | + | | + | | | + | | + | | + | | + | | + | | | + | | + | | + | | + | | + | | + | | | + | | + | | + | | + | | + | | | + | | + | | + | | + | | + | + | **Protein of unknown function DUF214** |
| **SSGZ1_0464** | | | | | + | | + | | + | | + | | | + | | + | | + | | + | | + | | | + | | + | | + | | + | | + | | | + | | + | | + | | + | | + | | + | | | + | | + | | + | | + | | + | | | + | | + | | + | | + | | + | + | **luciferase** |
| **SSGZ1_0465** | | | | | - | | - | | - | | - | | | - | | - | | - | | - | | - | | | - | | - | | - | | - | | - | | | - | | - | | - | | - | | - | | - | | | - | | - | | - | | - | | - | | | - | | - | | - | | - | | - | - | **putative transposon integrase, Tn916 ORF3-like** |
| **SSGZ1_0466** | | | | | - | | - | | - | | - | | | - | | - | | - | | - | | - | | | - | | - | | - | | - | | - | | | - | | + | | - | | - | | - | | - | | | - | | - | | - | | - | | - | | | + | | + | | - | | - | | - | - | **hypothetical protein** |
| **SSGZ1_0467** | | | | | - | | - | | - | | - | | | - | | - | | - | | - | | - | | | - | | - | | - | | - | | - | | | - | | + | | - | | - | | - | | + | | | - | | - | | - | | - | | - | | | + | | + | | - | | - | | - | - | **excisionase** |
| **SSGZ1_0468** | | | | | - | | - | | - | | - | | | - | | - | | - | | - | | - | | | - | | - | | - | | - | | - | | | - | | - | | - | | - | | - | | - | | | - | | - | | - | | - | | - | | | - | | - | | - | | - | | - | - | **PemK-like protein** |
| **SSGZ1_0469** | | | | | - | | - | | - | | - | | | - | | - | | - | | - | | - | | | - | | - | | - | | - | | - | | | - | | - | | - | | - | | - | | - | | | - | | - | | - | | - | | - | | | - | | - | | - | | - | | - | - | **Chromatin SPT2** |
| **SSGZ1_0470** | | | | | - | | - | | - | | - | | | - | | - | | - | | - | | - | | | - | | - | | - | | - | | - | | | - | | - | | - | | - | | - | | - | | | - | | - | | - | | - | | - | | | - | | + | | - | | - | | - | - | **hypothetical protein** |
| **SSGZ1_0471** | | | | | - | | - | | - | | - | | | - | | - | | - | | - | | - | | | - | | - | | - | | - | | - | | | - | | - | | - | | - | | - | | + | | | - | | - | | - | | - | | - | | | - | | + | | - | | - | | - | - | **hypothetical protein** |
| **SSGZ1_0472** | | | | | - | | - | | - | | - | | | - | | - | | - | | - | | - | | | - | | - | | - | | - | | - | | | - | | - | | - | | - | | - | | - | | | - | | - | | - | | - | | - | | | - | | - | | - | | - | | - | - | **DNA-directed DNA polymerase** |
| **SSGZ1_0473** | | | | | - | | - | | - | | - | | | - | | - | | - | | - | | - | | | - | | - | | - | | - | | - | | | - | | - | | - | | - | | - | | - | | | - | | - | | - | | - | | - | | | - | | - | | - | | - | | - | - | **Transcriptional regulator, Cro/CI family** |
| **SSGZ1_0474** | | | | | - | | - | | - | | - | | | - | | - | | - | | - | | - | | | - | | - | | - | | - | | - | | | - | | - | | - | | - | | - | | - | | | - | | - | | - | | - | | - | | | - | | + | | - | | - | | - | - | **protein of unknown function DUF955** |
| **SSGZ1_0475** | | | | | - | | - | | - | | - | | | - | | - | | - | | - | | - | | | - | | - | | - | | - | | - | | | - | | - | | - | | - | | - | | - | | | - | | - | | - | | - | | - | | | - | | - | | - | | - | | - | - | **Transcriptional regulator, MarR family** |
| **SSGZ1_0476** | | | | | - | | - | | - | | - | | | - | | - | | - | | - | | - | | | - | | - | | - | | - | | - | | | - | | - | | - | | - | | - | | - | | | - | | - | | - | | - | | - | | | - | | - | | - | | - | | - | - | **TetW** |
| **SSGZ1_0477** | | | | | - | | - | | - | | - | | | - | | - | | - | | - | | - | | | - | | - | | - | | - | | - | | | - | | - | | - | | - | | - | | - | | | - | | - | | - | | - | | - | | | - | | + | | - | | - | | - | - | **Helix-turn-helix type 3** |
| **SSGZ1_0478** | | | | | - | | - | | - | | - | | | - | | - | | - | | - | | - | | | - | | - | | - | | - | | - | | | - | | - | | - | | - | | - | | - | | | - | | - | | - | | - | | - | | | - | | + | | - | | - | | - | - | **hypothetical protein** |
| **SSGZ1_0479** | | | | | - | | - | | - | | - | | | - | | - | | - | | - | | - | | | - | | - | | - | | - | | - | | | - | | - | | - | | - | | - | | - | | | - | | - | | - | | - | | - | | | - | | - | | - | | - | | - | - | **antigen-like protein** |
| **SSGZ1_0480** | | | | | - | | - | | - | | - | | | - | | - | | - | | - | | - | | | - | | - | | - | | - | | - | | | - | | - | | - | | - | | - | | - | | | - | | - | | - | | - | | - | | | - | | - | | - | | - | | - | - | **Sortase B family protein** |
| **SSGZ1_0481** | | | | | - | | - | | - | | - | | | - | | - | | - | | - | | - | | | - | | - | | - | | - | | - | | | - | | - | | - | | - | | - | | - | | | - | | - | | - | | - | | - | | | - | | - | | - | | - | | - | - | **TrsE-like protein** |
| **SSGZ1_0482** | | | | | - | | - | | - | | - | | | - | | - | | - | | - | | - | | | - | | - | | - | | - | | - | | | - | | - | | - | | - | | - | | - | | | - | | - | | - | | - | | - | | | - | | - | | - | | - | | - | - | **hypothetical protein** |
| **SSGZ1_0483** | | | | | - | | - | | - | | - | | | - | | - | | - | | - | | - | | | - | | - | | - | | - | | - | | | - | | - | | - | | - | | - | | - | | | - | | - | | - | | - | | - | | | - | | + | | - | | - | | - | - | **hypothetical protein** |
| **SSGZ1_0484** | | | | | - | | - | | - | | - | | | - | | - | | - | | - | | - | | | - | | - | | - | | - | | - | | | - | | - | | - | | - | | - | | - | | | - | | - | | - | | - | | - | | | - | | + | | - | | - | | - | - | **hypothetical protein** |
| **SSGZ1_0485** | | | | | - | | - | | - | | - | | | - | | - | | - | | - | | - | | | - | | - | | - | | - | | - | | | - | | - | | - | | - | | - | | - | | | - | | - | | - | | - | | - | | | - | | - | | - | | - | | - | - | **hypothetical protein** |
| **SSGZ1_0486** | | | | | - | | - | | - | | - | | | - | | - | | - | | - | | - | | | - | | - | | - | | - | | - | | | - | | - | | - | | - | | - | | - | | | - | | - | | - | | - | | - | | | - | | + | | - | | - | | - | - | **VirB2-like protein** |
| **SSGZ1_0487** | | | | | - | | - | | - | | - | | | - | | - | | - | | - | | - | | | - | | - | | - | | - | | - | | | - | | - | | - | | - | | - | | - | | | - | | - | | - | | - | | - | | | - | | - | | - | | - | | - | - | **TraG/TraD family** |
| **SSGZ1_0488** | | | | | - | | - | | - | | - | | | - | | - | | - | | - | | - | | | - | | - | | - | | - | | - | | | - | | + | | - | | - | | - | | - | | | - | | - | | - | | - | | - | | | + | | + | | - | | - | | - | - | **hypothetical protein** |
| **SSGZ1_0489** | | | | | - | | - | | - | | - | | | - | | - | | - | | - | | - | | | - | | - | | - | | - | | - | | | - | | - | | - | | - | | - | | - | | | - | | - | | - | | - | | - | | | - | | + | | - | | - | | - | - | **LtrC-like protein** |
| **SSGZ1_0490** | | | | | - | | - | | - | | - | | | - | | - | | - | | - | | - | | | - | | - | | - | | - | | - | | | - | | - | | - | | - | | - | | - | | | - | | - | | - | | - | | - | | | - | | - | | - | | - | | - | - | **hypothetical protein** |
| **SSGZ1_0491** | | | | | - | | - | | - | | - | | | - | | - | | - | | - | | - | | | - | | - | | - | | - | | - | | | - | | - | | - | | - | | - | | - | | | - | | - | | - | | - | | - | | | - | | - | | - | | - | | - | - | **Rlx-like protein** |
| **SSGZ1_0492** | | | | | - | | - | | - | | - | | | - | | - | | - | | - | | - | | | - | | - | | - | | - | | - | | | - | | - | | - | | - | | - | | - | | | - | | - | | - | | - | | - | | | - | | - | | - | | - | | - | - | **MocB-like protein** |
| **SSGZ1_0493** | | | | | - | | - | | - | | - | | | - | | - | | - | | - | | - | | | - | | - | | - | | - | | - | | | - | | - | | - | | - | | - | | - | | | - | | - | | - | | - | | - | | | - | | - | | - | | - | | - | - | **hypothetical protein** |
| **SSGZ1_0494** | | | | | - | | - | | - | | - | | | - | | - | | - | | - | | - | | | - | | - | | - | | - | | - | | | - | | - | | - | | - | | - | | - | | | - | | - | | - | | - | | - | | | - | | - | | - | | - | | - | - | **LtrC** |
| **SSGZ1_0495** | | | | | - | | - | | - | | - | | | - | | - | | - | | - | | - | | | - | | - | | - | | - | | - | | | - | | - | | - | | - | | - | | - | | | - | | - | | - | | - | | - | | | - | | - | | - | | - | | - | - | **hypothetical protein** |
| **SSGZ1_0496** | | | | | - | | - | | - | | - | | | - | | - | | - | | - | | - | | | - | | - | | - | | - | | - | | | - | | - | | - | | - | | - | | - | | | - | | - | | - | | - | | - | | | - | | - | | - | | - | | - | - | **putative helicase** |
| **SSGZ1_0497** | | | | | - | | - | | - | | - | | | - | | - | | - | | - | | - | | | - | | - | | - | | - | | - | | | - | | - | | - | | - | | - | | - | | | - | | - | | - | | - | | - | | | - | | - | | - | | - | | - | - | **hypothetical protein** |
| **SSGZ1_0498** | | | | | - | | - | | - | | - | | | - | | - | | - | | - | | - | | | - | | - | | - | | - | | - | | | - | | - | | - | | - | | - | | - | | | - | | - | | - | | - | | - | | | - | | - | | - | | - | | - | - | **hypothetical protein** |
| **SSGZ1_0499** | | | | | - | | - | | - | | - | | | - | | - | | - | | - | | - | | | - | | - | | - | | - | | - | | | - | | - | | - | | - | | - | | - | | | - | | - | | - | | - | | - | | | - | | + | | - | | - | | - | - | **hypothetical protein** |
| **SSGZ1_0500** | | | | | - | | - | | - | | - | | | - | | - | | - | | - | | - | | | - | | - | | - | | - | | - | | | - | | - | | - | | - | | - | | - | | | - | | - | | - | | - | | - | | | - | | - | | - | | - | | - | - | **hypothetical protein** |
| **SSGZ1_0501** | | | | | - | | - | | - | | - | | | - | | - | | - | | - | | - | | | - | | - | | - | | - | | - | | | - | | - | | - | | - | | - | | - | | | - | | - | | - | | - | | - | | | - | | - | | - | | - | | - | - | **Collagen adhesion protein** |
| **SSGZ1_0502** | | | | | - | | - | | - | | - | | | - | | - | | - | | - | | - | | | - | | - | | - | | - | | - | | | - | | - | | - | | - | | - | | - | | | - | | - | | - | | - | | - | | | - | | - | | - | | - | | - | - | **Signal recognition particle-docking protein** |
| **SSGZ1_0503** | | | | | - | | - | | - | | - | | | - | | - | | - | | - | | - | | | - | | - | | - | | - | | - | | | - | | - | | - | | - | | - | | - | | | - | | - | | - | | - | | - | | | - | | - | | - | | - | | - | - | **Chromosome partitioning protein parB** |
| **SSGZ1_0504** | | | | | - | | - | | - | | - | | | - | | - | | - | | - | | - | | | - | | - | | - | | - | | - | | | - | | - | | - | | - | | - | | - | | | - | | - | | - | | - | | - | | | - | | - | | - | | - | | - | - | **Chromosome partitioning protein parA** |
| **SSGZ1_0505** | | | | | - | | - | | - | | - | | | - | | - | | - | | - | | - | | | - | | - | | - | | + | | - | | | - | | - | | - | | - | | - | | + | | | - | | - | | - | | - | | - | | | + | | + | | - | | - | | - | - | **hypothetical protein** |
| **SSGZ1_0506** | | | | | - | | - | | - | | - | | | - | | - | | - | | - | | - | | | - | | - | | - | | - | | - | | | - | | + | | - | | - | | - | | - | | | - | | - | | - | | - | | - | | | + | | + | | - | | - | | - | - | **DNA-binding protein** |
| **SSGZ1_0507** | | | | | - | | - | | - | | - | | | - | | - | | - | | - | | - | | | - | | - | | - | | - | | - | | | - | | - | | - | | - | | - | | - | | | - | | - | | - | | - | | - | | | - | | + | | - | | - | | - | - | **ORFG2-1** |
| **SSGZ1_0508** | | | | | - | | - | | - | | - | | | - | | - | | - | | - | | - | | | - | | - | | - | | - | | - | | | - | | - | | - | | - | | - | | - | | | - | | - | | - | | - | | - | | | - | | - | | - | | - | | - | - | **conserved domain protein** |
| **SSGZ1_0509** | | | | | + | | + | | + | | + | | | + | | + | | + | | + | | + | | | + | | + | | + | | + | | + | | | + | | + | | + | | + | | + | | + | | | + | | + | | + | | + | | + | | | + | | + | | + | | + | | + | + | **Lysyl-tRNA synthetase, class-2** |
| **SSGZ1_0510** | | | | | + | | + | | + | | + | | | + | | + | | + | | + | | + | | | + | | + | | + | | + | | + | | | + | | + | | + | | + | | + | | + | | | + | | + | | + | | + | | + | | | + | | + | | + | | + | | + | + | **bisphosphoglycerate mutase** |
| **SSGZ1_0511** | | | | | + | | + | | + | | + | | | + | | + | | + | | + | | + | | | + | | + | | + | | + | | + | | | + | | + | | + | | + | | + | | + | | | + | | + | | + | | + | | + | | | + | | + | | + | | + | | + | + | **YbaK** |
| **SSGZ1_0512** | | | | | + | | + | | + | | + | | | + | | + | | + | | + | | + | | | + | | + | | + | | + | | + | | | + | | + | | + | | + | | + | | + | | | + | | + | | + | | + | | + | | | + | | + | | + | | + | | + | + | **Predicted membrane protein** |
| **SSGZ1_0513** | | | | | + | | + | | + | | + | | | + | | + | | + | | + | | + | | | + | | + | | + | | + | | + | | | + | | + | | + | | + | | + | | + | | | + | | + | | + | | + | | + | | | + | | + | | + | | + | | + | + | **Predicted membrane protein** |
| **SSGZ1_0514** | | | | | + | | + | | + | | + | | | + | | + | | + | | + | | + | | | + | | + | | + | | + | | + | | | + | | + | | + | | + | | + | | + | | | + | | + | | + | | + | | + | | | + | | + | | + | | + | | + | + | **Glycoside hydrolase, family 25** |
| **SSGZ1_0515** | | | | | + | | + | | + | | + | | | + | | + | | + | | + | | + | | | + | | + | | + | | + | | + | | | + | | + | | + | | + | | + | | + | | | + | | + | | + | | + | | + | | | + | | + | | + | | + | | + | + | **Preprotein translocase subunit YidC** |
| **SSGZ1_0516** | | | | | + | | + | | + | | + | | | + | | + | | + | | + | | + | | | + | | + | | + | | + | | + | | | + | | + | | + | | + | | + | | + | | | + | | + | | + | | + | | + | | | + | | + | | + | | + | | + | + | **hypothetical protein** |
| **SSGZ1_0517** | | | | | + | | + | | + | | + | | | + | | + | | + | | + | | + | | | + | | + | | + | | + | | + | | | + | | + | | + | | + | | + | | + | | | + | | + | | + | | + | | + | | | + | | + | | + | | + | | + | + | **Cell cycle protein** |
| **SSGZ1_0518** | | | | | + | | + | | + | | + | | | + | | + | | + | | + | | + | | | + | | + | | + | | + | | + | | | + | | + | | + | | + | | + | | + | | | + | | + | | + | | + | | + | | | + | | + | | + | | + | | + | + | **Phosphoenolpyruvate carboxylase** |
| **SSGZ1_0519** | | | | | + | | + | | + | | + | | | + | | + | | + | | + | | + | | | + | | + | | + | | + | | + | | | + | | + | | + | | + | | + | | + | | | + | | + | | + | | + | | + | | | + | | + | | + | | + | | + | + | **DNA-directed RNA polymerase specialized sigma** |
| **SSGZ1_0520** | | | | | + | | + | | + | | + | | | + | | + | | + | | + | | + | | | + | | + | | - | | - | | - | | | + | | + | | - | | - | | + | | + | | | + | | + | | + | | - | | - | | | + | | + | | + | | + | | + | + | **hypothetical protein** |
| **SSGZ1_0521** | | | | | + | | + | | + | | + | | | + | | + | | + | | + | | + | | | + | | + | | + | | + | | + | | | + | | + | | + | | + | | + | | + | | | + | | + | | + | | + | | + | | | + | | + | | + | | + | | + | + | **Protein Translation Elongation Factor Tu** |
| **SSGZ1_0522** | | | | | + | | + | | + | | + | | | + | | + | | + | | + | | + | | | + | | + | | + | | + | | + | | | + | | + | | + | | + | | + | | + | | | + | | + | | + | | + | | + | | | + | | + | | + | | + | | + | + | **triosephosphate isomerase** |
| **SSGZ1_0523** | | | | | + | | - | | + | | - | | | - | | - | | - | | - | | - | | | + | | + | | + | | + | | + | | | + | | - | | + | | - | | - | | + | | | - | | - | | + | | + | | + | | | + | | + | | + | | + | | - | - | **Transposase, IS204/IS1001/IS1096/IS1165** |
| **SSGZ1_0524** | | | | | - | | + | | + | | + | | | + | | + | | + | | + | | + | | | + | | + | | + | | + | | + | | | + | | + | | + | | + | | + | | + | | | + | | + | | + | | + | | + | | | + | | + | | + | | + | | + | + | **hydrolase, haloacid dehalogenase-like family** |
| **SSGZ1_0525** | | | | | + | | + | | + | | + | | | + | | + | | + | | + | | + | | | + | | + | | + | | + | | + | | | + | | + | | + | | + | | + | | + | | | + | | + | | + | | + | | + | | | + | | + | | + | | + | | + | + | **Metal-dependent phosphohydrolase** |
| **SSGZ1_0526** | | | | | + | | + | | + | | + | | | + | | + | | + | | + | | + | | | + | | + | | + | | + | | + | | | + | | + | | + | | + | | + | | + | | | + | | + | | + | | + | | + | | | + | | + | | + | | + | | + | + | **hypothetical protein** |
| **SSGZ1_0527** | | | | | + | | + | | + | | + | | | + | | + | | + | | + | | + | | | + | | + | | + | | + | | + | | | + | | + | | + | | + | | + | | + | | | + | | + | | + | | + | | + | | | + | | + | | + | | + | | + | + | **hypothetical protein** |
| **SSGZ1_0528** | | | | | - | | + | | - | | + | | | + | | + | | + | | + | | + | | | - | | - | | - | | - | | - | | | + | | - | | - | | + | | + | | + | | | + | | + | | - | | + | | + | | | + | | + | | + | | - | | + | - | **transposase, IS30 family** |
| **SSGZ1_0529** | | | | | + | | + | | + | | + | | | + | | + | | + | | + | | + | | | + | | + | | + | | + | | + | | | + | | + | | + | | + | | + | | + | | | + | | + | | + | | + | | + | | | + | | + | | + | | + | | + | + | **Adenosine deaminase** |
| **SSGZ1_0530** | | | | | + | | + | | + | | + | | | + | | + | | + | | + | | + | | | + | | + | | + | | + | | + | | | + | | + | | + | | + | | + | | + | | | + | | + | | + | | + | | + | | | + | | + | | + | | + | | + | + | **Predicted membrane protein** |
| **SSGZ1_0531** | | | | | + | | + | | + | | + | | | + | | + | | + | | + | | + | | | + | | + | | + | | + | | + | | | + | | + | | + | | + | | + | | + | | | + | | + | | + | | + | | + | | | + | | + | | + | | + | | + | + | **ABC-type multidrug transport system, ATPase** |
| **SSGZ1_0532** | | | | | + | | + | | + | | + | | | + | | + | | + | | + | | + | | | + | | + | | + | | + | | + | | | + | | + | | + | | + | | + | | + | | | + | | + | | + | | + | | + | | | + | | + | | + | | + | | + | + | **regulatory protein, GntR** |
| **SSGZ1_0533** | | | | | + | | + | | + | | + | | | + | | + | | + | | + | | + | | | + | | + | | + | | + | | + | | | + | | + | | + | | + | | + | | + | | | + | | + | | + | | + | | + | | | + | | + | | + | | + | | + | + | **DNA polymerase III alpha subunit** |
| **SSGZ1_0534** | | | | | + | | + | | + | | + | | | + | | + | | + | | + | | + | | | + | | + | | + | | + | | + | | | + | | + | | + | | + | | + | | + | | | + | | + | | + | | + | | + | | | + | | + | | + | | + | | + | + | **6-phosphofructokinase** |
| **SSGZ1_0535** | | | | | + | | + | | + | | + | | | + | | + | | + | | + | | + | | | + | | + | | + | | + | | + | | | + | | + | | + | | + | | + | | + | | | + | | + | | + | | + | | + | | | + | | + | | + | | + | | + | + | **Pyruvate kinase** |
| **SSGZ1_0536** | | | | | + | | + | | + | | + | | | + | | + | | + | | + | | + | | | + | | + | | + | | + | | + | | | + | | - | | + | | + | | + | | - | | | + | | + | | + | | - | | + | | | - | | - | | + | | + | | + | + | **YSIRK Gram-positive signal peptide** |
| **SSGZ1_0537** | | | | | + | | + | | + | | + | | | + | | + | | + | | + | | + | | | + | | + | | + | | + | | + | | | + | | + | | + | | + | | + | | + | | | + | | + | | + | | - | | + | | | + | | + | | + | | + | | + | + | **FAD dependent oxidoreductase** |
| **SSGZ1_0538** | | | | | + | | + | | + | | + | | | + | | + | | + | | + | | + | | | + | | + | | + | | + | | + | | | + | | + | | + | | + | | + | | + | | | + | | + | | + | | - | | + | | | + | | + | | + | | + | | + | + | **Lipolytic enzyme, G-D-S-L** |
| **SSGZ1_0539** | | | | | + | | + | | + | | + | | | + | | + | | + | | + | | + | | | + | | + | | + | | + | | + | | | + | | + | | + | | + | | + | | + | | | + | | + | | + | | - | | + | | | + | | + | | + | | + | | + | + | **Signal peptidase I** |
| **SSGZ1_0540** | | | | | + | | + | | + | | + | | | + | | + | | + | | + | | + | | | + | | + | | + | | + | | + | | | + | | + | | + | | + | | + | | + | | | + | | + | | + | | + | | + | | | + | | + | | + | | + | | + | + | **glucosamine--fructose-6-phosphate** |
| **SSGZ1_0541** | | | | | + | | + | | + | | + | | | + | | + | | + | | + | | + | | | + | | + | | + | | + | | + | | | + | | + | | + | | + | | + | | + | | | + | | + | | + | | + | | + | | | + | | + | | + | | + | | + | + | **amino acid (glutamine) ABC transporter, permease** |
| **SSGZ1_0542** | | | | | + | | + | | + | | + | | | + | | + | | + | | + | | + | | | + | | + | | + | | + | | + | | | + | | + | | + | | + | | + | | + | | | + | | + | | + | | + | | + | | | + | | + | | + | | + | | + | + | **ABC transporter ATP-binding protein** |
| **SSGZ1_0543** | | | | | + | | + | | + | | + | | | + | | + | | + | | + | | + | | | + | | + | | + | | + | | + | | | + | | + | | + | | + | | + | | + | | | + | | + | | + | | + | | + | | | + | | + | | + | | + | | + | + | **ABC transporter substrate-binding protein** |
| **SSGZ1_0546** | | | | | + | | + | | + | | + | | | + | | + | | + | | + | | + | | | + | | + | | + | | + | | + | | | + | | + | | + | | + | | + | | + | | | + | | + | | + | | + | | + | | | + | | + | | + | | + | | + | + | **Beta-lactamase-like protein** |
| **SSGZ1_0547** | | | | | + | | + | | + | | + | | | + | | + | | + | | + | | + | | | + | | + | | + | | + | | + | | | + | | + | | + | | + | | + | | + | | | + | | + | | + | | + | | + | | | + | | + | | + | | + | | + | + | **DnaQ exonuclease** |
| **SSGZ1_0548** | | | | | + | | + | | + | | + | | | + | | + | | + | | + | | + | | | + | | + | | + | | + | | + | | | + | | + | | + | | + | | + | | + | | | + | | + | | + | | + | | + | | | + | | + | | + | | + | | + | + | **Glutamate 5-kinase** |
| **SSGZ1_0549** | | | | | + | | + | | + | | + | | | + | | + | | + | | + | | + | | | + | | + | | + | | + | | + | | | + | | + | | + | | + | | + | | + | | | + | | + | | + | | + | | + | | | + | | + | | + | | + | | + | + | **amma-glutamyl phosphate reductase GPR** |
| **SSGZ1_0550** | | | | | + | | + | | + | | + | | | + | | + | | + | | + | | + | | | + | | + | | + | | + | | + | | | + | | + | | + | | + | | + | | + | | | + | | + | | + | | + | | + | | | + | | + | | + | | + | | + | + | **Delta 1-pyrroline-5-carboxylate reductase** |
| **SSGZ1_0551** | | | | | + | | + | | + | | + | | | + | | + | | + | | + | | + | | | + | | + | | + | | + | | + | | | + | | + | | + | | + | | + | | + | | | + | | + | | + | | + | | + | | | + | | + | | + | | + | | + | + | **Transcriptional regulator** |
| **SSGZ1_0552** | | | | | + | | + | | + | | + | | | + | | + | | + | | + | | + | | | + | | + | | + | | + | | + | | | + | | + | | + | | + | | + | | + | | | + | | + | | + | | + | | + | | | + | | + | | + | | + | | + | + | **DegV** |
| **SSGZ1_0553** | | | | | + | | + | | + | | + | | | + | | + | | + | | + | | + | | | + | | + | | + | | + | | + | | | + | | + | | + | | + | | + | | + | | | + | | + | | + | | + | | + | | | + | | + | | + | | + | | + | + | **putative transcriptional** |
| **SSGZ1_0554** | | | | | + | | + | | + | | + | | | + | | + | | + | | + | | + | | | + | | + | | + | | + | | + | | | + | | + | | + | | + | | + | | + | | | + | | + | | + | | + | | + | | | + | | + | | + | | + | | + | + | **hypothetical protein** |
| **SSGZ1_0555** | | | | | + | | + | | + | | + | | | + | | + | | + | | + | | + | | | + | | + | | + | | + | | + | | | + | | + | | + | | + | | + | | + | | | + | | - | | + | | + | | + | | | + | | + | | + | | + | | + | + | **Cps2A** |
| **SSGZ1_0556** | | | | | + | | + | | + | | + | | | + | | + | | + | | + | | + | | | + | | + | | + | | + | | + | | | + | | + | | + | | + | | + | | + | | | + | | - | | + | | + | | + | | | + | | + | | + | | + | | + | + | **Cps2B** |
| **SSGZ1_0557** | | | | | + | | + | | + | | + | | | + | | + | | + | | + | | + | | | + | | + | | + | | + | | + | | | + | | + | | + | | + | | + | | + | | | + | | - | | + | | + | | + | | | + | | + | | + | | + | | + | + | **Cps2C** |
| **SSGZ1_0558** | | | | | + | | + | | + | | + | | | + | | + | | + | | + | | + | | | + | | + | | + | | + | | + | | | + | | + | | + | | + | | + | | + | | | + | | - | | + | | + | | + | | | + | | + | | + | | + | | + | + | **Cps2D** |
| **SSGZ1_0559** | | | | | + | | + | | + | | + | | | + | | + | | + | | + | | + | | | + | | - | | - | | - | | - | | | - | | + | | - | | - | | - | | - | | | + | | - | | + | | - | | - | | | - | | + | | - | | + | | - | - | **Cps2E** |
| **SSGZ1_0560** | | | | | + | | + | | + | | + | | | + | | + | | + | | + | | + | | | + | | - | | - | | - | | + | | | - | | + | | - | | - | | - | | - | | | - | | - | | - | | - | | - | | | + | | + | | - | | - | | - | - | **Cps2F** |
| **SSGZ1_0561** | | | | | + | | + | | + | | + | | | + | | + | | + | | + | | + | | | + | | - | | - | | - | | - | | | - | | - | | - | | - | | - | | - | | | - | | - | | - | | - | | - | | | - | | + | | - | | - | | - | - | **Cps2G** |
| **SSGZ1_0562** | | | | | + | | + | | + | | + | | | + | | + | | + | | + | | + | | | + | | - | | - | | - | | - | | | - | | + | | - | | - | | - | | - | | | - | | - | | - | | - | | - | | | + | | + | | - | | - | | - | - | **Cps2H** |
| **SSGZ1_0563** | | | | | + | | + | | + | | + | | | + | | + | | + | | + | | + | | | + | | - | | - | | - | | - | | | + | | + | | - | | - | | - | | + | | | - | | - | | + | | - | | - | | | + | | + | | - | | - | | - | - | **Cps2I** |
| **SSGZ1_0564** | | | | | + | | + | | + | | + | | | + | | + | | + | | + | | + | | | + | | - | | - | | - | | - | | | - | | + | | - | | - | | - | | + | | | - | | - | | - | | - | | - | | | + | | + | | - | | - | | - | - | **Cps2J** |
| **SSGZ1_0565** | | | | | + | | + | | + | | + | | | + | | + | | + | | + | | + | | | + | | - | | - | | - | | - | | | - | | - | | - | | - | | - | | - | | | + | | - | | + | | - | | - | | | - | | + | | - | | - | | - | - | **Cps2K** |
| **SSGZ1_0566** | | | | | + | | + | | + | | + | | | + | | + | | + | | + | | + | | | + | | - | | - | | - | | - | | | - | | + | | - | | - | | - | | - | | | + | | - | | - | | - | | - | | | - | | + | | - | | - | | - | - | **hypothetical protein** |
| **SSGZ1_0567** | | | | | + | | + | | + | | + | | | + | | + | | + | | + | | + | | | + | | - | | - | | - | | - | | | - | | + | | - | | - | | - | | - | | | + | | - | | - | | - | | - | | | - | | + | | - | | - | | - | - | **hypothetical protein** |
| **SSGZ1_0569** | | | | | + | | + | | + | | + | | | + | | + | | + | | + | | + | | | + | | - | | - | | - | | - | | | - | | - | | - | | - | | - | | - | | | + | | - | | - | | - | | - | | | - | | + | | - | | - | | - | - | **Cps2L** |
| **SSGZ1_0570** | | | | | + | | + | | + | | + | | | + | | + | | + | | + | | + | | | + | | - | | - | | - | | - | | | - | | - | | - | | - | | - | | - | | | + | | - | | - | | - | | - | | | - | | - | | - | | - | | - | - | **polysaccharide biosynthesis protein CpsL** |
| **SSGZ1_0571** | | | | | + | | + | | + | | + | | | + | | + | | + | | + | | + | | | + | | - | | - | | - | | + | | | - | | + | | - | | - | | - | | - | | | + | | - | | + | | - | | - | | | - | | + | | - | | - | | - | - | **N-acylneuraminate-9-phosphate synthase** |
| **SSGZ1_0572** | | | | | + | | + | | + | | + | | | + | | + | | + | | + | | + | | | + | | - | | - | | - | | - | | | - | | - | | - | | - | | - | | - | | | + | | - | | + | | - | | - | | | - | | - | | - | | - | | - | - | **UDP-N-acetylglucosamine 2-epimerase** |
| **SSGZ1_0573** | | | | | + | | + | | + | | + | | | + | | + | | + | | + | | + | | | + | | - | | - | | - | | + | | | - | | - | | - | | - | | - | | - | | | + | | - | | + | | - | | - | | | + | | + | | - | | - | | - | - | **transferase hexapeptide repeat** |
| **SSGZ1_0574** | | | | | + | | + | | + | | + | | | + | | + | | + | | + | | + | | | + | | - | | - | | - | | - | | | - | | - | | - | | - | | - | | - | | | + | | - | | - | | - | | - | | | - | | - | | - | | - | | - | - | **Acylneuraminate cytidylyltransferase** |
| **SSGZ1_0575** | | | | | + | | + | | + | | + | | | + | | + | | + | | + | | + | | | + | | - | | - | | - | | - | | | - | | - | | - | | - | | - | | + | | | + | | - | | - | | - | | - | | | - | | - | | - | | - | | - | - | **Transposase and inactivated derivative, IS5** |
| **SSGZ1_0576** | | | | | + | | + | | + | | + | | | + | | + | | + | | + | | + | | | + | | - | | - | | - | | - | | | - | | - | | - | | - | | - | | + | | | + | | - | | - | | - | | - | | | - | | + | | - | | - | | - | - | **Transposase, IS4** |
| **SSGZ1_0577** | | | | | + | | + | | + | | + | | | + | | + | | + | | + | | + | | | + | | - | | - | | - | | - | | | - | | - | | - | | - | | - | | + | | | + | | - | | - | | - | | - | | | - | | + | | - | | - | | - | - | **hypothetical protein** |
| **SSGZ1_0578** | | | | | + | | + | | + | | + | | | + | | + | | + | | + | | + | | | + | | + | | + | | + | | + | | | + | | + | | + | | + | | + | | + | | | + | | + | | + | | + | | + | | | + | | + | | + | | + | | + | + | **Transposase, Synechocystis PCC 6803** |
| **SSGZ1_0579** | | | | | + | | + | | + | | + | | | + | | + | | + | | + | | + | | | + | | + | | + | | + | | - | | | - | | + | | - | | - | | - | | + | | | + | | + | | + | | + | | + | | | + | | + | | + | | + | | + | + | **similar to Transposase and inactivated** |
| **SSGZ1_0580** | | | | | + | | + | | + | | + | | | + | | + | | + | | + | | + | | | + | | + | | + | | + | | - | | | + | | + | | + | | + | | + | | - | | | + | | + | | - | | + | | + | | | + | | + | | + | | - | | + | + | **IS66 family element, Orf1** |
| **SSGZ1_0581** | | | | | + | | + | | + | | + | | | + | | + | | + | | + | | + | | | + | | + | | + | | + | | - | | | + | | + | | + | | + | | + | | - | | | + | | - | | - | | + | | + | | | + | | + | | + | | - | | + | + | **IS66 Orf2 like protein** |
| **SSGZ1_0582** | | | | | + | | + | | + | | + | | | + | | + | | + | | + | | + | | | + | | + | | + | | + | | - | | | - | | - | | + | | + | | + | | - | | | + | | - | | - | | + | | + | | | + | | + | | + | | - | | - | - | **hypothetical protein** |
| **SSGZ1_0583** | | | | | + | | + | | + | | + | | | + | | + | | + | | + | | + | | | + | | + | | + | | + | | - | | | - | | - | | + | | - | | - | | - | | | + | | - | | - | | + | | + | | | + | | + | | + | | - | | - | - | **Transposase IS66** |
| **SSGZ1_0584** | | | | | + | | + | | + | | + | | | + | | + | | + | | + | | + | | | + | | + | | + | | + | | - | | | + | | + | | + | | + | | + | | + | | | + | | - | | + | | + | | + | | | + | | + | | + | | + | | - | - | **Transposase and inactivated derivative** |
| **SSGZ1_0585** | | | | | + | | + | | + | | + | | | + | | + | | + | | + | | + | | | + | | - | | - | | - | | - | | | + | | + | | + | | - | | - | | - | | | + | | - | | - | | + | | + | | | + | | + | | - | | - | | - | + | **IS1239 transposase** |
| **SSGZ1_0586** | | | | | + | | + | | + | | + | | | + | | + | | + | | + | | + | | | + | | + | | + | | + | | - | | | + | | + | | + | | + | | + | | + | | | + | | + | | + | | + | | + | | | + | | + | | + | | - | | + | + | **IS66 family element, Orf1** |
| **SSGZ1_0588** | | | | | + | | + | | + | | + | | | + | | + | | + | | + | | + | | | + | | + | | + | | + | | - | | | + | | + | | - | | + | | + | | + | | | + | | + | | + | | + | | + | | | + | | + | | + | | - | | + | + | **hypothetical protein** |
| **SSGZ1_0590** | | | | | + | | + | | + | | + | | | + | | + | | + | | + | | + | | | + | | + | | - | | + | | + | | | + | | + | | + | | + | | + | | + | | | + | | - | | + | | + | | + | | | + | | + | | - | | + | | + | + | **ATPase involved in DNA repair** |
| **SSGZ1_0591** | | | | | + | | + | | + | | + | | | + | | + | | + | | + | | + | | | + | | + | | + | | + | | + | | | + | | + | | + | | + | | + | | + | | | + | | + | | + | | + | | + | | | + | | + | | + | | + | | + | + | **3-phosphoshikimate 1-carboxyvinyltransferase** |
| **SSGZ1_0592** | | | | | + | | + | | + | | + | | | + | | + | | + | | + | | + | | | + | | + | | + | | + | | + | | | + | | + | | + | | + | | + | | + | | | + | | + | | + | | + | | + | | | + | | + | | + | | + | | + | + | **putative shikimate kinase** |
| **SSGZ1_0593** | | | | | + | | + | | + | | + | | | + | | + | | + | | + | | + | | | + | | + | | + | | + | | + | | | + | | + | | + | | + | | + | | + | | | + | | + | | + | | + | | + | | | + | | + | | + | | + | | + | + | **putative prephenate dehydratase** |
| **SSGZ1_0594** | | | | | + | | + | | + | | + | | | + | | + | | + | | + | | + | | | + | | + | | + | | + | | + | | | + | | + | | + | | + | | + | | + | | | + | | + | | + | | + | | + | | | + | | + | | + | | + | | + | + | **putative prephenate dehydratase** |
| **SSGZ1_0595** | | | | | + | | + | | + | | + | | | + | | + | | + | | + | | + | | | + | | + | | + | | + | | + | | | + | | + | | + | | + | | + | | + | | | + | | + | | + | | + | | + | | | + | | + | | + | | + | | + | + | **Cell envelope-related transcriptional** |
| **SSGZ1_0596** | | | | | + | | + | | + | | + | | | + | | + | | + | | + | | + | | | + | | + | | + | | + | | + | | | + | | + | | + | | + | | + | | + | | | + | | + | | + | | + | | + | | | + | | + | | + | | + | | + | + | **tRNA (uracil-5-)-methyltransferase/TrmA** |
| **SSGZ1_0598** | | | | | + | | + | | + | | + | | | + | | + | | + | | + | | + | | | + | | + | | + | | + | | + | | | + | | + | | + | | + | | + | | + | | | + | | + | | + | | + | | + | | | + | | + | | + | | + | | + | + | **UDP-galactopyranose mutase** |
| **SSGZ1_0599** | | | | | + | | + | | + | | + | | | + | | + | | + | | + | | + | | | + | | + | | + | | + | | + | | | + | | + | | + | | + | | + | | + | | | + | | + | | + | | + | | + | | | + | | + | | + | | + | | + | + | **NADPH-dependent FMN reductase** |
| **SSGZ1_0600** | | | | | + | | + | | + | | + | | | + | | + | | + | | + | | + | | | + | | + | | + | | + | | + | | | + | | + | | + | | + | | + | | + | | | + | | + | | + | | + | | + | | | + | | + | | + | | + | | + | + | **regulatory protein, MarR** |
| **SSGZ1_0601** | | | | | + | | + | | + | | + | | | + | | + | | + | | + | | + | | | + | | + | | + | | + | | + | | | + | | + | | + | | + | | - | | + | | | + | | + | | + | | + | | + | | | + | | + | | + | | + | | + | + | **IS630-Spn1, transposase Orf1** |
| **SSGZ1_0602** | | | | | + | | + | | + | | + | | | + | | + | | + | | + | | + | | | + | | + | | + | | + | | - | | | - | | + | | - | | - | | - | | + | | | + | | + | | + | | + | | + | | | + | | + | | + | | - | | + | - | **IS630-Spn1, transposase Orf2** |
| **SSGZ1_0603** | | | | | + | | + | | + | | + | | | + | | + | | + | | + | | + | | | + | | + | | + | | + | | + | | | + | | + | | + | | + | | + | | + | | | + | | + | | + | | + | | + | | | + | | + | | + | | + | | + | + | **hypothetical protein** |
| **SSGZ1_0604** | | | | | + | | + | | + | | + | | | + | | + | | + | | + | | + | | | + | | + | | + | | + | | + | | | + | | + | | + | | + | | + | | + | | | + | | + | | + | | + | | + | | | + | | + | | + | | + | | + | + | **Aspartate aminotransferase** |
| **SSGZ1_0605** | | | | | + | | + | | + | | + | | | + | | + | | + | | + | | + | | | + | | + | | + | | + | | + | | | + | | + | | + | | + | | + | | + | | | + | | + | | + | | + | | + | | | + | | + | | + | | + | | + | + | **Aspartyl/asparaginyl-tRNA synthetase** |
| **SSGZ1_0606** | | | | | + | | + | | + | | + | | | + | | + | | + | | + | | + | | | + | | + | | + | | + | | + | | | + | | + | | + | | + | | + | | + | | | + | | + | | + | | + | | + | | | + | | + | | + | | + | | + | + | **Asparaginyl-tRNA synthetase** |
| **SSGZ1_0607** | | | | | + | | + | | - | | + | | | + | | + | | + | | + | | + | | | - | | + | | + | | + | | + | | | + | | + | | + | | + | | - | | + | | | + | | + | | + | | + | | + | | | + | | + | | + | | + | | + | + | **hypothetical protein** |
| **SSGZ1_0608** | | | | | + | | + | | - | | + | | | + | | + | | + | | + | | + | | | - | | + | | + | | + | | + | | | + | | + | | + | | - | | - | | + | | | + | | + | | + | | + | | + | | | + | | + | | + | | + | | + | + | **Plasmid stabilization system** |
| **SSGZ1_0609** | | | | | + | | + | | + | | + | | | + | | + | | + | | + | | + | | | + | | + | | + | | + | | + | | | + | | + | | + | | + | | + | | + | | | + | | + | | + | | + | | + | | | + | | + | | + | | + | | + | + | **Multi antimicrobial extrusion protein MatE** |
| **SSGZ1_0610** | | | | | + | | + | | + | | + | | | + | | + | | + | | + | | + | | | + | | + | | + | | + | | + | | | + | | + | | + | | + | | + | | + | | | + | | + | | + | | + | | + | | | + | | + | | + | | + | | + | + | **YjgF-like protein** |
| **SSGZ1_0611** | | | | | + | | + | | + | | + | | | + | | + | | + | | + | | + | | | + | | + | | + | | + | | + | | | + | | + | | + | | + | | + | | + | | | + | | + | | + | | + | | + | | | + | | + | | + | | + | | + | + | **Uncharacterised P-loop ATPase protein** |
| **SSGZ1_0612** | | | | | + | | + | | + | | + | | | + | | + | | + | | + | | + | | | + | | + | | + | | + | | + | | | + | | + | | + | | + | | + | | + | | | + | | + | | + | | + | | + | | | + | | + | | + | | + | | + | + | **hypothetical protein** |
| **SSGZ1_0613** | | | | | + | | + | | + | | + | | | + | | + | | + | | + | | + | | | + | | + | | + | | + | | + | | | + | | + | | + | | + | | + | | + | | | + | | + | | + | | + | | + | | | + | | + | | + | | + | | + | + | **Protein of unknown function DUF199** |
| **SSGZ1_0614** | | | | | + | | + | | + | | + | | | + | | + | | + | | + | | + | | | + | | + | | + | | + | | + | | | + | | + | | + | | + | | + | | + | | | + | | + | | + | | + | | + | | | + | | + | | + | | + | | + | + | **Cyclic nucleotide-binding protein** |
| **SSGZ1_0615** | | | | | + | | + | | + | | + | | | + | | + | | + | | + | | + | | | + | | + | | + | | + | | + | | | + | | + | | + | | + | | + | | + | | | + | | + | | + | | + | | + | | | + | | + | | + | | + | | + | + | **Arginine deiminase** |
| **SSGZ1_0616** | | | | | + | | + | | + | | + | | | + | | + | | + | | + | | + | | | + | | + | | + | | + | | + | | | + | | + | | + | | + | | + | | + | | | + | | + | | + | | + | | + | | | + | | + | | + | | + | | + | + | **GCN5-related N-acetyltransferase** |
| **SSGZ1_0617** | | | | | + | | + | | + | | + | | | + | | + | | + | | + | | + | | | + | | + | | + | | + | | + | | | + | | + | | + | | + | | + | | + | | | + | | + | | + | | + | | + | | | + | | + | | + | | + | | + | + | **ornithine carbamoyltransferase** |
| **SSGZ1_0618** | | | | | + | | + | | + | | + | | | + | | + | | + | | + | | + | | | + | | + | | + | | + | | + | | | + | | + | | + | | + | | + | | + | | | + | | + | | + | | + | | + | | | + | | + | | + | | + | | + | + | **Carbamate kinase** |
| **SSGZ1_0619** | | | | | + | | + | | + | | + | | | + | | + | | + | | + | | + | | | + | | + | | + | | + | | + | | | + | | + | | + | | + | | + | | + | | | + | | + | | + | | + | | + | | | + | | + | | + | | + | | + | + | **C4-dicarboxylate anaerobic carrier** |
| **SSGZ1_0620** | | | | | + | | + | | + | | + | | | + | | + | | + | | + | | + | | | + | | + | | + | | + | | + | | | + | | + | | + | | + | | + | | + | | | + | | + | | + | | + | | + | | | + | | + | | + | | + | | + | + | **ArcT** |
| **SSGZ1_0621** | | | | | + | | + | | + | | + | | | + | | + | | + | | + | | + | | | + | | + | | + | | + | | + | | | + | | + | | + | | + | | + | | - | | | + | | + | | + | | + | | + | | | + | | + | | + | | + | | + | + | **ArcH** |
| **SSGZ1_0622** | | | | | + | | + | | + | | + | | | + | | + | | + | | + | | + | | | + | | + | | + | | + | | + | | | + | | + | | + | | + | | + | | + | | | + | | + | | + | | + | | + | | | + | | + | | + | | + | | + | + | **Arginine regulator** |
| **SSGZ1_0623** | | | | | + | | + | | + | | + | | | + | | + | | + | | + | | + | | | + | | + | | + | | + | | + | | | + | | + | | + | | + | | + | | + | | | + | | + | | + | | + | | + | | | + | | + | | + | | + | | + | + | **Queuosine biosynthesis protein** |
| **SSGZ1_0624** | | | | | + | | + | | + | | + | | | + | | + | | + | | + | | + | | | + | | + | | + | | + | | + | | | + | | + | | + | | + | | + | | + | | | + | | + | | + | | + | | + | | | + | | + | | + | | + | | + | + | **hypothetical protein** |
| **SSGZ1_0625** | | | | | + | | + | | + | | + | | | + | | + | | + | | + | | + | | | + | | + | | + | | + | | + | | | + | | + | | + | | + | | + | | + | | | + | | + | | + | | + | | + | | | + | | + | | + | | + | | + | + | **Glucosamine/galactosamine-6-phosphate isomerase** |
| **SSGZ1_0626** | | | | | + | | + | | + | | + | | | + | | + | | + | | + | | + | | | + | | + | | + | | + | | + | | | + | | - | | + | | + | | + | | + | | | + | | + | | + | | - | | - | | | + | | + | | + | | + | | + | + | **hypothetical protein** |
| **SSGZ1_0627** | | | | | + | | + | | + | | + | | | + | | + | | + | | + | | + | | | + | | + | | + | | + | | + | | | + | | - | | + | | + | | + | | - | | | + | | + | | + | | - | | - | | | + | | - | | + | | + | | + | + | **hypothetical protein** |
| **SSGZ1_0628** | | | | | + | | + | | + | | + | | | + | | + | | + | | + | | + | | | + | | + | | + | | + | | + | | | + | | - | | + | | + | | + | | - | | | + | | + | | + | | - | | - | | | + | | - | | + | | + | | + | + | **hypothetical protein** |
| **SSGZ1_0629** | | | | | + | | + | | + | | + | | | + | | + | | + | | + | | + | | | + | | + | | + | | + | | + | | | + | | - | | + | | + | | + | | + | | | + | | + | | + | | + | | + | | | + | | + | | + | | + | | + | + | **Amino acid adenylation: D-alanine-activating** |
| **SSGZ1_0630** | | | | | + | | + | | + | | + | | | + | | + | | + | | + | | + | | | + | | + | | + | | + | | + | | | + | | - | | + | | + | | + | | + | | | + | | + | | + | | + | | + | | | + | | + | | + | | + | | + | + | **Membrane bound O-acyl transferase, MBOAT** |
| **SSGZ1_0631** | | | | | + | | + | | + | | + | | | + | | + | | + | | + | | + | | | + | | + | | + | | + | | + | | | + | | - | | + | | + | | + | | + | | | + | | + | | + | | + | | + | | | + | | + | | + | | + | | + | + | **D-alanyl carrier protein** |
| **SSGZ1_0632** | | | | | + | | + | | + | | + | | | + | | + | | + | | + | | + | | | + | | + | | + | | + | | + | | | + | | - | | + | | + | | + | | + | | | + | | + | | + | | + | | + | | | + | | + | | + | | + | | + | + | **putative extramembranal protein, DltD protein** |
| **SSGZ1_0633** | | | | | + | | + | | + | | + | | | + | | + | | + | | + | | + | | | + | | + | | + | | + | | + | | | + | | - | | + | | + | | + | | + | | | + | | + | | + | | + | | + | | | + | | + | | + | | + | | - | + | **putative low temperature requirement A protein** |
| **SSGZ1_0634** | | | | | + | | + | | + | | + | | | + | | + | | + | | + | | + | | | + | | + | | + | | + | | + | | | + | | + | | + | | + | | + | | + | | | + | | + | | + | | + | | + | | | + | | + | | + | | + | | + | + | **Glutathione S-transferase, C-terminal** |
| **SSGZ1_0635** | | | | | + | | + | | + | | + | | | + | | + | | + | | + | | + | | | + | | + | | + | | + | | + | | | + | | + | | + | | + | | + | | + | | | + | | + | | + | | + | | + | | | + | | + | | + | | + | | + | + | **Pseudouridine synthase, Rsu** |
| **SSGZ1_0636** | | | | | + | | + | | + | | + | | | + | | + | | + | | + | | + | | | + | | + | | + | | + | | + | | | + | | + | | + | | + | | + | | + | | | + | | + | | + | | + | | + | | | + | | + | | + | | + | | + | + | **Glutathione peroxidase** |
| **SSGZ1_0637** | | | | | + | | + | | + | | + | | | + | | + | | + | | + | | + | | | + | | + | | + | | + | | + | | | + | | + | | + | | + | | + | | + | | | + | | + | | + | | + | | + | | | + | | + | | + | | + | | + | + | **Ferrichrome transport system permease protein** |
| **SSGZ1_0638** | | | | | + | | + | | + | | + | | | + | | + | | + | | + | | + | | | + | | + | | + | | + | | + | | | + | | + | | + | | + | | + | | + | | | + | | + | | + | | + | | + | | | + | | + | | + | | + | | + | + | **Ferrichrome transport system permease protein** |
| **SSGZ1_0639** | | | | | + | | + | | + | | + | | | + | | + | | + | | + | | + | | | + | | + | | + | | + | | + | | | + | | + | | + | | + | | + | | + | | | + | | + | | + | | + | | + | | | + | | + | | + | | + | | + | + | **Ferrichrome-binding protein precursor** |
| **SSGZ1_0640** | | | | | + | | + | | + | | + | | | + | | + | | + | | + | | + | | | + | | + | | + | | + | | + | | | + | | + | | + | | + | | + | | + | | | + | | + | | + | | + | | + | | | + | | + | | + | | + | | + | + | **Ferrichrome transport ATP-binding protein fhuC** |
| **SSGZ1_0641** | | | | | + | | + | | + | | + | | | + | | + | | + | | + | | + | | | + | | + | | + | | + | | + | | | + | | + | | + | | + | | + | | + | | | + | | - | | + | | + | | + | | | + | | + | | + | | + | | + | + | **UDP-N-acetylmuramoylalanyl-D-glutamate--2,** |
| **SSGZ1_0642** | | | | | + | | + | | + | | + | | | + | | + | | + | | + | | + | | | + | | + | | + | | + | | + | | | + | | + | | + | | + | | + | | + | | | - | | + | | + | | + | | + | | | + | | + | | + | | + | | + | + | **UDP-N-acetylmuramoyl-L-alanyl-D-glutamate--** |
| **SSGZ1_0643** | | | | | + | | + | | + | | + | | | + | | + | | + | | + | | + | | | + | | + | | + | | + | | + | | | + | | + | | + | | + | | + | | + | | | + | | + | | + | | + | | + | | | + | | + | | + | | + | | + | + | **Phospholipid/glycerol acyltransferase** |
| **SSGZ1_0644** | | | | | + | | + | | + | | + | | | + | | + | | + | | + | | + | | | + | | + | | + | | + | | + | | | + | | + | | + | | + | | + | | + | | | + | | + | | + | | + | | + | | | + | | + | | + | | + | | + | + | **Competence protein ComEA helix-hairpin-helix** |
| **SSGZ1_0645** | | | | | + | | + | | + | | + | | | + | | + | | + | | + | | + | | | + | | + | | + | | + | | + | | | + | | + | | + | | + | | + | | + | | | + | | + | | + | | + | | + | | | + | | + | | + | | + | | + | + | **ComEC/Rec2-related protein: DNA** |
| **SSGZ1_0646** | | | | | + | | + | | + | | + | | | + | | + | | + | | + | | + | | | + | | + | | + | | + | | + | | | + | | + | | + | | + | | + | | + | | | + | | + | | + | | + | | + | | | + | | + | | + | | + | | + | + | **putative transposase** |
| **SSGZ1_0647** | | | | | + | | + | | + | | + | | | + | | + | | + | | + | | + | | | + | | + | | + | | + | | + | | | + | | + | | + | | + | | + | | + | | | + | | + | | + | | + | | + | | | + | | + | | + | | + | | + | + | **hypothetical protein** |
| **SSGZ1_0648** | | | | | + | | + | | + | | + | | | + | | + | | + | | + | | + | | | + | | + | | + | | + | | + | | | + | | + | | + | | + | | + | | + | | | + | | + | | + | | + | | + | | | + | | + | | + | | + | | + | + | **hypothetical protein** |
| **SSGZ1_0649** | | | | | + | | + | | + | | + | | | + | | + | | + | | + | | + | | | + | | + | | + | | + | | + | | | + | | + | | + | | + | | + | | + | | | + | | + | | + | | + | | + | | | + | | + | | + | | + | | + | + | **tellurite resistance protein,TelA** |
| **SSGZ1_0650** | | | | | + | | + | | + | | + | | | + | | + | | + | | + | | + | | | + | | + | | + | | + | | + | | | + | | + | | + | | + | | + | | + | | | + | | + | | + | | + | | + | | | + | | + | | + | | + | | + | + | **hypothetical protein** |
| **SSGZ1_0651** | | | | | + | | + | | + | | + | | | + | | + | | + | | + | | + | | | + | | + | | + | | + | | + | | | + | | + | | + | | + | | + | | + | | | + | | + | | + | | + | | + | | | + | | + | | + | | + | | + | + | **Thymidylate kinase** |
| **SSGZ1_0652** | | | | | + | | + | | + | | + | | | + | | + | | + | | + | | + | | | + | | + | | + | | + | | + | | | + | | + | | + | | + | | + | | + | | | + | | + | | + | | + | | + | | | + | | + | | + | | + | | + | + | **putative DNA polymerase III, delta subunit** |
| **SSGZ1_0653** | | | | | + | | + | | + | | + | | | + | | + | | + | | + | | + | | | + | | + | | + | | + | | + | | | + | | + | | + | | + | | + | | + | | | + | | + | | + | | + | | + | | | + | | + | | + | | + | | + | + | **Protein of unknown function DUF972** |
| **SSGZ1_0654** | | | | | + | | + | | + | | + | | | + | | + | | + | | + | | + | | | + | | + | | + | | + | | + | | | + | | + | | + | | + | | + | | + | | | + | | + | | + | | + | | + | | | + | | + | | + | | + | | + | + | **Protein of unknown function UPF0011** |
| **SSGZ1_0655** | | | | | + | | + | | + | | + | | | + | | + | | + | | + | | + | | | + | | + | | + | | + | | + | | | + | | + | | + | | + | | + | | + | | | + | | + | | + | | + | | + | | | + | | + | | + | | + | | + | + | **Phosphoserine aminotransferase** |
| **SSGZ1_0656** | | | | | + | | + | | + | | + | | | + | | + | | + | | + | | + | | | + | | + | | + | | + | | + | | | + | | + | | + | | + | | + | | + | | | + | | + | | + | | + | | + | | | + | | + | | + | | + | | + | + | **GCN5-related N-acetyltransferase** |
| **SSGZ1_0657** | | | | | + | | + | | + | | + | | | + | | + | | + | | + | | + | | | + | | + | | + | | + | | + | | | + | | + | | + | | + | | + | | + | | | + | | + | | + | | + | | + | | | + | | + | | + | | + | | + | + | **putative D-3-phosphoglycerate dehydrogenase** |
| **SSGZ1_0658** | | | | | + | | + | | + | | + | | | + | | + | | + | | + | | + | | | + | | + | | + | | + | | + | | | + | | + | | + | | + | | + | | + | | | + | | + | | + | | + | | + | | | + | | + | | + | | + | | + | + | **Methylated-DNA-(protein)-cysteine** |
| **SSGZ1_0659** | | | | | + | | + | | + | | + | | | + | | + | | + | | + | | + | | | + | | + | | + | | + | | + | | | + | | + | | + | | + | | + | | + | | | + | | + | | + | | + | | + | | | + | | + | | + | | + | | + | + | **hypothetical protein** |
| **SSGZ1_0660** | | | | | + | | + | | + | | + | | | + | | + | | + | | + | | + | | | + | | + | | + | | + | | + | | | + | | + | | + | | + | | + | | - | | | + | | + | | + | | + | | + | | | + | | + | | + | | + | | + | + | **Cation efflux protein** |
| **SSGZ1_0661** | | | | | + | | + | | + | | + | | | + | | + | | + | | + | | + | | | + | | + | | + | | + | | + | | | + | | + | | + | | + | | + | | + | | | + | | + | | + | | + | | + | | | + | | + | | + | | + | | + | + | **exodeoxyribonuclease III** |
| **SSGZ1_0662** | | | | | + | | + | | + | | + | | | + | | + | | + | | + | | + | | | + | | + | | + | | + | | + | | | + | | + | | + | | + | | + | | + | | | + | | + | | + | | + | | + | | | + | | + | | + | | + | | + | + | **hypothetical protein** |
| **SSGZ1_0663** | | | | | + | | + | | + | | + | | | + | | + | | + | | + | | + | | | + | | + | | + | | + | | + | | | + | | + | | + | | + | | + | | + | | | + | | + | | + | | + | | + | | | + | | + | | + | | + | | + | + | **putative amino acid permease** |
| **SSGZ1_0664** | | | | | + | | + | | + | | + | | | + | | + | | + | | + | | + | | | + | | + | | + | | + | | + | | | + | | + | | + | | + | | + | | + | | | + | | + | | + | | + | | + | | | + | | + | | + | | + | | + | + | **Predicted integral membrane protein** |
| **SSGZ1_0665** | | | | | + | | + | | + | | + | | | + | | + | | + | | + | | + | | | + | | + | | + | | + | | + | | | + | | + | | + | | + | | + | | + | | | + | | + | | + | | + | | + | | | + | | + | | + | | + | | + | + | **Predicted integral membrane protein** |
| **SSGZ1_0666** | | | | | + | | + | | + | | + | | | + | | + | | + | | + | | + | | | + | | + | | + | | + | | + | | | + | | + | | + | | + | | + | | + | | | + | | + | | + | | + | | + | | | + | | - | | + | | + | | + | + | **Abortive infection protein** |
| **SSGZ1_0667** | | | | | + | | + | | + | | + | | | + | | + | | + | | + | | + | | | + | | + | | + | | + | | + | | | + | | + | | + | | + | | + | | + | | | + | | + | | + | | + | | + | | | + | | + | | + | | + | | + | + | **hypothetical protein** |
| **SSGZ1_0668** | | | | | + | | + | | + | | + | | | + | | + | | + | | + | | + | | | + | | + | | + | | + | | + | | | + | | + | | + | | + | | + | | + | | | + | | + | | + | | + | | + | | | + | | + | | + | | + | | + | + | **hypothetical protein** |
| **SSGZ1_0669** | | | | | + | | + | | + | | + | | | + | | + | | + | | + | | + | | | + | | + | | + | | + | | + | | | + | | + | | + | | + | | - | | + | | | + | | + | | + | | + | | + | | | + | | + | | + | | + | | + | + | **hypothetical protein** |
| **SSGZ1_0670** | | | | | - | | + | | + | | + | | | + | | + | | + | | + | | + | | | + | | - | | - | | - | | - | | | + | | + | | - | | - | | - | | - | | | + | | + | | - | | + | | + | | | + | | + | | + | | - | | - | + | **TPR repeat** |
| **SSGZ1_0671** | | | | | - | | + | | + | | + | | | + | | + | | + | | + | | + | | | + | | - | | - | | - | | - | | | + | | + | | - | | - | | - | | - | | | + | | + | | - | | + | | + | | | + | | + | | + | | - | | - | + | **hypothetical protein** |
| **SSGZ1_0672** | | | | | - | | + | | + | | + | | | + | | + | | + | | + | | + | | | + | | - | | - | | - | | - | | | + | | + | | - | | - | | - | | - | | | + | | + | | - | | + | | + | | | + | | + | | + | | - | | - | + | **putative modification enzyme of type III** |
| **SSGZ1_0673** | | | | | - | | + | | + | | + | | | + | | + | | + | | + | | + | | | + | | - | | - | | - | | - | | | + | | + | | - | | - | | - | | - | | | + | | + | | - | | + | | + | | | + | | + | | + | | - | | - | + | **type III restriction-modification system,** |
| **SSGZ1_0674** | | | | | + | | + | | + | | + | | | + | | + | | + | | + | | + | | | + | | + | | + | | + | | + | | | + | | + | | + | | + | | + | | + | | | + | | - | | + | | + | | + | | | + | | + | | + | | + | | + | + | **transposase of IS200 family** |
| **SSGZ1_0675** | | | | | + | | + | | + | | + | | | + | | + | | + | | + | | + | | | + | | + | | + | | + | | + | | | + | | + | | + | | + | | + | | + | | | + | | + | | + | | + | | + | | | + | | + | | + | | + | | + | + | **putative phosphomannomutase** |
| **SSGZ1_0676** | | | | | + | | + | | - | | + | | | + | | + | | + | | + | | + | | | + | | + | | + | | + | | + | | | + | | + | | + | | + | | - | | + | | | + | | - | | + | | + | | + | | | + | | + | | + | | + | | + | + | **Predicted membrane protein** |
| **SSGZ1_0677** | | | | | + | | + | | + | | + | | | + | | + | | + | | + | | + | | | + | | + | | + | | + | | + | | | + | | + | | + | | + | | + | | + | | | + | | - | | + | | + | | + | | | + | | + | | + | | + | | + | + | **Flavoprotein** |
| **SSGZ1_0678** | | | | | + | | + | | + | | + | | | + | | + | | + | | + | | + | | | + | | + | | + | | + | | + | | | + | | + | | + | | + | | + | | + | | | + | | + | | + | | + | | + | | | + | | + | | + | | + | | + | + | **Phosphopantothenoylcysteine** |
| **SSGZ1_0679** | | | | | + | | + | | + | | + | | | + | | + | | + | | + | | + | | | + | | + | | + | | + | | + | | | + | | + | | + | | + | | + | | + | | | + | | + | | + | | + | | + | | | + | | + | | + | | + | | + | + | **Formate--tetrahydrofolate ligase** |
| **SSGZ1_0680** | | | | | + | | + | | + | | + | | | + | | + | | + | | + | | + | | | + | | + | | + | | + | | + | | | + | | + | | + | | + | | + | | + | | | + | | + | | + | | + | | + | | | + | | + | | + | | + | | + | + | **Predicted membrane protein** |
| **SSGZ1_0681** | | | | | + | | + | | + | | + | | | + | | + | | + | | + | | + | | | + | | + | | - | | - | | + | | | + | | + | | + | | + | | + | | + | | | + | | + | | + | | + | | + | | | + | | + | | + | | + | | + | - | **FAD-dependent pyridine nucleotide-disulphide** |
| **SSGZ1_0682** | | | | | - | | + | | - | | + | | | + | | + | | + | | + | | + | | | - | | - | | - | | - | | - | | | + | | + | | - | | - | | - | | + | | | + | | + | | + | | + | | + | | | + | | + | | + | | - | | - | - | **putative HsdR** |
| **SSGZ1_0683** | | | | | - | | + | | - | | + | | | + | | + | | + | | + | | + | | | - | | - | | - | | - | | - | | | + | | - | | - | | - | | - | | - | | | + | | + | | - | | + | | + | | | + | | - | | + | | - | | - | - | **putative HsdR** |
| **SSGZ1_0685** | | | | | + | | + | | + | | + | | | + | | + | | + | | + | | + | | | + | | - | | + | | + | | - | | | + | | + | | + | | - | | - | | + | | | + | | + | | - | | + | | + | | | + | | + | | + | | - | | + | + | **Transposase, IS4** |
| **SSGZ1_0687** | | | | | - | | + | | - | | + | | | + | | + | | + | | + | | + | | | - | | + | | - | | + | | - | | | + | | + | | + | | - | | - | | + | | | + | | + | | + | | + | | + | | | + | | + | | + | | + | | + | - | **hypothetical protein** |
| **SSGZ1_0688** | | | | | - | | + | | - | | + | | | + | | + | | + | | + | | + | | | - | | - | | - | | - | | - | | | + | | + | | - | | - | | - | | + | | | + | | + | | + | | + | | + | | | + | | + | | + | | - | | - | - | **putative HsdS** |
| **SSGZ1_0689** | | | | | - | | + | | - | | + | | | + | | + | | + | | + | | + | | | - | | - | | - | | - | | - | | | - | | + | | - | | - | | - | | + | | | + | | + | | + | | - | | - | | | + | | + | | - | | - | | - | - | **hypothetical protein** |
| **SSGZ1_0690** | | | | | - | | + | | - | | + | | | + | | + | | + | | + | | + | | | - | | - | | - | | - | | - | | | - | | - | | - | | - | | - | | - | | | + | | + | | - | | - | | - | | | - | | + | | - | | - | | - | - | **hypothetical protein** |
| **SSGZ1_0691** | | | | | + | | + | | + | | + | | | + | | + | | + | | + | | + | | | + | | + | | + | | + | | + | | | + | | + | | + | | + | | + | | + | | | + | | + | | + | | + | | + | | | + | | + | | + | | + | | + | + | **ATPase (PilT family)** |
| **SSGZ1_0692** | | | | | + | | + | | + | | + | | | + | | + | | + | | + | | + | | | + | | + | | + | | + | | + | | | + | | + | | + | | + | | + | | + | | | + | | + | | + | | + | | + | | | + | | + | | + | | + | | + | + | **GCN5-related N-acetyltransferase** |
| **SSGZ1_0693** | | | | | + | | + | | + | | + | | | + | | + | | + | | + | | + | | | + | | + | | + | | + | | + | | | + | | + | | + | | + | | + | | + | | | + | | + | | + | | + | | + | | | + | | + | | + | | + | | + | + | **DNA alkylation repair enzyme** |
| **SSGZ1_0694** | | | | | + | | + | | + | | + | | | + | | + | | + | | + | | + | | | + | | + | | + | | + | | + | | | + | | + | | + | | + | | + | | + | | | + | | + | | + | | + | | + | | | + | | + | | + | | + | | + | + | **DNA alkylation repair enzyme** |
| **SSGZ1_0695** | | | | | + | | + | | + | | + | | | + | | + | | + | | + | | + | | | + | | + | | + | | + | | + | | | + | | + | | + | | + | | + | | + | | | + | | + | | + | | + | | + | | | + | | + | | + | | + | | + | + | **conserved hypothetical protein** |
| **SSGZ1_0696** | | | | | + | | + | | + | | + | | | + | | + | | + | | + | | + | | | + | | + | | + | | + | | + | | | - | | + | | + | | + | | + | | + | | | + | | + | | + | | - | | - | | | - | | + | | - | | + | | + | + | **Radical SAM** |
| **SSGZ1_0697** | | | | | + | | + | | + | | + | | | + | | + | | + | | + | | + | | | + | | + | | + | | + | | + | | | - | | + | | + | | + | | + | | - | | | + | | + | | + | | - | | - | | | - | | + | | - | | + | | + | + | **regulatory protein, DeoR** |
| **SSGZ1_0698** | | | | | + | | + | | + | | + | | | + | | + | | + | | + | | + | | | + | | + | | + | | + | | + | | | - | | + | | + | | + | | + | | - | | | + | | + | | + | | - | | - | | | - | | + | | - | | + | | + | + | **Sorbitol operon regulator** |
| **SSGZ1_0699** | | | | | + | | + | | + | | + | | | + | | + | | + | | + | | + | | | + | | + | | + | | + | | + | | | - | | + | | + | | + | | + | | - | | | + | | + | | + | | - | | - | | | - | | + | | - | | + | | + | + | **Phosphotransferase system,lactose/cellobiose-specific IIA subunit** |
| **SSGZ1_0700** | | | | | + | | + | | + | | + | | | + | | + | | + | | + | | + | | | + | | + | | + | | + | | + | | | - | | + | | + | | + | | + | | - | | | + | | + | | + | | - | | - | | | - | | - | | - | | + | | + | + | **Phosphotransferase system,lactose/cellobiose-specific IIB subunit** |
| **SSGZ1_0701** | | | | | + | | + | | + | | + | | | + | | + | | + | | + | | + | | | + | | + | | + | | + | | + | | | - | | + | | + | | + | | + | | - | | | + | | + | | + | | - | | - | | | - | | - | | - | | + | | + | + | **PTS lactose/cellobiose IIC component** |
| **SSGZ1_0702** | | | | | + | | + | | + | | + | | | + | | + | | + | | + | | + | | | + | | + | | + | | + | | + | | | - | | + | | + | | + | | + | | - | | | + | | + | | + | | - | | - | | | + | | + | | - | | + | | + | + | **Phosphotransferase system cellobiose-specific component IIC** |
| **SSGZ1_0703** | | | | | + | | + | | + | | + | | | + | | + | | + | | + | | + | | | + | | + | | + | | + | | + | | | - | | + | | + | | + | | + | | - | | | + | | + | | + | | - | | - | | | - | | - | | - | | + | | + | + | **Pyruvate formate-lyase** |
| **SSGZ1_0704** | | | | | + | | + | | + | | + | | | + | | + | | + | | + | | + | | | + | | + | | + | | + | | + | | | - | | + | | + | | + | | + | | - | | | + | | + | | + | | - | | - | | | - | | + | | - | | + | | + | + | **Transaldolase** |
| **SSGZ1_0705** | | | | | + | | + | | + | | + | | | + | | + | | + | | + | | + | | | + | | + | | + | | + | | + | | | - | | + | | + | | + | | + | | - | | | + | | + | | + | | - | | - | | | - | | + | | - | | + | | + | + | **Glycerol dehydrogenase and related enzymes-like protein** |
| **SSGZ1_0706** | | | | | + | | + | | + | | + | | | + | | + | | + | | + | | + | | | + | | + | | + | | + | | + | | | - | | + | | + | | + | | + | | - | | | + | | + | | + | | - | | - | | | - | | - | | - | | + | | + | + | **Glycerol dehydrogenase** |
| **SSGZ1_0707** | | | | | + | | + | | + | | + | | | + | | + | | + | | + | | + | | | + | | + | | + | | + | | + | | | + | | + | | + | | + | | + | | + | | | + | | + | | + | | + | | + | | | + | | + | | + | | + | | + | + | **aspartate-semialdehyde dehydrogenase** |
| **SSGZ1_0708** | | | | | + | | + | | + | | + | | | + | | + | | + | | + | | + | | | + | | + | | + | | + | | + | | | + | | + | | + | | + | | + | | + | | | + | | + | | + | | + | | + | | | + | | + | | + | | + | | + | + | **Dihydrodipicolinate synthase subfamily** |
| **SSGZ1_0709** | | | | | - | | + | | + | | + | | | + | | + | | + | | + | | + | | | + | | - | | + | | + | | + | | | + | | + | | + | | - | | - | | - | | | + | | + | | + | | + | | + | | | + | | + | | + | | + | | + | - | **oxidoreductase, pyridine nucleotide-disulfide** |
| **SSGZ1_0710** | | | | | - | | + | | + | | + | | | + | | + | | + | | + | | + | | | + | | - | | + | | + | | + | | | + | | + | | + | | - | | - | | - | | | + | | + | | + | | + | | + | | | + | | + | | + | | + | | + | - | **hypothetical protein** |
| **SSGZ1_0711** | | | | | - | | + | | + | | + | | | + | | + | | + | | + | | + | | | + | | - | | + | | + | | + | | | + | | + | | + | | - | | - | | - | | | + | | + | | + | | + | | + | | | + | | + | | + | | + | | + | - | **Glycerol kinase** |
| **SSGZ1_0712** | | | | | - | | + | | + | | + | | | + | | + | | + | | + | | + | | | + | | - | | + | | + | | + | | | + | | + | | + | | - | | - | | - | | | + | | + | | + | | + | | + | | | + | | + | | + | | + | | + | - | **Glycerol-3-phosphate dehydrogenase** |
| **SSGZ1_0713** | | | | | - | | + | | + | | + | | | + | | + | | + | | + | | + | | | + | | - | | + | | + | | + | | | + | | + | | + | | - | | - | | - | | | + | | + | | + | | + | | + | | | + | | + | | + | | + | | + | - | **hypothetical protein** |
| **SSGZ1_0714** | | | | | - | | + | | + | | + | | | + | | + | | + | | + | | + | | | + | | - | | + | | + | | + | | | + | | + | | + | | - | | - | | - | | | + | | + | | + | | + | | + | | | + | | + | | + | | + | | + | + | **Glycerol uptake facilitator protein** |
| **SSGZ1_0715** | | | | | - | | + | | + | | + | | | + | | + | | + | | + | | + | | | + | | - | | + | | + | | + | | | + | | + | | + | | - | | - | | - | | | + | | + | | + | | + | | + | | | + | | + | | + | | + | | + | - | **glyoxalase family protein** |
| **SSGZ1_0716** | | | | | - | | + | | + | | + | | | + | | + | | + | | + | | + | | | + | | - | | + | | + | | + | | | + | | + | | + | | - | | - | | + | | | + | | + | | + | | + | | + | | | + | | + | | + | | + | | + | - | **hypothetical protein** |
| **SSGZ1_0717** | | | | | + | | + | | + | | + | | | + | | + | | + | | + | | + | | | + | | + | | + | | + | | + | | | + | | + | | + | | + | | + | | + | | | + | | + | | + | | + | | + | | | + | | + | | + | | + | | + | + | **oligopeptidase F** |
| **SSGZ1_0718** | | | | | + | | + | | + | | + | | | + | | + | | + | | + | | + | | | + | | + | | + | | + | | + | | | + | | + | | + | | + | | + | | + | | | + | | + | | + | | + | | + | | | + | | + | | + | | + | | + | + | **Anaerobic ribonucleoside-triphosphate reductase** |
| **SSGZ1_0719** | | | | | + | | + | | + | | + | | | + | | + | | + | | + | | + | | | + | | + | | + | | + | | + | | | + | | + | | + | | + | | + | | + | | | + | | + | | + | | + | | + | | | + | | + | | + | | + | | + | + | **NADH oxidase** |
| **SSGZ1_0720** | | | | | + | | + | | + | | + | | | + | | + | | + | | + | | + | | | + | | + | | + | | + | | + | | | + | | + | | + | | + | | + | | + | | | + | | + | | + | | + | | + | | | + | | + | | + | | + | | + | + | **Superfamily I DNA and RNA helicase** |
| **SSGZ1_0721** | | | | | + | | + | | + | | + | | | + | | + | | + | | + | | + | | | + | | + | | + | | + | | + | | | + | | + | | + | | + | | + | | + | | | + | | + | | + | | + | | + | | | + | | + | | + | | + | | + | + | **ATP-dependent DNA helicase PcrA** |
| **SSGZ1_0722** | | | | | - | | + | | - | | + | | | + | | + | | + | | + | | + | | | - | | - | | + | | + | | - | | | - | | - | | - | | - | | - | | - | | | + | | + | | - | | + | | + | | | + | | + | | + | | - | | + | - | **putative hydrolase** |
| **SSGZ1_0723** | | | | | - | | + | | - | | + | | | + | | + | | + | | + | | + | | | - | | - | | + | | + | | - | | | - | | - | | - | | - | | - | | - | | | + | | + | | - | | + | | + | | | + | | - | | + | | - | | + | - | **phosphomethylpyrimidine kinase** |
| **SSGZ1_0724** | | | | | - | | + | | - | | + | | | + | | + | | + | | + | | + | | | - | | - | | + | | + | | - | | | - | | - | | - | | - | | - | | - | | | + | | + | | - | | + | | + | | | + | | + | | + | | - | | + | - | **hydroxyethylthiazole kinase** |
| **SSGZ1_0725** | | | | | - | | + | | - | | + | | | + | | + | | + | | + | | + | | | - | | - | | + | | + | | - | | | - | | - | | - | | - | | - | | + | | | + | | + | | - | | + | | + | | | + | | + | | + | | - | | + | - | **thiamine-phosphate pyrophosphorylase** |
| **SSGZ1_0726** | | | | | - | | + | | - | | + | | | + | | + | | + | | + | | + | | | - | | - | | + | | + | | - | | | - | | + | | - | | - | | - | | + | | | + | | + | | - | | + | | + | | | + | | + | | + | | - | | + | - | **Cyclic nucleotide-binding** |
| **SSGZ1_0727** | | | | | - | | + | | - | | + | | | + | | + | | + | | + | | + | | | - | | - | | + | | + | | - | | | - | | - | | - | | - | | - | | - | | | + | | + | | - | | + | | + | | | + | | - | | + | | - | | + | - | **uridine phosphorylase** |
| **SSGZ1_0728** | | | | | - | | + | | - | | + | | | + | | + | | + | | + | | + | | | - | | - | | + | | + | | - | | | - | | - | | - | | - | | - | | - | | | + | | + | | - | | + | | + | | | + | | + | | + | | - | | + | - | **conserved hypothetical protein** |
| **SSGZ1_0729** | | | | | - | | + | | - | | + | | | + | | + | | + | | + | | + | | | - | | - | | + | | + | | - | | | - | | - | | - | | - | | - | | - | | | + | | + | | - | | + | | + | | | + | | - | | + | | - | | + | - | **cobalt ABC transporter permease protein** |
| **SSGZ1_0730** | | | | | - | | + | | - | | + | | | + | | + | | + | | + | | + | | | - | | - | | + | | + | | - | | | - | | - | | - | | - | | - | | - | | | + | | + | | - | | + | | + | | | + | | + | | + | | - | | + | - | **cobalt ABC transporter ATP-binding protein** |
| **SSGZ1_0731** | | | | | - | | + | | - | | + | | | + | | + | | + | | + | | + | | | - | | - | | + | | + | | - | | | - | | - | | - | | - | | - | | - | | | + | | + | | - | | + | | + | | | + | | + | | + | | - | | + | - | **cobalt ABC transporter ATP-binding protein** |
| **SSGZ1_0732** | | | | | + | | + | | + | | + | | | + | | + | | + | | + | | + | | | + | | + | | + | | + | | + | | | + | | + | | + | | + | | + | | + | | | + | | + | | + | | + | | + | | | + | | + | | + | | + | | + | + | **hydrolase, haloacid dehalogenase-like family** |
| **SSGZ1_0733** | | | | | + | | + | | + | | + | | | + | | + | | + | | + | | + | | | + | | + | | + | | + | | + | | | + | | + | | + | | + | | + | | + | | | + | | + | | + | | + | | + | | | + | | + | | + | | + | | + | + | **Abortive infection protein** |
| **SSGZ1_0734** | | | | | + | | + | | + | | + | | | + | | + | | + | | + | | + | | | + | | + | | + | | + | | + | | | + | | + | | + | | + | | + | | + | | | + | | + | | + | | + | | + | | | + | | + | | + | | + | | + | + | **hypothetical protein** |
| **SSGZ1_0735** | | | | | + | | + | | + | | + | | | + | | + | | + | | + | | + | | | + | | + | | + | | + | | + | | | + | | + | | + | | + | | + | | + | | | + | | + | | + | | + | | + | | | + | | + | | + | | + | | + | + | **D-alanyl-D-alanine carboxypeptidase** |
| **SSGZ1_0736** | | | | | + | | + | | + | | + | | | + | | + | | + | | + | | + | | | + | | + | | + | | + | | + | | | + | | + | | + | | + | | + | | + | | | + | | + | | + | | + | | + | | | + | | + | | + | | + | | + | + | **glyoxalase family protein** |
| **SSGZ1_0737** | | | | | + | | + | | + | | + | | | + | | + | | + | | + | | + | | | + | | + | | + | | + | | + | | | + | | + | | + | | + | | + | | + | | | + | | + | | + | | + | | + | | | + | | + | | + | | + | | + | + | **Predicted permease** |
| **SSGZ1_0738** | | | | | + | | + | | + | | + | | | + | | + | | + | | + | | + | | | + | | + | | + | | + | | + | | | + | | + | | + | | + | | + | | + | | | + | | + | | + | | + | | + | | | + | | + | | + | | + | | + | + | **putative ABC transporter, ATP-binding protein** |
| **SSGZ1_0739** | | | | | + | | + | | + | | + | | | + | | + | | + | | + | | + | | | + | | + | | + | | + | | + | | | + | | + | | + | | + | | + | | + | | | + | | + | | + | | + | | + | | | + | | + | | + | | + | | + | + | **excinuclease ABC, subunit C** |
| **SSGZ1_0740** | | | | | + | | + | | + | | + | | | + | | + | | + | | + | | + | | | + | | + | | + | | + | | + | | | + | | + | | + | | + | | + | | + | | | + | | + | | + | | + | | + | | | + | | + | | + | | + | | + | + | **conserved hypothetical protein** |
| **SSGZ1_0741** | | | | | + | | + | | + | | + | | | + | | + | | + | | + | | + | | | + | | + | | + | | + | | + | | | + | | + | | + | | + | | + | | + | | | + | | + | | + | | + | | + | | | + | | + | | + | | + | | + | + | **GTP-binding protein, GTP1/Obg family** |
| **SSGZ1_0742** | | | | | + | | + | | + | | + | | | + | | + | | + | | + | | + | | | + | | + | | + | | + | | + | | | + | | + | | + | | + | | + | | + | | | + | | + | | + | | + | | + | | | + | | + | | + | | + | | + | + | **conserved hypothetical protein** |
| **SSGZ1_0743** | | | | | + | | + | | + | | + | | | + | | + | | + | | + | | + | | | + | | + | | + | | + | | - | | | + | | + | | - | | + | | + | | + | | | + | | - | | - | | + | | + | | | + | | + | | + | | - | | + | - | **'muramidase-released protein** |
| **SSGZ1_0744** | | | | | + | | + | | + | | + | | | + | | + | | + | | + | | + | | | + | | + | | + | | + | | + | | | + | | + | | + | | + | | + | | + | | | + | | + | | + | | + | | + | | | + | | + | | + | | + | | + | + | **putative exonuclease RexB** |
| **SSGZ1_0745** | | | | | + | | + | | + | | + | | | + | | + | | + | | + | | + | | | + | | + | | + | | + | | + | | | + | | + | | + | | + | | + | | + | | | + | | + | | + | | + | | + | | | + | | + | | + | | + | | + | + | **putative exonuclease RexA** |
| **SSGZ1_0746** | | | | | + | | + | | + | | + | | | + | | + | | + | | + | | + | | | + | | + | | + | | + | | + | | | + | | + | | + | | + | | + | | + | | | + | | + | | + | | + | | + | | | + | | + | | + | | + | | + | + | **putative membrane protein** |
| **SSGZ1_0747** | | | | | + | | + | | + | | + | | | + | | + | | + | | + | | + | | | + | | + | | + | | + | | + | | | + | | + | | + | | + | | + | | + | | | + | | + | | + | | + | | + | | | + | | + | | + | | + | | + | + | **topoisomerase IV subunit B** |
| **SSGZ1_0748** | | | | | - | | + | | - | | + | | | + | | + | | + | | + | | + | | | - | | - | | + | | + | | + | | | - | | + | | - | | + | | - | | + | | | + | | + | | + | | - | | - | | | + | | + | | + | | + | | + | - | **ABC-type phosphate transport system, ATPase** |
| **SSGZ1_0749** | | | | | + | | + | | + | | + | | | + | | + | | + | | + | | + | | | + | | + | | - | | - | | + | | | + | | + | | + | | + | | + | | + | | | + | | + | | + | | + | | + | | | + | | + | | + | | + | | + | - | **Protein involved in cell division** |
| **SSGZ1_0750** | | | | | + | | + | | + | | + | | | + | | + | | + | | + | | + | | | + | | + | | - | | - | | - | | | + | | + | | + | | + | | + | | + | | | + | | + | | + | | + | | + | | | + | | + | | + | | + | | + | - | **Filamentation induced by cAMP protein Fic** |
| **SSGZ1_0751** | | | | | + | | + | | + | | + | | | + | | + | | + | | + | | + | | | + | | + | | + | | + | | - | | | + | | + | | - | | + | | + | | - | | | + | | + | | - | | + | | + | | | + | | + | | + | | - | | + | + | **hypothetical protein** |
| **SSGZ1_0752** | | | | | + | | + | | + | | + | | | + | | + | | + | | + | | + | | | + | | + | | + | | + | | - | | | + | | + | | - | | + | | + | | - | | | + | | + | | - | | + | | + | | | + | | + | | + | | - | | + | + | **hypothetical protein** |
| **SSGZ1_0753** | | | | | + | | + | | + | | + | | | + | | + | | + | | + | | + | | | + | | + | | + | | + | | + | | | + | | + | | + | | + | | + | | + | | | + | | + | | + | | + | | + | | | + | | + | | + | | + | | + | + | **topoisomerase IV, subunit A** |
| **SSGZ1_0754** | | | | | + | | + | | + | | + | | | + | | + | | + | | + | | + | | | + | | + | | + | | + | | + | | | + | | + | | + | | + | | + | | + | | | + | | + | | + | | + | | + | | | + | | + | | + | | + | | + | + | **conserved hypothetical protein** |
| **SSGZ1_0755** | | | | | + | | + | | + | | + | | | + | | + | | + | | + | | + | | | + | | + | | + | | + | | + | | | + | | + | | + | | + | | + | | + | | | + | | + | | + | | + | | + | | | + | | + | | + | | + | | + | + | **Phosphoribosylformylglycinamidine (FGAM)** |
| **SSGZ1_0756** | | | | | + | | + | | + | | + | | | + | | + | | + | | + | | + | | | + | | + | | + | | + | | + | | | + | | + | | + | | + | | + | | + | | | + | | + | | + | | + | | + | | | + | | + | | + | | + | | + | + | **branched-chain amino acid aminotransferase** |
| **SSGZ1_0757** | | | | | + | | + | | + | | + | | | + | | + | | + | | + | | + | | | + | | + | | + | | + | | + | | | + | | + | | + | | + | | + | | + | | | + | | + | | + | | + | | + | | | + | | + | | + | | + | | + | + | **tRNA-Gln** |
| **SSGZ1_0758** | | | | | + | | + | | + | | + | | | + | | + | | + | | + | | + | | | + | | + | | + | | + | | + | | | + | | + | | + | | + | | + | | + | | | + | | + | | + | | + | | + | | | + | | + | | + | | + | | + | + | **tRNA-Arg** |
| **SSGZ1_0759** | | | | | + | | + | | + | | + | | | + | | + | | + | | + | | + | | | + | | + | | + | | + | | + | | | + | | + | | + | | + | | + | | + | | | + | | + | | + | | + | | + | | | + | | + | | + | | + | | + | + | **Threonine aldolase** |
| **SSGZ1_0760** | | | | | + | | + | | + | | + | | | + | | + | | + | | + | | + | | | + | | + | | + | | + | | + | | | + | | + | | + | | + | | + | | + | | | + | | + | | + | | - | | - | | | + | | + | | - | | + | | + | + | **hypothetical protein** |
| **SSGZ1_0761** | | | | | + | | + | | + | | + | | | + | | + | | + | | + | | + | | | + | | + | | + | | + | | + | | | + | | + | | + | | + | | + | | + | | | + | | + | | + | | - | | - | | | - | | + | | - | | + | | + | + | **hypothetical protein** |
| **SSGZ1_0762** | | | | | + | | + | | + | | + | | | + | | + | | + | | + | | + | | | + | | + | | + | | + | | + | | | + | | + | | + | | + | | + | | + | | | + | | + | | + | | + | | + | | | + | | + | | + | | + | | + | + | **Aminotransferase, class V** |
| **SSGZ1_0763** | | | | | + | | + | | + | | + | | | + | | + | | + | | + | | + | | | + | | + | | + | | + | | + | | | + | | + | | + | | + | | + | | + | | | + | | + | | + | | + | | + | | | + | | + | | + | | + | | + | + | **Thiamine biosynthesis protein** |
| **SSGZ1_0764** | | | | | + | | + | | + | | + | | | + | | + | | + | | + | | + | | | + | | + | | + | | + | | + | | | + | | + | | + | | + | | + | | + | | | + | | + | | + | | + | | + | | | + | | + | | + | | + | | + | + | **Branched-chain amino acid transport system II** |
| **SSGZ1_0765** | | | | | + | | + | | + | | + | | | + | | + | | + | | + | | + | | | + | | + | | + | | + | | + | | | + | | + | | + | | + | | + | | + | | | + | | + | | + | | + | | + | | | + | | + | | + | | + | | + | + | **ribosomal protein L21** |
| **SSGZ1_0766** | | | | | + | | + | | + | | + | | | + | | + | | + | | + | | + | | | + | | + | | + | | + | | + | | | + | | + | | + | | + | | + | | + | | | + | | + | | + | | + | | + | | | + | | + | | + | | + | | + | + | **ribosomal protein L27** |
| **SSGZ1_0767** | | | | | + | | + | | + | | + | | | + | | + | | + | | + | | + | | | + | | + | | + | | + | | + | | | + | | + | | + | | + | | + | | + | | | + | | + | | + | | + | | + | | | + | | + | | + | | + | | + | + | **C-P lyase regulatory protein** |
| **SSGZ1_0768** | | | | | + | | + | | + | | + | | | + | | + | | + | | + | | + | | | + | | + | | + | | + | | + | | | + | | + | | + | | + | | + | | + | | | + | | + | | + | | + | | + | | | + | | + | | + | | + | | + | + | **CpsY** |
| **SSGZ1_0769** | | | | | + | | + | | + | | + | | | + | | + | | + | | + | | + | | | + | | + | | + | | + | | + | | | + | | + | | + | | + | | + | | + | | | + | | + | | + | | + | | + | | | + | | + | | + | | + | | + | + | **prolipoprotein signal peptidase** |
| **SSGZ1_0770** | | | | | + | | + | | + | | + | | | + | | + | | + | | + | | + | | | + | | + | | + | | + | | + | | | + | | + | | + | | + | | + | | + | | | + | | + | | + | | + | | + | | | + | | + | | + | | + | | + | + | **YlyB** |
| **SSGZ1_0771** | | | | | + | | + | | + | | + | | | + | | + | | + | | + | | + | | | + | | + | | + | | + | | + | | | + | | + | | + | | + | | + | | + | | | + | | + | | + | | + | | + | | | + | | + | | + | | + | | + | + | **phnA protein** |
| **SSGZ1_0772** | | | | | + | | + | | + | | + | | | + | | + | | + | | + | | + | | | + | | + | | + | | + | | + | | | + | | + | | + | | + | | + | | + | | | + | | + | | + | | + | | + | | | + | | + | | + | | + | | + | + | **hypothetical protein** |
| **SSGZ1_0773** | | | | | + | | + | | + | | + | | | + | | + | | + | | + | | + | | | + | | + | | + | | + | | + | | | + | | + | | + | | + | | + | | + | | | + | | + | | + | | + | | + | | | + | | + | | + | | + | | + | + | **Uracil phosphoribosyltransferase** |
| **SSGZ1_0774** | | | | | + | | + | | + | | + | | | + | | + | | + | | + | | + | | | + | | + | | + | | + | | + | | | + | | + | | + | | + | | + | | + | | | + | | + | | + | | + | | + | | | + | | + | | + | | + | | + | + | **Aspartate carbamoyltransferase** |
| **SSGZ1_0775** | | | | | + | | + | | + | | + | | | + | | + | | + | | + | | + | | | + | | + | | + | | + | | + | | | + | | + | | + | | + | | + | | + | | | + | | + | | + | | + | | + | | | + | | + | | + | | + | | + | + | **Carbamoyl-phosphate synthase, small subunit** |
| **SSGZ1_0776** | | | | | + | | + | | + | | + | | | + | | + | | + | | + | | + | | | + | | + | | + | | + | | + | | | + | | + | | + | | + | | + | | + | | | + | | + | | + | | + | | + | | | + | | + | | + | | + | | + | + | **Carbamoyl-phosphate synthase, large subunit** |
| **SSGZ1_0777** | | | | | + | | + | | + | | + | | | + | | + | | + | | + | | + | | | + | | + | | + | | + | | + | | | + | | + | | + | | + | | + | | + | | | + | | + | | + | | + | | + | | | + | | + | | + | | + | | + | + | **SAM-dependent methyltransferases** |
| **SSGZ1_0778** | | | | | + | | + | | + | | + | | | + | | + | | + | | + | | + | | | + | | + | | + | | + | | + | | | + | | + | | + | | + | | + | | + | | | + | | + | | + | | + | | + | | | + | | + | | + | | + | | + | + | **pyrrolidone-carboxylate peptidase** |
| **SSGZ1_0779** | | | | | + | | + | | + | | + | | | + | | + | | + | | + | | + | | | + | | + | | + | | + | | + | | | + | | + | | + | | + | | + | | + | | | + | | + | | + | | + | | + | | | + | | + | | + | | + | | + | + | **hypothetical protein** |
| **SSGZ1_0780** | | | | | + | | + | | + | | + | | | + | | + | | + | | + | | + | | | + | | + | | + | | + | | + | | | + | | + | | + | | + | | + | | + | | | + | | + | | + | | + | | + | | | + | | + | | + | | + | | + | + | **ribosomal protein S16** |
| **SSGZ1_0781** | | | | | + | | + | | + | | + | | | + | | + | | + | | + | | + | | | + | | + | | + | | + | | + | | | + | | + | | + | | + | | + | | + | | | + | | + | | + | | + | | + | | | + | | + | | + | | + | | + | + | **KH domain protein** |
| **SSGZ1_0782** | | | | | + | | + | | + | | + | | | + | | + | | + | | + | | + | | | + | | + | | + | | + | | + | | | + | | + | | + | | + | | + | | + | | | + | | + | | + | | + | | + | | | + | | + | | + | | + | | + | + | **putative ABC transporter, ATP-binding protein** |
| **SSGZ1_0783** | | | | | + | | + | | + | | + | | | + | | + | | + | | + | | + | | | + | | + | | + | | + | | + | | | + | | + | | + | | + | | + | | + | | | + | | + | | + | | + | | + | | | + | | + | | + | | + | | + | + | **ABC transporter, transmembrane region containing** |
| **SSGZ1_0784** | | | | | + | | + | | + | | + | | | + | | + | | + | | + | | + | | | + | | + | | + | | + | | + | | | + | | + | | + | | + | | + | | + | | | + | | + | | + | | + | | + | | | + | | + | | + | | + | | + | + | **Polysaccharide deacetylase** |
| **SSGZ1_0785** | | | | | + | | + | | + | | + | | | + | | + | | + | | + | | + | | | + | | + | | + | | + | | + | | | + | | + | | + | | + | | + | | + | | | + | | + | | + | | + | | + | | | + | | + | | + | | + | | + | + | **Homoserine dehydrogenase** |
| **SSGZ1_0786** | | | | | + | | + | | + | | + | | | + | | + | | + | | + | | + | | | + | | + | | + | | + | | + | | | + | | + | | + | | + | | + | | + | | | + | | + | | + | | + | | + | | | + | | + | | + | | + | | + | + | **Homoserine kinase** |
| **SSGZ1_0787** | | | | | + | | + | | + | | + | | | + | | + | | + | | + | | + | | | + | | + | | + | | + | | + | | | + | | + | | + | | + | | + | | + | | | + | | + | | + | | + | | + | | | + | | + | | + | | + | | + | + | **UDP-N-acetylmuramate dehydrogenase** |
| **SSGZ1_0788** | | | | | + | | + | | + | | + | | | + | | + | | + | | + | | + | | | + | | + | | + | | + | | + | | | + | | + | | + | | + | | + | | + | | | + | | + | | + | | + | | + | | | + | | + | | + | | + | | + | + | **spermidine/putrescine ABC transporter,** |
| **SSGZ1_0789** | | | | | + | | + | | + | | + | | | + | | + | | + | | + | | + | | | + | | + | | + | | + | | + | | | + | | + | | + | | + | | + | | + | | | + | | + | | + | | + | | + | | | + | | + | | + | | + | | + | + | **Binding-protein-dependent transport systems** |
| **SSGZ1_0790** | | | | | + | | + | | + | | + | | | + | | + | | + | | + | | + | | | + | | + | | + | | + | | + | | | + | | + | | + | | + | | + | | + | | | + | | + | | + | | + | | + | | | + | | + | | + | | + | | + | + | **Binding-protein-dependent transport systems** |
| **SSGZ1_0791** | | | | | + | | + | | + | | + | | | + | | + | | + | | + | | + | | | + | | + | | + | | + | | + | | | + | | + | | + | | + | | + | | + | | | + | | + | | + | | + | | + | | | + | | + | | + | | + | | + | + | **extracellular solute-binding protein, family 1** |
| **SSGZ1_0792** | | | | | + | | + | | + | | + | | | + | | + | | + | | + | | + | | | + | | + | | + | | + | | - | | | + | | - | | - | | + | | + | | + | | | + | | + | | - | | - | | - | | | - | | + | | - | | - | | + | + | **Chloride channel protein, EriC** |
| **SSGZ1_0793** | | | | | + | | + | | + | | + | | | + | | + | | + | | + | | + | | | + | | + | | + | | + | | + | | | - | | - | | + | | + | | + | | + | | | + | | + | | + | | + | | + | | | + | | + | | + | | + | | + | + | **Transposase and inactivated derivative** |
| **SSGZ1_0794** | | | | | + | | + | | + | | + | | | + | | + | | + | | + | | + | | | + | | + | | + | | + | | + | | | + | | + | | + | | + | | + | | + | | | + | | + | | + | | + | | + | | | + | | + | | - | | + | | + | + | **YSIRK Gram-positive signal peptide** |
| **SSGZ1_0795** | | | | | + | | + | | + | | + | | | + | | + | | + | | + | | + | | | + | | + | | + | | + | | + | | | + | | + | | + | | + | | + | | + | | | + | | + | | + | | + | | + | | | + | | + | | + | | + | | + | + | **Predicted sugar kinase** |
| **SSGZ1_0796** | | | | | + | | + | | + | | + | | | + | | + | | + | | + | | + | | | + | | + | | + | | + | | + | | | + | | + | | + | | + | | + | | + | | | + | | + | | + | | + | | + | | | + | | + | | + | | + | | + | + | **Endonuclease III/Nth** |
| **SSGZ1_0797** | | | | | + | | + | | + | | + | | | + | | + | | + | | + | | + | | | + | | + | | + | | + | | + | | | + | | + | | + | | + | | + | | + | | | + | | + | | + | | + | | + | | | + | | + | | + | | + | | + | + | **GMP reductase** |
| **SSGZ1_0798** | | | | | + | | + | | + | | + | | | + | | + | | + | | + | | + | | | + | | + | | + | | + | | + | | | + | | + | | + | | + | | + | | + | | | + | | + | | + | | + | | + | | | + | | + | | + | | + | | + | + | **MATE efflux family protein** |
| **SSGZ1_0799** | | | | | + | | + | | + | | + | | | + | | + | | + | | + | | + | | | + | | + | | + | | + | | + | | | + | | + | | + | | + | | + | | + | | | + | | + | | + | | + | | + | | | + | | + | | + | | + | | + | + | **multidrug resistance like protein** |
| **SSGZ1_0800** | | | | | + | | + | | + | | + | | | + | | + | | + | | + | | + | | | + | | + | | + | | + | | + | | | + | | + | | + | | + | | + | | + | | | + | | + | | + | | + | | + | | | + | | + | | + | | + | | + | + | **RimM** |
| **SSGZ1_0801** | | | | | + | | + | | + | | + | | | + | | + | | + | | + | | + | | | + | | + | | + | | + | | + | | | + | | + | | + | | + | | + | | + | | | + | | + | | + | | + | | + | | | + | | + | | + | | + | | + | + | **tRNA (guanine-N1-)-methyltransferase** |
| **SSGZ1_0802** | | | | | + | | + | | + | | + | | | + | | + | | + | | + | | + | | | + | | + | | + | | + | | + | | | + | | + | | + | | + | | + | | + | | | + | | + | | + | | + | | + | | | + | | + | | + | | + | | + | + | **Aminotransferase, class I and II** |
| **SSGZ1_0803** | | | | | + | | + | | + | | + | | | + | | + | | + | | + | | + | | | + | | + | | + | | + | | + | | | + | | + | | + | | + | | + | | + | | | + | | + | | + | | + | | + | | | + | | + | | + | | + | | + | + | **regulatory protein DeoR** |
| **SSGZ1_0804** | | | | | + | | + | | + | | + | | | + | | + | | + | | + | | + | | | + | | + | | + | | + | | + | | | + | | + | | + | | + | | + | | + | | | + | | + | | + | | + | | + | | | + | | + | | + | | + | | + | + | **1-phosphofructokinase** |
| **SSGZ1_0805** | | | | | + | | + | | + | | + | | | + | | + | | + | | + | | + | | | + | | + | | + | | + | | + | | | + | | + | | + | | + | | + | | + | | | + | | + | | + | | + | | + | | | + | | + | | + | | + | | + | + | **PTS system related protein** |
| **SSGZ1_0806** | | | | | + | | + | | + | | + | | | + | | + | | + | | + | | + | | | + | | + | | + | | + | | + | | | + | | + | | + | | + | | + | | + | | | + | | + | | + | | + | | + | | | + | | + | | + | | + | | + | + | **hypothetical protein** |
| **SSGZ1_0807** | | | | | + | | + | | + | | + | | | + | | + | | + | | + | | + | | | + | | + | | + | | + | | + | | | + | | + | | + | | + | | + | | + | | | + | | + | | + | | + | | + | | | + | | + | | + | | + | | + | + | **DegV** |
| **SSGZ1_0808** | | | | | + | | + | | + | | + | | | + | | + | | + | | + | | + | | | + | | + | | + | | + | | + | | | + | | + | | + | | + | | + | | + | | | + | | + | | + | | + | | + | | | + | | + | | + | | + | | + | + | **Dihydrodipicolinate reductase** |
| **SSGZ1_0809** | | | | | + | | + | | + | | + | | | + | | + | | + | | + | | + | | | + | | + | | + | | + | | + | | | + | | + | | + | | + | | + | | + | | | + | | + | | + | | + | | + | | | + | | + | | + | | + | | + | + | **Polynucleotide adenylyltransferase** |
| **SSGZ1_0810** | | | | | + | | + | | + | | + | | | + | | + | | + | | + | | + | | | + | | + | | + | | + | | + | | | + | | + | | + | | + | | + | | + | | | + | | + | | + | | + | | + | | | + | | + | | + | | + | | + | + | **spermidine/putrescine ABC transporter,** |
| **SSGZ1_0811** | | | | | + | | + | | + | | + | | | + | | + | | + | | + | | + | | | + | | + | | + | | + | | + | | | + | | + | | + | | + | | + | | + | | | + | | + | | + | | + | | + | | | + | | + | | + | | + | | + | + | **hypothetical protein** |
| **SSGZ1_0812** | | | | | + | | + | | + | | + | | | + | | + | | + | | + | | + | | | + | | + | | + | | + | | + | | | + | | + | | + | | + | | + | | + | | | + | | + | | + | | + | | + | | | + | | + | | + | | + | | + | + | **Glucokinase ROK** |
| **SSGZ1_0813** | | | | | + | | + | | + | | + | | | + | | + | | + | | + | | + | | | + | | + | | + | | + | | + | | | + | | + | | + | | + | | + | | + | | | + | | + | | + | | + | | + | | | + | | + | | + | | + | | + | + | **hypothetical protein** |
| **SSGZ1_0814** | | | | | + | | + | | + | | + | | | + | | + | | + | | + | | + | | | + | | + | | + | | + | | + | | | + | | + | | + | | + | | + | | + | | | + | | + | | + | | + | | + | | | + | | + | | + | | + | | + | + | **hypothetical protein** |
| **SSGZ1_0815** | | | | | + | | + | | + | | + | | | + | | + | | + | | + | | + | | | + | | + | | + | | + | | + | | | + | | + | | + | | + | | + | | + | | | + | | + | | + | | + | | + | | | + | | + | | + | | + | | + | + | **Thymidylate synthase** |
| **SSGZ1_0816** | | | | | + | | + | | + | | + | | | + | | + | | + | | + | | + | | | + | | + | | + | | + | | + | | | + | | + | | + | | + | | + | | + | | | + | | + | | + | | + | | + | | | + | | + | | + | | + | | + | + | **Dihydrofolate reductase** |
| **SSGZ1_0817** | | | | | + | | + | | + | | + | | | + | | + | | + | | + | | + | | | + | | + | | + | | + | | + | | | + | | + | | + | | + | | + | | + | | | + | | + | | + | | + | | + | | | + | | + | | + | | + | | + | + | **conserved hypothetical protein** |
| **SSGZ1_0818** | | | | | + | | + | | + | | + | | | + | | + | | + | | + | | + | | | + | | + | | + | | + | | + | | | + | | + | | + | | + | | + | | + | | | + | | + | | + | | + | | + | | | + | | + | | + | | + | | + | + | **ClpX, ATPase regulatory subunit** |
| **SSGZ1_0819** | | | | | + | | + | | + | | + | | | + | | + | | + | | + | | + | | | + | | + | | + | | + | | + | | | + | | + | | + | | + | | + | | + | | | + | | + | | + | | + | | + | | | + | | + | | + | | + | | + | + | **GTP-binding protein domain containing protein** |
| **SSGZ1_0820** | | | | | + | | + | | + | | + | | | + | | + | | + | | + | | + | | | + | | + | | + | | + | | + | | | + | | + | | + | | + | | + | | + | | | + | | + | | + | | + | | + | | | + | | + | | + | | + | | + | + | **Nucleoside-diphosphate kinase** |
| **SSGZ1_0821** | | | | | - | | + | | - | | + | | | + | | + | | + | | + | | + | | | - | | - | | - | | - | | - | | | - | | + | | - | | - | | + | | + | | | + | | + | | + | | - | | - | | | - | | + | | - | | - | | - | - | **hypothetical protein** |
| **SSGZ1_0822** | | | | | + | | + | | + | | + | | | + | | + | | + | | + | | + | | | + | | + | | + | | + | | + | | | + | | + | | + | | + | | + | | + | | | + | | + | | + | | + | | + | | | + | | + | | + | | + | | + | + | **Membrane GTPase LepA** |
| **SSGZ1_0823** | | | | | + | | + | | + | | + | | | + | | + | | + | | + | | + | | | + | | + | | + | | + | | + | | | + | | + | | + | | + | | + | | + | | | + | | + | | + | | + | | + | | | + | | + | | + | | + | | + | + | **4-oxalocrotonate tautomerase** |
| **SSGZ1_0824** | | | | | + | | + | | + | | + | | | + | | + | | + | | + | | + | | | + | | + | | + | | + | | + | | | + | | + | | + | | + | | + | | + | | | + | | + | | + | | + | | + | | | + | | + | | + | | + | | + | + | **hypothetical protein** |
| **SSGZ1_0825** | | | | | + | | + | | + | | + | | | + | | + | | + | | + | | + | | | + | | + | | + | | + | | + | | | + | | + | | + | | + | | + | | + | | | + | | + | | + | | + | | + | | | + | | + | | + | | + | | + | + | **hypothetical protein** |
| **SSGZ1_0826** | | | | | + | | + | | + | | + | | | + | | + | | + | | + | | + | | | + | | + | | + | | + | | + | | | + | | + | | + | | + | | + | | + | | | + | | + | | + | | + | | + | | | + | | + | | + | | + | | + | + | **Thymidine kinase** |
| **SSGZ1_0827** | | | | | + | | + | | + | | + | | | + | | + | | + | | + | | + | | | + | | + | | + | | + | | + | | | + | | + | | + | | + | | + | | + | | | + | | + | | + | | + | | + | | | + | | + | | + | | + | | + | + | **Peptide chain release factor 1** |
| **SSGZ1_0828** | | | | | + | | + | | + | | + | | | + | | + | | + | | + | | + | | | + | | + | | + | | + | | + | | | + | | + | | + | | + | | + | | + | | | + | | + | | + | | + | | + | | | + | | + | | + | | + | | + | + | **Modification methylase HemK** |
| **SSGZ1_0829** | | | | | + | | + | | + | | + | | | + | | + | | + | | + | | + | | | + | | + | | + | | + | | + | | | + | | + | | + | | + | | + | | + | | | + | | + | | + | | + | | + | | | + | | + | | + | | + | | + | + | **Sua5/YciO/YrdC/YwlC family protein** |
| **SSGZ1_0830** | | | | | + | | + | | + | | + | | | + | | + | | + | | + | | + | | | + | | + | | + | | + | | + | | | + | | + | | + | | + | | + | | + | | | + | | + | | + | | + | | + | | | + | | + | | + | | + | | + | + | **acetyltransferase, GNAT family protein** |
| **SSGZ1_0831** | | | | | + | | + | | + | | + | | | + | | + | | + | | + | | + | | | + | | + | | + | | + | | + | | | + | | + | | + | | + | | + | | + | | | + | | + | | + | | + | | + | | | + | | + | | + | | + | | + | + | **Glycine hydroxymethyltransferase** |
| **SSGZ1_0832** | | | | | + | | + | | + | | + | | | + | | + | | + | | + | | + | | | + | | + | | + | | + | | + | | | + | | + | | + | | + | | + | | + | | | + | | + | | + | | + | | + | | | + | | + | | + | | + | | + | + | **hypothetical protein** |
| **SSGZ1_0833** | | | | | + | | + | | + | | + | | | + | | + | | + | | + | | + | | | + | | + | | + | | + | | + | | | + | | + | | + | | + | | + | | + | | | + | | + | | + | | + | | + | | | + | | + | | + | | + | | + | + | **pneumococcal vaccine antigen A -like protein** |
| **SSGZ1_0834** | | | | | + | | + | | + | | + | | | + | | + | | + | | + | | + | | | + | | + | | + | | + | | + | | | + | | + | | + | | + | | + | | + | | | + | | + | | + | | + | | + | | | + | | + | | + | | + | | + | + | **reductase SDR** |
| **SSGZ1_0835** | | | | | + | | + | | + | | + | | | + | | + | | + | | + | | + | | | + | | + | | + | | + | | + | | | + | | + | | + | | + | | + | | + | | | + | | + | | + | | + | | + | | | + | | + | | + | | + | | + | + | **Translation initiation factor 1 (IF-1)** |
| **SSGZ1_0836** | | | | | + | | + | | + | | + | | | + | | + | | + | | + | | + | | | + | | + | | + | | + | | + | | | + | | + | | + | | + | | + | | + | | | + | | + | | + | | + | | + | | | + | | + | | + | | + | | + | + | **hypothetical protein** |
| **SSGZ1_0837** | | | | | + | | + | | + | | + | | | + | | + | | + | | + | | + | | | + | | + | | + | | + | | + | | | + | | + | | + | | + | | + | | + | | | + | | + | | + | | + | | + | | | + | | + | | + | | + | | + | + | **hypothetical protein** |
| **SSGZ1_0838** | | | | | + | | + | | + | | + | | | + | | + | | + | | + | | + | | | + | | + | | + | | + | | + | | | + | | + | | + | | + | | + | | + | | | + | | + | | + | | + | | + | | | + | | + | | + | | + | | + | + | **hypothetical protein** |
| **SSGZ1_0839** | | | | | + | | + | | - | | + | | | + | | + | | + | | + | | + | | | - | | + | | + | | + | | + | | | + | | + | | + | | + | | + | | + | | | + | | + | | + | | + | | + | | | + | | + | | + | | + | | + | + | **ATPase** |
| **SSGZ1_0840** | | | | | + | | + | | - | | + | | | + | | + | | + | | + | | + | | | - | | + | | + | | + | | + | | | + | | + | | + | | + | | + | | + | | | + | | + | | + | | + | | + | | | + | | + | | + | | + | | + | + | **putative restriction enzyme modulator protein** |
| **SSGZ1_0841** | | | | | + | | + | | + | | + | | | + | | + | | + | | + | | + | | | + | | + | | + | | + | | + | | | + | | + | | + | | + | | + | | + | | | + | | + | | + | | + | | + | | | + | | + | | + | | + | | + | + | **GMP synthase** |
| **SSGZ1_0842** | | | | | + | | + | | + | | + | | | + | | + | | + | | + | | + | | | + | | + | | + | | + | | + | | | + | | + | | + | | + | | + | | + | | | + | | + | | + | | + | | + | | | + | | + | | + | | + | | + | + | **Bacterial regulatory protein, GntR family** |
| **SSGZ1_0843** | | | | | + | | + | | + | | + | | | + | | + | | + | | + | | + | | | + | | + | | + | | + | | + | | | + | | + | | + | | + | | + | | + | | | + | | + | | + | | + | | + | | | + | | + | | + | | + | | + | + | **Putative helix-turn-helix protein** |
| **SSGZ1_0844** | | | | | + | | + | | + | | + | | | + | | + | | + | | + | | + | | | + | | + | | + | | + | | + | | | + | | + | | + | | + | | + | | + | | | + | | + | | + | | + | | + | | | + | | + | | + | | + | | + | + | **Signal recognition particle protein** |
| **SSGZ1_0845** | | | | | + | | + | | + | | + | | | + | | + | | + | | + | | + | | | + | | + | | + | | + | | + | | | + | | + | | + | | + | | + | | + | | | + | | + | | + | | + | | + | | | + | | + | | + | | + | | + | + | **hypothetical protein** |
| **SSGZ1_0846** | | | | | + | | + | | + | | + | | | + | | + | | + | | + | | + | | | + | | + | | + | | + | | + | | | + | | + | | + | | + | | + | | + | | | + | | + | | + | | + | | + | | | + | | + | | + | | + | | + | + | **hypothetical protein** |
| **SSGZ1_0847** | | | | | + | | + | | + | | + | | | + | | + | | + | | + | | + | | | + | | + | | + | | + | | + | | | + | | + | | + | | + | | + | | + | | | + | | + | | + | | + | | + | | | + | | + | | + | | + | | + | + | **Adenylylsulfate kinase, C-terminal** |
| **SSGZ1_0848** | | | | | - | | + | | - | | + | | | + | | + | | + | | + | | + | | | - | | - | | - | | - | | - | | | + | | - | | - | | - | | - | | - | | | + | | + | | - | | - | | - | | | - | | + | | - | | - | | - | - | **Abortive infection bacteriophage resistance** |
| **SSGZ1_0849** | | | | | - | | + | | - | | + | | | + | | + | | + | | + | | + | | | - | | - | | - | | + | | - | | | + | | + | | - | | - | | - | | + | | | + | | + | | + | | - | | - | | | + | | + | | - | | + | | - | - | **hypothetical protein** |
| **SSGZ1_0850** | | | | | + | | + | | + | | + | | | + | | + | | + | | + | | + | | | + | | + | | + | | + | | + | | | + | | + | | + | | + | | + | | + | | | + | | + | | + | | + | | + | | | + | | + | | + | | + | | + | + | **Phage integrase** |
| **SSGZ1_0851** | | | | | + | | + | | + | | + | | | + | | + | | + | | + | | + | | | + | | + | | + | | + | | + | | | + | | + | | + | | + | | + | | + | | | + | | + | | + | | + | | + | | | + | | + | | + | | + | | + | + | **Phosphoglucomutase** |
| **SSGZ1_0852** | | | | | + | | + | | + | | + | | | + | | + | | + | | + | | + | | | + | | + | | + | | + | | + | | | + | | + | | + | | + | | + | | + | | | + | | + | | + | | + | | + | | | + | | + | | + | | + | | + | + | **ATPase-like protein** |
| **SSGZ1_0853** | | | | | + | | + | | + | | + | | | + | | + | | + | | + | | + | | | + | | + | | + | | + | | + | | | + | | + | | + | | + | | + | | + | | | + | | + | | + | | + | | + | | | + | | + | | + | | + | | + | + | **Transcriptional regulatory protein** |
| **SSGZ1_0854** | | | | | + | | + | | + | | + | | | + | | + | | + | | + | | + | | | + | | + | | + | | + | | + | | | + | | + | | + | | + | | + | | + | | | + | | + | | + | | + | | + | | | + | | + | | + | | + | | + | + | **plasmid addiction system poison protein** |
| **SSGZ1_0855** | | | | | + | | + | | + | | + | | | + | | + | | + | | + | | + | | | + | | + | | + | | + | | + | | | + | | + | | + | | + | | + | | + | | | + | | + | | + | | + | | + | | | + | | + | | + | | + | | + | + | **conserved hypothetical protein** |
| **SSGZ1_0856** | | | | | + | | + | | + | | + | | | + | | + | | + | | + | | + | | | + | | + | | + | | + | | + | | | + | | + | | + | | + | | + | | + | | | + | | + | | + | | + | | + | | | + | | + | | + | | + | | + | + | **putative permease protein** |
| **SSGZ1_0857** | | | | | + | | + | | + | | + | | | + | | + | | + | | + | | + | | | + | | + | | + | | + | | + | | | + | | + | | + | | + | | + | | + | | | + | | + | | + | | + | | + | | | + | | + | | + | | + | | + | + | **putative ABC transporter, ATP-binding protein** |
| **SSGZ1_0858** | | | | | - | | + | | - | | + | | | + | | + | | + | | + | | + | | | - | | - | | - | | - | | - | | | + | | + | | - | | - | | - | | + | | | + | | - | | - | | + | | + | | | + | | + | | + | | - | | + | - | **putative bacteroiocin operon protein ScnG** |
| **SSGZ1_0859** | | | | | - | | + | | - | | + | | | + | | + | | + | | + | | + | | | - | | - | | - | | - | | - | | | + | | - | | - | | - | | - | | - | | | + | | - | | - | | + | | + | | | + | | + | | + | | - | | + | - | **putative bacteriocin operon protein ScnE** |
| **SSGZ1_0860** | | | | | + | | + | | - | | + | | | + | | + | | + | | + | | + | | | - | | - | | - | | - | | - | | | + | | + | | - | | - | | - | | + | | | + | | - | | + | | + | | + | | | + | | + | | + | | + | | + | - | **ABC transporter** |
| **SSGZ1_0861** | | | | | + | | + | | + | | + | | | + | | + | | + | | + | | + | | | + | | + | | + | | + | | + | | | + | | + | | + | | + | | + | | + | | | + | | + | | + | | + | | + | | | + | | + | | + | | + | | + | + | **lipoprotein** |
| **SSGZ1_0862** | | | | | + | | + | | + | | + | | | + | | + | | + | | + | | + | | | + | | + | | + | | + | | + | | | + | | + | | + | | + | | + | | + | | | + | | + | | + | | + | | + | | | + | | + | | + | | + | | + | + | **hypothetical protein** |
| **SSGZ1_0863** | | | | | + | | + | | + | | + | | | + | | + | | + | | + | | + | | | + | | + | | + | | + | | + | | | + | | + | | + | | + | | + | | + | | | + | | - | | + | | + | | + | | | - | | + | | + | | + | | + | + | **ATP-grasp fold** |
| **SSGZ1_0864** | | | | | + | | + | | + | | + | | | + | | + | | + | | + | | + | | | + | | + | | + | | + | | + | | | + | | + | | + | | + | | + | | + | | | + | | + | | + | | + | | + | | | - | | + | | + | | + | | + | + | **Putative esterase** |
| **SSGZ1_0865** | | | | | + | | + | | + | | + | | | + | | + | | + | | + | | + | | | + | | + | | + | | + | | + | | | + | | + | | + | | + | | + | | + | | | + | | + | | + | | + | | + | | | - | | + | | + | | + | | + | + | **Putative esterase** |
| **SSGZ1_0866** | | | | | + | | + | | + | | + | | | + | | + | | + | | + | | + | | | + | | + | | + | | + | | + | | | + | | + | | + | | + | | + | | - | | | + | | + | | + | | + | | + | | | + | | - | | + | | + | | + | + | **hypothetical protein** |
| **SSGZ1_0867** | | | | | + | | + | | - | | + | | | + | | + | | + | | + | | + | | | - | | + | | - | | + | | + | | | - | | + | | - | | + | | + | | - | | | + | | + | | + | | + | | + | | | + | | + | | + | | + | | + | + | **transcription activator** |
| **SSGZ1_0868** | | | | | + | | + | | + | | + | | | + | | + | | + | | + | | + | | | + | | + | | + | | + | | + | | | + | | + | | + | | + | | + | | + | | | + | | + | | + | | + | | + | | | + | | + | | + | | + | | + | + | **Gid protein** |
| **SSGZ1_0869** | | | | | + | | + | | + | | + | | | + | | + | | + | | + | | + | | | + | | + | | + | | + | | + | | | + | | + | | + | | + | | + | | + | | | + | | + | | + | | + | | + | | | + | | + | | + | | + | | + | + | **Haloacid dehalogenase-like hydrolase** |
| **SSGZ1_0870** | | | | | + | | + | | + | | + | | | + | | + | | + | | + | | + | | | + | | + | | + | | + | | + | | | + | | + | | + | | + | | + | | + | | | + | | + | | + | | + | | + | | | + | | + | | + | | + | | + | + | **Ribosomal protein L7/L12** |
| **SSGZ1_0871** | | | | | + | | + | | + | | + | | | + | | + | | + | | + | | + | | | + | | + | | + | | + | | + | | | + | | + | | + | | + | | + | | + | | | + | | + | | + | | + | | + | | | + | | + | | + | | + | | + | + | **Ribosomal protein L10** |
| **SSGZ1_0872** | | | | | + | | + | | + | | + | | | + | | + | | + | | + | | + | | | + | | + | | + | | + | | + | | | + | | + | | + | | + | | + | | + | | | + | | + | | + | | + | | + | | | + | | + | | + | | + | | + | + | **DNA topoisomerase I** |
| **SSGZ1_0873** | | | | | + | | + | | + | | + | | | + | | + | | + | | + | | + | | | + | | + | | + | | + | | + | | | + | | + | | + | | + | | + | | + | | | + | | + | | + | | + | | + | | | + | | + | | + | | + | | + | + | **SMF protein** |
| **SSGZ1_0874** | | | | | + | | + | | + | | + | | | + | | + | | + | | + | | + | | | + | | + | | + | | + | | + | | | + | | + | | + | | + | | + | | + | | | + | | + | | + | | + | | + | | | + | | + | | + | | + | | + | + | **Maltose O-acetyltransferase** |
| **SSGZ1_0875** | | | | | + | | + | | + | | + | | | + | | + | | + | | + | | + | | | + | | + | | + | | + | | + | | | + | | + | | + | | + | | + | | + | | | + | | + | | + | | - | | - | | | + | | + | | - | | + | | + | + | **Protein of unknown function DUF156** |
| **SSGZ1_0876** | | | | | + | | + | | + | | + | | | + | | + | | + | | + | | + | | | + | | + | | + | | + | | + | | | + | | + | | - | | - | | + | | + | | | + | | + | | + | | - | | - | | | - | | + | | - | | - | | + | + | **Rhodanese-like protein** |
| **SSGZ1_0877** | | | | | + | | + | | + | | + | | | + | | + | | + | | + | | + | | | + | | + | | + | | + | | + | | | + | | - | | - | | - | | + | | + | | | + | | + | | - | | - | | - | | | - | | + | | - | | - | | + | + | **coenzyme A disulfide reductase** |
| **SSGZ1_0878** | | | | | + | | + | | + | | + | | | + | | + | | + | | + | | + | | | + | | + | | + | | + | | + | | | + | | + | | + | | + | | + | | + | | | + | | + | | + | | + | | + | | | + | | + | | + | | + | | + | + | **hypothetical protein** |
| **SSGZ1_0879** | | | | | + | | + | | + | | + | | | + | | + | | + | | + | | + | | | + | | + | | + | | + | | + | | | + | | + | | + | | + | | + | | + | | | + | | + | | + | | + | | + | | | + | | + | | + | | + | | + | + | **HylII** |
| **SSGZ1_0880** | | | | | + | | + | | + | | + | | | + | | + | | + | | + | | + | | | + | | + | | + | | + | | + | | | + | | + | | + | | + | | + | | + | | | + | | + | | + | | + | | + | | | + | | + | | + | | + | | + | + | **hypothetical protein** |
| **SSGZ1_0881** | | | | | + | | + | | + | | + | | | + | | + | | + | | + | | + | | | + | | + | | + | | + | | + | | | + | | + | | + | | + | | + | | + | | | + | | + | | + | | + | | + | | | + | | + | | + | | + | | + | + | **Ribonuclease H** |
| **SSGZ1_0882** | | | | | + | | + | | + | | + | | | + | | + | | + | | + | | + | | | + | | + | | + | | + | | + | | | + | | + | | + | | + | | + | | + | | | + | | + | | + | | + | | + | | | + | | + | | + | | + | | + | + | **GTP-binding protein** |
| **SSGZ1_0883** | | | | | + | | + | | + | | + | | | + | | + | | + | | + | | + | | | + | | + | | + | | + | | + | | | + | | + | | + | | + | | + | | + | | | + | | + | | + | | + | | + | | | + | | + | | + | | + | | + | + | **Predicted Fe-S-cluster oxidoreductase** |
| **SSGZ1_0884** | | | | | + | | + | | + | | + | | | + | | + | | + | | + | | + | | | + | | + | | + | | + | | + | | | + | | + | | + | | + | | + | | + | | | + | | + | | + | | + | | + | | | + | | + | | + | | + | | + | + | **Maf-like protein** |
| **SSGZ1_0885** | | | | | + | | + | | + | | + | | | + | | + | | + | | + | | + | | | + | | + | | + | | + | | + | | | + | | + | | + | | + | | + | | + | | | + | | + | | + | | + | | + | | | + | | + | | + | | + | | + | + | **putative 5'-nucleotidase** |
| **SSGZ1_0886** | | | | | + | | + | | + | | + | | | + | | + | | + | | + | | + | | | + | | + | | + | | + | | + | | | + | | + | | + | | + | | + | | + | | | + | | + | | + | | + | | + | | | + | | + | | + | | + | | + | + | **dihydroorotase** |
| **SSGZ1_0887** | | | | | + | | + | | + | | + | | | + | | + | | + | | + | | + | | | + | | + | | + | | + | | + | | | + | | + | | + | | + | | + | | + | | | + | | + | | + | | + | | + | | | + | | + | | + | | + | | + | + | **uracil-DNA glycosylase** |
| **SSGZ1_0888** | | | | | + | | + | | + | | + | | | + | | + | | + | | + | | + | | | + | | + | | + | | + | | + | | | + | | + | | + | | + | | + | | + | | | + | | + | | + | | + | | + | | | + | | + | | + | | + | | + | + | **putative glycosyltransferase** |
| **SSGZ1_0889** | | | | | + | | + | | + | | + | | | + | | + | | + | | + | | + | | | + | | + | | + | | + | | + | | | + | | + | | + | | + | | + | | + | | | + | | + | | + | | + | | + | | | + | | + | | + | | + | | + | + | **orotate phosphoribosyltransferase** |
| **SSGZ1_0890** | | | | | + | | + | | + | | + | | | + | | + | | + | | + | | + | | | + | | + | | + | | + | | + | | | + | | + | | + | | + | | + | | + | | | + | | + | | + | | + | | + | | | + | | + | | + | | + | | + | + | **hypothetical protein** |
| **SSGZ1_0891** | | | | | + | | + | | + | | + | | | + | | + | | + | | + | | + | | | + | | + | | + | | + | | + | | | + | | + | | + | | + | | + | | + | | | + | | + | | + | | + | | + | | | + | | + | | + | | + | | + | + | **orotidine-5-Phosphate decarboxylase** |
| **SSGZ1_0892** | | | | | + | | + | | + | | + | | | + | | + | | + | | + | | + | | | + | | + | | + | | + | | + | | | + | | + | | + | | + | | + | | + | | | + | | + | | + | | + | | + | | | + | | + | | + | | + | | + | + | **putative dihydroorotate dehydrogenase B** |
| **SSGZ1_0893** | | | | | + | | + | | + | | + | | | + | | + | | + | | + | | + | | | + | | + | | + | | + | | + | | | + | | + | | + | | + | | + | | + | | | + | | + | | + | | + | | + | | | + | | + | | + | | + | | + | + | **putative Dihydroorotate dehydrogenase electron** |
| **SSGZ1_0894** | | | | | + | | + | | + | | + | | | + | | + | | + | | + | | + | | | + | | + | | + | | + | | + | | | + | | + | | + | | + | | + | | + | | | + | | + | | + | | + | | + | | | + | | + | | + | | + | | + | + | **capsule expression regulating protein** |
| **SSGZ1_0895** | | | | | + | | + | | + | | + | | | + | | + | | + | | + | | + | | | + | | + | | + | | + | | + | | | + | | + | | + | | + | | + | | + | | | + | | + | | + | | + | | + | | | + | | + | | + | | + | | + | + | **Nucleotidyl transferase** |
| **SSGZ1_0896** | | | | | + | | + | | + | | + | | | + | | + | | + | | + | | + | | | + | | + | | + | | + | | + | | | + | | + | | + | | + | | + | | + | | | + | | + | | + | | + | | + | | | + | | + | | + | | + | | + | + | **Glucose-1-phosphate adenylyltransferase** |
| **SSGZ1_0897** | | | | | + | | + | | + | | + | | | + | | + | | + | | + | | + | | | + | | + | | + | | + | | + | | | + | | + | | + | | + | | + | | + | | | + | | + | | + | | + | | + | | | + | | + | | + | | + | | + | + | **Starch (bacterial glycogen) synthase** |
| **SSGZ1_0898** | | | | | + | | + | | + | | + | | | + | | + | | + | | + | | + | | | + | | + | | + | | + | | + | | | + | | + | | + | | + | | + | | + | | | + | | + | | + | | + | | + | | | + | | + | | + | | + | | + | + | **1,4-alpha-glucan branching enzyme** |
| **SSGZ1_0899** | | | | | + | | + | | + | | + | | | + | | + | | + | | + | | + | | | + | | + | | + | | + | | + | | | + | | + | | + | | + | | + | | + | | | + | | + | | + | | + | | + | | | + | | + | | + | | + | | + | + | **extracellular solute-binding protein, family 3** |
| **SSGZ1_0900** | | | | | + | | + | | + | | + | | | + | | + | | + | | + | | + | | | + | | + | | + | | + | | + | | | + | | + | | + | | + | | + | | - | | | + | | + | | + | | + | | + | | | + | | + | | + | | + | | + | + | **putative amino acid transporter** |
| **SSGZ1_0901** | | | | | + | | + | | + | | + | | | + | | + | | + | | + | | + | | | + | | + | | + | | + | | + | | | + | | + | | + | | + | | + | | + | | | + | | + | | + | | + | | + | | | + | | + | | + | | + | | + | + | **NUDIX hydrolase** |
| **SSGZ1_0902** | | | | | + | | + | | + | | + | | | + | | + | | + | | + | | + | | | + | | + | | + | | + | | + | | | + | | + | | + | | + | | + | | + | | | + | | + | | + | | + | | + | | | + | | + | | + | | + | | + | + | **GCN5-related N-acetyltransferase** |
| **SSGZ1_0903** | | | | | + | | + | | + | | + | | | + | | + | | + | | + | | + | | | + | | - | | + | | + | | - | | | + | | + | | + | | + | | + | | - | | | + | | + | | + | | + | | + | | | + | | + | | + | | + | | + | + | **anchor region containing Surface protein** |
| **SSGZ1_0904** | | | | | - | | + | | - | | + | | | + | | + | | + | | + | | + | | | - | | + | | - | | + | | - | | | + | | + | | + | | + | | - | | + | | | + | | - | | + | | + | | - | | | + | | + | | - | | + | | + | - | **hypothetical protein** |
| **SSGZ1_0905** | | | | | + | | + | | + | | + | | | + | | + | | + | | + | | + | | | + | | + | | + | | + | | + | | | + | | + | | + | | + | | + | | + | | | + | | + | | + | | + | | + | | | + | | + | | + | | + | | + | + | **Excinuclease ABC, B subunit** |
| **SSGZ1_0906** | | | | | + | | + | | + | | + | | | + | | + | | + | | + | | + | | | + | | + | | + | | + | | + | | | + | | + | | + | | + | | + | | + | | | + | | + | | + | | + | | + | | | + | | + | | + | | + | | + | + | **Amino acid ABC transporter,3-TM region** |
| **SSGZ1_0907** | | | | | + | | + | | + | | + | | | + | | + | | + | | + | | + | | | + | | + | | + | | + | | + | | | + | | + | | + | | + | | + | | + | | | + | | + | | + | | + | | + | | | + | | + | | + | | + | | + | + | **Amino acid ABC transporter,3-TM region** |
| **SSGZ1_0908** | | | | | + | | + | | + | | + | | | + | | + | | + | | + | | + | | | + | | + | | + | | + | | + | | | + | | + | | + | | + | | + | | + | | | + | | + | | + | | + | | + | | | + | | + | | + | | + | | + | + | **Phosphate-transporting ATPase** |
| **SSGZ1_0909** | | | | | + | | + | | + | | + | | | + | | + | | + | | + | | + | | | + | | + | | + | | + | | + | | | + | | + | | + | | + | | + | | + | | | + | | + | | + | | + | | + | | | + | | + | | + | | + | | + | + | **GCN5-related N-acetyltransferase** |
| **SSGZ1_0910** | | | | | + | | + | | + | | + | | | + | | + | | + | | + | | + | | | + | | + | | + | | + | | + | | | + | | + | | + | | + | | + | | + | | | + | | - | | + | | + | | + | | | + | | + | | + | | + | | + | + | **ABC transporter, ATP-binding protein** |
| **SSGZ1_0911** | | | | | + | | + | | + | | + | | | + | | + | | + | | + | | + | | | + | | + | | + | | + | | + | | | + | | + | | + | | + | | + | | + | | | + | | + | | + | | + | | + | | | + | | + | | + | | + | | + | + | **O-acetylhomoserine sulfhydrylase** |
| **SSGZ1_0912** | | | | | + | | + | | + | | + | | | + | | + | | + | | + | | + | | | + | | + | | + | | + | | + | | | + | | + | | + | | + | | + | | + | | | + | | + | | + | | + | | - | | | + | | - | | + | | + | | + | + | **hypothetical protein** |
| **SSGZ1_0913** | | | | | + | | + | | + | | + | | | + | | + | | + | | + | | + | | | + | | + | | + | | + | | + | | | + | | + | | + | | + | | + | | + | | | + | | + | | + | | + | | + | | | + | | + | | + | | + | | + | + | **hypothetical protein** |
| **SSGZ1_0914** | | | | | + | | + | | + | | + | | | + | | + | | + | | + | | + | | | + | | + | | + | | + | | + | | | + | | + | | + | | + | | + | | + | | | + | | + | | + | | + | | + | | | + | | + | | + | | + | | + | + | **Aldose 1-epimerase** |
| **SSGZ1_0915** | | | | | + | | + | | + | | + | | | + | | + | | + | | + | | + | | | + | | + | | + | | + | | + | | | + | | + | | + | | + | | + | | + | | | + | | + | | + | | + | | + | | | + | | + | | + | | + | | + | + | **6-phospho-beta-galactosidase** |
| **SSGZ1_0916** | | | | | + | | + | | + | | + | | | + | | + | | + | | + | | + | | | + | | + | | + | | + | | + | | | + | | + | | + | | + | | + | | + | | | + | | + | | + | | + | | + | | | + | | + | | + | | + | | + | + | **PTS system, lactose-specific IIBC component** |
| **SSGZ1_0917** | | | | | + | | + | | + | | + | | | + | | + | | + | | + | | + | | | + | | + | | + | | + | | + | | | + | | + | | + | | + | | + | | + | | | + | | + | | + | | + | | + | | | + | | + | | + | | + | | + | + | **PTS system, lactose-specific IIBC component** |
| **SSGZ1_0918** | | | | | + | | + | | + | | + | | | + | | + | | + | | + | | + | | | + | | + | | + | | + | | + | | | + | | + | | + | | + | | + | | + | | | + | | + | | + | | + | | + | | | + | | + | | + | | + | | + | + | **CAT RNA-binding region containing protein** |
| **SSGZ1_0919** | | | | | + | | + | | + | | + | | | + | | + | | + | | + | | + | | | + | | + | | + | | + | | + | | | + | | + | | + | | + | | + | | + | | | + | | + | | + | | + | | + | | | + | | + | | + | | + | | + | + | **Tagatose 1,6-diphosphate aldolase** |
| **SSGZ1_0920** | | | | | + | | + | | + | | + | | | + | | + | | + | | + | | + | | | + | | + | | + | | + | | + | | | + | | + | | + | | + | | + | | + | | | + | | + | | + | | + | | + | | | + | | + | | + | | + | | + | + | **Tagatose-6-phosphate kinase** |
| **SSGZ1_0921** | | | | | + | | + | | + | | + | | | + | | + | | + | | + | | + | | | + | | + | | + | | + | | + | | | + | | + | | + | | + | | + | | + | | | - | | + | | + | | + | | + | | | + | | + | | + | | + | | + | + | **Tagatose-6-phosphate kinase** |
| **SSGZ1_0922** | | | | | + | | + | | + | | + | | | + | | + | | + | | + | | + | | | + | | + | | + | | + | | + | | | + | | + | | + | | + | | + | | + | | | - | | + | | + | | + | | + | | | + | | + | | + | | + | | + | + | **Ribose/galactose isomerase:** |
| **SSGZ1_0923** | | | | | + | | + | | + | | + | | | + | | + | | + | | + | | + | | | + | | + | | + | | + | | + | | | + | | + | | + | | + | | + | | + | | | + | | + | | + | | + | | + | | | + | | + | | + | | + | | + | + | **Ribose/galactose isomerase:** |
| **SSGZ1_0924** | | | | | + | | + | | + | | + | | | + | | + | | + | | + | | + | | | + | | + | | + | | + | | + | | | + | | + | | + | | + | | + | | + | | | + | | + | | + | | + | | + | | | + | | + | | + | | + | | + | + | **regulatory protein, DeoR** |
| **SSGZ1_0925** | | | | | + | | + | | + | | + | | | + | | + | | + | | + | | + | | | + | | + | | + | | + | | + | | | + | | - | | + | | + | | + | | - | | | + | | + | | + | | + | | - | | | + | | - | | + | | + | | + | + | **hypothetical protein** |
| **SSGZ1_0926** | | | | | + | | + | | + | | + | | | + | | + | | + | | + | | + | | | + | | + | | + | | + | | + | | | + | | + | | + | | + | | + | | + | | | + | | + | | + | | + | | - | | | + | | + | | + | | + | | + | + | **Phage integrase** |
| **SSGZ1_0927** | | | | | + | | + | | + | | + | | | + | | + | | + | | + | | + | | | + | | + | | + | | + | | + | | | + | | + | | + | | + | | + | | - | | | + | | + | | + | | + | | - | | | + | | + | | + | | + | | + | + | **hypothetical protein** |
| **SSGZ1_0928** | | | | | + | | + | | + | | + | | | + | | + | | + | | + | | + | | | + | | + | | + | | + | | + | | | + | | + | | + | | + | | + | | - | | | + | | + | | + | | + | | - | | | + | | + | | + | | + | | + | + | **hypothetical protein** |
| **SSGZ1_0929** | | | | | + | | + | | + | | + | | | + | | + | | + | | + | | + | | | + | | + | | + | | + | | + | | | + | | + | | + | | + | | + | | + | | | + | | + | | + | | + | | + | | | + | | + | | + | | + | | + | + | **Helix-turn-helix motif containing protein** |
| **SSGZ1_0930** | | | | | + | | + | | + | | + | | | + | | + | | + | | + | | + | | | + | | + | | + | | + | | + | | | + | | + | | + | | + | | + | | + | | | + | | + | | + | | + | | + | | | + | | + | | + | | + | | + | + | **A/G-specific adenine glycosylase MutY** |
| **SSGZ1_0931** | | | | | + | | + | | + | | + | | | + | | + | | + | | + | | + | | | + | | + | | + | | + | | + | | | + | | + | | + | | + | | + | | + | | | + | | + | | + | | + | | + | | | + | | + | | + | | + | | + | + | **Phenylacetic acid degradation-related protein** |
| **SSGZ1_0932** | | | | | + | | + | | + | | + | | | + | | + | | + | | + | | + | | | + | | + | | + | | + | | + | | | + | | + | | + | | + | | + | | + | | | + | | + | | + | | + | | + | | | + | | + | | + | | + | | + | + | **ABC transporter related protein** |
| **SSGZ1_0933** | | | | | + | | + | | + | | + | | | + | | + | | + | | + | | + | | | + | | + | | + | | + | | + | | | + | | + | | + | | + | | + | | + | | | + | | + | | + | | + | | + | | | + | | + | | + | | + | | + | + | **ABC transporter related protein** |
| **SSGZ1_0934** | | | | | + | | + | | + | | + | | | + | | + | | + | | + | | + | | | + | | + | | + | | + | | + | | | + | | + | | + | | + | | + | | + | | | + | | + | | + | | + | | + | | | + | | + | | + | | + | | + | + | **Phosphate acetyltransferase** |
| **SSGZ1_0935** | | | | | + | | + | | + | | + | | | + | | + | | + | | + | | + | | | + | | + | | + | | + | | + | | | + | | + | | + | | + | | + | | + | | | + | | + | | + | | + | | + | | | + | | + | | + | | + | | + | + | **Pseudouridine synthase, RluD** |
| **SSGZ1_0936** | | | | | + | | + | | + | | + | | | + | | + | | + | | + | | + | | | + | | + | | + | | + | | + | | | + | | + | | + | | + | | + | | + | | | + | | + | | + | | + | | + | | | + | | + | | + | | + | | + | + | **NAD(+) kinase** |
| **SSGZ1_0937** | | | | | + | | + | | + | | + | | | + | | + | | + | | + | | + | | | + | | + | | + | | + | | + | | | + | | + | | + | | + | | + | | + | | | + | | + | | + | | + | | + | | | + | | + | | + | | + | | + | + | **RelA/SpoT** |
| **SSGZ1_0938** | | | | | + | | + | | + | | + | | | + | | + | | + | | + | | + | | | + | | + | | + | | + | | + | | | + | | + | | + | | + | | + | | + | | | + | | + | | + | | + | | + | | | + | | + | | + | | + | | + | + | **Adenylate cyclase** |
| **SSGZ1_0939** | | | | | + | | + | | + | | + | | | + | | + | | + | | + | | + | | | + | | + | | + | | + | | + | | | + | | + | | + | | + | | + | | + | | | + | | + | | + | | + | | + | | | + | | + | | + | | + | | + | + | **Ribose-phosphate pyrophosphokinase** |
| **SSGZ1_0940** | | | | | + | | + | | + | | + | | | + | | + | | + | | + | | + | | | + | | + | | + | | + | | + | | | + | | + | | + | | + | | + | | + | | | + | | + | | + | | + | | + | | | + | | + | | + | | + | | + | + | **Aminotransferase, class V** |
| **SSGZ1_0941** | | | | | + | | + | | + | | + | | | + | | + | | + | | + | | + | | | + | | + | | + | | + | | + | | | + | | + | | + | | + | | + | | + | | | + | | + | | + | | + | | + | | | + | | + | | + | | + | | + | + | **conserved hypothetical protein** |
| **SSGZ1_0942** | | | | | + | | - | | + | | + | | | + | | + | | + | | + | | + | | | + | | + | | + | | + | | + | | | + | | + | | + | | + | | + | | + | | | + | | + | | + | | + | | + | | | + | | + | | + | | + | | + | + | **hypothetical protein** |
| **SSGZ1_0943** | | | | | + | | + | | + | | + | | | + | | + | | + | | + | | + | | | + | | + | | + | | + | | + | | | + | | + | | + | | + | | + | | + | | | + | | + | | + | | + | | + | | | + | | + | | + | | + | | + | + | **Putative DNA-binding protein** |
| **SSGZ1_0944** | | | | | + | | + | | + | | + | | | + | | + | | + | | + | | + | | | + | | + | | + | | + | | + | | | + | | + | | + | | + | | + | | + | | | + | | + | | + | | + | | + | | | + | | + | | + | | + | | + | + | **Phage integrase** |
| **SSGZ1_0945** | | | | | + | | + | | + | | + | | | + | | + | | + | | + | | + | | | + | | + | | + | | + | | + | | | + | | + | | + | | + | | + | | + | | | + | | + | | + | | + | | + | | | + | | + | | + | | + | | + | + | **putative DNA repair protein** |
| **SSGZ1_0946** | | | | | + | | + | | + | | + | | | + | | + | | + | | + | | + | | | + | | + | | + | | + | | + | | | + | | + | | + | | + | | + | | + | | | + | | + | | + | | + | | + | | | + | | + | | + | | + | | + | + | **sortase** |
| **SSGZ1_0947** | | | | | + | | + | | + | | + | | | + | | + | | + | | + | | + | | | + | | + | | + | | + | | + | | | + | | + | | + | | + | | + | | + | | | + | | + | | + | | + | | + | | | + | | + | | + | | + | | + | + | **DNA gyrase subunit A** |
| **SSGZ1_0948** | | | | | + | | + | | + | | + | | | + | | + | | + | | + | | + | | | + | | + | | + | | + | | + | | | + | | + | | + | | + | | + | | + | | | + | | + | | + | | + | | + | | | + | | + | | + | | + | | + | + | **L-lactate dehydrogenase** |
| **SSGZ1_0949** | | | | | + | | + | | + | | + | | | + | | + | | + | | + | | + | | | + | | + | | + | | + | | + | | | + | | + | | + | | + | | + | | + | | | + | | - | | + | | + | | - | | | + | | + | | + | | + | | + | + | **Dihydrofolate reductase** |
| **SSGZ1_0950** | | | | | + | | + | | + | | + | | | + | | + | | + | | + | | + | | | + | | + | | + | | + | | + | | | + | | + | | + | | + | | + | | + | | | + | | + | | + | | + | | - | | | + | | + | | + | | + | | + | + | **pyrimidine Reductase** |
| **SSGZ1_0951** | | | | | + | | + | | + | | + | | | + | | + | | + | | + | | + | | | + | | + | | + | | + | | + | | | + | | + | | + | | + | | + | | + | | | + | | + | | + | | + | | + | | | + | | + | | + | | + | | + | + | **putative sugar ABC transporter, permease** |
| **SSGZ1_0952** | | | | | + | | + | | + | | + | | | + | | + | | + | | + | | + | | | + | | + | | + | | + | | + | | | + | | + | | + | | + | | + | | + | | | + | | + | | + | | + | | + | | | + | | + | | + | | + | | + | + | **ABC transporter membrane-spanning permease -** |
| **SSGZ1_0953** | | | | | + | | + | | + | | + | | | + | | + | | + | | + | | + | | | + | | + | | + | | + | | + | | | + | | + | | + | | + | | + | | + | | | + | | + | | + | | + | | + | | | + | | + | | + | | + | | + | + | **putative Sugar ABC transporter, ATP-binding** |
| **SSGZ1_0954** | | | | | + | | + | | + | | + | | | + | | + | | + | | + | | + | | | + | | + | | + | | + | | + | | | + | | + | | + | | + | | + | | + | | | + | | + | | + | | + | | + | | | + | | + | | + | | + | | + | + | **Basic membrane lipoprotein** |
| **SSGZ1_0955** | | | | | + | | + | | + | | + | | | + | | + | | + | | + | | + | | | + | | + | | + | | + | | + | | | + | | + | | + | | + | | + | | + | | | + | | + | | + | | + | | + | | | + | | + | | + | | + | | + | + | **Cytidine deaminase** |
| **SSGZ1_0956** | | | | | + | | + | | + | | + | | | + | | + | | + | | + | | + | | | + | | + | | + | | + | | + | | | + | | + | | + | | + | | + | | + | | | + | | + | | + | | + | | + | | | + | | + | | + | | + | | + | + | **Deoxyribose-phosphate aldolase** |
| **SSGZ1_0957** | | | | | + | | + | | + | | + | | | + | | + | | + | | + | | + | | | + | | + | | + | | + | | + | | | + | | + | | + | | + | | + | | + | | | + | | + | | + | | + | | + | | | + | | + | | + | | + | | + | + | **Thymidine phosphorylase** |
| **SSGZ1_0958** | | | | | + | | + | | + | | + | | | + | | + | | + | | + | | + | | | + | | + | | + | | + | | + | | | + | | + | | + | | + | | + | | + | | | + | | + | | + | | + | | + | | | + | | + | | + | | + | | + | + | **Methyltransferase** |
| **SSGZ1_0959** | | | | | + | | + | | + | | + | | | + | | + | | + | | + | | + | | | + | | + | | + | | + | | + | | | + | | + | | + | | + | | + | | + | | | + | | + | | + | | + | | + | | | + | | + | | + | | + | | + | + | **uridine kinase** |
| **SSGZ1_0960** | | | | | + | | + | | + | | + | | | + | | + | | + | | + | | + | | | + | | + | | + | | + | | + | | | + | | + | | + | | + | | + | | + | | | + | | + | | + | | + | | + | | | + | | + | | + | | + | | + | + | **ribosomal protein S20** |
| **SSGZ1_0961** | | | | | + | | + | | + | | + | | | + | | + | | + | | + | | + | | | + | | + | | + | | + | | + | | | + | | + | | + | | + | | + | | + | | | + | | + | | + | | + | | + | | | + | | + | | + | | + | | + | + | **Transglutaminase-like protein** |
| **SSGZ1_0962** | | | | | + | | + | | + | | + | | | + | | + | | + | | + | | + | | | + | | + | | + | | + | | + | | | + | | + | | + | | + | | + | | + | | | + | | + | | + | | + | | + | | | + | | + | | + | | + | | + | + | **Signal transduction histidine kinase** |
| **SSGZ1_0963** | | | | | + | | + | | + | | + | | | + | | + | | + | | + | | + | | | + | | + | | + | | + | | + | | | + | | + | | + | | + | | + | | + | | | + | | + | | + | | + | | + | | | + | | + | | + | | + | | + | + | **Response regulator consisting of a CheY-like** |
| **SSGZ1_0964** | | | | | + | | - | | + | | - | | | + | | + | | + | | - | | + | | | + | | + | | - | | - | | + | | | + | | + | | + | | + | | + | | + | | | - | | + | | + | | + | | + | | | + | | + | | + | | + | | + | + | **hypothetical protein** |
| **SSGZ1_0965** | | | | | + | | + | | + | | + | | | + | | + | | + | | + | | + | | | + | | + | | + | | + | | + | | | + | | + | | + | | + | | + | | + | | | + | | + | | + | | + | | + | | | + | | + | | + | | + | | + | + | **PhoU** |
| **SSGZ1_0966** | | | | | + | | + | | + | | + | | | + | | + | | + | | + | | + | | | + | | + | | + | | + | | + | | | + | | + | | + | | + | | + | | + | | | + | | + | | + | | + | | + | | | + | | + | | + | | + | | + | + | **putative Phosphate import ATP-binding protein** |
| **SSGZ1_0967** | | | | | + | | + | | + | | + | | | + | | + | | + | | + | | + | | | + | | + | | + | | + | | + | | | + | | + | | + | | + | | + | | + | | | + | | + | | + | | + | | + | | | + | | + | | + | | + | | + | + | **ABC transporter ATP-binding protein - phosphate** |
| **SSGZ1_0968** | | | | | + | | + | | + | | + | | | + | | + | | + | | + | | + | | | + | | + | | + | | + | | + | | | + | | + | | + | | + | | + | | + | | | + | | + | | + | | + | | + | | | + | | + | | + | | + | | + | + | **Binding-protein-dependent transport systems** |
| **SSGZ1_0969** | | | | | + | | + | | + | | + | | | + | | + | | + | | + | | + | | | + | | + | | + | | + | | + | | | + | | + | | + | | + | | + | | + | | | + | | + | | + | | + | | + | | | + | | + | | + | | + | | + | + | **Binding-protein-dependent transport systems** |
| **SSGZ1_0970** | | | | | + | | + | | + | | + | | | + | | + | | + | | + | | + | | | + | | + | | + | | + | | + | | | + | | + | | + | | + | | + | | + | | | + | | + | | + | | + | | + | | | + | | + | | + | | + | | + | + | **extracellular solute-binding protein, family 1** |
| **SSGZ1_0971** | | | | | + | | + | | + | | + | | | + | | + | | + | | + | | + | | | + | | + | | + | | + | | + | | | + | | + | | + | | + | | + | | + | | | + | | + | | + | | + | | + | | | + | | + | | + | | + | | + | + | **Putative 23S rRNA m(5)C methyltransferase** |
| **SSGZ1_0972** | | | | | + | | + | | + | | + | | | + | | + | | + | | + | | + | | | + | | + | | + | | + | | + | | | + | | + | | + | | + | | + | | + | | | + | | + | | + | | + | | + | | | + | | + | | + | | + | | + | + | **inositol monophosphatase family related protein** |
| **SSGZ1_0973** | | | | | + | | + | | + | | + | | | + | | + | | + | | + | | + | | | + | | + | | + | | + | | + | | | + | | + | | + | | + | | + | | + | | | + | | + | | + | | + | | + | | | + | | + | | + | | + | | + | + | **hypothetical protein** |
| **SSGZ1_0974** | | | | | + | | + | | + | | + | | | + | | + | | + | | + | | + | | | + | | + | | + | | + | | + | | | + | | + | | + | | + | | + | | + | | | + | | + | | + | | + | | + | | | + | | + | | + | | + | | + | + | **Arsenate reductase and related proteins,** |
| **SSGZ1_0975** | | | | | + | | + | | + | | + | | | + | | + | | + | | + | | + | | | + | | + | | + | | + | | + | | | + | | + | | + | | + | | + | | + | | | + | | + | | + | | + | | + | | | + | | + | | + | | + | | + | + | **Ion transport protein** |
| **SSGZ1_0976** | | | | | + | | + | | + | | + | | | + | | + | | + | | + | | + | | | + | | + | | + | | + | | + | | | + | | + | | + | | + | | + | | + | | | + | | + | | + | | + | | + | | | + | | + | | + | | + | | + | + | **FAD synthetase** |
| **SSGZ1_0977** | | | | | + | | + | | + | | + | | | + | | + | | + | | + | | + | | | + | | + | | + | | + | | + | | | + | | + | | + | | + | | + | | + | | | + | | + | | + | | + | | + | | | + | | + | | + | | + | | + | + | **tRNA pseudouridine synthase B** |
| **SSGZ1_0978** | | | | | + | | + | | + | | + | | | + | | + | | + | | + | | + | | | + | | + | | + | | + | | + | | | + | | + | | + | | + | | + | | + | | | + | | + | | - | | + | | + | | | + | | + | | + | | + | | + | + | **hypothetical protein** |
| **SSGZ1_0979** | | | | | + | | + | | + | | + | | | + | | + | | + | | + | | + | | | + | | + | | + | | + | | + | | | + | | + | | + | | + | | + | | + | | | + | | - | | + | | + | | - | | | + | | + | | + | | + | | + | + | **hypothetical protein** |
| **SSGZ1_0980** | | | | | + | | + | | + | | + | | | + | | + | | + | | + | | + | | | + | | + | | + | | + | | + | | | + | | + | | + | | + | | + | | + | | | + | | + | | + | | + | | + | | | + | | + | | + | | + | | + | + | **hypothetical protein** |
| **SSGZ1_0981** | | | | | + | | + | | + | | + | | | + | | + | | + | | + | | + | | | + | | + | | + | | + | | + | | | + | | + | | + | | + | | + | | + | | | + | | + | | + | | + | | - | | | + | | - | | + | | + | | + | + | **hypothetical protein** |
| **SSGZ1_0982** | | | | | + | | + | | + | | + | | | + | | + | | + | | + | | + | | | + | | + | | + | | + | | + | | | + | | + | | + | | + | | + | | + | | | + | | + | | + | | + | | - | | | + | | - | | + | | + | | + | + | **hypothetical protein** |
| **SSGZ1_0983** | | | | | + | | + | | + | | + | | | + | | + | | + | | + | | + | | | + | | + | | + | | + | | + | | | + | | + | | + | | + | | + | | + | | | + | | + | | + | | + | | - | | | + | | + | | + | | + | | + | + | **GCN5-related N-acetyltransferase** |
| **SSGZ1_0984** | | | | | + | | + | | + | | + | | | + | | + | | + | | + | | + | | | + | | + | | + | | + | | - | | | - | | + | | + | | + | | - | | + | | | + | | + | | - | | + | | + | | | + | | + | | + | | - | | + | + | **IS630-Spn1, transposase Orf1** |
| **SSGZ1_0985** | | | | | + | | + | | + | | + | | | + | | + | | + | | + | | + | | | + | | + | | + | | + | | - | | | - | | - | | - | | - | | - | | - | | | + | | + | | - | | + | | + | | | + | | + | | + | | - | | - | + | **IS630-Spn1, transposase Orf2** |
| **SSGZ1_0986** | | | | | + | | + | | + | | + | | | + | | + | | + | | + | | + | | | + | | + | | + | | + | | + | | | + | | + | | + | | + | | + | | + | | | + | | + | | + | | + | | + | | | + | | + | | + | | + | | + | + | **GCN5-related N-acetyltransferase** |
| **SSGZ1_0987** | | | | | + | | + | | + | | + | | | + | | + | | + | | + | | + | | | + | | + | | + | | + | | + | | | + | | + | | + | | + | | + | | + | | | + | | + | | + | | + | | + | | | + | | + | | + | | + | | + | + | **hypothetical protein** |
| **SSGZ1_0988** | | | | | + | | + | | + | | + | | | + | | + | | + | | + | | + | | | + | | + | | + | | + | | + | | | + | | + | | + | | + | | + | | + | | | + | | + | | + | | + | | + | | | + | | + | | + | | + | | + | + | **hypothetical protein** |
| **SSGZ1_0989** | | | | | + | | + | | + | | + | | | + | | + | | + | | + | | + | | | + | | + | | + | | + | | + | | | + | | + | | + | | + | | + | | + | | | + | | - | | + | | + | | + | | | + | | + | | + | | + | | + | + | **hypothetical protein** |
| **SSGZ1_0990** | | | | | + | | + | | + | | + | | | + | | + | | + | | + | | + | | | + | | + | | + | | + | | + | | | + | | + | | + | | + | | + | | + | | | + | | + | | + | | + | | + | | | + | | + | | + | | + | | + | + | **Cof protein: HAD-superfamily hydrolase,** |
| **SSGZ1_0991** | | | | | + | | + | | + | | + | | | + | | + | | + | | + | | + | | | + | | + | | + | | + | | + | | | + | | + | | + | | + | | + | | + | | | + | | + | | + | | + | | + | | | + | | + | | + | | + | | + | + | **Peptidase M20B, tripeptide aminopeptidase** |
| **SSGZ1_0992** | | | | | + | | + | | + | | + | | | + | | + | | + | | + | | + | | | + | | + | | + | | + | | + | | | + | | + | | + | | + | | + | | + | | | + | | + | | + | | + | | + | | | + | | + | | + | | + | | + | + | **Predicted membrane protein** |
| **SSGZ1_0993** | | | | | + | | + | | + | | + | | | + | | + | | + | | + | | + | | | + | | + | | + | | + | | + | | | + | | + | | + | | + | | + | | + | | | + | | + | | + | | + | | + | | | + | | + | | + | | + | | + | + | **Auxin Efflux Carrier protein** |
| **SSGZ1_0994** | | | | | + | | + | | - | | + | | | + | | + | | + | | + | | + | | | + | | + | | + | | + | | + | | | + | | + | | + | | + | | + | | + | | | - | | + | | + | | + | | + | | | + | | + | | + | | + | | + | + | **Transcriptional regulator PadR-like protein** |
| **SSGZ1_0995** | | | | | + | | + | | - | | + | | | + | | + | | + | | + | | + | | | + | | + | | + | | + | | + | | | + | | + | | + | | + | | + | | + | | | - | | + | | + | | + | | + | | | + | | + | | + | | + | | + | + | **membrane like protein** |
| **SSGZ1_0996** | | | | | + | | + | | + | | + | | | + | | + | | + | | + | | + | | | + | | + | | - | | - | | + | | | + | | + | | + | | + | | + | | + | | | + | | - | | + | | + | | + | | | + | | + | | + | | + | | + | + | **transposase of IS200 family** |
| **SSGZ1_0997** | | | | | + | | + | | + | | + | | | + | | + | | + | | + | | + | | | + | | + | | + | | + | | + | | | + | | + | | + | | + | | + | | + | | | + | | + | | + | | + | | + | | | + | | + | | + | | + | | + | + | **Predicted membrane protein** |
| **SSGZ1_0998** | | | | | + | | + | | - | | + | | | + | | + | | + | | + | | + | | | + | | + | | + | | + | | + | | | + | | + | | + | | + | | - | | + | | | - | | + | | + | | + | | + | | | + | | + | | + | | + | | + | + | **regulatory protein, MarR** |
| **SSGZ1_0999** | | | | | + | | + | | + | | + | | | + | | + | | + | | + | | + | | | + | | + | | + | | + | | + | | | + | | + | | + | | + | | - | | + | | | + | | + | | + | | + | | + | | | + | | + | | + | | + | | + | + | **hypothetical protein** |
| **SSGZ1_1000** | | | | | + | | + | | - | | + | | | + | | + | | + | | + | | + | | | + | | + | | + | | + | | + | | | + | | + | | + | | + | | - | | + | | | - | | + | | + | | + | | + | | | + | | + | | + | | + | | + | + | **hypothetical protein** |
| **SSGZ1_1001** | | | | | + | | + | | + | | + | | | + | | + | | + | | + | | + | | | + | | + | | + | | + | | + | | | + | | + | | + | | + | | - | | + | | | + | | + | | + | | + | | + | | | + | | + | | + | | + | | + | + | **putative cation efflux transporter** |
| **SSGZ1_1002** | | | | | + | | + | | - | | + | | | + | | + | | + | | + | | + | | | + | | + | | + | | + | | + | | | + | | + | | + | | + | | + | | + | | | - | | + | | + | | + | | + | | | + | | + | | + | | + | | + | + | **putative permease** |
| **SSGZ1_1003** | | | | | + | | + | | - | | + | | | + | | + | | + | | + | | + | | | + | | + | | + | | + | | + | | | + | | + | | + | | + | | + | | - | | | + | | + | | + | | + | | + | | | + | | + | | + | | + | | + | + | **putative** |
| **SSGZ1_1004** | | | | | + | | + | | + | | + | | | + | | + | | + | | + | | + | | | + | | + | | + | | + | | + | | | + | | + | | + | | + | | + | | - | | | + | | + | | + | | + | | + | | | + | | + | | + | | + | | + | + | **dihydroneopterin aldolase** |
| **SSGZ1_1005** | | | | | + | | + | | - | | + | | | + | | + | | + | | + | | + | | | + | | + | | + | | + | | + | | | + | | + | | + | | + | | + | | - | | | - | | + | | + | | + | | + | | | + | | + | | + | | + | | + | + | **dihydropteroate synthase** |
| **SSGZ1_1006** | | | | | + | | + | | + | | + | | | + | | + | | + | | + | | + | | | + | | + | | + | | + | | + | | | + | | + | | + | | + | | + | | + | | | + | | + | | + | | + | | + | | | + | | + | | + | | + | | + | + | **GTP cyclohydrolase I** |
| **SSGZ1_1007** | | | | | + | | + | | - | | + | | | + | | + | | + | | + | | + | | | + | | + | | + | | + | | + | | | + | | + | | + | | + | | + | | + | | | - | | + | | + | | + | | + | | | + | | + | | + | | + | | + | + | **NUDIX hydrolase** |
| **SSGZ1_1008** | | | | | + | | + | | + | | + | | | + | | + | | + | | + | | + | | | + | | + | | + | | + | | + | | | + | | + | | + | | + | | + | | + | | | + | | + | | + | | + | | + | | | + | | + | | + | | + | | + | + | **hypothetical protein** |
| **SSGZ1_1009** | | | | | + | | + | | + | | + | | | + | | + | | + | | + | | + | | | + | | + | | + | | + | | + | | | + | | + | | + | | + | | + | | + | | | + | | + | | + | | + | | + | | | + | | + | | + | | + | | + | + | **SPX domain-containing protein involved in** |
| **SSGZ1_1010** | | | | | + | | + | | + | | + | | | + | | + | | + | | + | | + | | | + | | + | | + | | + | | + | | | + | | + | | + | | + | | + | | + | | | + | | + | | + | | + | | + | | | + | | + | | + | | + | | + | + | **similar to unknown protein** |
| **SSGZ1_1011** | | | | | + | | + | | + | | + | | | + | | + | | + | | + | | + | | | + | | + | | + | | + | | + | | | + | | + | | + | | + | | + | | + | | | + | | + | | + | | + | | + | | | + | | + | | + | | + | | + | + | **hypothetical protein** |
| **SSGZ1_1012** | | | | | + | | + | | + | | + | | | + | | + | | + | | + | | + | | | + | | + | | + | | + | | + | | | + | | + | | + | | + | | + | | + | | | + | | + | | + | | + | | + | | | + | | + | | + | | + | | + | + | **phenylalanyl-tRNA synthetase, beta subunit** |
| **SSGZ1_1013** | | | | | + | | + | | + | | + | | | + | | + | | + | | + | | + | | | + | | + | | + | | + | | - | | | + | | + | | + | | + | | + | | + | | | + | | - | | - | | + | | + | | | + | | + | | + | | + | | - | + | **GCN5-related N-acetyltransferase** |
| **SSGZ1_1014** | | | | | + | | + | | + | | + | | | + | | + | | + | | + | | + | | | + | | + | | + | | + | | + | | | + | | + | | + | | + | | + | | + | | | + | | + | | + | | + | | + | | | + | | + | | + | | + | | + | + | **Phenylalanyl-tRNA synthetase, alpha subunit** |
| **SSGZ1_1015** | | | | | + | | + | | + | | + | | | + | | + | | + | | + | | + | | | + | | + | | + | | + | | + | | | + | | - | | + | | + | | + | | + | | | + | | + | | + | | + | | + | | | + | | + | | + | | + | | - | + | **Glycoside hydrolase, family 3** |
| **SSGZ1_1016** | | | | | + | | + | | + | | + | | | + | | + | | + | | + | | + | | | + | | + | | + | | + | | + | | | + | | - | | + | | + | | + | | + | | | + | | + | | + | | + | | + | | | + | | + | | + | | + | | - | + | **glycosyl hydrolase, family 3** |
| **SSGZ1_1017** | | | | | + | | + | | + | | + | | | + | | + | | + | | + | | + | | | + | | + | | + | | + | | + | | | + | | - | | + | | + | | + | | + | | | + | | + | | + | | + | | + | | | + | | + | | + | | + | | - | + | **Predicted phosphatase** |
| **SSGZ1_1018** | | | | | + | | + | | + | | + | | | + | | + | | + | | + | | + | | | + | | + | | + | | + | | + | | | + | | - | | + | | + | | + | | + | | | + | | - | | + | | + | | + | | | + | | + | | + | | + | | - | + | **reductase SDR** |
| **SSGZ1_1019** | | | | | + | | + | | + | | + | | | + | | + | | + | | + | | + | | | + | | + | | + | | + | | + | | | + | | - | | + | | + | | + | | + | | | + | | + | | + | | + | | + | | | + | | + | | + | | + | | - | + | **Mannonate dehydratase** |
| **SSGZ1_1020** | | | | | + | | + | | + | | + | | | + | | + | | + | | + | | + | | | + | | + | | + | | + | | + | | | + | | - | | + | | + | | + | | + | | | + | | + | | + | | + | | + | | | + | | + | | + | | + | | - | + | **Glucuronate isomerase** |
| **SSGZ1_1021** | | | | | + | | + | | + | | + | | | + | | + | | + | | + | | + | | | + | | + | | + | | + | | + | | | + | | - | | + | | + | | + | | + | | | + | | + | | + | | + | | + | | | + | | + | | + | | + | | - | + | **KDPG and KHG aldolase** |
| **SSGZ1_1022** | | | | | + | | + | | + | | + | | | + | | + | | + | | + | | + | | | + | | + | | + | | + | | + | | | + | | - | | + | | + | | + | | + | | | + | | + | | + | | + | | + | | | + | | + | | + | | + | | - | + | **regulatory protein, GntR** |
| **SSGZ1_1023** | | | | | + | | + | | + | | + | | | + | | + | | + | | + | | + | | | + | | + | | + | | + | | + | | | + | | - | | + | | + | | + | | + | | | + | | + | | + | | + | | + | | | + | | + | | + | | + | | - | + | **Beta-glucuronidase** |
| **SSGZ1_1024** | | | | | + | | + | | + | | + | | | + | | + | | + | | + | | + | | | + | | + | | + | | + | | + | | | + | | - | | + | | + | | + | | + | | | + | | + | | + | | + | | + | | | + | | + | | + | | + | | - | + | **Carbohydrate kinase, PfkB** |
| **SSGZ1_1025** | | | | | + | | + | | + | | + | | | + | | + | | + | | + | | + | | | + | | + | | + | | + | | + | | | + | | - | | + | | + | | + | | + | | | + | | + | | + | | + | | + | | | + | | + | | + | | + | | - | + | **sugar/sodium symporter** |
| **SSGZ1_1026** | | | | | + | | + | | + | | + | | | + | | + | | + | | + | | + | | | + | | + | | + | | + | | + | | | + | | + | | + | | + | | + | | + | | | + | | + | | + | | + | | + | | | + | | + | | + | | + | | + | + | **DNA-entry nuclease** |
| **SSGZ1_1027** | | | | | + | | + | | + | | + | | | + | | + | | + | | + | | + | | | + | | + | | + | | + | | + | | | + | | + | | + | | + | | + | | + | | | + | | + | | + | | + | | + | | | + | | + | | + | | + | | + | + | **conserved hypothetical protein** |
| **SSGZ1_1028** | | | | | + | | + | | + | | + | | | + | | + | | + | | + | | + | | | + | | + | | + | | + | | + | | | - | | + | | + | | + | | + | | + | | | + | | + | | + | | + | | + | | | + | | + | | + | | + | | + | + | **UDP-N-acetylglucosamine** |
| **SSGZ1_1029** | | | | | + | | + | | + | | + | | | + | | + | | + | | + | | + | | | + | | + | | + | | + | | + | | | + | | + | | + | | + | | + | | + | | | + | | + | | + | | + | | + | | | + | | + | | + | | + | | + | + | **hypothetical membrane associated protein** |
| **SSGZ1_1030** | | | | | + | | + | | + | | + | | | + | | + | | + | | + | | + | | | + | | + | | + | | + | | + | | | + | | + | | + | | + | | + | | + | | | + | | + | | + | | + | | + | | | + | | + | | + | | + | | + | + | **H+-transporting two-sector ATPase, delta/epsilon** |
| **SSGZ1_1031** | | | | | + | | + | | + | | + | | | + | | + | | + | | + | | + | | | + | | + | | + | | + | | + | | | + | | + | | + | | + | | + | | + | | | + | | + | | + | | + | | + | | | + | | + | | + | | + | | + | + | **ATP synthase F1, beta subunit** |
| **SSGZ1_1032** | | | | | + | | + | | + | | + | | | + | | + | | + | | + | | + | | | + | | + | | + | | + | | + | | | + | | + | | + | | + | | + | | + | | | + | | + | | + | | + | | + | | | + | | + | | + | | + | | + | + | **H+-transporting two-sector ATPase, gamma** |
| **SSGZ1_1033** | | | | | + | | + | | + | | + | | | + | | + | | + | | + | | + | | | + | | + | | + | | + | | + | | | + | | + | | + | | + | | + | | + | | | + | | + | | + | | + | | + | | | + | | + | | + | | + | | + | + | **ATP synthase F1, alpha subunit** |
| **SSGZ1_1034** | | | | | + | | + | | + | | + | | | + | | + | | + | | + | | + | | | + | | + | | + | | + | | + | | | + | | + | | + | | + | | + | | + | | | + | | + | | + | | + | | + | | | + | | + | | + | | + | | + | + | **H+-transporting two-sector ATPase, delta (OSCP)** |
| **SSGZ1_1035** | | | | | + | | + | | + | | + | | | + | | + | | + | | + | | + | | | + | | + | | + | | + | | + | | | + | | + | | + | | + | | + | | + | | | + | | + | | + | | + | | + | | | + | | + | | + | | + | | + | + | **ATP synthase F0, subunit B** |
| **SSGZ1_1036** | | | | | + | | + | | + | | + | | | + | | + | | + | | + | | + | | | + | | + | | + | | + | | + | | | + | | + | | + | | + | | + | | + | | | + | | + | | + | | + | | + | | | + | | + | | + | | + | | + | + | **H+-transporting two-sector ATPase, A subunit** |
| **SSGZ1_1037** | | | | | + | | + | | + | | + | | | + | | + | | + | | + | | + | | | + | | + | | + | | + | | + | | | + | | + | | + | | + | | + | | + | | | + | | + | | + | | + | | + | | | + | | + | | + | | + | | + | + | **H+-transporting two-sector ATPase, C subunit** |
| **SSGZ1_1038** | | | | | + | | + | | + | | + | | | + | | + | | + | | + | | + | | | + | | + | | + | | + | | + | | | + | | + | | + | | + | | + | | + | | | + | | + | | + | | + | | + | | | + | | + | | + | | + | | + | + | **Sphingosine kinase and enzymes related to** |
| **SSGZ1_1039** | | | | | + | | + | | + | | + | | | + | | + | | + | | + | | + | | | + | | + | | + | | + | | + | | | + | | + | | + | | + | | + | | + | | | + | | + | | + | | + | | + | | | + | | + | | + | | + | | + | + | **NAD-dependent DNA ligase** |
| **SSGZ1_1040** | | | | | + | | + | | + | | + | | | + | | + | | + | | + | | + | | | + | | + | | + | | + | | + | | | + | | + | | + | | + | | + | | + | | | + | | + | | + | | + | | + | | | + | | + | | + | | + | | + | + | **hypothetical protein** |
| **SSGZ1_1041** | | | | | + | | + | | + | | + | | | + | | + | | + | | + | | + | | | + | | + | | + | | + | | + | | | + | | + | | + | | + | | + | | + | | | + | | + | | + | | + | | + | | | + | | + | | + | | + | | + | + | **permease** |
| **SSGZ1_1042** | | | | | + | | + | | + | | + | | | + | | + | | + | | + | | + | | | + | | + | | + | | + | | + | | | + | | + | | + | | + | | + | | + | | | + | | + | | + | | + | | + | | | + | | + | | + | | + | | + | + | **Predicted membrane protein** |
| **SSGZ1_1043** | | | | | + | | + | | + | | + | | | + | | + | | + | | + | | + | | | + | | + | | + | | + | | + | | | + | | + | | + | | + | | + | | + | | | + | | + | | + | | + | | + | | | + | | + | | + | | + | | + | + | **Glucose-6-phosphate dehydrogenase** |
| **SSGZ1_1044** | | | | | + | | + | | + | | + | | | + | | + | | + | | + | | + | | | + | | + | | + | | + | | + | | | + | | + | | + | | + | | + | | + | | | + | | + | | + | | + | | + | | | + | | + | | + | | + | | + | + | **hypothetical protein** |
| **SSGZ1_1045** | | | | | + | | + | | + | | + | | | + | | + | | + | | + | | + | | | - | | + | | - | | - | | + | | | + | | + | | + | | + | | + | | + | | | + | | + | | + | | + | | - | | | + | | + | | + | | + | | + | + | **GCN5-related N-acetyltransferase** |
| **SSGZ1_1046** | | | | | + | | + | | + | | + | | | + | | + | | + | | + | | + | | | + | | + | | + | | + | | + | | | + | | + | | + | | + | | + | | + | | | + | | + | | + | | + | | + | | | + | | + | | + | | + | | + | + | **Cell division transporter substrate-binding** |
| **SSGZ1_1047** | | | | | + | | + | | + | | + | | | + | | + | | + | | + | | + | | | + | | + | | + | | + | | + | | | + | | + | | + | | + | | + | | + | | | + | | + | | + | | + | | + | | | + | | + | | + | | + | | + | + | **Cof protein: HAD-superfamily hydrolase,** |
| **SSGZ1_1048** | | | | | + | | + | | + | | + | | | + | | + | | + | | + | | + | | | + | | + | | + | | + | | + | | | + | | + | | + | | + | | + | | + | | | + | | + | | + | | + | | + | | | + | | + | | + | | + | | + | + | **HAD-superfamily hydrolase, subfamily IIB** |
| **SSGZ1_1049** | | | | | + | | + | | + | | + | | | + | | + | | + | | + | | + | | | + | | + | | + | | + | | + | | | + | | + | | + | | + | | + | | + | | | + | | + | | + | | + | | + | | | + | | + | | + | | + | | + | + | **SMC protein** |
| **SSGZ1_1050** | | | | | + | | + | | + | | + | | | + | | + | | + | | + | | + | | | + | | + | | + | | + | | + | | | + | | + | | + | | + | | + | | + | | | + | | + | | + | | + | | + | | | + | | + | | + | | + | | + | + | **Ribonuclease III** |
| **SSGZ1_1051** | | | | | + | | + | | + | | + | | | + | | + | | + | | + | | + | | | + | | + | | + | | + | | + | | | + | | + | | + | | + | | - | | + | | | + | | + | | + | | + | | + | | | + | | + | | + | | + | | + | + | **putative transmembrane protein** |
| **SSGZ1_1052** | | | | | + | | + | | + | | + | | | + | | + | | + | | + | | + | | | + | | + | | + | | + | | + | | | + | | + | | + | | + | | + | | + | | | + | | - | | + | | + | | + | | | + | | + | | + | | + | | + | + | **regulatory protein, ArsR** |
| **SSGZ1_1053** | | | | | + | | + | | + | | + | | | + | | + | | + | | + | | + | | | - | | + | | + | | + | | + | | | + | | + | | + | | + | | + | | + | | | + | | + | | + | | + | | + | | | + | | + | | + | | + | | + | + | **lantibiotic efflux protein** |
| **SSGZ1_1054** | | | | | - | | + | | + | | + | | | + | | + | | + | | + | | + | | | - | | - | | - | | - | | - | | | + | | - | | - | | + | | + | | + | | | + | | + | | - | | + | | + | | | + | | + | | + | | - | | + | - | **transposase, IS30 family, putative, truncation** |
| **SSGZ1_1055** | | | | | + | | + | | + | | + | | | + | | + | | + | | + | | + | | | + | | + | | + | | + | | + | | | + | | + | | + | | + | | + | | + | | | + | | + | | + | | + | | + | | | + | | + | | + | | + | | + | + | **hypothetical protein** |
| **SSGZ1_1056** | | | | | + | | + | | + | | + | | | + | | + | | + | | + | | + | | | + | | + | | + | | + | | + | | | + | | + | | + | | + | | + | | + | | | + | | + | | + | | + | | + | | | + | | + | | + | | + | | + | + | **Phosphoenolpyruvate-protein phosphotransferase** |
| **SSGZ1_1057** | | | | | + | | + | | + | | + | | | + | | + | | + | | + | | + | | | + | | + | | + | | + | | + | | | + | | + | | + | | + | | + | | + | | | + | | + | | + | | + | | + | | | + | | + | | + | | + | | + | + | **Phosphocarrier HPr protein** |
| **SSGZ1_1058** | | | | | + | | + | | + | | + | | | + | | + | | + | | + | | + | | | + | | + | | + | | + | | + | | | + | | + | | + | | + | | + | | + | | | + | | + | | + | | + | | + | | | + | | + | | + | | + | | + | + | **isopropylmalate dehydrogenase** |
| **SSGZ1_1059** | | | | | + | | + | | + | | + | | | + | | + | | + | | + | | + | | | + | | + | | + | | + | | + | | | + | | + | | + | | + | | + | | + | | | + | | + | | + | | + | | + | | | + | | + | | + | | + | | + | + | **2-methylcitrate synthase** |
| **SSGZ1_1060** | | | | | + | | + | | + | | + | | | + | | + | | + | | + | | + | | | + | | + | | + | | + | | + | | | + | | + | | + | | + | | + | | + | | | + | | + | | + | | + | | + | | | + | | + | | + | | + | | + | + | **Aconitate hydratase 1** |
| **SSGZ1_1061** | | | | | + | | + | | + | | + | | | + | | + | | + | | + | | + | | | + | | + | | + | | + | | + | | | + | | + | | + | | + | | + | | + | | | + | | + | | + | | + | | + | | | + | | + | | + | | + | | + | + | **NrdH-redoxin** |
| **SSGZ1_1062** | | | | | + | | + | | + | | + | | | + | | + | | + | | + | | + | | | + | | + | | + | | + | | + | | | + | | + | | + | | + | | + | | + | | | + | | + | | + | | + | | + | | | + | | + | | + | | + | | + | + | **ribonucleotide-diphosphate reductase alpha** |
| **SSGZ1_1063** | | | | | + | | + | | + | | + | | | + | | + | | + | | + | | + | | | + | | + | | + | | + | | + | | | + | | + | | + | | + | | + | | + | | | + | | - | | + | | + | | + | | | + | | + | | + | | + | | + | + | **hypothetical protein** |
| **SSGZ1_1064** | | | | | + | | + | | + | | + | | | + | | + | | + | | + | | + | | | + | | + | | + | | + | | + | | | + | | + | | + | | + | | + | | + | | | + | | + | | + | | + | | + | | | + | | + | | + | | + | | + | + | **ribonucleotide-diphosphate reductase beta** |
| **SSGZ1_1065** | | | | | + | | + | | + | | + | | | + | | + | | + | | + | | + | | | + | | + | | + | | + | | + | | | + | | + | | + | | + | | + | | + | | | + | | + | | + | | + | | + | | | + | | + | | + | | + | | + | + | **hypothetical protein** |
| **SSGZ1_1066** | | | | | + | | + | | + | | + | | | + | | + | | + | | + | | + | | | + | | + | | + | | + | | + | | | + | | - | | - | | + | | + | | + | | | + | | + | | - | | - | | - | | | - | | - | | - | | - | | - | + | **LacI transcriptional regulator** |
| **SSGZ1_1067** | | | | | + | | + | | + | | + | | | + | | + | | + | | + | | + | | | + | | + | | + | | + | | - | | | + | | - | | - | | + | | + | | - | | | + | | + | | - | | - | | - | | | - | | - | | - | | - | | - | + | **hypothetical protein** |
| **SSGZ1_1068** | | | | | + | | + | | + | | + | | | + | | + | | + | | + | | + | | | + | | + | | + | | + | | - | | | + | | - | | - | | + | | + | | + | | | + | | + | | - | | - | | - | | | - | | + | | - | | - | | - | + | **hyaluronidase, truncated** |
| **SSGZ1_1069** | | | | | + | | + | | + | | + | | | + | | + | | + | | + | | + | | | + | | + | | + | | + | | - | | | + | | - | | - | | + | | + | | - | | | + | | + | | - | | - | | - | | | - | | - | | - | | - | | - | + | **hyaluronidase, truncated** |
| **SSGZ1_1070** | | | | | + | | + | | + | | + | | | + | | + | | + | | + | | + | | | + | | + | | + | | + | | - | | | + | | - | | - | | + | | + | | - | | | + | | + | | - | | - | | - | | | - | | - | | - | | - | | - | + | **hyaluronidase, truncated** |
| **SSGZ1_1071** | | | | | + | | + | | + | | + | | | + | | + | | + | | + | | + | | | + | | + | | + | | + | | - | | | + | | - | | - | | + | | + | | - | | | + | | + | | - | | - | | - | | | - | | - | | - | | - | | - | + | **hyaluronidase, truncated** |
| **SSGZ1_1072** | | | | | + | | + | | + | | + | | | + | | + | | + | | + | | + | | | + | | + | | + | | + | | - | | | + | | - | | - | | + | | + | | - | | | + | | + | | - | | - | | - | | | - | | - | | - | | - | | - | + | **preprotein translocase YajC subunit** |
| **SSGZ1_1073** | | | | | + | | + | | + | | + | | | + | | + | | + | | + | | + | | | + | | + | | + | | + | | - | | | + | | - | | - | | + | | + | | - | | | + | | + | | - | | - | | - | | | - | | - | | - | | - | | - | + | **PTS system component protein** |
| **SSGZ1_1074** | | | | | + | | + | | + | | + | | | + | | + | | + | | + | | + | | | + | | + | | + | | + | | - | | | + | | - | | - | | + | | + | | - | | | + | | + | | - | | - | | - | | | - | | - | | - | | - | | - | + | **sugar-specific EII component protein** |
| **SSGZ1_1075** | | | | | + | | + | | + | | + | | | + | | + | | + | | + | | + | | | + | | + | | + | | + | | - | | | + | | - | | - | | + | | + | | - | | | + | | + | | - | | - | | - | | | - | | - | | - | | - | | - | + | **PTS system sorbose subfamily IIB component** |
| **SSGZ1_1076** | | | | | + | | + | | + | | + | | | + | | + | | + | | + | | + | | | + | | + | | + | | + | | - | | | + | | - | | - | | + | | + | | - | | | + | | + | | - | | - | | - | | | - | | - | | - | | - | | - | + | **Glycosyl hydrolase, family 88** |
| **SSGZ1_1077** | | | | | + | | + | | + | | + | | | + | | + | | + | | + | | + | | | + | | + | | + | | + | | - | | | + | | - | | - | | + | | + | | - | | | + | | + | | - | | - | | - | | | - | | - | | - | | - | | - | + | **sugar-specific EII component protein** |
| **SSGZ1_1078** | | | | | + | | + | | + | | + | | | + | | + | | + | | + | | + | | | + | | + | | + | | + | | - | | | + | | - | | - | | + | | + | | - | | | + | | + | | - | | - | | - | | | - | | - | | - | | - | | - | + | **KDPG aldolase** |
| **SSGZ1_1079** | | | | | + | | + | | + | | + | | | + | | + | | + | | + | | + | | | + | | + | | + | | + | | - | | | + | | - | | - | | + | | + | | - | | | + | | + | | - | | - | | - | | | - | | - | | - | | - | | - | + | **Carbohydrate kinase, PfkB** |
| **SSGZ1_1080** | | | | | + | | + | | + | | + | | | + | | + | | + | | + | | + | | | + | | + | | + | | + | | - | | | + | | - | | - | | + | | + | | - | | | + | | + | | - | | - | | - | | | - | | - | | - | | - | | - | + | **Putative ribose 5-phosphate isomerase** |
| **SSGZ1_1081** | | | | | + | | + | | + | | + | | | + | | + | | + | | + | | + | | | + | | + | | + | | + | | - | | | + | | - | | - | | + | | + | | - | | | + | | + | | - | | - | | - | | | - | | - | | - | | - | | - | + | **oxidoreductase** |
| **SSGZ1_1082** | | | | | + | | + | | + | | + | | | + | | + | | + | | + | | + | | | - | | + | | + | | + | | - | | | + | | - | | - | | + | | + | | - | | | + | | + | | - | | + | | + | | | + | | + | | - | | - | | + | + | **transposase** |
| **SSGZ1_1083** | | | | | + | | + | | + | | + | | | + | | + | | + | | + | | + | | | + | | + | | + | | + | | + | | | - | | + | | + | | + | | + | | + | | | + | | - | | + | | + | | + | | | + | | + | | + | | + | | + | + | **Predicted metal-dependent membrane protease** |
| **SSGZ1_1084** | | | | | + | | + | | + | | + | | | + | | + | | + | | + | | + | | | + | | + | | + | | + | | + | | | + | | + | | + | | + | | + | | + | | | + | | - | | + | | + | | + | | | + | | + | | + | | + | | + | + | **Hypothetical protein** |
| **SSGZ1_1085** | | | | | + | | + | | + | | + | | | + | | + | | + | | + | | + | | | + | | + | | + | | + | | + | | | + | | + | | + | | + | | + | | + | | | + | | + | | + | | + | | + | | | + | | + | | + | | + | | + | + | **hypothetical protein** |
| **SSGZ1_1086** | | | | | + | | + | | + | | + | | | + | | + | | + | | + | | + | | | + | | + | | + | | + | | + | | | + | | + | | + | | + | | + | | + | | | + | | + | | + | | + | | + | | | + | | + | | + | | + | | + | + | **hypothetical protein** |
| **SSGZ1_1087** | | | | | + | | + | | + | | + | | | + | | + | | + | | + | | + | | | + | | + | | + | | + | | + | | | + | | + | | + | | + | | + | | + | | | + | | + | | + | | + | | + | | | + | | + | | + | | + | | + | + | **Hypothetical protein** |
| **SSGZ1_1088** | | | | | + | | + | | + | | + | | | + | | + | | + | | + | | + | | | + | | + | | + | | + | | + | | | + | | + | | + | | + | | + | | + | | | + | | - | | + | | + | | + | | | + | | + | | + | | + | | + | + | **Transcriptional regulator, Cro/CI family** |
| **SSGZ1_1089** | | | | | + | | + | | + | | + | | | + | | + | | + | | + | | + | | | + | | + | | + | | + | | + | | | + | | + | | + | | + | | + | | + | | | + | | + | | + | | + | | + | | | + | | - | | + | | + | | + | + | **putative regulator protein** |
| **SSGZ1_1090** | | | | | + | | + | | + | | + | | | + | | + | | + | | + | | + | | | + | | + | | + | | + | | + | | | + | | + | | + | | + | | + | | + | | | + | | + | | + | | + | | + | | | + | | + | | + | | + | | + | + | **hypothetical protein** |
| **SSGZ1_1091** | | | | | - | | + | | + | | + | | | + | | + | | + | | + | | + | | | + | | - | | - | | - | | - | | | + | | + | | - | | - | | - | | + | | | + | | + | | - | | + | | - | | | - | | - | | + | | - | | - | + | **hypothetical protein** |
| **SSGZ1_1092** | | | | | - | | + | | + | | + | | | + | | + | | + | | + | | + | | | + | | - | | - | | - | | - | | | + | | + | | - | | - | | - | | + | | | + | | + | | - | | - | | - | | | - | | - | | - | | - | | - | + | **hypothetical protein** |
| **SSGZ1_1093** | | | | | + | | + | | + | | + | | | + | | + | | + | | + | | + | | | + | | + | | + | | + | | + | | | + | | + | | + | | + | | + | | + | | | + | | + | | + | | + | | + | | | + | | + | | + | | + | | + | + | **Alanyl-tRNA synthetase, class IIc** |
| **SSGZ1_1094** | | | | | + | | + | | + | | + | | | + | | + | | + | | + | | + | | | + | | + | | + | | + | | + | | | + | | + | | + | | + | | + | | + | | | + | | + | | + | | + | | + | | | + | | + | | + | | + | | + | + | **Alanyl-tRNA synthetase, class IIc** |
| **SSGZ1_1095** | | | | | + | | + | | + | | + | | | + | | + | | + | | + | | + | | | + | | + | | + | | + | | + | | | + | | + | | + | | + | | + | | + | | | + | | + | | + | | + | | + | | | + | | + | | + | | + | | + | + | **unknown function protein** |
| **SSGZ1_1096** | | | | | + | | + | | + | | + | | | + | | + | | + | | + | | + | | | + | | + | | + | | + | | + | | | + | | + | | + | | + | | + | | + | | | + | | + | | + | | + | | + | | | + | | + | | + | | + | | + | + | **PpiC-type peptidyl-prolyl cis-trans isomerase** |
| **SSGZ1_1097** | | | | | - | | + | | + | | + | | | + | | + | | + | | + | | + | | | - | | - | | - | | - | | - | | | + | | - | | - | | + | | + | | + | | | + | | + | | - | | + | | + | | | + | | + | | + | | - | | + | - | **transposase, IS30 family, putative** |
| **SSGZ1_1098** | | | | | + | | + | | + | | + | | | + | | + | | + | | + | | + | | | + | | + | | + | | + | | + | | | + | | + | | + | | + | | + | | + | | | + | | + | | + | | + | | + | | | + | | + | | + | | + | | + | + | **O-methyltransferase, family 3** |
| **SSGZ1_1099** | | | | | + | | + | | + | | + | | | + | | + | | + | | + | | + | | | + | | + | | + | | + | | + | | | + | | + | | + | | + | | + | | + | | | + | | + | | + | | + | | + | | | + | | + | | + | | + | | + | + | **hypothetical protein** |
| **SSGZ1_1100** | | | | | + | | + | | + | | + | | | + | | + | | + | | + | | + | | | + | | + | | + | | + | | + | | | + | | + | | + | | + | | + | | + | | | + | | + | | + | | + | | + | | | + | | + | | + | | + | | + | + | **hypothetical protein** |
| **SSGZ1_1101** | | | | | + | | + | | + | | + | | | + | | + | | + | | + | | + | | | + | | + | | + | | + | | + | | | + | | + | | + | | + | | + | | + | | | + | | + | | + | | + | | + | | | + | | + | | + | | + | | + | + | **oligoendopeptidase F** |
| **SSGZ1_1102** | | | | | + | | + | | + | | + | | | + | | + | | + | | + | | + | | | + | | + | | + | | + | | + | | | + | | + | | + | | + | | + | | + | | | + | | + | | + | | + | | + | | | + | | + | | + | | + | | + | + | **Competence CoiA-like protein** |
| **SSGZ1_1103** | | | | | + | | + | | + | | + | | | + | | + | | + | | + | | + | | | + | | + | | + | | + | | + | | | + | | + | | + | | + | | + | | + | | | + | | + | | + | | + | | + | | | + | | + | | + | | + | | + | + | **Methionyl-tRNA synthetase, class Ia** |
| **SSGZ1_1104** | | | | | + | | + | | + | | + | | | + | | + | | + | | + | | + | | | + | | + | | + | | + | | + | | | + | | + | | + | | + | | + | | + | | | + | | + | | + | | + | | + | | | + | | + | | + | | + | | + | + | **Predicted membrane protein** |
| **SSGZ1_1105** | | | | | + | | + | | + | | + | | | + | | + | | + | | + | | + | | | + | | + | | + | | + | | + | | | + | | + | | + | | + | | + | | + | | | + | | + | | + | | + | | + | | | + | | + | | + | | + | | + | + | **Phospho-2-dehydro-3-deoxyheptonate aldolase,** |
| **SSGZ1_1106** | | | | | + | | + | | + | | + | | | + | | + | | + | | + | | + | | | + | | + | | + | | + | | + | | | + | | + | | + | | + | | + | | + | | | + | | + | | + | | + | | + | | | + | | + | | + | | + | | + | + | **3-deoxy-7-phosphoheptulonate synthase** |
| **SSGZ1_1107** | | | | | + | | + | | + | | + | | | + | | + | | + | | + | | + | | | + | | + | | + | | + | | + | | | + | | + | | + | | + | | + | | + | | | + | | + | | + | | + | | + | | | + | | + | | + | | + | | + | + | **Shikimate 5-dehydrogenase** |
| **SSGZ1_1108** | | | | | + | | + | | + | | + | | | + | | + | | + | | + | | + | | | + | | + | | + | | + | | + | | | + | | + | | + | | + | | + | | + | | | + | | + | | + | | + | | + | | | + | | + | | + | | + | | + | + | **3-dehydroquinate synthase** |
| **SSGZ1_1109** | | | | | + | | + | | + | | + | | | + | | + | | + | | + | | + | | | + | | + | | + | | + | | + | | | + | | + | | + | | + | | + | | + | | | + | | + | | + | | + | | + | | | + | | + | | + | | + | | + | + | **ABC transporter, ATP binding protein** |
| **SSGZ1_1110** | | | | | + | | + | | + | | + | | | + | | + | | + | | + | | + | | | + | | + | | + | | + | | + | | | + | | + | | + | | + | | + | | + | | | + | | + | | + | | + | | + | | | + | | + | | + | | + | | + | + | **inner-membrane translocator** |
| **SSGZ1_1111** | | | | | + | | + | | + | | + | | | + | | + | | + | | + | | + | | | + | | + | | + | | + | | + | | | + | | + | | + | | + | | + | | + | | | + | | + | | + | | + | | + | | | + | | + | | + | | + | | + | + | **substrate-binding protein** |
| **SSGZ1_1112** | | | | | + | | + | | + | | + | | | + | | + | | + | | + | | + | | | + | | + | | + | | + | | + | | | + | | + | | + | | + | | + | | + | | | + | | + | | + | | + | | + | | | + | | + | | + | | + | | + | + | **ABC transporter, permease protein** |
| **SSGZ1_1113** | | | | | + | | + | | + | | + | | | + | | + | | + | | + | | + | | | + | | + | | + | | + | | + | | | + | | + | | + | | + | | + | | + | | | + | | + | | + | | + | | + | | | + | | + | | + | | + | | + | + | **Tagatose 1,6-diphosphate aldolase 2** |
| **SSGZ1_1114** | | | | | + | | + | | + | | + | | | + | | + | | + | | + | | + | | | + | | + | | + | | + | | + | | | + | | + | | + | | + | | + | | + | | | + | | + | | + | | + | | + | | | + | | + | | + | | + | | + | + | **N-acetylglucosamine-6-phosphate deacetylase** |
| **SSGZ1_1115** | | | | | + | | + | | + | | + | | | + | | + | | + | | + | | + | | | + | | + | | + | | + | | + | | | + | | + | | + | | + | | + | | + | | | + | | + | | + | | + | | + | | | + | | + | | + | | + | | + | + | **Protein of unknown function DUF964** |
| **SSGZ1_1116** | | | | | + | | + | | + | | + | | | + | | + | | + | | + | | + | | | + | | + | | + | | + | | + | | | + | | + | | + | | + | | + | | + | | | + | | + | | + | | + | | + | | | + | | + | | + | | + | | + | + | **Prephenate dehydrogenase** |
| **SSGZ1_1117** | | | | | + | | + | | + | | + | | | + | | + | | + | | + | | + | | | + | | + | | + | | + | | + | | | + | | + | | + | | + | | + | | + | | | + | | + | | + | | + | | + | | | + | | + | | + | | + | | + | + | **Chorismate synthase** |
| **SSGZ1_1118** | | | | | + | | + | | + | | + | | | + | | + | | + | | + | | + | | | + | | + | | + | | + | | + | | | + | | + | | + | | + | | + | | + | | | + | | + | | + | | + | | + | | | + | | + | | + | | + | | + | + | **Dehydroquinase class I** |
| **SSGZ1_1119** | | | | | + | | + | | + | | + | | | + | | + | | + | | + | | + | | | + | | + | | + | | + | | + | | | + | | + | | + | | + | | + | | + | | | + | | + | | + | | + | | + | | | + | | + | | + | | + | | + | + | **hypothetical protein** |
| **SSGZ1_1120** | | | | | + | | + | | + | | + | | | + | | + | | + | | + | | + | | | + | | + | | + | | + | | + | | | + | | + | | + | | + | | + | | + | | | + | | + | | + | | + | | + | | | + | | + | | + | | + | | + | + | **Sulfatase** |
| **SSGZ1_1121** | | | | | + | | + | | + | | + | | | + | | + | | + | | + | | + | | | + | | + | | + | | + | | + | | | + | | + | | + | | + | | + | | + | | | + | | + | | + | | + | | + | | | + | | + | | + | | + | | + | + | **Streptococcal histidine triad** |
| **SSGZ1_1122** | | | | | + | | + | | + | | + | | | + | | + | | + | | + | | + | | | + | | + | | + | | + | | + | | | + | | + | | + | | + | | + | | + | | | + | | + | | + | | + | | + | | | + | | + | | + | | + | | + | + | **ribosomal protein L20** |
| **SSGZ1_1123** | | | | | + | | + | | + | | + | | | + | | + | | + | | + | | + | | | + | | + | | + | | + | | + | | | + | | + | | + | | + | | + | | + | | | + | | + | | + | | + | | + | | | + | | + | | + | | + | | + | + | **50S ribosomal protein L35** |
| **SSGZ1_1124** | | | | | + | | + | | + | | + | | | + | | + | | + | | + | | + | | | + | | + | | + | | + | | + | | | + | | + | | + | | + | | + | | + | | | + | | + | | + | | + | | + | | | + | | + | | + | | + | | + | + | **translation initiation factor IF-3** |
| **SSGZ1_1125** | | | | | + | | + | | + | | + | | | + | | + | | + | | + | | + | | | + | | + | | + | | + | | + | | | + | | + | | + | | + | | + | | + | | | + | | + | | + | | + | | + | | | + | | + | | + | | + | | + | + | **Cytidylate kinase** |
| **SSGZ1_1126** | | | | | + | | + | | + | | + | | | + | | + | | + | | + | | + | | | + | | + | | + | | + | | + | | | + | | + | | + | | + | | + | | + | | | + | | + | | + | | + | | + | | | + | | + | | + | | + | | + | + | **conserved hypothetical protein** |
| **SSGZ1_1127** | | | | | + | | + | | + | | + | | | + | | + | | + | | + | | + | | | + | | + | | + | | + | | + | | | + | | + | | + | | + | | + | | + | | | + | | + | | + | | + | | + | | | + | | + | | + | | + | | + | + | **Ferredoxin** |
| **SSGZ1_1128** | | | | | + | | + | | + | | + | | | + | | + | | + | | + | | + | | | + | | + | | + | | + | | + | | | + | | + | | + | | + | | + | | + | | | + | | + | | + | | + | | + | | | + | | + | | + | | + | | + | + | **conserved hypothetical protein** |
| **SSGZ1_1129** | | | | | + | | + | | + | | + | | | + | | + | | + | | + | | + | | | + | | + | | + | | + | | + | | | + | | + | | + | | + | | + | | + | | | + | | + | | + | | + | | + | | | + | | + | | + | | + | | + | + | **Glycosyl transferase, family 2** |
| **SSGZ1_1130** | | | | | + | | + | | + | | + | | | + | | + | | + | | + | | + | | | + | | + | | + | | + | | + | | | + | | + | | + | | + | | + | | + | | | + | | + | | + | | + | | + | | | + | | + | | + | | + | | + | + | **conserved hypothetical protein** |
| **SSGZ1_1131** | | | | | + | | + | | + | | + | | | + | | + | | + | | + | | + | | | + | | + | | + | | + | | + | | | + | | + | | + | | + | | + | | + | | | + | | + | | + | | + | | + | | | + | | + | | + | | + | | + | + | **Glycosyl transferase, family 2** |
| **SSGZ1_1132** | | | | | + | | + | | + | | + | | | + | | + | | + | | + | | + | | | + | | + | | + | | + | | + | | | + | | + | | + | | - | | + | | + | | | + | | + | | + | | + | | + | | | + | | + | | + | | + | | + | + | **Glycosyl transferase, group 1** |
| **SSGZ1_1133** | | | | | + | | + | | + | | + | | | + | | + | | + | | + | | + | | | + | | + | | + | | + | | + | | | + | | + | | + | | - | | + | | + | | | + | | + | | + | | + | | + | | | + | | + | | + | | + | | + | + | **Glycosyl transferase, group 1** |
| **SSGZ1_1134** | | | | | - | | + | | + | | + | | | + | | + | | + | | + | | + | | | + | | + | | + | | + | | + | | | - | | + | | + | | - | | + | | + | | | + | | - | | + | | + | | - | | | + | | + | | + | | + | | + | + | **Glycosyl transferase, family 2** |
| **SSGZ1_1135** | | | | | + | | + | | + | | + | | | + | | + | | + | | + | | + | | | + | | + | | + | | + | | + | | | + | | + | | + | | - | | + | | - | | | + | | + | | + | | + | | + | | | + | | + | | + | | + | | + | + | **Glycosyl transferase, family 8** |
| **SSGZ1_1136** | | | | | + | | + | | + | | + | | | + | | + | | + | | + | | + | | | + | | + | | + | | + | | + | | | + | | + | | + | | - | | + | | - | | | + | | + | | + | | + | | + | | | + | | + | | + | | + | | + | + | **similar to membrane protein** |
| **SSGZ1_1137** | | | | | + | | + | | + | | + | | | + | | + | | + | | + | | + | | | + | | + | | + | | + | | + | | | + | | + | | + | | - | | + | | + | | | + | | + | | + | | + | | + | | | + | | + | | + | | + | | + | + | **polysaccharide/teichoic acid transporter,** |
| **SSGZ1_1138** | | | | | + | | + | | + | | + | | | + | | + | | + | | + | | + | | | + | | + | | + | | + | | + | | | + | | + | | + | | + | | + | | + | | | + | | + | | + | | + | | + | | | + | | + | | + | | + | | + | + | **polysaccharide biosynthesis protein** |
| **SSGZ1_1139** | | | | | + | | + | | + | | + | | | + | | + | | + | | + | | + | | | + | | + | | + | | + | | + | | | + | | + | | + | | - | | + | | - | | | - | | + | | + | | + | | + | | | + | | + | | + | | + | | + | + | **polysaccharide biosynthesis protein** |
| **SSGZ1_1140** | | | | | + | | + | | + | | + | | | + | | + | | + | | + | | + | | | + | | + | | + | | + | | + | | | + | | + | | + | | - | | + | | - | | | - | | + | | + | | + | | + | | | + | | + | | + | | + | | + | + | **putative ABC-type lipopolysaccharide transport** |
| **SSGZ1_1141** | | | | | + | | + | | + | | + | | | + | | + | | + | | + | | + | | | + | | + | | + | | + | | + | | | + | | + | | + | | - | | + | | - | | | + | | - | | + | | + | | + | | | + | | + | | + | | + | | + | + | **polysaccharide ABC exporter membrane-spanning** |
| **SSGZ1_1142** | | | | | + | | + | | + | | + | | | + | | + | | + | | + | | + | | | + | | + | | + | | + | | + | | | + | | + | | + | | + | | + | | + | | | + | | - | | + | | + | | + | | | + | | + | | + | | + | | + | + | **putative Polysaccharide biosynthesis protein/** |
| **SSGZ1_1143** | | | | | + | | + | | + | | + | | | + | | + | | + | | + | | + | | | + | | + | | + | | + | | + | | | + | | + | | + | | + | | + | | + | | | + | | + | | + | | + | | + | | | + | | + | | + | | + | | + | + | **Glycosyltransferase** |
| **SSGZ1_1144** | | | | | + | | + | | + | | + | | | + | | + | | + | | + | | + | | | + | | + | | + | | + | | + | | | + | | + | | + | | - | | + | | + | | | + | | + | | + | | + | | + | | | + | | + | | + | | + | | + | + | **similar to Phosphoglycerol transferase and** |
| **SSGZ1_1145** | | | | | + | | + | | + | | + | | | + | | + | | + | | + | | + | | | + | | + | | + | | + | | + | | | + | | + | | + | | + | | + | | + | | | + | | + | | + | | + | | + | | | + | | + | | + | | + | | + | + | **Mannosyl-glycoprotein** |
| **SSGZ1_1146** | | | | | + | | + | | + | | + | | | + | | + | | + | | + | | + | | | + | | + | | + | | + | | + | | | + | | + | | + | | - | | + | | + | | | + | | + | | + | | + | | + | | | + | | + | | + | | + | | + | + | **Cell wall hydrolase/autolysin** |
| **SSGZ1_1147** | | | | | + | | + | | + | | + | | | + | | + | | + | | + | | + | | | + | | + | | + | | + | | + | | | + | | + | | + | | - | | + | | + | | | + | | + | | + | | + | | + | | | + | | - | | + | | + | | + | + | **Surface protein from Gram-positive cocci, anchor** |
| **SSGZ1_1148** | | | | | + | | + | | + | | + | | | + | | + | | + | | + | | + | | | + | | + | | + | | + | | + | | | + | | + | | + | | + | | + | | + | | | + | | + | | + | | + | | + | | | + | | + | | + | | + | | + | + | **dTDP-4-dehydrorhamnose reductase** |
| **SSGZ1_1149** | | | | | + | | + | | + | | + | | | + | | + | | + | | + | | + | | | + | | + | | + | | + | | + | | | + | | + | | + | | + | | + | | + | | | + | | - | | + | | + | | + | | | + | | + | | + | | + | | + | + | **dTDP-glucose 4,6-dehydratase** |
| **SSGZ1_1150** | | | | | + | | + | | + | | + | | | + | | + | | + | | + | | + | | | + | | + | | + | | + | | + | | | + | | + | | + | | + | | + | | + | | | + | | + | | + | | + | | + | | | + | | + | | + | | + | | + | + | **putative phosphoribosylanthranilate isomerase** |
| **SSGZ1_1151** | | | | | + | | + | | + | | + | | | + | | + | | + | | + | | + | | | + | | + | | + | | + | | + | | | + | | + | | + | | + | | + | | + | | | + | | - | | + | | + | | + | | | + | | + | | + | | + | | + | + | **dTDP-4-dehydrorhamnose 3,5-epimerase** |
| **SSGZ1_1152** | | | | | + | | + | | + | | + | | | + | | + | | + | | + | | + | | | + | | + | | + | | + | | + | | | + | | + | | + | | + | | + | | + | | | + | | + | | + | | + | | + | | | + | | + | | + | | + | | + | + | **glucose-1-phosphate thymidylyltransferase** |
| **SSGZ1_1153** | | | | | + | | + | | + | | + | | | + | | + | | + | | + | | + | | | + | | + | | + | | + | | + | | | + | | + | | + | | + | | + | | + | | | + | | + | | + | | + | | + | | | + | | + | | + | | + | | + | + | **Zinc transporter ZIP** |
| **SSGZ1_1154** | | | | | + | | + | | + | | + | | | + | | + | | + | | + | | + | | | + | | + | | + | | + | | + | | | + | | + | | + | | + | | + | | + | | | + | | + | | + | | + | | + | | | + | | + | | + | | + | | + | + | **hypothetical protein** |
| **SSGZ1_1155** | | | | | + | | + | | + | | + | | | + | | + | | + | | + | | + | | | + | | + | | + | | + | | + | | | + | | + | | + | | + | | + | | + | | | + | | + | | + | | + | | + | | | + | | + | | + | | + | | + | + | **Predicted SAM-dependent methyltransferase** |
| **SSGZ1_1156** | | | | | + | | + | | + | | + | | | + | | + | | + | | + | | + | | | + | | + | | + | | + | | + | | | + | | + | | + | | + | | + | | + | | | + | | + | | + | | + | | + | | | + | | + | | + | | + | | + | + | **DNA replication protein dnaD** |
| **SSGZ1_1157** | | | | | + | | + | | + | | + | | | + | | + | | + | | + | | + | | | + | | + | | + | | + | | + | | | + | | + | | + | | + | | + | | + | | | + | | + | | + | | + | | + | | | + | | + | | + | | + | | + | + | **Homoserine O-succinyltransferase** |
| **SSGZ1_1158** | | | | | + | | + | | + | | + | | | + | | + | | + | | + | | + | | | + | | + | | + | | + | | + | | | + | | + | | + | | + | | + | | + | | | + | | + | | + | | + | | + | | | + | | + | | + | | + | | + | + | **Adenine phosphoribosyl transferase** |
| **SSGZ1_1159** | | | | | + | | + | | + | | + | | | + | | + | | + | | + | | + | | | + | | + | | + | | + | | + | | | + | | + | | + | | + | | + | | + | | | + | | + | | + | | + | | + | | | + | | + | | + | | + | | + | + | **hypothetical protein** |
| **SSGZ1_1160** | | | | | + | | + | | + | | + | | | + | | + | | + | | + | | + | | | + | | + | | + | | + | | + | | | + | | + | | + | | + | | + | | + | | | + | | + | | + | | + | | + | | | + | | + | | + | | + | | + | + | **RecJ exonuclease** |
| **SSGZ1_1161** | | | | | + | | + | | + | | + | | | + | | + | | + | | + | | + | | | + | | + | | + | | + | | + | | | + | | + | | + | | + | | + | | + | | | + | | + | | + | | + | | + | | | + | | + | | + | | + | | + | + | **Phospholipase D/Transphosphatidylase** |
| **SSGZ1_1162** | | | | | + | | + | | + | | + | | | + | | + | | + | | + | | + | | | + | | + | | + | | + | | + | | | + | | - | | + | | + | | + | | - | | | + | | + | | + | | + | | + | | | + | | - | | + | | + | | + | + | **hypothetical protein** |
| **SSGZ1_1163** | | | | | + | | + | | + | | + | | | + | | + | | + | | + | | + | | | + | | + | | + | | + | | + | | | + | | + | | + | | + | | + | | + | | | + | | + | | + | | + | | + | | | + | | + | | + | | + | | + | + | **Short-chain dehydrogenase/reductase SDR** |
| **SSGZ1_1164** | | | | | + | | + | | + | | + | | | + | | + | | + | | + | | + | | | + | | + | | + | | + | | + | | | + | | + | | + | | + | | + | | + | | | + | | + | | + | | + | | + | | | + | | + | | + | | + | | + | + | **Beta-lactamase-like protein** |
| **SSGZ1_1165** | | | | | + | | + | | + | | + | | | + | | + | | + | | + | | + | | | + | | + | | + | | + | | + | | | + | | + | | + | | + | | + | | + | | | + | | + | | + | | + | | + | | | + | | + | | + | | + | | + | + | **HrpA-like helicase** |
| **SSGZ1_1166** | | | | | + | | + | | + | | + | | | + | | + | | + | | + | | + | | | + | | + | | + | | + | | + | | | + | | + | | + | | + | | + | | + | | | + | | - | | + | | + | | + | | | + | | + | | + | | + | | + | + | **putative GTP-binding protein** |
| **SSGZ1_1167** | | | | | + | | + | | + | | + | | | + | | + | | + | | + | | + | | | + | | + | | + | | + | | + | | | + | | + | | + | | + | | + | | + | | | + | | + | | + | | + | | + | | | + | | + | | + | | + | | + | + | **tRNA isopentenyltransferase** |
| **SSGZ1_1168** | | | | | + | | + | | + | | + | | | + | | + | | + | | + | | + | | | + | | + | | + | | + | | + | | | + | | + | | + | | + | | + | | + | | | + | | + | | + | | + | | + | | | + | | + | | + | | + | | + | + | **hypothetical protein** |
| **SSGZ1_1169** | | | | | + | | + | | + | | + | | | + | | + | | + | | + | | + | | | + | | + | | + | | + | | + | | | + | | + | | + | | + | | + | | + | | | + | | + | | + | | + | | + | | | + | | + | | + | | + | | + | + | **Transporter, putative** |
| **SSGZ1_1170** | | | | | + | | + | | + | | + | | | + | | + | | + | | + | | + | | | + | | + | | + | | + | | + | | | + | | + | | + | | + | | + | | + | | | + | | + | | + | | + | | + | | | + | | + | | + | | + | | + | + | **hypothetical protein** |
| **SSGZ1_1171** | | | | | + | | + | | + | | + | | | + | | + | | + | | + | | + | | | + | | + | | + | | + | | + | | | + | | + | | + | | + | | + | | + | | | + | | + | | + | | + | | + | | | + | | + | | + | | + | | + | + | **PhoH-like protein** |
| **SSGZ1_1172** | | | | | + | | + | | + | | + | | | + | | + | | + | | + | | + | | | + | | + | | + | | + | | + | | | + | | + | | + | | + | | + | | + | | | + | | + | | + | | + | | + | | | + | | + | | + | | + | | + | + | **Protein of unknown function DUF1250** |
| **SSGZ1_1173** | | | | | + | | + | | + | | + | | | + | | + | | + | | + | | + | | | + | | + | | + | | + | | + | | | + | | + | | + | | + | | + | | + | | | + | | + | | + | | + | | + | | | + | | + | | + | | + | | + | + | **hypothetical protein** |
| **SSGZ1_1174** | | | | | + | | + | | + | | + | | | + | | + | | + | | + | | + | | | + | | + | | + | | + | | + | | | + | | + | | + | | + | | + | | + | | | + | | + | | + | | + | | + | | | + | | + | | + | | + | | + | + | **Ketopantoate reductase ApbA/PanE** |
| **SSGZ1_1175** | | | | | + | | + | | + | | + | | | + | | + | | + | | + | | + | | | + | | + | | + | | + | | + | | | + | | + | | + | | + | | + | | + | | | + | | + | | + | | + | | + | | | + | | + | | + | | + | | + | + | **hypothetical protein** |
| **SSGZ1_1176** | | | | | + | | + | | + | | + | | | + | | + | | + | | + | | + | | | + | | + | | + | | + | | + | | | + | | + | | + | | + | | + | | + | | | + | | + | | + | | + | | + | | | + | | + | | + | | + | | + | + | **Ribosome recycling factor** |
| **SSGZ1_1177** | | | | | + | | + | | + | | + | | | + | | + | | + | | + | | + | | | + | | + | | + | | + | | + | | | + | | + | | + | | + | | + | | + | | | + | | + | | + | | + | | + | | | + | | + | | + | | + | | + | + | **uridylate kinase** |
| **SSGZ1_1178** | | | | | + | | + | | + | | + | | | + | | + | | + | | + | | + | | | + | | + | | + | | + | | + | | | + | | + | | + | | + | | + | | + | | | + | | + | | + | | + | | + | | | + | | + | | + | | + | | + | + | **Polysaccharide biosynthesis protein** |
| **SSGZ1_1179** | | | | | + | | + | | + | | + | | | + | | + | | + | | + | | + | | | + | | + | | + | | + | | + | | | + | | + | | + | | + | | + | | + | | | + | | + | | + | | + | | + | | | + | | + | | + | | + | | + | + | **putative glutathione S-transferase** |
| **SSGZ1_1180** | | | | | + | | + | | + | | + | | | + | | + | | + | | + | | + | | | + | | + | | + | | + | | + | | | + | | + | | + | | + | | + | | + | | | + | | + | | + | | + | | + | | | + | | + | | + | | + | | + | + | **hypothetical protein** |
| **SSGZ1_1181** | | | | | + | | + | | + | | + | | | + | | + | | + | | + | | + | | | + | | + | | + | | + | | + | | | + | | + | | + | | + | | + | | + | | | + | | + | | + | | + | | + | | | + | | + | | + | | + | | + | + | **ribosomal protein L1** |
| **SSGZ1_1182** | | | | | + | | + | | + | | + | | | + | | + | | + | | + | | + | | | + | | + | | + | | + | | + | | | + | | + | | + | | + | | + | | + | | | + | | + | | + | | + | | + | | | + | | + | | + | | + | | + | + | **ribosomal protein L11** |
| **SSGZ1_1183** | | | | | + | | + | | + | | + | | | + | | + | | + | | + | | + | | | + | | + | | + | | + | | + | | | + | | + | | + | | + | | + | | + | | | + | | + | | + | | + | | + | | | + | | + | | + | | + | | + | + | **hypothetical protein** |
| **SSGZ1_1184** | | | | | + | | + | | + | | + | | | + | | + | | + | | + | | + | | | + | | + | | + | | + | | + | | | + | | + | | + | | + | | + | | + | | | + | | + | | + | | + | | + | | | + | | + | | + | | + | | + | + | **putative lactoylglutathione lyase** |
| **SSGZ1_1185** | | | | | + | | + | | + | | + | | | + | | + | | + | | + | | + | | | + | | + | | + | | + | | + | | | + | | + | | + | | + | | + | | + | | | + | | + | | + | | + | | + | | | + | | + | | + | | + | | + | + | **Cell division protein FtsK** |
| **SSGZ1_1186** | | | | | + | | + | | + | | + | | | + | | + | | + | | + | | + | | | + | | + | | + | | + | | + | | | + | | - | | + | | + | | + | | + | | | + | | + | | + | | + | | + | | | + | | + | | + | | + | | + | + | **Beta-fructofuranosidase** |
| **SSGZ1_1187** | | | | | + | | + | | + | | + | | | + | | + | | + | | + | | + | | | + | | + | | + | | + | | + | | | + | | - | | + | | + | | + | | + | | | + | | + | | + | | + | | + | | | + | | + | | + | | + | | + | + | **extracellular solute-binding protein, family 1** |
| **SSGZ1_1188** | | | | | + | | + | | + | | + | | | + | | + | | + | | + | | + | | | + | | + | | + | | + | | + | | | + | | - | | + | | + | | + | | + | | | + | | + | | + | | + | | + | | | + | | + | | + | | + | | + | + | **Binding-protein-dependent transport systems** |
| **SSGZ1_1189** | | | | | + | | + | | + | | + | | | + | | + | | + | | + | | + | | | + | | + | | + | | + | | + | | | + | | - | | + | | + | | + | | + | | | + | | + | | + | | + | | + | | | + | | + | | + | | + | | + | + | **Binding-protein-dependent transport systems** |
| **SSGZ1_1190** | | | | | + | | + | | + | | + | | | + | | + | | + | | + | | + | | | + | | + | | + | | + | | + | | | + | | - | | + | | + | | + | | + | | | + | | + | | + | | + | | + | | | + | | + | | + | | + | | + | + | **regulatory protein, LacI** |
| **SSGZ1_1191** | | | | | + | | + | | + | | + | | | + | | + | | + | | + | | + | | | + | | + | | + | | + | | + | | | + | | + | | + | | + | | + | | + | | | + | | + | | + | | + | | + | | | + | | + | | + | | + | | + | + | **hypothetical protein** |
| **SSGZ1_1192** | | | | | + | | + | | + | | + | | | + | | + | | + | | + | | + | | | + | | + | | + | | + | | + | | | + | | + | | + | | + | | + | | + | | | + | | + | | + | | + | | + | | | + | | + | | + | | + | | + | + | **hypothetical protein** |
| **SSGZ1_1193** | | | | | + | | + | | + | | + | | | + | | + | | + | | + | | + | | | + | | + | | + | | + | | + | | | + | | + | | + | | + | | + | | + | | | + | | + | | + | | + | | + | | | + | | + | | + | | + | | + | + | **Glyoxalase/bleomycin resistance** |
| **SSGZ1_1194** | | | | | + | | + | | + | | + | | | + | | + | | + | | + | | + | | | + | | + | | + | | + | | + | | | + | | + | | + | | + | | + | | + | | | + | | + | | + | | + | | + | | | + | | + | | + | | + | | + | + | **hypothetical protein** |
| **SSGZ1_1195** | | | | | + | | + | | + | | + | | | + | | + | | + | | + | | + | | | + | | + | | + | | + | | + | | | + | | + | | + | | + | | + | | + | | | + | | + | | + | | + | | + | | | + | | + | | + | | + | | + | + | **Peptidylprolyl isomerase** |
| **SSGZ1_1196** | | | | | + | | + | | + | | + | | | + | | + | | + | | + | | + | | | - | | + | | - | | - | | + | | | + | | + | | + | | - | | - | | - | | | + | | + | | + | | + | | + | | | + | | + | | - | | + | | - | + | **Transposase, IS204/IS1001/IS1096/IS1165** |
| **SSGZ1_1197** | | | | | + | | + | | + | | + | | | + | | + | | + | | + | | + | | | + | | + | | + | | + | | + | | | + | | + | | + | | + | | + | | + | | | + | | + | | + | | + | | + | | | + | | + | | + | | + | | + | + | **Peptide chain release factor 3** |
| **SSGZ1_1198** | | | | | + | | + | | + | | + | | | + | | + | | + | | + | | + | | | + | | + | | + | | + | | + | | | + | | + | | + | | + | | + | | + | | | + | | + | | + | | + | | + | | | + | | + | | + | | + | | + | + | **Haloacid dehalogenase-like hydrolase** |
| **SSGZ1_1199** | | | | | + | | + | | + | | + | | | + | | + | | + | | + | | + | | | + | | + | | + | | + | | + | | | + | | + | | + | | + | | + | | + | | | + | | + | | + | | + | | + | | | + | | + | | + | | + | | + | + | **hypothetical protein** |
| **SSGZ1_1200** | | | | | + | | + | | + | | + | | | + | | + | | + | | + | | + | | | + | | + | | + | | + | | + | | | + | | + | | + | | + | | + | | + | | | + | | + | | + | | + | | + | | | + | | + | | + | | + | | + | + | **D-alanine--D-alanine ligase** |
| **SSGZ1_1201** | | | | | + | | + | | + | | + | | | + | | + | | + | | + | | + | | | + | | + | | + | | + | | + | | | + | | + | | + | | + | | + | | + | | | + | | - | | + | | + | | + | | | + | | + | | + | | + | | + | + | **RecR protein** |
| **SSGZ1_1202** | | | | | + | | + | | + | | + | | | + | | + | | + | | + | | + | | | + | | + | | + | | + | | + | | | + | | + | | + | | + | | + | | + | | | + | | + | | + | | + | | + | | | + | | + | | + | | + | | + | + | **Penicillin-binding protein, transpeptidase** |
| **SSGZ1_1203** | | | | | + | | + | | + | | + | | | + | | + | | + | | + | | + | | | + | | - | | + | | + | | + | | | - | | + | | + | | + | | + | | + | | | + | | + | | + | | + | | - | | | + | | + | | + | | + | | + | + | **Erythromycin resistance ATP-binding protein** |
| **SSGZ1_1204** | | | | | - | | + | | + | | + | | | + | | + | | + | | + | | + | | | + | | - | | + | | + | | + | | | - | | + | | + | | + | | + | | + | | | + | | + | | + | | + | | - | | | + | | + | | + | | + | | + | + | **ABC transporter, ATP-binding protein** |
| **SSGZ1_1205** | | | | | + | | + | | + | | + | | | + | | + | | + | | + | | + | | | + | | + | | + | | + | | + | | | + | | + | | + | | + | | + | | + | | | + | | + | | + | | + | | + | | | + | | + | | + | | + | | + | + | **Beta-lactamase-like protein** |
| **SSGZ1_1206** | | | | | + | | + | | + | | + | | | + | | + | | + | | + | | + | | | + | | + | | + | | + | | + | | | + | | + | | + | | + | | + | | + | | | + | | + | | + | | + | | + | | | + | | + | | + | | + | | + | + | **PAS** |
| **SSGZ1_1207** | | | | | + | | + | | + | | + | | | + | | + | | + | | + | | + | | | + | | + | | + | | + | | + | | | + | | + | | + | | + | | + | | + | | | + | | + | | + | | + | | + | | | + | | + | | + | | + | | + | + | **Response regulator receiver: Transcriptional** |
| **SSGZ1_1208** | | | | | + | | + | | + | | + | | | + | | + | | + | | + | | + | | | + | | + | | + | | + | | + | | | + | | + | | + | | + | | + | | + | | | + | | + | | + | | + | | + | | | + | | + | | + | | + | | + | + | **amino acid ABC transporter, ATP-binding protein** |
| **SSGZ1_1209** | | | | | + | | + | | + | | + | | | + | | + | | + | | + | | + | | | + | | + | | + | | + | | + | | | + | | + | | + | | + | | + | | + | | | + | | + | | + | | + | | + | | | + | | + | | + | | + | | + | + | **Major cell-binding factor precursor (CBF1)** |
| **SSGZ1_1210** | | | | | + | | + | | + | | + | | | + | | + | | + | | + | | + | | | + | | + | | + | | + | | + | | | + | | + | | + | | + | | + | | + | | | + | | + | | + | | + | | + | | | + | | + | | + | | + | | + | + | **ABC transporter membrane-spanning permease -** |
| **SSGZ1_1211** | | | | | + | | + | | + | | + | | | + | | + | | + | | + | | + | | | + | | + | | + | | + | | + | | | + | | + | | + | | + | | + | | + | | | + | | + | | + | | + | | + | | | + | | + | | + | | + | | + | + | **ABC transporter membrane-spanning permease -** |
| **SSGZ1_1212** | | | | | + | | + | | + | | + | | | + | | + | | + | | + | | + | | | + | | + | | + | | + | | + | | | + | | + | | + | | + | | + | | + | | | + | | + | | + | | + | | + | | | + | | + | | + | | + | | + | + | **Threonyl-tRNA synthetase** |
| **SSGZ1_1213** | | | | | + | | + | | + | | + | | | + | | + | | + | | + | | + | | | + | | + | | + | | + | | + | | | + | | + | | + | | + | | + | | + | | | + | | - | | + | | + | | + | | | + | | + | | + | | + | | + | + | **hypothetical protein** |
| **SSGZ1_1214** | | | | | + | | + | | + | | + | | | + | | + | | + | | + | | + | | | + | | + | | + | | + | | + | | | + | | + | | + | | + | | + | | + | | | + | | + | | + | | + | | + | | | + | | + | | + | | + | | + | + | **hypothetical protein** |
| **SSGZ1_1215** | | | | | + | | + | | + | | + | | | + | | + | | + | | + | | + | | | + | | + | | + | | + | | + | | | + | | + | | + | | + | | + | | + | | | + | | + | | + | | + | | + | | | + | | + | | + | | + | | + | + | **Glycosyl transferase, group 1** |
| **SSGZ1_1216** | | | | | + | | + | | + | | + | | | + | | + | | + | | + | | + | | | + | | + | | + | | + | | + | | | + | | + | | + | | + | | + | | + | | | + | | + | | + | | + | | + | | | + | | + | | + | | + | | + | + | **Glycosyl transferase, group 1** |
| **SSGZ1_1217** | | | | | + | | + | | + | | + | | | + | | + | | + | | + | | + | | | + | | + | | + | | + | | - | | | + | | - | | - | | + | | + | | + | | | + | | + | | - | | + | | + | | | + | | + | | + | | - | | + | + | **surface antigen SP1** |
| **SSGZ1_1218** | | | | | + | | + | | + | | + | | | + | | + | | + | | + | | + | | | + | | + | | + | | + | | + | | | + | | + | | + | | + | | + | | + | | | + | | + | | + | | + | | + | | | + | | + | | + | | + | | + | + | **Catabolite control protein A** |
| **SSGZ1_1219** | | | | | + | | + | | + | | + | | | + | | + | | + | | + | | + | | | + | | + | | + | | + | | + | | | + | | + | | + | | + | | + | | + | | | + | | + | | + | | + | | + | | | + | | + | | + | | + | | + | + | **Peptidase M24** |
| **SSGZ1_1220** | | | | | + | | + | | + | | + | | | + | | + | | + | | + | | + | | | + | | + | | + | | + | | + | | | + | | + | | + | | + | | + | | + | | | + | | + | | + | | + | | + | | | + | | + | | + | | + | | + | + | **Queuine tRNA-ribosyltransferase** |
| **SSGZ1_1221** | | | | | + | | + | | + | | + | | | + | | + | | + | | + | | + | | | + | | + | | + | | + | | + | | | + | | + | | + | | + | | + | | + | | | + | | + | | + | | + | | + | | | + | | + | | + | | + | | + | + | **Predicted integral membrane protein** |
| **SSGZ1_1222** | | | | | + | | + | | + | | + | | | + | | + | | + | | + | | + | | | + | | + | | + | | + | | + | | | + | | + | | + | | + | | + | | + | | | + | | + | | + | | + | | + | | | + | | + | | + | | + | | + | + | **CoA-binding** |
| **SSGZ1_1223** | | | | | + | | + | | + | | + | | | + | | + | | + | | + | | + | | | + | | + | | + | | + | | + | | | + | | + | | + | | + | | + | | + | | | + | | + | | + | | + | | + | | | + | | + | | + | | + | | + | + | **Glucose-6-phosphate isomerase** |
| **SSGZ1_1224** | | | | | + | | + | | + | | + | | | + | | + | | + | | + | | + | | | + | | + | | + | | + | | + | | | + | | + | | + | | + | | + | | + | | | + | | + | | + | | + | | + | | | + | | + | | + | | + | | + | + | **DNA polymerase I** |
| **SSGZ1_1225** | | | | | + | | + | | + | | + | | | + | | + | | + | | + | | + | | | + | | + | | + | | + | | + | | | + | | - | | + | | + | | + | | + | | | + | | + | | + | | + | | + | | | + | | + | | + | | + | | + | + | **hypothetical protein** |
| **SSGZ1_1226** | | | | | + | | + | | + | | + | | | + | | + | | + | | + | | + | | | + | | + | | + | | + | | + | | | + | | + | | + | | + | | + | | + | | | + | | + | | + | | + | | + | | | + | | + | | + | | + | | + | + | **ABC-type antimicrobial peptide transport system,** |
| **SSGZ1_1227** | | | | | + | | + | | + | | + | | | + | | + | | + | | + | | + | | | + | | + | | + | | + | | + | | | + | | + | | + | | + | | + | | + | | | + | | + | | + | | + | | + | | | + | | + | | + | | + | | + | + | **ABC transporter, ATP-binding protein** |
| **SSGZ1_1228** | | | | | + | | + | | + | | + | | | + | | + | | + | | + | | + | | | + | | + | | + | | + | | + | | | + | | + | | + | | + | | + | | + | | | + | | + | | + | | + | | + | | | + | | + | | + | | + | | + | + | **Superfamily I DNA and RNA helicase** |
| **SSGZ1_1229** | | | | | + | | + | | + | | + | | | + | | + | | + | | + | | + | | | + | | + | | + | | + | | + | | | + | | + | | + | | + | | + | | + | | | + | | + | | + | | + | | + | | | + | | + | | + | | + | | + | + | **Probable thiol peroxidase** |
| **SSGZ1_1230** | | | | | + | | + | | + | | + | | | + | | + | | + | | + | | + | | | + | | + | | + | | + | | + | | | + | | + | | + | | + | | + | | + | | | + | | - | | + | | + | | + | | | + | | + | | + | | + | | + | + | **Copper-transporting ATPase 1** |
| **SSGZ1_1231** | | | | | + | | + | | + | | + | | | + | | + | | + | | + | | + | | | + | | + | | + | | + | | + | | | + | | + | | + | | + | | + | | + | | | + | | + | | + | | + | | + | | | + | | + | | + | | + | | + | + | **amylase-binding protein B** |
| **SSGZ1_1232** | | | | | + | | + | | + | | + | | | + | | + | | + | | + | | + | | | + | | + | | + | | + | | + | | | + | | + | | + | | + | | + | | + | | | + | | - | | + | | + | | + | | | + | | + | | + | | + | | + | + | **Tellurite resistance protein tehB** |
| **SSGZ1_1233** | | | | | + | | + | | + | | + | | | + | | + | | + | | + | | + | | | + | | + | | + | | + | | + | | | + | | + | | + | | + | | + | | + | | | + | | + | | + | | + | | + | | | + | | + | | + | | + | | + | + | **Zinc-containing alcohol dehydrogenase** |
| **SSGZ1_1234** | | | | | + | | + | | + | | + | | | + | | + | | + | | + | | + | | | + | | + | | + | | + | | + | | | + | | + | | + | | + | | + | | + | | | + | | + | | + | | + | | + | | | + | | + | | + | | + | | + | + | **SmpB protein** |
| **SSGZ1_1235** | | | | | + | | + | | + | | + | | | + | | + | | + | | + | | + | | | + | | + | | + | | + | | + | | | + | | + | | + | | + | | + | | + | | | + | | + | | + | | + | | + | | | + | | + | | + | | + | | + | + | **3'-5' exoribonuclease** |
| **SSGZ1_1236** | | | | | + | | + | | + | | + | | | + | | + | | + | | + | | + | | | + | | + | | + | | + | | + | | | + | | + | | + | | + | | + | | + | | | + | | - | | + | | + | | + | | | + | | + | | + | | + | | + | + | **Preprotein translocase subunit SecG** |
| **SSGZ1_1237** | | | | | + | | + | | + | | + | | | + | | + | | + | | + | | + | | | + | | + | | + | | + | | + | | | + | | + | | + | | + | | + | | + | | | + | | + | | + | | + | | + | | | + | | + | | + | | + | | + | + | **multidrug resistance protein** |
| **SSGZ1_1238** | | | | | + | | + | | + | | + | | | + | | + | | + | | + | | + | | | + | | + | | + | | + | | + | | | + | | + | | + | | + | | + | | + | | | + | | + | | + | | + | | + | | | + | | + | | + | | + | | + | + | **Dephospho-CoA kinase** |
| **SSGZ1_1239** | | | | | + | | + | | + | | + | | | + | | + | | + | | + | | + | | | + | | + | | + | | + | | + | | | + | | + | | + | | + | | + | | + | | | + | | + | | + | | + | | + | | | + | | + | | + | | + | | + | + | **Formamidopyrimidine-DNA glycolase** |
| **SSGZ1_1240** | | | | | + | | + | | + | | + | | | + | | + | | + | | + | | + | | | + | | + | | + | | + | | + | | | + | | + | | + | | + | | + | | + | | | + | | + | | + | | + | | + | | | + | | + | | + | | + | | + | + | **Small GTP-binding protein Era** |
| **SSGZ1_1241** | | | | | + | | + | | + | | + | | | + | | + | | + | | + | | + | | | + | | + | | + | | + | | + | | | + | | + | | + | | + | | + | | + | | | + | | + | | + | | + | | + | | | + | | + | | + | | + | | + | + | **diacyglycerol kinase** |
| **SSGZ1_1242** | | | | | + | | + | | + | | + | | | + | | + | | + | | + | | + | | | + | | + | | + | | + | | + | | | + | | + | | + | | + | | + | | + | | | + | | + | | + | | + | | + | | | + | | + | | + | | + | | + | + | **hypothetical protein** |
| **SSGZ1_1243** | | | | | + | | + | | + | | + | | | + | | + | | + | | + | | + | | | + | | + | | + | | + | | + | | | + | | + | | + | | + | | + | | + | | | + | | + | | + | | + | | + | | | + | | + | | + | | + | | + | + | **Sugar isomerase (SIS)** |
| **SSGZ1_1244** | | | | | + | | + | | + | | + | | | + | | + | | + | | + | | + | | | + | | + | | + | | + | | + | | | + | | + | | + | | + | | + | | + | | | + | | + | | + | | + | | + | | | + | | - | | + | | + | | + | + | **PTS system, glucose-like IIB component** |
| **SSGZ1_1245** | | | | | + | | + | | + | | + | | | + | | + | | + | | + | | + | | | + | | + | | + | | + | | + | | | + | | + | | + | | + | | + | | + | | | + | | + | | + | | + | | + | | | + | | + | | + | | + | | + | + | **putative N-acetylmannosamine 6-P epimerase** |
| **SSGZ1_1246** | | | | | - | | + | | + | | + | | | + | | + | | + | | + | | + | | | + | | - | | + | | + | | - | | | + | | - | | - | | - | | - | | - | | | + | | + | | - | | + | | + | | | + | | - | | + | | - | | + | + | **suilysin** |
| **SSGZ1_1247** | | | | | + | | + | | + | | + | | | + | | + | | + | | + | | + | | | + | | + | | + | | + | | + | | | + | | + | | + | | + | | + | | + | | | + | | + | | + | | + | | + | | | + | | + | | + | | + | | + | + | **hypothetical protein** |
| **SSGZ1_1248** | | | | | + | | + | | + | | + | | | + | | + | | + | | + | | + | | | + | | + | | + | | + | | + | | | + | | + | | + | | + | | + | | + | | | + | | + | | + | | + | | + | | | + | | + | | + | | + | | + | + | **Putative ABC transporter, ATP-binding protein** |
| **SSGZ1_1249** | | | | | + | | + | | + | | + | | | + | | + | | + | | + | | + | | | + | | + | | + | | + | | + | | | + | | + | | + | | + | | + | | + | | | + | | + | | + | | + | | + | | | + | | + | | + | | + | | + | + | **Multidrug resistance-like ATP-binding protein** |
| **SSGZ1_1250** | | | | | + | | + | | + | | + | | | + | | + | | + | | + | | + | | | + | | + | | + | | + | | + | | | + | | + | | + | | + | | + | | + | | | + | | + | | + | | + | | + | | | + | | + | | + | | + | | + | + | **tRNA-Thr** |
| **SSGZ1_1251** | | | | | + | | + | | + | | + | | | + | | + | | + | | + | | + | | | + | | + | | + | | + | | + | | | + | | + | | + | | + | | + | | + | | | + | | + | | + | | + | | + | | | + | | + | | + | | + | | + | + | **Ferrous iron transport protein A** |
| **SSGZ1_1252** | | | | | + | | + | | + | | + | | | + | | + | | + | | + | | + | | | + | | + | | + | | + | | + | | | + | | + | | + | | + | | + | | + | | | + | | + | | + | | + | | + | | | + | | + | | + | | + | | + | + | **Ferrous iron transport protein B** |
| **SSGZ1_1253** | | | | | + | | + | | + | | + | | | + | | + | | + | | + | | + | | | + | | + | | + | | + | | + | | | + | | + | | + | | + | | + | | + | | | + | | + | | + | | + | | + | | | + | | + | | + | | + | | + | + | **Cell division protein** |
| **SSGZ1_1254** | | | | | + | | + | | + | | + | | | + | | + | | + | | + | | + | | | + | | + | | + | | + | | + | | | + | | + | | + | | + | | + | | + | | | + | | + | | + | | + | | + | | | + | | + | | + | | + | | + | + | **Cell division ATP-binding protein** |
| **SSGZ1_1255** | | | | | + | | + | | + | | + | | | + | | + | | + | | + | | + | | | + | | + | | + | | + | | + | | | + | | + | | + | | + | | + | | + | | | + | | + | | + | | + | | + | | | + | | + | | + | | + | | + | + | **Peptide chain release factor 2** |
| **SSGZ1_1256** | | | | | + | | + | | + | | + | | | + | | + | | + | | + | | + | | | + | | + | | + | | + | | + | | | + | | + | | + | | + | | + | | + | | | + | | + | | + | | + | | + | | | + | | + | | + | | + | | + | + | **4Fe-4S ferredoxin** |
| **SSGZ1_1257** | | | | | + | | + | | + | | + | | | + | | + | | + | | + | | + | | | + | | + | | + | | + | | + | | | + | | + | | + | | + | | + | | + | | | + | | + | | + | | + | | + | | | + | | + | | + | | + | | + | + | **VanZ-like protein** |
| **SSGZ1_1258** | | | | | + | | + | | + | | + | | | + | | + | | + | | + | | + | | | + | | + | | + | | + | | + | | | + | | + | | + | | + | | + | | + | | | + | | + | | + | | + | | + | | | + | | + | | + | | + | | + | + | **TM2 domain family** |
| **SSGZ1_1259** | | | | | + | | + | | + | | + | | | + | | + | | + | | + | | + | | | + | | + | | + | | + | | + | | | + | | + | | + | | + | | + | | + | | | - | | + | | + | | + | | + | | | + | | + | | + | | + | | + | + | **hypothetical protein** |
| **SSGZ1_1260** | | | | | + | | + | | + | | + | | | + | | + | | + | | + | | + | | | + | | + | | + | | + | | + | | | + | | + | | + | | + | | + | | + | | | + | | + | | + | | + | | + | | | + | | + | | + | | + | | + | + | **Cation transporting ATPase** |
| **SSGZ1_1261** | | | | | - | | + | | + | | + | | | + | | + | | + | | + | | + | | | - | | - | | - | | - | | - | | | + | | - | | - | | + | | + | | + | | | + | | + | | - | | + | | + | | | + | | + | | + | | - | | + | - | **transposase, IS30 family, putative** |
| **SSGZ1_1262** | | | | | + | | + | | + | | + | | | + | | + | | + | | + | | + | | | + | | + | | + | | + | | + | | | + | | + | | + | | + | | + | | + | | | + | | + | | + | | + | | + | | | + | | + | | + | | + | | + | + | **hypothetical protein** |
| **SSGZ1_1263** | | | | | + | | + | | + | | + | | | + | | + | | + | | + | | + | | | + | | + | | + | | + | | + | | | + | | + | | + | | + | | + | | + | | | + | | + | | + | | + | | + | | | + | | + | | + | | + | | + | + | **hypothetical protein** |
| **SSGZ1_1264** | | | | | + | | + | | + | | + | | | + | | + | | + | | + | | + | | | + | | + | | + | | + | | + | | | + | | + | | - | | + | | + | | + | | | + | | + | | + | | + | | + | | | + | | + | | + | | - | | + | + | **Transposase IS200-like** |
| **SSGZ1_1265** | | | | | + | | + | | + | | + | | | + | | + | | + | | + | | + | | | + | | + | | + | | + | | + | | | + | | + | | - | | + | | + | | + | | | + | | + | | + | | + | | + | | | + | | + | | + | | - | | + | + | **hypothetical protein** |
| **SSGZ1_1267** | | | | | + | | + | | + | | + | | | + | | + | | + | | + | | + | | | + | | + | | + | | + | | + | | | + | | + | | + | | + | | + | | + | | | + | | + | | + | | + | | + | | | + | | + | | + | | + | | + | + | **Predicted metal-sulfur cluster biosynthetic** |
| **SSGZ1_1268** | | | | | + | | + | | + | | + | | | + | | + | | + | | + | | + | | | + | | + | | + | | + | | + | | | + | | + | | + | | + | | + | | + | | | + | | + | | + | | + | | + | | | + | | + | | + | | + | | + | + | **RNA polymerase sigma factor rpoD** |
| **SSGZ1_1269** | | | | | + | | + | | + | | + | | | + | | + | | + | | + | | + | | | + | | + | | + | | + | | + | | | + | | + | | + | | + | | + | | + | | | + | | + | | + | | + | | + | | | + | | + | | + | | + | | + | + | **DNA primase** |
| **SSGZ1_1270** | | | | | + | | + | | + | | + | | | + | | + | | + | | + | | + | | | + | | + | | + | | + | | + | | | + | | + | | + | | + | | + | | + | | | + | | + | | + | | + | | + | | | + | | + | | + | | + | | + | + | **Large-conductance mechanosensitive channel** |
| **SSGZ1_1271** | | | | | + | | + | | + | | + | | | + | | + | | + | | + | | + | | | + | | + | | + | | + | | + | | | + | | + | | + | | + | | + | | + | | | + | | + | | + | | + | | + | | | + | | + | | + | | + | | + | + | **UDP-glucose 4-epimerase** |
| **SSGZ1_1272** | | | | | + | | + | | + | | + | | | + | | + | | + | | + | | + | | | + | | + | | + | | + | | + | | | + | | + | | + | | + | | + | | + | | | + | | + | | + | | + | | + | | | + | | + | | + | | + | | + | + | **30S Ribosomal protein S21** |
| **SSGZ1_1273** | | | | | + | | + | | + | | + | | | + | | + | | + | | + | | + | | | + | | + | | + | | + | | + | | | - | | + | | + | | + | | + | | + | | | + | | + | | + | | + | | - | | | + | | + | | + | | + | | + | + | **Integral membrane protein** |
| **SSGZ1_1274** | | | | | + | | + | | + | | + | | | + | | + | | + | | + | | + | | | + | | + | | + | | + | | + | | | + | | + | | + | | + | | + | | + | | | + | | + | | + | | + | | + | | | + | | + | | + | | + | | + | + | **HAD-superfamily subfamily IIA hydrolase** |
| **SSGZ1_1275** | | | | | + | | + | | + | | + | | | + | | + | | + | | + | | + | | | + | | + | | + | | + | | + | | | + | | + | | + | | + | | + | | + | | | + | | + | | + | | + | | + | | | + | | + | | + | | + | | + | + | **NUDIX hydrolase** |
| **SSGZ1_1276** | | | | | + | | + | | + | | + | | | + | | + | | + | | + | | + | | | + | | + | | + | | + | | + | | | + | | + | | + | | + | | + | | + | | | + | | + | | + | | + | | + | | | + | | + | | + | | + | | + | + | **acyl-acyl carrier protein thioesterase** |
| **SSGZ1_1277** | | | | | + | | + | | + | | + | | | + | | + | | + | | + | | + | | | + | | + | | + | | + | | + | | | + | | + | | + | | + | | + | | + | | | + | | + | | + | | + | | + | | | + | | + | | + | | + | | + | + | **coproporphyrinogen III oxidase** |
| **SSGZ1_1278** | | | | | + | | + | | + | | + | | | + | | + | | + | | + | | + | | | + | | + | | + | | + | | + | | | + | | + | | + | | + | | + | | + | | | + | | + | | + | | + | | - | | | + | | + | | + | | + | | + | + | **hypothetical protein** |
| **SSGZ1_1279** | | | | | + | | + | | + | | + | | | + | | + | | + | | + | | + | | | + | | + | | + | | + | | + | | | + | | + | | + | | + | | + | | + | | | + | | + | | + | | + | | + | | | + | | + | | + | | + | | + | + | **Maltodextrin phosphorylase** |
| **SSGZ1_1280** | | | | | + | | + | | + | | + | | | + | | + | | + | | + | | + | | | + | | + | | + | | + | | + | | | + | | + | | + | | + | | + | | + | | | + | | + | | + | | + | | + | | | + | | + | | + | | + | | + | + | **Purine nucleoside phosphorylase deoD-type** |
| **SSGZ1_1281** | | | | | + | | + | | + | | + | | | + | | + | | + | | + | | + | | | + | | + | | + | | + | | + | | | + | | + | | + | | + | | + | | + | | | + | | + | | + | | + | | + | | | + | | + | | + | | + | | + | + | **Purine nucleoside phosphorylase 1** |
| **SSGZ1_1282** | | | | | + | | + | | + | | + | | | + | | + | | + | | + | | + | | | + | | + | | + | | + | | + | | | + | | + | | + | | + | | + | | + | | | + | | + | | + | | + | | + | | | + | | + | | + | | + | | + | + | **Arsenate reductase** |
| **SSGZ1_1283** | | | | | + | | + | | + | | + | | | + | | + | | + | | + | | + | | | + | | + | | + | | + | | + | | | + | | + | | + | | + | | + | | + | | | + | | + | | + | | + | | + | | | + | | + | | + | | + | | + | + | **Phosphopentomutase** |
| **SSGZ1_1284** | | | | | + | | + | | + | | + | | | + | | + | | + | | + | | + | | | + | | + | | + | | + | | + | | | + | | + | | + | | + | | + | | + | | | + | | + | | + | | + | | + | | | + | | + | | + | | + | | + | + | **Ribose 5-phosphate isomerase** |
| **SSGZ1_1285** | | | | | - | | + | | + | | + | | | + | | + | | + | | + | | + | | | - | | - | | - | | - | | + | | | - | | - | | - | | - | | - | | + | | | + | | - | | + | | - | | - | | | - | | - | | - | | - | | - | + | **Type I restriction modification DNA specificity** |
| **SSGZ1_1286** | | | | | - | | + | | + | | + | | | + | | + | | + | | + | | + | | | - | | - | | - | | - | | + | | | - | | - | | - | | - | | - | | - | | | + | | - | | - | | - | | - | | | - | | - | | - | | - | | - | + | **type I restriction-modification system, S** |
| **SSGZ1_1287** | | | | | - | | + | | + | | + | | | + | | + | | + | | + | | + | | | - | | - | | - | | - | | - | | | - | | - | | - | | - | | - | | - | | | + | | - | | - | | - | | - | | | - | | - | | - | | - | | - | + | **Type I restriction enzyme EcoEI M protein** |
| **SSGZ1_1288** | | | | | - | | + | | + | | + | | | + | | + | | + | | + | | + | | | - | | - | | - | | - | | + | | | - | | - | | - | | - | | - | | - | | | + | | - | | + | | - | | - | | | - | | - | | - | | - | | - | + | **Type I restriction enzyme EcoEI R protein** |
| **SSGZ1_1289** | | | | | + | | + | | + | | + | | | + | | + | | + | | + | | + | | | + | | + | | + | | + | | + | | | + | | + | | + | | + | | + | | + | | | + | | + | | + | | + | | + | | | + | | + | | + | | + | | + | + | **tRNA modification GTPase TrmE** |
| **SSGZ1_1290** | | | | | + | | + | | + | | + | | | + | | + | | + | | + | | + | | | + | | + | | + | | + | | + | | | + | | + | | + | | + | | + | | + | | | + | | + | | + | | + | | - | | | + | | + | | + | | + | | + | + | **hypothetical protein** |
| **SSGZ1_1291** | | | | | + | | + | | + | | + | | | + | | + | | + | | + | | + | | | + | | + | | + | | + | | + | | | + | | + | | + | | + | | + | | + | | | + | | + | | + | | + | | - | | | - | | - | | + | | + | | + | + | **hypothetical protein** |
| **SSGZ1_1292** | | | | | + | | + | | + | | + | | | + | | + | | + | | + | | + | | | + | | + | | + | | + | | + | | | + | | + | | + | | + | | + | | - | | | + | | + | | + | | - | | - | | | - | | - | | + | | + | | + | + | **hypothetical protein** |
| **SSGZ1_1293** | | | | | + | | + | | + | | + | | | + | | + | | + | | + | | + | | | + | | + | | + | | + | | + | | | + | | + | | + | | + | | + | | + | | | + | | + | | + | | + | | + | | | + | | + | | + | | + | | + | + | **LemA** |
| **SSGZ1_1294** | | | | | + | | + | | + | | + | | | + | | + | | + | | + | | + | | | + | | + | | + | | + | | + | | | + | | + | | + | | + | | + | | + | | | + | | + | | + | | + | | - | | | + | | + | | + | | + | | + | + | **hypothetical protein** |
| **SSGZ1_1295** | | | | | + | | + | | + | | + | | | + | | + | | + | | + | | + | | | + | | + | | + | | + | | + | | | + | | + | | + | | + | | + | | + | | | + | | + | | + | | + | | + | | | + | | + | | + | | + | | + | + | **Uracil-DNA glycosylase superfamily** |
| **SSGZ1_1296** | | | | | + | | + | | + | | + | | | + | | + | | + | | + | | + | | | + | | + | | + | | + | | + | | | + | | + | | + | | + | | + | | + | | | + | | + | | + | | + | | + | | | + | | + | | + | | + | | + | + | **GCN5-related N-acetyltransferase** |
| **SSGZ1_1297** | | | | | + | | + | | + | | + | | | + | | + | | + | | + | | + | | | + | | + | | + | | + | | + | | | + | | + | | + | | + | | + | | + | | | + | | + | | + | | + | | + | | | + | | + | | + | | + | | + | + | **Dipeptidase** |
| **SSGZ1_1298** | | | | | + | | + | | + | | + | | | + | | + | | + | | + | | + | | | + | | + | | + | | + | | + | | | + | | + | | + | | + | | + | | + | | | + | | + | | + | | + | | + | | | + | | + | | + | | + | | + | + | **serine/threonine: Na+ symporter** |
| **SSGZ1_1299** | | | | | + | | + | | + | | + | | | + | | + | | + | | + | | + | | | + | | + | | + | | + | | + | | | + | | + | | + | | + | | + | | + | | | + | | + | | + | | + | | + | | | + | | + | | + | | + | | + | + | **hypothetical protein** |
| **SSGZ1_1300** | | | | | - | | + | | + | | + | | | + | | + | | + | | + | | + | | | - | | - | | - | | - | | + | | | + | | + | | - | | - | | - | | - | | | + | | + | | + | | - | | - | | | - | | - | | - | | + | | - | + | **hypothetical protein** |
| **SSGZ1_1301** | | | | | - | | + | | + | | + | | | + | | + | | + | | + | | + | | | - | | - | | - | | - | | + | | | + | | + | | - | | - | | - | | - | | | + | | + | | + | | - | | - | | | - | | - | | - | | + | | - | - | **hypothetical protein** |
| **SSGZ1_1302** | | | | | - | | + | | + | | + | | | + | | + | | + | | + | | + | | | - | | - | | - | | - | | + | | | + | | + | | - | | + | | - | | + | | | + | | + | | + | | - | | - | | | - | | - | | + | | + | | + | + | **hypothetical protein** |
| **SSGZ1_1303** | | | | | + | | + | | + | | + | | | + | | + | | + | | + | | + | | | + | | + | | + | | + | | + | | | + | | + | | + | | + | | + | | - | | | + | | + | | + | | - | | - | | | - | | - | | + | | + | | + | + | **SAM-dependent methyltransferase** |
| **SSGZ1_1304** | | | | | + | | + | | + | | + | | | + | | + | | + | | + | | + | | | + | | + | | + | | + | | + | | | + | | + | | + | | + | | + | | + | | | + | | + | | + | | + | | + | | | + | | + | | + | | + | | + | + | **hypothetical protein** |
| **SSGZ1_1305** | | | | | + | | + | | + | | + | | | + | | + | | + | | + | | + | | | + | | + | | + | | + | | + | | | + | | + | | + | | + | | + | | + | | | + | | + | | + | | + | | + | | | + | | + | | + | | + | | + | + | **Phosphoglucosamine mutase** |
| **SSGZ1_1306** | | | | | + | | + | | + | | + | | | + | | + | | + | | + | | + | | | + | | + | | + | | + | | + | | | + | | + | | + | | + | | + | | + | | | + | | + | | + | | + | | + | | | + | | + | | + | | + | | + | + | **hypothetical protein** |
| **SSGZ1_1307** | | | | | + | | + | | + | | + | | | + | | + | | + | | + | | + | | | + | | + | | + | | + | | + | | | + | | + | | + | | + | | + | | + | | | + | | + | | + | | + | | + | | | + | | + | | + | | + | | + | + | **hypothetical protein** |
| **SSGZ1_1308** | | | | | + | | + | | + | | + | | | + | | + | | + | | + | | + | | | + | | + | | + | | + | | + | | | + | | + | | + | | + | | + | | + | | | + | | + | | + | | + | | + | | | + | | + | | + | | + | | + | + | **putative UDP-N-acetylmuramyl tripeptide** |
| **SSGZ1_1309** | | | | | + | | + | | + | | + | | | + | | + | | + | | + | | + | | | + | | + | | + | | + | | + | | | + | | + | | + | | + | | + | | + | | | + | | + | | + | | + | | + | | | + | | + | | + | | + | | + | + | **cobyric acid synthase** |
| **SSGZ1_1310** | | | | | + | | + | | + | | + | | | + | | + | | + | | + | | + | | | + | | + | | + | | + | | + | | | + | | + | | + | | + | | + | | + | | | + | | + | | + | | + | | + | | | + | | + | | + | | + | | + | + | **rRNA (guanine-N(1)-)-methyltransferase** |
| **SSGZ1_1311** | | | | | - | | + | | + | | + | | | + | | + | | + | | + | | + | | | + | | + | | + | | + | | + | | | - | | + | | + | | + | | + | | + | | | + | | - | | + | | + | | + | | | + | | + | | + | | + | | + | + | **hypothetical protein** |
| **SSGZ1_1312** | | | | | + | | + | | + | | + | | | + | | + | | + | | + | | + | | | + | | + | | + | | + | | + | | | + | | + | | + | | + | | + | | + | | | + | | + | | + | | + | | + | | | + | | + | | + | | + | | + | + | **ABC transporter, ATP-binding protein** |
| **SSGZ1_1313** | | | | | + | | + | | + | | + | | | + | | + | | + | | + | | + | | | + | | + | | + | | + | | + | | | + | | + | | + | | + | | + | | + | | | + | | + | | + | | + | | + | | | + | | + | | + | | + | | + | + | **hypothetical protein** |
| **SSGZ1_1314** | | | | | + | | + | | + | | + | | | + | | + | | + | | + | | + | | | + | | + | | + | | + | | + | | | + | | + | | + | | + | | + | | + | | | + | | + | | + | | + | | + | | | + | | + | | + | | + | | + | + | **Predicted Zn-dependent protease** |
| **SSGZ1_1315** | | | | | + | | + | | + | | + | | | + | | + | | + | | + | | + | | | + | | + | | + | | + | | + | | | + | | + | | + | | + | | + | | + | | | + | | + | | + | | + | | + | | | + | | + | | + | | + | | + | + | **Putative esterase** |
| **SSGZ1_1316** | | | | | + | | + | | + | | + | | | + | | + | | + | | + | | + | | | + | | + | | + | | + | | + | | | + | | + | | + | | + | | + | | + | | | + | | + | | + | | + | | + | | | + | | + | | + | | + | | + | + | **Beta-lactamase-like: RNA-metabolising** |
| **SSGZ1_1317** | | | | | + | | + | | + | | + | | | + | | + | | + | | + | | + | | | + | | + | | + | | + | | + | | | + | | + | | + | | + | | + | | + | | | + | | + | | + | | + | | + | | | + | | + | | + | | + | | + | + | **hypothetical protein** |
| **SSGZ1_1318** | | | | | + | | + | | + | | + | | | + | | + | | + | | + | | + | | | + | | + | | + | | + | | + | | | + | | + | | + | | + | | + | | + | | | + | | + | | + | | + | | + | | | + | | + | | + | | + | | + | + | **hypothetical protein** |
| **SSGZ1_1319** | | | | | + | | + | | + | | + | | | + | | + | | + | | + | | + | | | + | | + | | + | | + | | + | | | + | | + | | + | | + | | + | | + | | | + | | + | | + | | + | | + | | | + | | + | | + | | + | | + | + | **ABC transporter, ATP-binding protein** |
| **SSGZ1_1320** | | | | | + | | + | | + | | + | | | + | | + | | + | | + | | + | | | + | | + | | + | | + | | + | | | + | | + | | + | | + | | + | | + | | | + | | + | | + | | + | | + | | | + | | + | | + | | + | | + | + | **inner-membrane translocator** |
| **SSGZ1_1321** | | | | | + | | + | | + | | + | | | + | | + | | + | | + | | + | | | + | | + | | + | | + | | + | | | + | | + | | + | | + | | + | | + | | | + | | + | | + | | + | | + | | | + | | + | | + | | + | | + | + | **ABC-type uncharacterized transport system,** |
| **SSGZ1_1322** | | | | | + | | + | | + | | + | | | + | | + | | + | | + | | + | | | + | | + | | + | | + | | + | | | + | | - | | + | | + | | - | | + | | | + | | + | | + | | - | | - | | | - | | - | | + | | + | | + | + | **6-phospho-beta-glucosidase** |
| **SSGZ1_1323** | | | | | + | | + | | + | | + | | | + | | + | | + | | + | | + | | | + | | + | | + | | + | | + | | | + | | - | | + | | + | | - | | - | | | + | | + | | + | | - | | - | | | - | | - | | + | | + | | + | - | **Sugar-specific permease, EIIA 1 domain:PTS** |
| **SSGZ1_1324** | | | | | + | | + | | + | | + | | | + | | + | | + | | + | | + | | | + | | + | | + | | + | | + | | | + | | + | | + | | + | | - | | + | | | + | | + | | + | | + | | - | | | - | | + | | + | | + | | + | + | **Transcriptional antiterminator bglG** |
| **SSGZ1_1325** | | | | | + | | + | | + | | + | | | + | | + | | + | | + | | + | | | + | | + | | + | | + | | + | | | + | | + | | + | | + | | + | | + | | | + | | + | | + | | + | | + | | | + | | + | | + | | + | | + | + | **fibrinogen binding protein** |
| **SSGZ1_1326** | | | | | + | | + | | + | | + | | | + | | + | | + | | + | | + | | | + | | + | | + | | + | | + | | | + | | + | | + | | + | | + | | + | | | + | | + | | + | | + | | + | | | + | | + | | + | | + | | + | + | **Alpha-acetolactate decarboxylase** |
| **SSGZ1_1327** | | | | | + | | + | | + | | + | | | + | | + | | + | | + | | + | | | + | | + | | + | | + | | + | | | + | | + | | + | | + | | + | | + | | | + | | + | | + | | + | | + | | | + | | + | | + | | + | | + | + | **tetratricopeptide repeat family protein** |
| **SSGZ1_1328** | | | | | + | | + | | + | | + | | | + | | + | | + | | + | | + | | | + | | + | | + | | + | | + | | | + | | + | | + | | + | | + | | + | | | + | | + | | + | | + | | + | | | + | | + | | + | | + | | + | + | **Predicted permease** |
| **SSGZ1_1329** | | | | | + | | + | | + | | + | | | + | | + | | + | | + | | + | | | + | | + | | + | | + | | + | | | + | | + | | + | | + | | + | | + | | | + | | + | | + | | + | | + | | | + | | + | | + | | + | | + | + | **3-carboxymuconate cyclase** |
| **SSGZ1_1330** | | | | | + | | + | | + | | + | | | + | | + | | + | | + | | + | | | + | | + | | + | | + | | + | | | + | | + | | + | | + | | + | | + | | | + | | + | | + | | + | | + | | | + | | + | | + | | + | | + | + | **3-carboxymuconate cyclase** |
| **SSGZ1_1331** | | | | | + | | + | | + | | + | | | + | | + | | + | | + | | + | | | + | | + | | + | | + | | + | | | + | | + | | + | | + | | + | | + | | | + | | + | | + | | + | | + | | | + | | + | | + | | + | | + | + | **Isoleucyl-tRNA synthetase, putative** |
| **SSGZ1_1332** | | | | | + | | + | | + | | + | | | + | | + | | + | | + | | + | | | + | | + | | + | | + | | + | | | + | | + | | + | | + | | + | | + | | | + | | + | | + | | + | | + | | | + | | + | | + | | + | | + | + | **HAD-superfamily hydrolase** |
| **SSGZ1_1333** | | | | | - | | + | | + | | + | | | + | | + | | + | | + | | + | | | - | | + | | - | | - | | - | | | - | | + | | - | | - | | - | | - | | | + | | - | | - | | + | | - | | | - | | + | | - | | + | | - | + | **Cytotoxic translational repressor of** |
| **SSGZ1_1334** | | | | | - | | + | | + | | + | | | + | | + | | + | | + | | + | | | + | | + | | + | | - | | + | | | - | | + | | + | | + | | - | | + | | | + | | - | | + | | + | | - | | | - | | + | | + | | + | | + | + | **hypothetical protein** |
| **SSGZ1_1335** | | | | | + | | + | | + | | + | | | + | | + | | + | | + | | + | | | + | | + | | + | | + | | + | | | + | | + | | + | | + | | + | | + | | | + | | + | | + | | + | | + | | | + | | + | | + | | + | | + | + | **putative Enolase** |
| **SSGZ1_1336** | | | | | + | | + | | + | | + | | | + | | + | | + | | + | | + | | | + | | + | | + | | + | | + | | | + | | + | | + | | + | | + | | + | | | + | | + | | + | | + | | + | | | + | | + | | + | | + | | + | + | **hypothetical protein** |
| **SSGZ1_1337** | | | | | + | | + | | + | | + | | | + | | + | | + | | + | | + | | | + | | + | | + | | + | | + | | | + | | + | | + | | + | | + | | + | | | + | | + | | + | | + | | + | | | + | | + | | + | | + | | + | + | **hypothetical protein** |
| **SSGZ1_1338** | | | | | + | | + | | + | | + | | | + | | + | | + | | + | | + | | | + | | + | | + | | + | | + | | | + | | + | | + | | + | | + | | + | | | + | | + | | + | | + | | + | | | + | | + | | + | | + | | + | + | **hypothetical protein** |
| **SSGZ1_1339** | | | | | + | | + | | + | | + | | | + | | + | | + | | + | | + | | | + | | + | | + | | + | | + | | | + | | + | | + | | + | | + | | + | | | + | | + | | + | | - | | - | | | - | | + | | + | | + | | + | + | **Glycerate kinase** |
| **SSGZ1_1340** | | | | | + | | + | | + | | + | | | + | | + | | + | | + | | + | | | + | | + | | + | | + | | + | | | + | | + | | + | | + | | + | | + | | | + | | + | | + | | + | | + | | | + | | + | | + | | + | | + | + | **HAD-superfamily hydrolase** |
| **SSGZ1_1341** | | | | | + | | + | | + | | + | | | + | | + | | + | | + | | + | | | + | | + | | + | | + | | + | | | + | | + | | + | | + | | + | | + | | | + | | + | | + | | + | | + | | | + | | + | | + | | + | | + | + | **Transposase** |
| **SSGZ1_1342** | | | | | + | | + | | + | | + | | | + | | + | | + | | + | | + | | | + | | + | | + | | + | | + | | | + | | + | | + | | + | | + | | - | | | + | | + | | + | | + | | + | | | + | | + | | + | | + | | + | + | **Transposase** |
| **SSGZ1_1343** | | | | | + | | + | | + | | + | | | + | | + | | + | | + | | + | | | + | | + | | + | | + | | + | | | + | | + | | + | | + | | + | | + | | | + | | + | | + | | + | | + | | | + | | + | | + | | + | | + | + | **Phosphoserine phosphatase SerB** |
| **SSGZ1_1344** | | | | | + | | + | | + | | + | | | + | | + | | + | | + | | + | | | + | | + | | + | | + | | + | | | + | | + | | + | | + | | + | | + | | | + | | + | | + | | + | | + | | | + | | + | | + | | + | | + | + | **septation ring formation regulator EzrA** |
| **SSGZ1_1345** | | | | | + | | + | | + | | + | | | + | | + | | + | | + | | + | | | + | | + | | + | | + | | + | | | + | | + | | + | | + | | + | | + | | | + | | + | | + | | + | | + | | | + | | + | | + | | + | | + | + | **DNA gyrase, B subunit** |
| **SSGZ1_1346** | | | | | + | | + | | + | | + | | | + | | + | | + | | + | | + | | | + | | + | | + | | + | | + | | | + | | + | | + | | + | | + | | + | | | + | | + | | + | | + | | + | | | + | | + | | + | | + | | + | + | **4-methyl-5(B-hydroxyethyl)-thiazole** |
| **SSGZ1_1347** | | | | | + | | + | | + | | + | | | + | | + | | + | | + | | + | | | + | | + | | + | | + | | + | | | + | | + | | + | | + | | + | | + | | | + | | + | | + | | + | | + | | | + | | + | | + | | + | | + | + | **rod shape-determining protein RodA, putative** |
| **SSGZ1_1348** | | | | | - | | + | | + | | + | | | + | | + | | + | | + | | + | | | - | | - | | - | | - | | - | | | - | | + | | - | | - | | - | | - | | | + | | - | | - | | + | | + | | | + | | - | | - | | - | | - | - | **prophage LambdaSa2, site-specific recombinase,** |
| **SSGZ1_1349** | | | | | - | | + | | + | | + | | | + | | + | | + | | + | | + | | | - | | - | | - | | - | | - | | | - | | + | | - | | - | | - | | - | | | + | | - | | - | | + | | + | | | + | | - | | - | | - | | - | - | **hypothetical protein** |
| **SSGZ1_1350** | | | | | - | | + | | + | | + | | | + | | + | | + | | + | | + | | | - | | - | | - | | - | | - | | | - | | + | | - | | - | | - | | - | | | + | | - | | - | | + | | + | | | + | | - | | - | | - | | - | - | **Virulence-associated protein E** |
| **SSGZ1_1351** | | | | | - | | + | | + | | + | | | + | | + | | + | | + | | + | | | - | | - | | - | | - | | + | | | - | | + | | + | | - | | - | | + | | | + | | - | | + | | + | | + | | | + | | - | | - | | - | | - | + | **phage protein** |
| **SSGZ1_1352** | | | | | - | | + | | + | | + | | | + | | + | | + | | + | | + | | | + | | + | | + | | + | | + | | | - | | + | | + | | + | | + | | + | | | + | | - | | + | | + | | + | | | + | | + | | + | | - | | + | + | **hypothetical protein** |
| **SSGZ1_1353** | | | | | - | | + | | + | | + | | | + | | + | | + | | + | | + | | | - | | - | | - | | - | | - | | | - | | + | | - | | - | | - | | - | | | + | | - | | - | | + | | + | | | + | | - | | - | | - | | - | - | **unknown phage protein** |
| **SSGZ1_1354** | | | | | - | | + | | + | | + | | | + | | + | | + | | + | | + | | | - | | - | | - | | - | | - | | | - | | + | | - | | - | | - | | - | | | + | | - | | - | | + | | + | | | + | | - | | - | | - | | - | - | **bovine pathogenicity island protein** |
| **SSGZ1_1355** | | | | | - | | + | | + | | + | | | + | | + | | + | | + | | + | | | - | | - | | - | | - | | - | | | - | | + | | - | | - | | - | | - | | | + | | - | | - | | + | | + | | | + | | - | | - | | - | | - | - | **unknown phage protein** |
| **SSGZ1_1356** | | | | | - | | + | | + | | + | | | + | | + | | + | | + | | + | | | - | | - | | - | | - | | + | | | - | | + | | - | | - | | - | | - | | | + | | - | | - | | + | | + | | | + | | - | | - | | - | | - | + | **Trichomonas vaginalis G3 hypothetical protein** |
| **SSGZ1_1357** | | | | | - | | + | | + | | + | | | + | | + | | + | | + | | + | | | - | | - | | - | | - | | - | | | - | | + | | - | | - | | - | | - | | | + | | - | | - | | + | | + | | | + | | - | | - | | - | | - | - | **hypothetical protein** |
| **SSGZ1_1358** | | | | | - | | + | | + | | + | | | + | | + | | + | | + | | + | | | - | | - | | - | | - | | + | | | - | | + | | + | | - | | - | | + | | | + | | - | | + | | + | | + | | | + | | - | | - | | - | | - | + | **hypothetical protein** |
| **SSGZ1_1359** | | | | | - | | + | | + | | + | | | + | | + | | + | | + | | + | | | - | | - | | - | | - | | - | | | - | | + | | - | | - | | - | | - | | | + | | - | | + | | + | | + | | | + | | - | | - | | - | | - | + | **hypothetical protein** |
| **SSGZ1_1360** | | | | | - | | + | | + | | + | | | + | | + | | + | | + | | + | | | - | | - | | - | | - | | - | | | - | | + | | - | | - | | - | | - | | | + | | - | | - | | + | | + | | | + | | - | | - | | - | | - | + | **hypothetical protein** |
| **SSGZ1_1361** | | | | | - | | + | | + | | + | | | + | | + | | + | | + | | + | | | - | | - | | - | | - | | - | | | - | | + | | - | | - | | - | | - | | | + | | - | | - | | + | | - | | | + | | - | | - | | - | | - | + | **Predicted transcriptional regulator** |
| **SSGZ1_1362** | | | | | - | | + | | + | | + | | | + | | + | | + | | + | | + | | | - | | - | | - | | - | | + | | | - | | + | | + | | - | | - | | + | | | + | | - | | + | | + | | - | | | + | | + | | - | | - | | - | + | **prophage Sa05, DNA-binding protein** |
| **SSGZ1_1363** | | | | | - | | + | | + | | + | | | + | | + | | + | | + | | + | | | - | | - | | - | | - | | + | | | - | | + | | - | | - | | - | | + | | | + | | - | | + | | + | | - | | | + | | + | | - | | - | | - | + | **bacteriophage U153 int gene for U153 integrase** |
| **SSGZ1_1364** | | | | | - | | + | | + | | + | | | + | | + | | + | | + | | + | | | - | | - | | - | | - | | - | | | - | | + | | - | | - | | - | | - | | | + | | - | | - | | + | | + | | | + | | + | | - | | - | | - | + | **integrase** |
| **SSGZ1_1365** | | | | | + | | + | | + | | + | | | + | | + | | + | | + | | + | | | + | | + | | + | | + | | + | | | + | | + | | + | | + | | + | | + | | | + | | + | | + | | + | | + | | | + | | + | | + | | + | | + | + | **Ribosomal protein L31** |
| **SSGZ1_1366** | | | | | + | | + | | + | | + | | | + | | + | | + | | + | | + | | | + | | + | | + | | + | | + | | | + | | + | | + | | + | | + | | + | | | + | | + | | + | | + | | + | | | + | | + | | + | | + | | + | + | **lipoprotein involved thiamine biosynthesis** |
| **SSGZ1_1367** | | | | | + | | + | | + | | + | | | + | | + | | + | | + | | + | | | + | | + | | + | | + | | + | | | + | | + | | + | | + | | + | | + | | | + | | + | | + | | + | | + | | | + | | + | | + | | + | | + | + | **DHH subfamily 1 protein** |
| **SSGZ1_1368** | | | | | + | | + | | + | | + | | | + | | + | | + | | + | | + | | | + | | + | | + | | + | | + | | | + | | + | | + | | + | | + | | + | | | + | | + | | + | | + | | + | | | + | | + | | + | | + | | + | + | **flavodoxin** |
| **SSGZ1_1369** | | | | | + | | + | | + | | + | | | + | | + | | + | | + | | + | | | + | | + | | + | | + | | + | | | + | | + | | + | | + | | + | | + | | | + | | + | | + | | + | | + | | | + | | + | | + | | + | | + | + | **30S ribosomal protein S14** |
| **SSGZ1_1370** | | | | | + | | + | | + | | + | | | + | | + | | + | | + | | + | | | + | | + | | + | | + | | + | | | + | | + | | + | | + | | + | | + | | | + | | + | | + | | + | | + | | | + | | + | | + | | + | | + | + | **chorismate mutase** |
| **SSGZ1_1371** | | | | | + | | + | | + | | + | | | + | | + | | + | | + | | + | | | + | | + | | + | | + | | + | | | + | | + | | + | | + | | + | | + | | | + | | + | | + | | + | | + | | | + | | + | | + | | + | | + | + | **tRNA-Arg** |
| **SSGZ1_1372** | | | | | + | | + | | + | | + | | | + | | + | | + | | + | | + | | | + | | + | | + | | + | | + | | | + | | + | | + | | + | | + | | + | | | + | | + | | + | | + | | + | | | + | | + | | + | | + | | + | + | **putative 5'-nucleotidase** |
| **SSGZ1_1373** | | | | | + | | + | | + | | + | | | + | | + | | + | | + | | + | | | + | | + | | + | | + | | + | | | + | | + | | + | | + | | + | | + | | | + | | + | | + | | + | | + | | | + | | + | | + | | + | | + | + | **superoxide dismutase A** |
| **SSGZ1_1374** | | | | | + | | + | | + | | + | | | + | | + | | + | | + | | + | | | + | | + | | + | | + | | + | | | + | | + | | + | | + | | + | | + | | | + | | + | | + | | + | | + | | | + | | + | | + | | + | | + | + | **DNA polymerase III delta subunit** |
| **SSGZ1_1375** | | | | | + | | + | | + | | + | | | + | | + | | + | | + | | + | | | + | | + | | + | | + | | + | | | + | | + | | + | | + | | + | | + | | | + | | + | | + | | + | | + | | | + | | + | | + | | + | | + | + | **phage protein** |
| **SSGZ1_1376** | | | | | + | | + | | + | | + | | | + | | + | | + | | + | | + | | | + | | + | | + | | + | | + | | | + | | + | | + | | + | | + | | - | | | + | | + | | + | | + | | + | | | + | | + | | + | | + | | + | + | **hypothetical cytosolic protein** |
| **SSGZ1_1377** | | | | | + | | + | | + | | + | | | + | | + | | + | | + | | + | | | + | | + | | + | | + | | + | | | + | | + | | + | | + | | + | | + | | | + | | + | | + | | + | | + | | | + | | + | | + | | + | | + | + | **ABC-type branched-chain amino acid transport** |
| **SSGZ1_1378** | | | | | + | | + | | + | | + | | | + | | + | | + | | + | | + | | | + | | + | | + | | + | | + | | | + | | + | | + | | + | | + | | + | | | + | | + | | + | | + | | + | | | + | | + | | + | | + | | + | + | **branched-chain amino acid ABC transporter,** |
| **SSGZ1_1379** | | | | | + | | + | | + | | + | | | + | | + | | + | | + | | + | | | + | | + | | + | | + | | + | | | + | | + | | + | | + | | + | | + | | | + | | + | | + | | + | | + | | | + | | + | | + | | + | | + | + | **branched-chain amino acid ABC transporter,** |
| **SSGZ1_1380** | | | | | + | | + | | + | | + | | | + | | + | | + | | + | | + | | | + | | + | | + | | + | | + | | | + | | + | | + | | + | | + | | + | | | + | | + | | + | | + | | + | | | + | | + | | + | | + | | + | + | **branched-chain amino acid ABC transporter,** |
| **SSGZ1_1381** | | | | | + | | + | | + | | + | | | + | | + | | + | | + | | + | | | + | | + | | + | | + | | + | | | + | | + | | + | | + | | + | | + | | | + | | + | | + | | + | | + | | | + | | + | | + | | + | | + | + | **ABC-type branched-chain amino acid transport** |
| **SSGZ1_1382** | | | | | + | | + | | + | | + | | | + | | + | | + | | + | | + | | | + | | + | | + | | + | | + | | | + | | + | | + | | + | | + | | + | | | + | | + | | + | | + | | + | | | + | | + | | + | | + | | + | + | **hypothetical protein** |
| **SSGZ1_1383** | | | | | + | | + | | + | | + | | | + | | + | | + | | + | | + | | | + | | + | | + | | + | | + | | | + | | + | | + | | + | | + | | + | | | + | | + | | + | | + | | + | | | + | | + | | + | | + | | + | + | **ATP-dependent Clp protease, proteolytic subunit** |
| **SSGZ1_1384** | | | | | + | | + | | + | | + | | | + | | + | | + | | + | | + | | | + | | + | | + | | + | | + | | | + | | + | | + | | + | | + | | + | | | + | | + | | + | | + | | + | | | + | | + | | + | | + | | + | + | **uracil phosphoribosyltransferase** |
| **SSGZ1_1385** | | | | | + | | + | | + | | + | | | + | | + | | + | | + | | + | | | + | | + | | + | | + | | + | | | + | | + | | + | | + | | + | | + | | | + | | + | | + | | + | | + | | | + | | + | | + | | + | | + | + | **glucan 1,6-alpha-glucosidase** |
| **SSGZ1_1386** | | | | | + | | + | | + | | + | | | + | | + | | + | | + | | + | | | - | | + | | + | | + | | + | | | + | | + | | + | | + | | + | | + | | | - | | + | | + | | + | | + | | | + | | + | | + | | + | | + | + | **Sucrose phosphorylase** |
| **SSGZ1_1387** | | | | | + | | + | | + | | + | | | + | | + | | + | | + | | + | | | - | | + | | + | | + | | + | | | + | | + | | + | | + | | + | | + | | | - | | + | | + | | + | | + | | | + | | + | | + | | + | | + | + | **Binding-protein-dependent transport systems** |
| **SSGZ1_1388** | | | | | + | | + | | + | | + | | | + | | + | | + | | + | | + | | | - | | + | | + | | + | | + | | | + | | + | | + | | + | | + | | + | | | - | | + | | + | | + | | + | | | + | | + | | + | | + | | + | + | **Binding-protein-dependent transport systems** |
| **SSGZ1_1389** | | | | | + | | + | | + | | + | | | + | | + | | + | | + | | + | | | - | | + | | + | | + | | + | | | + | | + | | + | | + | | + | | + | | | - | | + | | + | | + | | + | | | + | | + | | + | | + | | + | + | **multiple sugar-binding ABC transporter,** |
| **SSGZ1_1390** | | | | | + | | + | | + | | + | | | + | | + | | + | | + | | + | | | - | | + | | + | | + | | + | | | + | | + | | + | | + | | + | | + | | | + | | + | | + | | + | | + | | | + | | + | | + | | + | | + | + | **alpha-galactosidase** |
| **SSGZ1_1391** | | | | | + | | + | | + | | + | | | + | | + | | + | | + | | + | | | - | | + | | + | | + | | + | | | + | | + | | + | | + | | + | | + | | | + | | + | | + | | + | | + | | | + | | + | | + | | + | | + | + | **putative MSM operon regulatory protein** |
| **SSGZ1_1392** | | | | | + | | + | | + | | + | | | + | | + | | + | | + | | + | | | + | | + | | + | | + | | + | | | + | | + | | + | | + | | + | | + | | | + | | + | | + | | + | | + | | | + | | + | | + | | + | | + | + | **aminotransferase** |
| **SSGZ1_1393** | | | | | + | | + | | + | | + | | | + | | + | | + | | + | | + | | | + | | + | | + | | + | | + | | | + | | + | | + | | + | | + | | + | | | + | | + | | + | | + | | + | | | + | | + | | + | | + | | + | + | **putative Cystathionine gamma-synthase** |
| **SSGZ1_1394** | | | | | + | | + | | + | | + | | | + | | + | | + | | + | | + | | | + | | + | | + | | + | | + | | | + | | + | | + | | + | | + | | + | | | + | | + | | + | | + | | + | | | + | | + | | + | | + | | + | + | **hypothetical protein** |
| **SSGZ1_1395** | | | | | + | | + | | + | | + | | | + | | + | | + | | + | | + | | | + | | + | | + | | + | | + | | | + | | + | | + | | + | | + | | + | | | + | | + | | + | | + | | + | | | + | | + | | + | | + | | + | + | **hypothetical protein** |
| **SSGZ1_1396** | | | | | + | | + | | + | | + | | | + | | + | | + | | + | | + | | | + | | + | | + | | + | | + | | | + | | + | | + | | + | | + | | + | | | + | | + | | + | | + | | + | | | + | | + | | + | | + | | + | + | **hypothetical protein** |
| **SSGZ1_1397** | | | | | + | | + | | + | | + | | | + | | + | | + | | + | | + | | | + | | + | | + | | + | | + | | | + | | + | | + | | + | | + | | + | | | + | | + | | + | | + | | + | | | + | | + | | + | | + | | + | + | **hypothetical protein** |
| **SSGZ1_1398** | | | | | + | | + | | + | | + | | | + | | + | | + | | + | | + | | | + | | + | | + | | + | | + | | | + | | + | | + | | + | | + | | + | | | + | | + | | + | | + | | + | | | + | | + | | + | | + | | + | + | **Predicted O-methyltransferase** |
| **SSGZ1_1399** | | | | | + | | + | | + | | + | | | + | | + | | + | | + | | + | | | + | | + | | + | | + | | + | | | + | | + | | + | | + | | + | | + | | | + | | + | | + | | + | | + | | | + | | + | | + | | + | | + | + | **Excinuclease ABC, C subunit, N-terminal** |
| **SSGZ1_1400** | | | | | - | | + | | + | | + | | | + | | + | | + | | + | | + | | | + | | - | | - | | - | | - | | | - | | + | | + | | + | | + | | + | | | + | | - | | - | | + | | + | | | + | | + | | + | | + | | + | + | **ion transport protein, putative** |
| **SSGZ1_1401** | | | | | + | | + | | + | | + | | | + | | + | | + | | + | | + | | | + | | + | | + | | + | | + | | | + | | + | | + | | + | | + | | + | | | + | | - | | + | | + | | + | | | + | | + | | + | | + | | + | + | **Acyl carrier protein phosphodiesterase** |
| **SSGZ1_1402** | | | | | + | | + | | + | | + | | | + | | + | | + | | + | | + | | | + | | + | | + | | + | | + | | | + | | + | | + | | + | | + | | + | | | + | | + | | + | | + | | + | | | + | | + | | + | | + | | + | + | **GCN5-related N-acetyltransferase** |
| **SSGZ1_1403** | | | | | + | | + | | + | | + | | | + | | + | | + | | + | | + | | | + | | + | | + | | + | | + | | | + | | + | | + | | + | | - | | + | | | + | | + | | + | | + | | + | | | + | | + | | + | | + | | + | + | **integral membrane protein** |
| **SSGZ1_1404** | | | | | + | | + | | + | | + | | | + | | + | | + | | + | | + | | | + | | + | | + | | + | | + | | | + | | + | | + | | + | | - | | + | | | + | | + | | + | | + | | + | | | + | | + | | + | | + | | + | + | **regulatory protein, ArsR** |
| **SSGZ1_1405** | | | | | + | | + | | + | | + | | | + | | + | | + | | + | | + | | | + | | + | | + | | + | | + | | | + | | + | | + | | + | | + | | + | | | + | | + | | + | | + | | + | | | + | | + | | + | | + | | + | + | **ATP-dependent RNA helicase, DEAD/DEAH box** |
| **SSGZ1_1406** | | | | | + | | + | | + | | + | | | + | | + | | + | | + | | + | | | + | | + | | + | | + | | + | | | + | | + | | + | | + | | + | | + | | | + | | + | | + | | + | | + | | | + | | - | | + | | + | | + | + | **YxkD** |
| **SSGZ1_1407** | | | | | + | | + | | + | | + | | | + | | + | | + | | + | | + | | | + | | + | | + | | + | | + | | | + | | + | | + | | + | | + | | + | | | + | | + | | + | | + | | + | | | + | | + | | + | | + | | + | + | **Superfamily I DNA and RNA helicase** |
| **SSGZ1_1408** | | | | | - | | + | | + | | + | | | + | | + | | + | | + | | + | | | + | | + | | + | | + | | + | | | + | | - | | + | | + | | + | | - | | | + | | + | | + | | + | | + | | | + | | - | | + | | + | | + | + | **Internalin protein** |
| **SSGZ1_1409** | | | | | + | | + | | + | | + | | | + | | + | | + | | + | | + | | | + | | + | | + | | + | | + | | | + | | + | | + | | + | | + | | + | | | + | | + | | + | | + | | + | | | + | | - | | + | | + | | + | + | **hypothetical protein** |
| **SSGZ1_1410** | | | | | + | | + | | + | | + | | | + | | + | | + | | + | | + | | | + | | + | | + | | + | | + | | | + | | + | | + | | + | | + | | + | | | + | | + | | + | | + | | + | | | + | | + | | + | | + | | + | + | **conserved hypothetical protein** |
| **SSGZ1_1411** | | | | | + | | + | | + | | + | | | + | | + | | + | | + | | + | | | + | | + | | + | | + | | + | | | + | | + | | + | | + | | + | | + | | | + | | + | | + | | + | | + | | | + | | + | | + | | + | | + | + | **pyridoxine kinase** |
| **SSGZ1_1412** | | | | | + | | + | | + | | + | | | + | | + | | + | | + | | + | | | + | | + | | + | | + | | + | | | + | | + | | + | | + | | + | | + | | | + | | + | | + | | + | | + | | | + | | + | | + | | + | | + | + | **Adenosylhomocysteine nucleosidase** |
| **SSGZ1_1413** | | | | | + | | + | | + | | + | | | + | | + | | + | | + | | + | | | + | | + | | + | | + | | + | | | + | | + | | + | | + | | + | | + | | | + | | + | | + | | + | | + | | | + | | + | | + | | + | | + | + | **conserved hypothetical protein** |
| **SSGZ1_1414** | | | | | + | | + | | + | | + | | | + | | + | | + | | + | | + | | | + | | + | | + | | + | | + | | | + | | + | | + | | + | | + | | + | | | + | | + | | + | | + | | + | | | + | | + | | + | | + | | + | + | **NUDIX hydrolase** |
| **SSGZ1_1415** | | | | | + | | + | | + | | + | | | + | | + | | + | | + | | + | | | + | | + | | + | | + | | + | | | + | | + | | + | | + | | + | | + | | | + | | + | | + | | + | | + | | | + | | + | | + | | + | | + | + | **UDP-N-acetylglucosamine pyrophosphorylase** |
| **SSGZ1_1416** | | | | | + | | + | | + | | + | | | + | | + | | + | | + | | + | | | + | | + | | + | | + | | + | | | + | | + | | + | | + | | + | | + | | | + | | + | | + | | + | | + | | | + | | + | | + | | + | | + | + | **conserved hypothetical protein** |
| **SSGZ1_1417** | | | | | + | | + | | + | | + | | | + | | + | | + | | + | | + | | | + | | + | | + | | + | | + | | | - | | + | | + | | + | | + | | + | | | + | | - | | + | | + | | + | | | + | | + | | + | | + | | + | + | **hypothetical protein** |
| **SSGZ1_1418** | | | | | + | | + | | + | | + | | | + | | + | | + | | + | | + | | | + | | + | | + | | + | | + | | | + | | + | | + | | + | | + | | + | | | + | | - | | + | | + | | + | | | + | | + | | + | | + | | + | + | **Short chain dehydrogenase** |
| **SSGZ1_1419** | | | | | + | | + | | + | | + | | | + | | + | | + | | + | | + | | | + | | + | | + | | + | | + | | | + | | + | | + | | + | | + | | + | | | + | | + | | + | | + | | + | | | + | | + | | + | | + | | + | + | **hypothetical protein** |
| **SSGZ1_1420** | | | | | - | | + | | + | | + | | | + | | + | | + | | + | | + | | | - | | - | | - | | - | | + | | | - | | + | | - | | - | | - | | + | | | + | | - | | - | | - | | - | | | - | | + | | - | | - | | - | + | **membrane protein, putative** |
| **SSGZ1_1421** | | | | | + | | + | | + | | + | | | + | | + | | + | | + | | + | | | + | | + | | + | | + | | + | | | + | | + | | + | | + | | + | | + | | | + | | - | | + | | + | | + | | | + | | + | | + | | + | | + | + | **transcriptional regulator, PadR family protein** |
| **SSGZ1_1422** | | | | | + | | + | | + | | + | | | + | | + | | + | | + | | + | | | + | | + | | + | | + | | + | | | + | | + | | + | | + | | + | | + | | | + | | - | | + | | + | | + | | | + | | + | | + | | + | | + | + | **hypothetical protein** |
| **SSGZ1_1423** | | | | | + | | + | | + | | + | | | + | | + | | + | | + | | + | | | + | | + | | + | | + | | + | | | + | | + | | + | | + | | + | | + | | | + | | - | | + | | + | | + | | | + | | + | | + | | + | | + | + | **transposase of IS200 family** |
| **SSGZ1_1424** | | | | | + | | + | | + | | + | | | + | | + | | + | | + | | + | | | + | | + | | + | | + | | + | | | + | | + | | + | | + | | + | | + | | | + | | + | | + | | + | | + | | | + | | + | | + | | + | | + | + | **putative permease protein** |
| **SSGZ1_1425** | | | | | + | | + | | + | | + | | | + | | + | | + | | + | | + | | | + | | + | | + | | + | | + | | | + | | + | | + | | + | | + | | + | | | + | | + | | + | | + | | + | | | + | | + | | + | | + | | + | + | **putative ABC-type bacitracin resistance protein** |
| **SSGZ1_1426** | | | | | + | | + | | + | | + | | | + | | + | | + | | + | | + | | | + | | + | | + | | + | | - | | | + | | + | | + | | + | | + | | + | | | + | | + | | + | | + | | + | | | + | | + | | + | | + | | + | + | **ATPase-like protein** |
| **SSGZ1_1427** | | | | | + | | + | | + | | + | | | + | | + | | + | | + | | + | | | + | | + | | + | | + | | - | | | + | | + | | + | | + | | + | | + | | | + | | + | | + | | + | | + | | | + | | + | | + | | + | | + | + | **putative regulator protein** |
| **SSGZ1_1428** | | | | | + | | + | | + | | + | | | + | | + | | + | | + | | + | | | + | | + | | + | | + | | + | | | + | | + | | + | | + | | + | | + | | | + | | + | | + | | + | | + | | | + | | + | | + | | + | | + | + | **hypothetical protein** |
| **SSGZ1_1429** | | | | | + | | + | | + | | + | | | + | | + | | + | | + | | + | | | + | | + | | + | | + | | + | | | + | | + | | + | | + | | + | | + | | | + | | + | | + | | + | | + | | | + | | + | | + | | + | | + | + | **Aminotransferase** |
| **SSGZ1_1430** | | | | | + | | + | | + | | + | | | + | | + | | + | | + | | + | | | + | | + | | + | | + | | + | | | + | | + | | + | | + | | + | | + | | | + | | - | | + | | + | | + | | | + | | + | | + | | + | | + | + | **hypothetical protein** |
| **SSGZ1_1431** | | | | | + | | + | | + | | + | | | + | | + | | + | | + | | + | | | + | | + | | + | | + | | + | | | + | | + | | + | | + | | + | | + | | | + | | + | | + | | + | | + | | | + | | + | | + | | + | | + | + | **putative osmoprotectant-binding protein** |
| **SSGZ1_1432** | | | | | + | | + | | + | | + | | | + | | + | | + | | + | | + | | | + | | + | | + | | + | | + | | | + | | + | | + | | + | | + | | + | | | + | | + | | + | | + | | + | | | + | | + | | + | | + | | + | + | **putative ATP-binding protein opuCA** |
| **SSGZ1_1433** | | | | | + | | + | | + | | + | | | + | | + | | + | | + | | + | | | + | | + | | + | | + | | + | | | + | | + | | + | | + | | + | | + | | | + | | + | | + | | + | | + | | | + | | + | | + | | + | | + | + | **regulatory protein, MarR** |
| **SSGZ1_1434** | | | | | + | | + | | + | | + | | | + | | + | | + | | + | | + | | | + | | + | | + | | + | | + | | | + | | + | | + | | + | | + | | + | | | + | | + | | + | | + | | + | | | + | | + | | + | | + | | + | + | **Signal recognition particle GTPase** |
| **SSGZ1_1435** | | | | | + | | + | | + | | + | | | + | | + | | + | | + | | + | | | + | | + | | + | | + | | + | | | + | | + | | + | | + | | + | | + | | | + | | + | | + | | + | | + | | | + | | + | | + | | + | | + | + | **Uncharacterized protein containing a divergent** |
| **SSGZ1_1436** | | | | | + | | + | | + | | + | | | + | | + | | + | | + | | + | | | + | | + | | + | | + | | + | | | + | | + | | + | | + | | + | | + | | | + | | + | | + | | + | | + | | | + | | + | | + | | + | | + | + | **Prolipoprotein diacylglyceryl transferase** |
| **SSGZ1_1437** | | | | | + | | + | | + | | + | | | + | | + | | + | | + | | + | | | + | | + | | + | | + | | + | | | + | | + | | + | | + | | + | | + | | | + | | + | | + | | + | | + | | | + | | + | | + | | + | | + | + | **HPr(Ser) kinase** |
| **SSGZ1_1438** | | | | | + | | + | | + | | + | | | + | | + | | + | | + | | + | | | + | | + | | + | | + | | + | | | + | | + | | + | | + | | + | | + | | | + | | + | | + | | + | | + | | | + | | + | | + | | + | | + | + | **Mannose-6-phosphate isomerase** |
| **SSGZ1_1439** | | | | | + | | + | | + | | + | | | + | | + | | + | | + | | + | | | + | | + | | + | | + | | + | | | - | | + | | + | | + | | + | | + | | | + | | + | | + | | + | | + | | | + | | + | | + | | + | | + | + | **Iron-containing alcohol dehydrogenase** |
| **SSGZ1_1440** | | | | | + | | + | | + | | + | | | + | | + | | + | | + | | + | | | + | | + | | + | | + | | + | | | - | | + | | + | | + | | - | | + | | | + | | + | | - | | + | | + | | | + | | + | | + | | - | | + | + | **IS630-Spn1, transposase Orf1** |
| **SSGZ1_1441** | | | | | + | | + | | + | | + | | | + | | + | | + | | + | | + | | | + | | + | | + | | + | | - | | | - | | - | | - | | - | | - | | - | | | + | | + | | - | | + | | + | | | + | | + | | + | | - | | - | + | **IS630-Spn1, transposase Orf2** |
| **SSGZ1_1442** | | | | | + | | + | | + | | + | | | + | | + | | + | | + | | + | | | + | | + | | + | | + | | + | | | + | | + | | + | | + | | + | | + | | | + | | + | | + | | + | | + | | | + | | + | | + | | + | | + | + | **hypothetical protein** |
| **SSGZ1_1443** | | | | | + | | + | | + | | + | | | + | | + | | + | | + | | + | | | + | | + | | + | | + | | + | | | + | | + | | + | | + | | + | | + | | | + | | + | | + | | + | | + | | | + | | + | | + | | + | | + | + | **hypothetical protein** |
| **SSGZ1_1444** | | | | | + | | + | | + | | + | | | + | | + | | + | | + | | + | | | + | | + | | + | | + | | + | | | + | | + | | + | | + | | + | | + | | | + | | + | | + | | + | | + | | | + | | + | | + | | + | | + | + | **RNA binding S1** |
| **SSGZ1_1445** | | | | | + | | + | | + | | + | | | + | | + | | + | | + | | + | | | + | | + | | + | | + | | + | | | + | | + | | + | | + | | + | | + | | | + | | + | | + | | + | | + | | | + | | + | | + | | + | | + | + | **putative reductase** |
| **SSGZ1_1446** | | | | | + | | + | | + | | + | | | + | | + | | + | | + | | + | | | + | | + | | + | | + | | + | | | + | | + | | + | | + | | + | | + | | | + | | + | | + | | + | | + | | | + | | + | | + | | + | | + | + | **hypothetical protein** |
| **SSGZ1_1447** | | | | | + | | + | | + | | + | | | + | | + | | + | | + | | + | | | + | | + | | + | | + | | + | | | + | | + | | + | | + | | + | | + | | | + | | + | | + | | + | | + | | | + | | + | | + | | + | | + | + | **GtrA-like protein** |
| **SSGZ1_1448** | | | | | + | | + | | + | | + | | | + | | + | | + | | + | | + | | | + | | + | | + | | + | | + | | | + | | + | | + | | + | | + | | + | | | + | | + | | + | | + | | + | | | + | | + | | + | | + | | + | + | **RNase BN** |
| **SSGZ1_1449** | | | | | + | | + | | + | | + | | | + | | + | | + | | + | | + | | | + | | + | | + | | + | | + | | | + | | + | | + | | + | | + | | + | | | + | | + | | + | | + | | + | | | + | | + | | + | | + | | + | + | **methionine aminopeptidase** |
| **SSGZ1_1450** | | | | | + | | + | | + | | + | | | + | | + | | + | | + | | + | | | + | | + | | + | | + | | + | | | + | | + | | + | | + | | + | | + | | | + | | + | | + | | + | | + | | | + | | + | | + | | + | | + | + | **GCN5-related N-acetyltransferase** |
| **SSGZ1_1451** | | | | | + | | + | | + | | + | | | + | | + | | + | | + | | + | | | + | | + | | + | | + | | + | | | + | | + | | + | | + | | + | | + | | | + | | + | | + | | + | | + | | | + | | + | | + | | + | | + | + | **UDP-N-acetylglucosamine enolpyruvyl transferase** |
| **SSGZ1_1452** | | | | | + | | + | | + | | + | | | + | | + | | + | | + | | + | | | + | | + | | + | | + | | + | | | + | | + | | + | | - | | + | | - | | | + | | + | | + | | + | | + | | | + | | - | | + | | + | | + | - | **Putative NAD(P)H oxidoreductase** |
| **SSGZ1_1453** | | | | | + | | + | | + | | + | | | + | | + | | + | | + | | + | | | + | | + | | + | | + | | + | | | + | | + | | + | | + | | + | | + | | | + | | + | | + | | + | | + | | | + | | + | | + | | + | | + | + | **S-adenosylmethionine synthetase** |
| **SSGZ1_1454** | | | | | + | | + | | + | | + | | | + | | + | | + | | + | | + | | | + | | + | | + | | + | | + | | | + | | + | | + | | + | | + | | + | | | + | | + | | + | | + | | + | | | + | | + | | + | | + | | + | + | **Deoxycytidylate deaminase** |
| **SSGZ1_1455** | | | | | + | | + | | + | | + | | | + | | + | | + | | + | | + | | | + | | + | | + | | + | | + | | | + | | + | | + | | + | | + | | + | | | + | | + | | + | | + | | + | | | + | | + | | + | | + | | + | + | **hypothetical protein** |
| **SSGZ1_1456** | | | | | + | | + | | + | | + | | | + | | + | | + | | + | | + | | | + | | + | | + | | + | | + | | | + | | + | | + | | + | | + | | + | | | + | | + | | + | | + | | + | | | + | | + | | + | | + | | + | + | **Peptidase M42** |
| **SSGZ1_1457** | | | | | + | | + | | + | | + | | | + | | + | | + | | + | | + | | | + | | + | | + | | + | | + | | | + | | + | | + | | + | | + | | + | | | + | | + | | + | | + | | + | | | + | | + | | + | | + | | + | + | **Biotin--acetyl-CoA-carboxylase ligase** |
| **SSGZ1_1458** | | | | | + | | + | | + | | + | | | + | | + | | + | | + | | + | | | + | | + | | + | | + | | + | | | + | | + | | + | | + | | + | | + | | | + | | + | | + | | + | | + | | | + | | + | | + | | + | | + | + | **hypothetical protein** |
| **SSGZ1_1459** | | | | | + | | + | | + | | + | | | + | | + | | + | | + | | + | | | + | | + | | + | | + | | + | | | + | | + | | + | | + | | + | | + | | | + | | + | | + | | + | | + | | | + | | + | | + | | + | | + | + | **DNA-directed DNA polymerase** |
| **SSGZ1_1460** | | | | | + | | + | | + | | + | | | + | | + | | + | | + | | + | | | + | | + | | + | | + | | + | | | + | | + | | + | | + | | + | | + | | | + | | + | | + | | + | | + | | | + | | + | | + | | + | | + | + | **Arginyl-tRNA synthetase** |
| **SSGZ1_1461** | | | | | + | | + | | + | | + | | | + | | + | | + | | + | | + | | | + | | + | | + | | + | | + | | | + | | + | | + | | + | | + | | + | | | + | | + | | + | | + | | + | | | + | | + | | + | | + | | + | + | **hypothetical protein** |
| **SSGZ1_1462** | | | | | + | | + | | + | | + | | | + | | + | | + | | + | | + | | | + | | + | | + | | + | | + | | | + | | + | | + | | + | | + | | + | | | + | | + | | + | | + | | + | | | + | | + | | + | | + | | + | + | **Transcriptional regulator protein** |
| **SSGZ1_1463** | | | | | + | | + | | + | | + | | | + | | + | | + | | + | | + | | | + | | + | | + | | + | | + | | | + | | + | | + | | + | | + | | + | | | + | | + | | + | | + | | + | | | + | | + | | + | | + | | + | + | **uridine kinase** |
| **SSGZ1_1464** | | | | | + | | + | | + | | + | | | + | | + | | + | | + | | + | | | + | | + | | + | | + | | + | | | + | | + | | + | | + | | + | | + | | | + | | + | | + | | + | | + | | | + | | + | | + | | + | | + | + | **Oxidoreductase, N-terminal** |
| **SSGZ1_1465** | | | | | + | | + | | + | | + | | | + | | + | | + | | + | | + | | | + | | + | | + | | + | | + | | | + | | + | | + | | + | | + | | + | | | + | | + | | + | | + | | + | | | + | | + | | + | | + | | + | + | **Predicted dehydrogenase and related proteins** |
| **SSGZ1_1466** | | | | | + | | + | | + | | + | | | + | | + | | + | | + | | + | | | + | | + | | + | | + | | + | | | + | | + | | + | | + | | + | | + | | | + | | + | | + | | + | | + | | | + | | + | | + | | + | | + | + | **helicase** |
| **SSGZ1_1467** | | | | | + | | + | | + | | + | | | + | | + | | + | | + | | + | | | + | | + | | + | | + | | + | | | + | | + | | + | | + | | + | | + | | | + | | + | | + | | + | | + | | | + | | + | | + | | + | | + | + | **Polysaccharide deacetylase** |
| **SSGZ1_1468** | | | | | + | | + | | + | | + | | | + | | + | | + | | + | | + | | | + | | + | | + | | + | | + | | | + | | + | | + | | + | | + | | + | | | + | | + | | + | | + | | + | | | + | | + | | + | | + | | + | + | **hypothetical protein** |
| **SSGZ1_1469** | | | | | + | | + | | + | | + | | | + | | + | | + | | + | | + | | | + | | + | | + | | + | | + | | | + | | + | | + | | + | | + | | + | | | + | | + | | + | | + | | + | | | + | | + | | + | | + | | + | + | **Lactoylglutathione lyase** |
| **SSGZ1_1470** | | | | | + | | + | | + | | + | | | + | | + | | + | | + | | + | | | + | | + | | - | | - | | + | | | + | | + | | + | | + | | + | | + | | | + | | - | | + | | + | | + | | | + | | + | | + | | + | | + | + | **transposase of IS200 family** |
| **SSGZ1_1471** | | | | | + | | + | | + | | + | | | + | | + | | + | | + | | + | | | + | | + | | + | | + | | + | | | + | | + | | + | | + | | + | | + | | | + | | + | | + | | + | | + | | | + | | + | | + | | + | | + | + | **Phosphoglycerate mutase 1** |
| **SSGZ1_1472** | | | | | + | | + | | + | | + | | | + | | + | | + | | + | | + | | | + | | + | | + | | + | | + | | | + | | + | | + | | + | | + | | + | | | + | | + | | + | | + | | + | | | + | | + | | + | | + | | + | + | **Thiolase** |
| **SSGZ1_1473** | | | | | + | | + | | + | | + | | | + | | + | | + | | + | | + | | | + | | + | | + | | + | | + | | | + | | + | | + | | + | | + | | + | | | + | | + | | + | | + | | + | | | + | | + | | + | | + | | + | + | **Hydroxymethylglutaryl-coenzyme A synthase,** |
| **SSGZ1_1474** | | | | | + | | + | | + | | + | | | + | | + | | + | | + | | + | | | + | | + | | + | | + | | + | | | + | | + | | + | | + | | + | | + | | | + | | + | | + | | + | | + | | | + | | + | | + | | + | | + | + | **Hydroxymethylglutaryl-CoA reductase** |
| **SSGZ1_1475** | | | | | + | | + | | + | | + | | | + | | + | | + | | + | | + | | | + | | + | | + | | + | | + | | | - | | - | | + | | + | | + | | + | | | + | | + | | + | | + | | + | | | + | | + | | + | | + | | + | + | **Transposase** |
| **SSGZ1_1476** | | | | | + | | + | | + | | + | | | + | | + | | + | | + | | + | | | + | | + | | + | | + | | + | | | + | | + | | + | | + | | + | | + | | | + | | + | | + | | + | | + | | | + | | + | | + | | + | | + | + | **GCN5-related N-acetyltransferase** |
| **SSGZ1_1477** | | | | | + | | + | | + | | + | | | + | | + | | + | | + | | + | | | + | | + | | + | | + | | + | | | + | | + | | + | | + | | + | | - | | | + | | - | | + | | + | | + | | | + | | + | | + | | + | | + | + | **Transposase** |
| **SSGZ1_1478** | | | | | + | | + | | + | | + | | | + | | + | | + | | + | | + | | | + | | + | | + | | + | | + | | | + | | + | | + | | + | | + | | + | | | + | | + | | + | | + | | + | | | + | | + | | + | | + | | + | + | **Histone-like bacterial DNA-binding protein** |
| **SSGZ1_1479** | | | | | + | | + | | + | | + | | | + | | + | | + | | + | | + | | | + | | + | | + | | + | | + | | | + | | + | | + | | + | | + | | + | | | + | | + | | + | | + | | + | | | + | | + | | + | | + | | + | + | **conserved hypothetical protein** |
| **SSGZ1_1480** | | | | | + | | + | | + | | + | | | + | | + | | + | | + | | + | | | + | | + | | + | | + | | + | | | + | | + | | + | | + | | + | | + | | | + | | + | | + | | + | | + | | | + | | + | | + | | + | | + | + | **Lysophospholipase L1 and related esterases** |
| **SSGZ1_1481** | | | | | + | | + | | + | | + | | | + | | + | | + | | + | | + | | | + | | + | | + | | + | | + | | | + | | + | | + | | + | | + | | + | | | + | | + | | + | | + | | + | | | + | | + | | + | | + | | + | + | **DegV** |
| **SSGZ1_1482** | | | | | + | | + | | + | | + | | | + | | + | | + | | + | | + | | | + | | + | | + | | + | | + | | | + | | + | | + | | + | | + | | + | | | + | | + | | + | | + | | + | | | + | | + | | + | | + | | + | + | **DNA repair protein RecN** |
| **SSGZ1_1483** | | | | | + | | + | | + | | + | | | + | | + | | + | | + | | + | | | + | | + | | + | | + | | + | | | + | | + | | + | | + | | + | | + | | | + | | + | | + | | + | | + | | | + | | + | | + | | + | | + | + | **Arginine repressor** |
| **SSGZ1_1484** | | | | | + | | + | | + | | + | | | + | | + | | + | | + | | + | | | + | | + | | + | | + | | + | | | + | | + | | + | | + | | + | | + | | | + | | + | | + | | + | | + | | | + | | + | | + | | + | | + | + | **Hemolysin A** |
| **SSGZ1_1485** | | | | | + | | + | | + | | + | | | + | | + | | + | | + | | + | | | + | | + | | + | | + | | + | | | + | | + | | + | | + | | + | | + | | | + | | + | | + | | + | | - | | | + | | + | | + | | + | | + | + | **Polyprenyl synthetase** |
| **SSGZ1_1486** | | | | | + | | + | | + | | + | | | + | | + | | + | | + | | + | | | + | | + | | + | | + | | + | | | + | | + | | + | | + | | + | | + | | | + | | + | | + | | + | | + | | | + | | + | | + | | + | | + | + | **Exonuclease VII, small subunit** |
| **SSGZ1_1487** | | | | | + | | + | | + | | + | | | + | | + | | + | | + | | + | | | + | | + | | + | | + | | + | | | + | | + | | + | | + | | + | | + | | | + | | + | | + | | + | | + | | | + | | + | | + | | + | | + | + | **Exonuclease VII, large subunit** |
| **SSGZ1_1488** | | | | | + | | + | | + | | + | | | + | | + | | + | | + | | + | | | + | | + | | + | | + | | + | | | + | | + | | + | | + | | + | | + | | | + | | + | | + | | + | | + | | | + | | + | | + | | + | | + | + | **Metallophosphoesterase** |
| **SSGZ1_1489** | | | | | + | | + | | + | | + | | | + | | + | | + | | + | | + | | | + | | + | | + | | + | | + | | | + | | + | | + | | + | | + | | + | | | + | | + | | + | | + | | + | | | + | | + | | + | | + | | + | + | **hypothetical protein** |
| **SSGZ1_1490** | | | | | + | | + | | + | | + | | | + | | + | | + | | + | | + | | | + | | + | | + | | + | | + | | | + | | + | | + | | + | | + | | + | | | + | | + | | + | | + | | + | | | + | | + | | + | | + | | + | + | **Isoprenylcysteine carboxyl methyltransferase** |
| **SSGZ1_1491** | | | | | + | | + | | + | | + | | | + | | + | | + | | + | | + | | | + | | + | | + | | + | | + | | | + | | + | | + | | + | | + | | + | | | + | | + | | + | | + | | + | | | + | | + | | + | | + | | + | + | **two-component sensor** |
| **SSGZ1_1492** | | | | | + | | + | | + | | + | | | + | | + | | + | | + | | + | | | + | | + | | + | | + | | + | | | + | | + | | + | | + | | + | | + | | | + | | + | | + | | + | | + | | | + | | + | | + | | + | | + | + | **two-component regulator** |
| **SSGZ1_1493** | | | | | + | | + | | + | | + | | | + | | + | | + | | + | | + | | | + | | + | | + | | + | | + | | | + | | + | | + | | + | | + | | + | | | + | | + | | + | | + | | + | | | + | | + | | + | | + | | + | + | **Abortive infection protein** |
| **SSGZ1_1494** | | | | | + | | + | | + | | + | | | + | | + | | + | | + | | + | | | + | | + | | + | | + | | + | | | + | | - | | + | | + | | + | | - | | | + | | + | | + | | + | | + | | | + | | - | | + | | + | | + | + | **serum opacity factor** |
| **SSGZ1_1495** | | | | | + | | + | | + | | + | | | + | | + | | + | | + | | + | | | + | | + | | + | | + | | + | | | + | | - | | + | | + | | + | | - | | | + | | - | | + | | + | | + | | | + | | - | | + | | + | | + | + | **von Willebrand factor, type A** |
| **SSGZ1_1496** | | | | | + | | + | | + | | + | | | + | | + | | + | | + | | + | | | + | | + | | + | | + | | + | | | + | | - | | + | | + | | + | | - | | | + | | + | | + | | + | | + | | | + | | - | | + | | + | | + | + | **YSIRK Gram-positive signal peptide** |
| **SSGZ1_1497** | | | | | + | | + | | + | | + | | | + | | + | | + | | + | | + | | | + | | + | | + | | + | | + | | | + | | + | | + | | + | | + | | + | | | + | | + | | + | | + | | + | | | + | | + | | + | | + | | + | + | **Inorganic diphosphatase** |
| **SSGZ1_1498** | | | | | + | | + | | + | | + | | | + | | + | | + | | + | | + | | | + | | + | | + | | + | | + | | | + | | + | | + | | + | | + | | + | | | + | | + | | + | | + | | + | | | + | | + | | + | | + | | + | + | **probable oligoendopeptidase F** |
| **SSGZ1_1499** | | | | | + | | + | | + | | + | | | + | | + | | + | | + | | + | | | + | | + | | + | | + | | + | | | + | | + | | + | | + | | + | | + | | | + | | + | | + | | + | | + | | | + | | + | | + | | + | | + | + | **pyruvate-formate lyase activating enzyme** |
| **SSGZ1_1500** | | | | | + | | + | | + | | + | | | + | | + | | + | | + | | + | | | + | | + | | + | | + | | + | | | + | | + | | + | | + | | + | | + | | | + | | + | | + | | + | | + | | | + | | + | | + | | + | | + | + | **CBS:Protein of unknown function** |
| **SSGZ1_1501** | | | | | + | | + | | + | | + | | | + | | + | | + | | + | | + | | | + | | + | | + | | + | | + | | | + | | + | | + | | + | | + | | + | | | + | | + | | + | | + | | + | | | + | | + | | + | | + | | + | + | **ABC-type molybdenum transport system, ATPase** |
| **SSGZ1_1502** | | | | | + | | + | | + | | + | | | + | | + | | + | | + | | + | | | + | | + | | + | | + | | + | | | + | | + | | + | | + | | + | | + | | | + | | + | | + | | + | | + | | | + | | + | | + | | + | | + | + | **Putative rRNA methylase** |
| **SSGZ1_1503** | | | | | + | | + | | + | | + | | | + | | + | | + | | + | | + | | | + | | + | | + | | + | | + | | | + | | + | | + | | + | | + | | + | | | + | | + | | + | | + | | + | | | + | | + | | + | | + | | + | + | **Type II secretory pathway, prepilin signal** |
| **SSGZ1_1504** | | | | | + | | + | | + | | + | | | + | | + | | + | | + | | + | | | + | | + | | + | | + | | + | | | + | | + | | + | | + | | + | | + | | | + | | + | | + | | + | | + | | | + | | + | | + | | + | | + | + | **Nicotinate phosphoribosyltransferase related** |
| **SSGZ1_1505** | | | | | + | | + | | + | | + | | | + | | + | | + | | + | | + | | | + | | + | | + | | + | | + | | | + | | + | | + | | + | | + | | + | | | + | | + | | + | | + | | + | | | + | | + | | + | | + | | + | + | **NAD+ synthase** |
| **SSGZ1_1506** | | | | | + | | + | | + | | + | | | + | | + | | + | | + | | + | | | + | | + | | + | | + | | + | | | + | | + | | + | | + | | + | | + | | | + | | + | | + | | + | | + | | | - | | + | | + | | + | | + | + | **VanZ like protein** |
| **SSGZ1_1507** | | | | | + | | + | | + | | + | | | + | | + | | + | | + | | + | | | + | | + | | + | | + | | + | | | + | | + | | + | | + | | + | | + | | | + | | + | | + | | + | | + | | | + | | + | | + | | + | | + | + | **Predicted Fe-S-cluster redox enzyme** |
| **SSGZ1_1508** | | | | | + | | + | | + | | + | | | + | | + | | + | | + | | + | | | + | | + | | + | | + | | + | | | + | | + | | + | | + | | + | | + | | | + | | + | | + | | + | | + | | | + | | + | | + | | + | | + | + | **hypothetical protein** |
| **SSGZ1_1509** | | | | | + | | + | | + | | + | | | + | | + | | + | | + | | + | | | + | | + | | + | | + | | + | | | + | | + | | + | | + | | + | | + | | | + | | + | | + | | + | | + | | | + | | + | | + | | + | | + | + | **Predicted secreted protein containing a PDZ** |
| **SSGZ1_1510** | | | | | + | | + | | + | | + | | | + | | + | | + | | + | | + | | | + | | + | | + | | + | | + | | | + | | + | | + | | + | | + | | + | | | + | | + | | + | | + | | + | | | + | | + | | + | | + | | + | + | **lipopolysaccharide core biosynthesis protein** |
| **SSGZ1_1511** | | | | | + | | + | | + | | + | | | + | | + | | + | | + | | + | | | + | | + | | + | | + | | + | | | + | | + | | + | | + | | + | | + | | | + | | + | | + | | + | | + | | | + | | + | | + | | + | | + | + | **Hypothetical protein** |
| **SSGZ1_1512** | | | | | + | | + | | + | | + | | | + | | + | | + | | + | | + | | | + | | + | | + | | + | | + | | | + | | + | | + | | + | | + | | + | | | + | | + | | + | | + | | + | | | + | | + | | + | | + | | + | + | **extracellular serine protease** |
| **SSGZ1_1513** | | | | | + | | + | | + | | + | | | + | | + | | + | | + | | + | | | + | | + | | + | | + | | + | | | + | | + | | + | | + | | + | | + | | | + | | + | | + | | + | | + | | | + | | + | | + | | + | | + | + | **Rhodanese-related sulfurtransferase** |
| **SSGZ1_1514** | | | | | + | | + | | + | | + | | | + | | + | | + | | + | | + | | | + | | + | | + | | + | | + | | | + | | + | | + | | + | | + | | + | | | + | | + | | + | | + | | + | | | + | | + | | + | | + | | + | + | **hypothetical protein** |
| **SSGZ1_1515** | | | | | + | | + | | + | | + | | | + | | + | | + | | + | | + | | | + | | + | | + | | + | | + | | | + | | + | | + | | + | | + | | + | | | + | | + | | + | | + | | + | | | + | | + | | + | | + | | + | + | **Response regulator** |
| **SSGZ1_1516** | | | | | + | | + | | + | | + | | | + | | + | | + | | + | | + | | | + | | + | | + | | + | | + | | | + | | + | | + | | + | | + | | + | | | + | | + | | + | | + | | + | | | + | | + | | + | | + | | + | + | **putative sensor histidine kinase** |
| **SSGZ1_1517** | | | | | + | | + | | + | | + | | | + | | + | | + | | + | | + | | | + | | + | | + | | + | | + | | | + | | + | | + | | + | | + | | + | | | + | | - | | + | | + | | + | | | + | | + | | + | | + | | + | + | **putative ABC transporter, ATP-binding protein** |
| **SSGZ1_1518** | | | | | + | | + | | + | | + | | | + | | + | | + | | + | | + | | | + | | + | | + | | + | | + | | | + | | + | | + | | + | | + | | + | | | + | | + | | + | | + | | + | | | + | | + | | + | | + | | + | + | **putative ATPase** |
| **SSGZ1_1519** | | | | | + | | + | | + | | + | | | + | | + | | + | | + | | + | | | + | | + | | + | | + | | + | | | + | | + | | + | | + | | + | | + | | | + | | + | | + | | + | | + | | | + | | + | | + | | + | | + | + | **Dpr protein** |
| **SSGZ1_1520** | | | | | + | | + | | + | | + | | | + | | + | | + | | + | | + | | | + | | + | | + | | + | | + | | | + | | + | | + | | + | | + | | + | | | + | | + | | + | | + | | + | | | + | | + | | + | | + | | + | + | **MF3-like protein** |
| **SSGZ1_1521** | | | | | + | | + | | + | | + | | | + | | + | | + | | + | | + | | | + | | + | | + | | + | | + | | | + | | + | | + | | + | | + | | + | | | + | | + | | + | | + | | + | | | + | | + | | + | | + | | + | + | **putative phosphotyrosine protein phosphatase** |
| **SSGZ1_1522** | | | | | + | | + | | + | | + | | | + | | + | | + | | + | | + | | | + | | + | | + | | + | | + | | | + | | + | | + | | + | | + | | + | | | + | | + | | + | | + | | + | | | + | | + | | + | | + | | + | + | **hypothetical protein** |
| **SSGZ1_1523** | | | | | + | | + | | + | | + | | | + | | + | | + | | + | | + | | | + | | + | | + | | + | | + | | | + | | + | | + | | + | | + | | + | | | + | | + | | + | | + | | + | | | + | | + | | + | | + | | + | + | **Acyltransferase 3** |
| **SSGZ1_1524** | | | | | + | | + | | + | | + | | | + | | + | | + | | + | | + | | | + | | + | | + | | + | | + | | | + | | + | | + | | + | | + | | + | | | + | | + | | + | | + | | + | | | + | | + | | + | | + | | + | + | **Phosphoesterase, PA-phosphatase related** |
| **SSGZ1_1525** | | | | | + | | + | | + | | + | | | + | | + | | + | | + | | + | | | + | | + | | + | | + | | + | | | + | | + | | + | | + | | + | | + | | | + | | + | | + | | + | | + | | | + | | + | | + | | + | | + | + | **Predicted membrane protein** |
| **SSGZ1_1526** | | | | | + | | + | | + | | + | | | + | | + | | + | | + | | + | | | + | | + | | + | | + | | + | | | + | | + | | + | | + | | + | | + | | | + | | + | | + | | + | | + | | | + | | + | | + | | + | | + | + | **hypothetical protein** |
| **SSGZ1_1527** | | | | | + | | + | | + | | + | | | + | | + | | + | | + | | + | | | + | | + | | + | | + | | + | | | + | | + | | + | | + | | + | | + | | | + | | + | | + | | + | | + | | | + | | + | | + | | + | | + | + | **rRNA methyltransferase, TrmH family** |
| **SSGZ1_1528** | | | | | + | | + | | + | | + | | | + | | + | | + | | + | | + | | | + | | + | | + | | + | | + | | | + | | + | | + | | + | | + | | + | | | + | | + | | + | | + | | + | | | + | | + | | + | | + | | + | + | **hypothetical protein** |
| **SSGZ1_1529** | | | | | + | | + | | + | | + | | | + | | + | | + | | + | | + | | | + | | + | | + | | + | | + | | | + | | + | | + | | + | | + | | + | | | + | | - | | + | | + | | + | | | + | | + | | + | | + | | + | + | **Protein of unknown function DUF37** |
| **SSGZ1_1530** | | | | | + | | + | | + | | + | | | + | | + | | + | | + | | + | | | + | | + | | + | | + | | + | | | + | | + | | + | | + | | + | | + | | | + | | + | | + | | + | | + | | | + | | + | | + | | + | | + | + | **Pseudouridine synthase, Rsu** |
| **SSGZ1_1531** | | | | | + | | + | | + | | + | | | + | | + | | + | | + | | + | | | + | | + | | + | | + | | + | | | + | | + | | + | | + | | + | | + | | | + | | + | | + | | + | | + | | | + | | + | | + | | + | | + | + | **Predicted transcriptional regulator containing** |
| **SSGZ1_1532** | | | | | + | | + | | + | | + | | | + | | + | | + | | + | | + | | | + | | + | | + | | + | | + | | | + | | + | | + | | + | | + | | + | | | + | | + | | + | | + | | + | | | + | | + | | + | | + | | + | + | **Segregation and condensation protein A,** |
| **SSGZ1_1533** | | | | | + | | + | | + | | + | | | + | | + | | + | | + | | + | | | + | | + | | + | | + | | + | | | + | | + | | + | | + | | + | | + | | | + | | + | | + | | + | | + | | | + | | + | | + | | + | | + | + | **putative phage integrase** |
| **SSGZ1_1534** | | | | | + | | + | | + | | + | | | + | | + | | + | | + | | + | | | + | | + | | + | | + | | + | | | + | | + | | + | | + | | + | | + | | | + | | + | | + | | + | | + | | | + | | + | | + | | + | | + | + | **CBS domain protein** |
| **SSGZ1_1535** | | | | | + | | + | | + | | + | | | + | | + | | + | | + | | + | | | + | | + | | + | | + | | + | | | + | | + | | + | | + | | + | | + | | | + | | - | | + | | + | | + | | | + | | + | | + | | + | | + | + | **Predicted phosphoesterase** |
| **SSGZ1_1536** | | | | | + | | + | | + | | + | | | + | | + | | + | | + | | + | | | + | | + | | + | | + | | + | | | + | | + | | + | | + | | + | | + | | | + | | + | | + | | + | | + | | | + | | + | | + | | + | | + | + | **Ham1-like protein** |
| **SSGZ1_1537** | | | | | + | | + | | + | | + | | | + | | + | | + | | + | | + | | | + | | + | | + | | + | | + | | | + | | + | | + | | + | | + | | + | | | + | | + | | + | | + | | + | | | + | | + | | + | | + | | + | + | **Glutamate racemase** |
| **SSGZ1_1538** | | | | | + | | + | | + | | + | | | + | | + | | + | | + | | + | | | + | | + | | + | | + | | + | | | + | | + | | + | | + | | + | | + | | | + | | + | | + | | + | | + | | | + | | + | | + | | + | | + | + | **hypothetical protein** |
| **SSGZ1_1539** | | | | | + | | + | | + | | + | | | + | | + | | + | | + | | + | | | + | | + | | + | | + | | + | | | + | | + | | + | | + | | + | | + | | | + | | + | | + | | + | | + | | | + | | + | | + | | + | | + | + | **putative diaminopimelate decarboxylase** |
| **SSGZ1_1540** | | | | | + | | + | | + | | + | | | + | | + | | + | | + | | + | | | + | | + | | + | | + | | + | | | + | | + | | + | | + | | + | | + | | | + | | + | | + | | + | | + | | | + | | + | | + | | + | | + | + | **cysteine aminopeptidase C** |
| **SSGZ1_1541** | | | | | + | | + | | + | | + | | | + | | + | | + | | + | | + | | | + | | + | | + | | + | | + | | | + | | + | | + | | + | | + | | + | | | + | | + | | + | | + | | + | | | + | | + | | + | | + | | + | + | **membrane protein** |
| **SSGZ1_1542** | | | | | + | | + | | + | | + | | | + | | + | | + | | + | | + | | | + | | + | | + | | + | | + | | | + | | + | | + | | + | | + | | + | | | + | | + | | + | | + | | + | | | + | | + | | + | | + | | + | + | **hypothetical protein** |
| **SSGZ1_1543** | | | | | + | | + | | + | | + | | | + | | + | | + | | + | | + | | | + | | + | | + | | + | | + | | | + | | + | | + | | + | | + | | + | | | + | | - | | + | | + | | + | | | + | | + | | + | | + | | + | + | **rRNA methyltransferase** |
| **SSGZ1_1544** | | | | | + | | + | | - | | + | | | + | | + | | + | | + | | + | | | + | | + | | + | | + | | + | | | + | | + | | + | | + | | + | | + | | | - | | - | | + | | + | | + | | | + | | + | | + | | + | | + | + | **Acylphosphatase** |
| **SSGZ1_1545** | | | | | + | | - | | - | | - | | | - | | - | | - | | - | | - | | | + | | - | | + | | - | | + | | | - | | + | | + | | + | | + | | + | | | - | | + | | + | | + | | + | | | + | | + | | + | | + | | + | + | **putative inner membrane protein** |
| **SSGZ1_1546** | | | | | + | | + | | - | | + | | | + | | + | | + | | + | | + | | | + | | + | | + | | + | | + | | | + | | + | | + | | + | | + | | + | | | + | | + | | + | | + | | + | | | + | | + | | + | | + | | + | + | **Prokaryotic transcription elongation factor** |
| **SSGZ1_1547** | | | | | + | | + | | + | | + | | | + | | + | | + | | + | | + | | | + | | + | | + | | + | | + | | | + | | + | | + | | + | | + | | + | | | + | | + | | + | | + | | + | | | + | | + | | + | | + | | + | + | **Predicted periplasmic solute-binding protein** |
| **SSGZ1_1548** | | | | | + | | + | | + | | + | | | + | | + | | + | | + | | + | | | + | | + | | + | | + | | + | | | + | | + | | + | | + | | + | | + | | | + | | + | | + | | + | | + | | | + | | + | | + | | + | | + | + | **GCN5-related N-acetyltransferase** |
| **SSGZ1_1549** | | | | | + | | + | | + | | + | | | + | | + | | + | | + | | + | | | + | | + | | + | | + | | + | | | + | | + | | + | | + | | + | | + | | | + | | + | | + | | + | | + | | | + | | + | | + | | + | | + | + | **UDP-N-acetylmuramate--L-alanine ligase** |
| **SSGZ1_1550** | | | | | + | | + | | + | | + | | | + | | + | | + | | + | | + | | | + | | + | | + | | + | | + | | | + | | + | | + | | + | | + | | + | | | + | | + | | + | | + | | + | | | + | | + | | + | | + | | + | + | **hypothetical protein** |
| **SSGZ1_1551** | | | | | + | | + | | + | | + | | | + | | + | | + | | + | | + | | | + | | + | | + | | + | | + | | | + | | + | | + | | + | | + | | + | | | + | | + | | + | | + | | + | | | + | | + | | + | | + | | + | + | **SWIM domain-containing protein** |
| **SSGZ1_1552** | | | | | + | | + | | - | | + | | | + | | + | | + | | + | | + | | | + | | + | | + | | + | | + | | | + | | + | | + | | + | | + | | + | | | - | | + | | + | | + | | + | | | + | | + | | + | | + | | + | + | **hypothetical protein** |
| **SSGZ1_1553** | | | | | + | | - | | - | | - | | | - | | - | | - | | - | | - | | | - | | + | | - | | - | | + | | | - | | + | | + | | + | | + | | + | | | - | | - | | + | | + | | - | | | + | | + | | + | | + | | + | + | **Permease of the major facilitator superfamily** |
| **SSGZ1_1554** | | | | | + | | + | | + | | + | | | + | | + | | + | | + | | + | | | + | | + | | + | | + | | + | | | + | | + | | + | | + | | + | | + | | | + | | + | | + | | + | | + | | | + | | + | | + | | + | | + | + | **Small GTP-binding domain containing protein** |
| **SSGZ1_1555** | | | | | + | | + | | - | | + | | | + | | + | | + | | + | | + | | | + | | + | | + | | + | | + | | | + | | + | | + | | + | | + | | + | | | + | | + | | + | | + | | + | | | + | | + | | + | | + | | + | + | **putative nitroreductase** |
| **SSGZ1_1556** | | | | | + | | + | | - | | + | | | + | | + | | + | | + | | + | | | + | | + | | + | | + | | + | | | + | | + | | + | | + | | + | | + | | | - | | + | | + | | + | | + | | | + | | + | | + | | + | | + | + | **Primosomal DnaI** |
| **SSGZ1_1557** | | | | | + | | + | | - | | + | | | + | | + | | + | | + | | + | | | + | | + | | + | | + | | + | | | + | | + | | + | | + | | + | | + | | | - | | + | | + | | + | | + | | | + | | + | | + | | + | | + | + | **putative chromosome replication initiation** |
| **SSGZ1_1558** | | | | | + | | + | | - | | + | | | + | | + | | + | | + | | + | | | + | | + | | + | | + | | + | | | + | | + | | + | | + | | + | | + | | | - | | + | | + | | + | | + | | | + | | + | | + | | + | | + | + | **Predicted transcriptional regulator, consists of** |
| **SSGZ1_1559** | | | | | + | | + | | + | | + | | | + | | + | | + | | + | | + | | | + | | + | | + | | + | | + | | | + | | + | | + | | + | | + | | + | | | + | | + | | + | | + | | + | | | + | | + | | + | | + | | + | + | **hypothetical protein** |
| **SSGZ1_1560** | | | | | + | | + | | + | | + | | | + | | + | | + | | + | | + | | | + | | + | | + | | + | | + | | | + | | + | | + | | + | | + | | + | | | + | | + | | + | | + | | + | | | + | | + | | + | | + | | + | + | **probable permease** |
| **SSGZ1_1561** | | | | | + | | + | | + | | + | | | + | | + | | + | | + | | + | | | + | | + | | + | | + | | + | | | - | | + | | + | | + | | + | | + | | | + | | + | | + | | + | | + | | | + | | + | | + | | + | | + | + | **Response regulator** |
| **SSGZ1_1562** | | | | | + | | + | | + | | + | | | + | | + | | + | | + | | + | | | + | | + | | + | | + | | + | | | + | | + | | + | | + | | + | | + | | | + | | + | | + | | + | | + | | | + | | + | | + | | + | | + | + | **6-phosphogluconate dehydrogenase** |
| **SSGZ1_1563** | | | | | + | | + | | + | | + | | | + | | + | | + | | + | | + | | | + | | + | | + | | + | | + | | | + | | + | | + | | + | | + | | + | | | + | | + | | + | | + | | + | | | + | | + | | + | | + | | + | + | **YlbN-like hypothetical protein** |
| **SSGZ1_1564** | | | | | + | | + | | + | | + | | | + | | + | | + | | + | | + | | | + | | + | | + | | + | | + | | | + | | + | | + | | + | | + | | + | | | + | | + | | + | | + | | + | | | + | | + | | + | | + | | + | + | **thioredoxin reductase** |
| **SSGZ1_1565** | | | | | + | | + | | + | | + | | | + | | + | | + | | + | | + | | | + | | + | | + | | + | | + | | | + | | + | | + | | + | | + | | + | | | + | | + | | + | | + | | + | | | + | | + | | + | | + | | + | + | **hypothetical protein** |
| **SSGZ1_1566** | | | | | + | | + | | + | | + | | | + | | + | | + | | + | | + | | | + | | + | | + | | + | | + | | | + | | + | | + | | + | | + | | + | | | + | | + | | + | | + | | + | | | + | | + | | + | | + | | + | + | **putative ATP-dependent RNA helicase** |
| **SSGZ1_1567** | | | | | + | | + | | + | | + | | | + | | + | | + | | + | | + | | | + | | + | | + | | + | | + | | | + | | + | | + | | + | | + | | + | | | + | | + | | + | | + | | + | | | + | | + | | + | | + | | + | + | **ABC-type uncharacterized transport system,** |
| **SSGZ1_1568** | | | | | + | | + | | + | | + | | | + | | + | | + | | + | | + | | | + | | + | | + | | + | | + | | | + | | + | | + | | + | | + | | + | | | + | | + | | + | | + | | + | | | + | | + | | + | | + | | + | + | **Phospho-N-acetylmuramoyl-pentapeptide** |
| **SSGZ1_1569** | | | | | + | | + | | + | | + | | | + | | + | | + | | + | | + | | | + | | + | | + | | + | | + | | | + | | + | | + | | + | | + | | + | | | + | | + | | + | | + | | + | | | + | | + | | + | | + | | + | + | **Peptidoglycan glycosyltransferase** |
| **SSGZ1_1570** | | | | | + | | + | | + | | + | | | + | | + | | + | | + | | + | | | + | | + | | + | | + | | + | | | + | | + | | + | | + | | + | | + | | | + | | + | | + | | + | | + | | | + | | + | | + | | + | | + | + | **putative cell division protein FtsL** |
| **SSGZ1_1571** | | | | | + | | + | | + | | + | | | + | | + | | + | | + | | + | | | + | | + | | + | | + | | + | | | + | | + | | + | | + | | + | | + | | | + | | + | | + | | + | | + | | | + | | + | | + | | + | | + | + | **methyltransferase** |
| **SSGZ1_1572** | | | | | + | | + | | + | | + | | | + | | + | | + | | + | | + | | | + | | + | | + | | + | | + | | | + | | + | | + | | + | | + | | + | | | + | | + | | + | | + | | + | | | + | | + | | + | | + | | + | + | **Predicted transcriptional regulator** |
| **SSGZ1_1573** | | | | | + | | + | | + | | + | | | + | | + | | + | | + | | + | | | + | | + | | + | | + | | + | | | + | | + | | + | | + | | + | | + | | | + | | + | | + | | + | | - | | | + | | + | | + | | + | | + | + | **Hemolysins and related proteins containing CBS** |
| **SSGZ1_1574** | | | | | + | | + | | + | | + | | | + | | + | | + | | + | | + | | | + | | + | | + | | + | | + | | | + | | + | | + | | + | | + | | + | | | + | | + | | + | | + | | + | | | + | | + | | + | | + | | + | + | **hypothetical protein** |
| **SSGZ1_1575** | | | | | + | | + | | + | | + | | | + | | + | | + | | + | | + | | | + | | + | | + | | + | | + | | | + | | + | | + | | + | | + | | + | | | + | | + | | + | | + | | + | | | + | | + | | + | | + | | + | + | **hypothetical protein** |
| **SSGZ1_1576** | | | | | + | | + | | + | | + | | | + | | + | | + | | + | | + | | | + | | + | | + | | + | | + | | | + | | + | | + | | + | | + | | + | | | + | | + | | + | | + | | + | | | + | | + | | + | | + | | + | + | **Permease of the major facilitator superfamily** |
| **SSGZ1_1577** | | | | | + | | + | | + | | + | | | + | | + | | + | | + | | + | | | + | | + | | + | | + | | + | | | + | | + | | + | | + | | + | | + | | | + | | - | | + | | + | | + | | | + | | + | | + | | + | | + | + | **hypothetical protein** |
| **SSGZ1_1578** | | | | | + | | + | | + | | + | | | + | | + | | + | | + | | + | | | + | | + | | + | | + | | + | | | + | | + | | + | | + | | + | | + | | | + | | + | | + | | + | | + | | | + | | + | | + | | + | | + | + | **hypothetical protein** |
| **SSGZ1_1579** | | | | | + | | + | | + | | + | | | + | | + | | + | | + | | + | | | + | | + | | + | | + | | + | | | + | | + | | + | | + | | + | | + | | | + | | + | | + | | + | | + | | | + | | + | | + | | + | | + | + | **UbiA prenyltransferase** |
| **SSGZ1_1580** | | | | | + | | + | | + | | + | | | + | | + | | + | | + | | + | | | + | | + | | + | | + | | + | | | + | | + | | + | | + | | + | | + | | | + | | + | | + | | + | | + | | | + | | + | | + | | + | | + | + | **ApbE-like lipoprotein** |
| **SSGZ1_1581** | | | | | + | | + | | + | | + | | | + | | + | | + | | + | | + | | | + | | + | | + | | + | | + | | | + | | + | | + | | + | | + | | + | | | + | | + | | + | | + | | + | | | + | | + | | + | | + | | + | + | **pheromone cAD1 precursor lipoprotein** |
| **SSGZ1_1582** | | | | | + | | + | | + | | + | | | + | | + | | + | | + | | + | | | + | | + | | + | | + | | + | | | + | | + | | + | | + | | + | | + | | | + | | + | | + | | + | | + | | | + | | + | | + | | + | | + | + | **Polyprenyl synthetase** |
| **SSGZ1_1583** | | | | | + | | + | | + | | + | | | + | | + | | + | | + | | + | | | + | | + | | + | | + | | + | | | + | | + | | + | | + | | + | | + | | | + | | + | | + | | + | | + | | | + | | + | | + | | + | | + | + | **NADH dehydrogenase** |
| **SSGZ1_1584** | | | | | + | | + | | + | | + | | | + | | + | | + | | + | | + | | | + | | + | | + | | + | | + | | | + | | + | | + | | + | | + | | + | | | + | | + | | + | | + | | + | | | + | | + | | + | | + | | + | + | **hypothetical protein** |
[truncated: 102,410 more chars]
