# Supplementary material for: Identification of Genes and Genomic Islands Correlated with High Pathogenicity in Streptococcus suis Using Whole Genome Tilling Microarrays
Source: PLoS One. 2011 Mar 30;6(3):e17987. doi: 10.1371/journal.pone.0017987 (PMC3068143; doi:10.1371/journal.pone.0017987)
Supplement: Table S2 — Distribution of virulence related factors or immunogenic proteins in core or accessory genome of Streptococcus suis GZ1. (DOC) [file pone.0017987.s002.doc]

**Table S2 Distribution of virulence related factors or immunogenic proteins in core or accessory genome of *Streptococcus suis* GZ1**

| **Virulence related factor or immunogenic protein** | **Core genome** | **Accessory genome** | **Locus tags in GZ1 genome** | **References** |
| --- | --- | --- | --- | --- |
| AdcR | **+** |  | SSGZ1_0105 | Aranda J et al., 2010 |
| 2-Glyceraldehyde-3-phosphate dehydrogenase (GAPDH) | **+** |  | SSGZ1_0145 | Brassard et al., 2004 |
| Glutamine synthetase | **+** |  | SSGZ1_0149 | Si, Y., et al., 2009 |
| Extracellular factor (EF) |  | **+** | SSGZ1_0164 | Smith et al., 1997; Gottschalk et al., 1998 |
| Protease (dipeptidyl aminopeptidase IV) |  | **+** | SSGZ1_0184 | Ge, J., et al., 2009 |
| IgG-binding protein | **+** |  | SSGZ1_0208 | Benkirane et al., 1997, 1998 |
| Glutamate dehydrogenase | **+** |  | SSGZ1_0228 | Okwumabua et al., 2001 |
| Heat shock protein Hsp70 | **+** |  | SSGZ1_0276 | Benkirane et al., 1997 |
| LuxS | **+** |  | SSGZ1_0373 | X. G. Han and C. P. Lu, 2008; 2009 |
| O-acetylserine lyase | **+** |  | SSGZ1_0387 | Osaki et al., 2000 |
| SrtF pilus |  | **+** | RD15(SSGZ1_0420-SSGZ1_0424) | Daisuke Takamatsu et al., 2009 |
| Predicted metalloendopeptidase | **+** |  | SSGZ1_0461 | SUN Qiang-zheng et al., 2008 |
| Translation elongation factor EF-Tu | **+** |  | SSGZ1_0521 | SUN Qiang-zheng et al., 2008 |
| CPS2 |  | **+** | RD17(SSGZ1_0555-SSGZ1_0590) | Charland et al., 1998; Smith et al., 1999 |
| Arginine deiminase | **+** |  | SSGZ1_0615 | Winterhoff et al., 2002 |
| Muramidase released protein (Mrp) |  | **+** | SSGZ1_0743 | Smith et al., 1997; Gottschalk et al., 1998 |
| Proliprotein signal peptidase | **+** |  | SSGZ1_0769 | de Greeff et al., 2003 |
| Putative 5'-nucleotidase | **+** |  | SSGZ1_0885 | SUN Qiang-zheng et al., 2008 |
| Sortase A | **+** |  | SSGZ1_0946 | Vanier, G., et al.,2007; Changjun Wang et al., 2009 |
| Hyaluronate lyase |  | **+** | RD36(SSGZ1_1066-SSGZ1_1082) | Allen et al., 2004 |
| Cell wall hydrolase (Autolysin) |  | **+** | SSGZ1_1146 | Gu, H. and C. Lu, 2008 |
| Sao |  | **+** | SSGZ1_1217 | Yuanyi Li et al., 2006 |
| Suilysin |  | **+** | SSGZ1_1246 | Gottschalk et al., 1998; Allen et al., 2001; |
| Ferrous iron transport protein B | **+** |  | SSGZ1_1252 | Aranda, J., et al., 2009 |
| Fibronectin-binding protein | **+** |  | SSGZ1_1325 | de Greeff et al., 2002 |
| Alpha-enolase | **+** |  | SSGZ1_1335 | Esgleas, M., et al., 2008; Feng, Y., et al., 2009 |
| Superoxide dismutase | **+** |  | SSGZ1_1373 | Langford et al., 1991 |
| ABC-type branched-chain amino acid transport systems, periplasmic component | **+** |  | SSGZ1_1381 | SUN Qiang-zheng et al., 2008 |
| PgdA | **+** |  | SSGZ1_1467 | Fittipaldi, N., et al., 2009 |
| Serum opacity factor (Sof) |  | **+** | SSGZ1_1494 | Baums et al., 2006 |
| Dpr protein | **+** |  | SSGZ1_1519 | Arto Tapio Pulliainen et al., 2003 |
| CovR |  | **+** | SSGZ1_1561 | Xiuzhen Pan et al., 2009 |
| DNAse |  | **+** | SSGZ1_1784 | Fontaine et al., 2004 |
| Elongation factor TS | **+** |  | SSGZ1_1794 | Martinez et al., 2003 |
| Surface-associated subtilisin-like serine protease (SspA) |  | **+** | SSGZ1_1797 | Hu, Q., et al., 2009 |
| RevS |  | **+** | SSGZ1_1897 | Astrid de Greeff, et al., 2002  Ju, A.P., et al., 2008 |
| SrtBCD pilus |  | **+** | RD60(SSGZ1_1903-SSGZ1_1911) | Daisuke Takamatsu et al., 2009 |
| Inosine 5-monophosphate dehydrogenase (IMPDH). | **+** |  | SSGZ1_1978 | Zhang X.H., et al., 2009 |
| Total | 23 | 15 |  |  |
