# Supplementary material for: Identification of Genes and Genomic Islands Correlated with High Pathogenicity in Streptococcus suis Using Whole Genome Tilling Microarrays
Source: PLoS One. 2011 Mar 30;6(3):e17987. doi: 10.1371/journal.pone.0017987 (PMC3068143; doi:10.1371/journal.pone.0017987)
Supplement: Table S3 — Summary of genomic RDs and their distribution in 31 test strains according to their pathogenic capacity. (DOC) [file pone.0017987.s003.doc]

**Table S3 Summary of regions of difference and their distribution in 31 test strains.**

| Region of difference | HP^#^ strains | Non-HP strains | P value^$^ | Annotated functions | Position in GZ1 | Virulence  determinants | C+G % |
| --- | --- | --- | --- | --- | --- | --- | --- |
|  |  |  |  |  |  |  |  |
| RD1 | 7/7 | 17/24 | 0.16082789 | Abortive infection protein; hypothetical protein | 47170..48191 |  | 37.48 |
| RD2 | 7/7 | 19/24 | 0.562229638 | Hypothetical protein; ABC transporter | 49056..51825 |  | 40.18 |
| RD3 | 7/7 | 13/24 | 0.03311942 | Hypothetical protein; competence-specific global transcription regulator | 69530..70483 |  | 34.28 |
| RD4 | 7/7 | 8/24 | 0.002447164 | Integrase, catalytic core; phage hypothetical protein; replication initiation factor; hypothetical protein; cell division FtsK/SpoIIIE protein | 92725..97439 |  | 35.74 |
| RD5 | 7/7 | 22/24 | 1 | Binding-protein-dependent transport systems; glycoside hydrolase | 154660..158626 |  | 43.56 |
| RD6 | 7/7 | 3/24 | 4.56E-05 | EF; putative RTX family exoprotein | 161407..165101 | EF | 41.27 |
| RD7 | 7/7 | 17/24 | 0.16082789 | Transcriptional antiterminator; phosphotransferase system; putative sugar-specific permease; transketolase | 171752..177253 |  | 40.73 |
| RD8 | 7/7 | 5/24 | 0.000301189 | Transcriptional regulator; PTS lactose/cellobiose IIC component; ABC-type transport system; cation transport ATPase | 196309..211456 |  | 34.04 |
| RD9 | 7/7 | 21/24 | 1 | Murein hydrolase LrgA; LrgB; Formate/nitrite transporter | 254143..256158 |  | 37.65 |
| RD10 | 7/7 | 19/24 | 0.562229638 | Translation initiation factor 2 (IF-2); methyl-accepting chemotaxis protein | 259659..264472 |  | 42.29 |
| RD11 | 7/7 | 21/24 | 1 | Short-chain dehydrogenase/reductase; NADH: flavin oxidoreductase /NADH oxidase | 311142..313179 |  | 45.39 |
| RD12 | 7/7 | 1/24 | 3.04E-06 | Putative transcriptional regulator; hypothetical protein | 366030..373710 |  | 38.76 |
| RD13 | 7/7 | 22/24 | 1 | Hypothetical protein; Transcriptional regulator PadR-like protein | 384667..385615 |  | 37.41 |
| RD14 | 7/7 | 6/24 | 0.000652577 | Cell filamentation protein Fic-related protein; DNA or RNA helicases of superfamily II; hypothetical protein; transcriptional regulator | 444447..453071 |  | 33.58 |
| RD15 | 7/7 | 4/24 | 0.000125496 | Pilus structure | 458414..466556 | SrtF pilus | 39.44 |
| RD16 | 7/7 | 20/24 | 0.549721913 | Putative signal peptidase IB; hypothetical protein | 487950..489240 |  | 36.17 |
| RD17 | 7/7 | 3/24 | 4.56E-05 | Cps2; transposase; IS | 602866.. 634057 | CPS2 | 35.96 |
| RD18 | 7/7 | 20/24 | 0.549721913 | Hypothetical protein; plasmid stabilization system | 646968..647454 |  | 40.25 |
| RD19 | 7/7 | 19/24 | 0.562229638 | Hypothetical protein | 668700..670978 |  | 32.87 |
| RD20 | 7/7 | 12/24 | 0.026139585 | TPR repeat; hypothetical protein; putative type III restriction-modification system | 709816..714910 |  | 43.75 |
| RD21 | 7/7 | 2/24 | 1.37E-05 | Putative HsdR; transposase; putative HsdM; putative HsdS; hypothetical protein | 723957..733142 |  | 38.55 |
| RD22 | 7/7 | 17/24 | 0.16082789 | PTS lactose/cellobiose system; pyruvate formate lyase; transaldolase; glycerol dehydrogenase | 736262..746544 |  | 40.11 |
| RD23 | 7/7 | 18/24 | 0.292832492 | Oxidoreductase; hypothetical protein; Glycerol kinase; Glycerol-3-phosphate dehydrogenase; Glycerol uptake facilitator protein; glyoxalase family protein | 749167..758038 |  | 42.05 |
| RD24 | 7/7 | 9/24 | 0.006797676 | Putative hydrolase; phosphomethylpyrimidine kinase; hydroxyethylthiazole kinase; thiamine-phosphate pyrophosphorylase; cyclic nucleotide-binding; uridine phosphorylase; cobalt ABC transporter | 764967..772835 |  | 38.56 |
| RD25 | 7/7 | 20/24 | 0.549721913 | Protein involved in cell division; filamentation induced by cAMP protein Fic | 798973..799579 |  | 38.71 |
| RD26 | 7/7 | 19/24 | 0.562229638 | Hypothetical protein | 799600..800570 |  | 38.41 |
| RD27 | 7/7 | 20/24 | 0.549721913 | Hypothetical protein | 808808..810476 |  | 44.76 |
| RD28 | 7/7 | 22/24 | 1 | ATPase; putative restriction enzyme modulator protein | 883585..886340 |  | 36.9 |
| RD29 | 7/7 | 4/24 | 0.000125496 | Abortive infection bacteriophage resistance protein; hypothetical protein | 893715..894745 |  | 32.88 |
| RD30 | 7/7 | 8/24 | 0.002447164 | Putative bacteroiocin operon protein ScnG; ScnE; ABC transporter | 902913..905299 |  | 34.06 |
| RD31 | 7/7 | 15/24 | 0.076641282 | Rhodanese-like protein; coenzyme A disulfide reductase | 918434..920779 |  | 40.88 |
| RD32 | 7/7 | 20/24 | 0.549721913 | Phage integrase; hypothetical protein | 973632..977060 |  | 35.87 |
| RD33 | 7/7 | 22/24 | 1 | Hypothetical protein | 1022582..1023945 |  | 48.53 |
| RD34 | 7/7 | 22/24 | 1 | Transcriptional regulator PadR-like protein; membrane like protein | 1031391..1032817 |  | 40.36 |
| RD35 | 7/7 | 21/24 | 1 | Glycosidase; predicted phosphatase; reductase SDR; Mannonate dehydratase; glucuronate isomerase; KDPG and KHG aldolase; regulatory protein GntR; Beta-glucuronidase; Carbohydrate kinase, PfkB; sugar/sodium symporter | 1048602..1062290 |  | 42.31 |
| RD36 | 7/7 | 11/24 | 0.024501298 | LacI transcriptional regulator; hyaluronidase; PTS sugar-specific system; KDPG aldolase; Carbohydrate kinase, PfkB; Putative ribose 5-phosphate isomerase; oxidoreductase | 1105384..1122024 | Hyaluronidase | 40.35 |
| RD37 | 7/7 | 8/24 | 0.002447164 | Hypothetical protein | 1125851..1126309 |  | 37.47 |
| RD38 | 7/7 | 20/24 | 0.549721913 | Polysaccharide biosynthesis protein; putative ABC-type lipopolysaccharide transporter | 1171231..1178604 |  | 32.89 |
| RD39 | 7/7 | 20/24 | 0.549721913 | Erythromycin resistance ATP-binding protein; ABC transporter | 1245514..1246968 |  | 41.92 |
| RD40 | 7/7 | 3/24 | 4.56E-05 | Restriction-modification system | 1336815..1343465 |  | 35.75 |
| RD41 | 7/7 | 19/24 | 0.562229638 | Hypothetical protein; | 1346369..1348229 |  | 43.04 |
| RD42 | 7/7 | 8/24 | 0.002447164 | Hypothetical protein; SAM-dependent methyltransferase | 1353883..1354688 |  | 41.69 |
| RD43 | 7/7 | 16/24 | 0.146011047 | Glucosidase; PTS Sugar-specific permease; Transcriptional antiterminator bglG | 1375032..1379231 |  | 36.52 |
| RD44 | 7/7 | 8/24 | 0.002447164 | Cytotoxic translational repressor; hypothetical protein | 1386486..1387026 |  | 34.57 |
| RD45 | 7/7 | 5/24 | 0.000301189 | Bacteriophage protein; hypothetical protein; | 1401082..1411123 |  | 37.41 |
| RD46 | 7/7 | 22/24 | 1 | Sucrose phosphorylase; multiple sugar-binding ABC transporter | 1430764..1435366 |  | 38.58 |
| RD47 | 7/7 | 20/24 | 0.549721913 | Serum opacity factor; putative surface-anchored protein | 1523152..1528938 | SOF | 36.77 |
| RD48 | 7/7 | 22/24 | 1 | Primosomal DnaI; putative chromosome replication initiation;  Predicted transcriptional regulator | 1581351..1583534 |  | 43.36 |
| RD49 | 7/7 | 9/24 | 0.006797676 | Type I restriction-modification system; integrase; hypothetical protein | 1629917..1639210 |  | 41 |
| RD50 | 7/7 | 20/24 | 0.549721913 | Hypothetical protein; Pyridoxamine 5'-phosphate oxidase-related protein | 1736605..1737079 |  | 39.79 |
| RD51 | 7/7 | 16/24 | 0.146011047 | Response regulator; putative sensor histidine kinase; Predicted integral membrane protein; transposase; ABC transporter; glycosyl hydrolase-related protein; Transcriptional regulator/sugar kinase; hypothetical protein; putative alpha-1,2-mannosidase; putative endo-beta-N- acetylglucosaminidase | 1745573..1769213 |  | 43.42 |
| RD52 | 7/7 | 7/24 | 0.001305154 | Predicted acetyltransferase; hypothetical protein; outer surface protein; sugar PTS EIIBC; glucokinase regulatory protein; transcriptional regulator | 1775265..1780188 |  | 38.3 |
| RD53 | 7/7 | 3/24 | 4.56E-05 | MutT/NUDIX hydrolase family protein; nicotinamide mononucleotide transporter; transcriptional regulator; ATP-dependent Clp protease; transcriptional regulator CtsR | 1820834..1826499 |  | 47.07 |
| RD54 | 7/7 | 20/24 | 0.549721913 | Putative bacterocin transport accessory protein; acetyltransferase; | 1826649..1827688 |  | 46.06 |
| RD55 | 7/7 | 21/24 | 1 | Argininosuccinate lyase; argininosuccinate synthase | 1865563..1868194 |  | 44.11 |
| RD56 | 7/7 | 21/24 | 1 | 3H domain containing protein; uncharacterized conserved protein, phnB family | 1868979..1870523 |  | 45.05 |
| RD57 | 7/7 | 14/24 | 0.066067711 | Homocysteine methyltransferase; amino acid permease | 1871141..1873477 |  | 44.89 |
| RD58 | 7/7 | 22/24 | 1 | Amino acid ABC transporter, permease protein; putative amino-acid ABC transporter extracellular solute-binding protein | 1915654..1917129 |  | 43.56 |
| RD59 | 7/7 | 17/24 | 0.16082789 | PTS lactose/cellobiose system; transcriptional antiterminator bglG; hypothetical protein; beta-glucosidase; glycoside hydrolase | 1918221..1926700 |  | 39.91 |
| RD60 | 7/7 | 4/24 | 0.000125496 | Putative pilus structure; Hypothetical protein; | 1944710..1957895 | SrtBCD pilus | 38.44 |
| RD61 | 7/7 | 11/24 | 0.024501298 | Putative membrane protein; Response regulator: LytR/AlgR family | 1971184..1972064 |  | 42.79 |
| RD62 | 7/7 | 13/24 | 0.03311942 | Thermostable beta-glucosidase B; Hypothetical protein; Beta-glucosidase A; Sensory transduction protein lytT; ATP-binding ATPase-like protein | 1994286..2002729 |  | 41.63 |

^#^HP: highly pathogenic. All other strains are treated as non-HP.

$: P value based on Fisher exact test.
