# Supplementary material for: Identification of Genes and Genomic Islands Correlated with High Pathogenicity in Streptococcus suis Using Whole Genome Tilling Microarrays
Source: PLoS One. 2011 Mar 30;6(3):e17987. doi: 10.1371/journal.pone.0017987 (PMC3068143; doi:10.1371/journal.pone.0017987)
Supplement: Table S4 — Characteristics of putative genomic islands. GI features for 26 regions of difference (RDs) are described. (DOC) [file pone.0017987.s004.doc]

**Table S4 Summary of the GI features for 26 regions of difference (RDs).**

| **RD** | **Position in GZ1 genome** | **Length (bp)** | **GC content (%)** | **Dinucleotide bias^#^** | **Flanking repeat (bp)^$^** | **Type** | **Insertion site** | **Virulence-related and other notable factors encoded** |
| --- | --- | --- | --- | --- | --- | --- | --- | --- |
| **RD3** | 69530..70483 | 953 | 34.28 | bias | ND |  | ribosomal protein S10 |  |
| **RD4** | 92725..97439 | 4714 | 35.74 | bias | 19 | Putative prophage; remnant; Integrase | SSGZ1_tLeu001 (tRNA-Leu) |  |
| **RD8** | 196309..211456 | 15147 | 34.04 |  | ND |  |  |  |
| **RD14** | 444447..453071 | 8624 | 33.58 |  | 24 |  | SSGZ1_0409 (Valyl-tRNA synthetase, class Ia) |  |
| **RD15** | 458414..466556 | 8142 | 39.44 |  | ND | Transposase flanked |  | SrtF pilus |
| **RD16** | 487950..489240 | 1290 | 36.17 |  | ND | IS flanked |  |  |
| **RD17** | 602866..634057 | 31191 | 35.96 | bias | ND | Transposase, IS4; Transposase IS66; IS flanked |  | CPS2 |
| **RD18** | 646968..647454 | 486 | 40.25 | bias | ND |  | SSGZ1_0606 (Asparaginyl-tRNA synthetase) |  |
| **RD20** | 709816..714910 | 5094 | 43.75 | bias | ND | IS flanked |  | Type Ⅲ R/M |
| **RD21** | 723957..733142 | 9185 | 38.55 |  | ND | Transposase, IS4 |  | Type Ⅰ R/M |
| **RD28** | 883585..886340 | 2755 | 36.9 |  | ND |  |  | Putative restriction enzyme modulator protein |
| **RD29** | 893715..894745 | 1030 | 32.88 | bias | 21 | Putative prophage; remnant; Integrase flanked |  |  |
| **RD30** | 902913..905299 | 2386 | 34.06 |  | 37 |  |  | ScnG;ScnE |
| **RD32** | 973632..977060 | 3428 | 35.87 |  | 19 | Phage integrase |  |  |
| **RD34** | 1031391..1032817 | 1426 | 40.36 | bias | ND | IS flanked |  |  |
| **RD36** | 1105384..1122024 | 16640 | 40.35 |  | ND | Transposase |  | Hyaluronidase |
| **RD37** | 1125851..1126309 | 458 | 37.47 | bias | ND |  | SSGZ1_1093(Alanyl-tRNA synthetase, class Ⅱc) |  |
| **RD40** | 1336815..1343465 | 6650 | 35.75 |  | 19 |  |  | Type Ⅰ R/M |
| **RD44** | 1386486..1387026 | 540 | 34.57 | bias | 18 |  |  |  |
| **RD45** | 1401082..1411123 | 10041 | 37.41 | bias | 18 | Prophage; integrase; | SSGZ1_1365(Ribosomal protein L31) | Virulence-associated protein E |
| **RD49** | 1629917..1639210 | 9293 | 41 |  | 19 | Integrase |  | Type Ⅰ R/M |
| **RD50** | 1736605..1737079 | 474 | 39.79 | bias | ND |  | SSGZ1_1712(ribosomal protein S9) |  |
| **RD51** | 1745573..1769213 | 23640 | 43.42 | bias | ND | Integrase |  |  |
| **RD53** | 1820834..1826499 | 5665 | 47.07 | bias | ND |  |  |  |
| **RD54** | 1826649..1827688 | 1039 | 46.06 | bias | 18 |  |  |  |
| **RD60** | 1944710..1957895 | 13185 | 38.44 | bias | ND |  |  | SrtBCD pilus |

^#^ Calculated by bioinformatics tool IslandViewer.

^$^ direct repeat.

ND: not detected.
