# Supplementary material for: Identification of Genes and Genomic Islands Correlated with High Pathogenicity in Streptococcus suis Using Whole Genome Tilling Microarrays
Source: PLoS One. 2011 Mar 30;6(3):e17987. doi: 10.1371/journal.pone.0017987 (PMC3068143; doi:10.1371/journal.pone.0017987)
Supplement: Table S5 — Summary of SVG and their distribution in 31 test strains according to their pathogenic capacity. (DOC) [file pone.0017987.s005.doc]

**Summary of singular variable genes and their distribution in 31 sample strains.**

| Singular variable gene | HP^#^ strains | Non-HP strains | P value^$^ | Annotated functions | Virulence  determinants |
| --- | --- | --- | --- | --- | --- |
| SSGZ1_0034 | 7/7 | 14/24 | 0.066067711 | Hypothetical protein |  |
| SSGZ1_0035 | 7/7 | 22/24 | 1 | Hypothetical protein |  |
| SSGZ1_0046 | 7/7 | 14/24 | 0.066067711 | GCN5-related N-acetyltransferase |  |
| SSGZ1_0101 | 7/7 | 20/24 | 0.549721913 | Hypothetical protein |  |
| SSGZ1_0144 | 7/7 | 18/24 | 0.292832492 | Endothelin-converting enzyme 1 |  |
| SSGZ1_0147 | 7/7 | 22/24 | 1 | Hypothetical protein |  |
| SSGZ1_0167 | 7/7 | 22/24 | 1 | PIG-X/PBN1 |  |
| SSGZ1_0177 | 0/7 | 15/24 | 0.006797676 | GCN5-related N-acetyltransferase |  |
| SSGZ1_0182 | 0/7 | 13/24 | 0.024501298 | Surface protein from Gram-positive cocci, anchor |  |
| SSGZ1_0186 | 3/7 | 24/24 | 1 | Beta-lactamase |  |
| SSGZ1_0217 | 7/7 | 20/24 | 0.549721913 | Acetyltransferase, GNAT family protein |  |
| SSGZ1_0221 | 7/7 | 16/24 | 0.146011047 | Transcriptional activator |  |
| SSGZ1_0222 | 7/7 | 18/24 | 0.292832492 | Bacteriocin-associated integral membrane |  |
| SSGZ1_0231 | 7/7 | 21/24 | 1 | Predicted hydrolase of the HAD superfamily |  |
| SSGZ1_0237 | 7/7 | 19/24 | 0.562229638 | Hypothetical protein |  |
| SSGZ1_0278 | 7/7 | 22/24 | 1 | Lipoate-protein ligase A |  |
| SSGZ1_0283 | 7/7 | 21/24 | 1 | Hypothetical protein |  |
| SSGZ1_0292 | 7/7 | 19/24 | 0.562229638 | Hypothetical protein |  |
| SSGZ1_0298 | 7/7 | 17/24 | 0.16082789 | Hypothetical protein |  |
| SSGZ1_0301 | 7/7 | 11/24 | 0.024501298 | Lipase |  |
| SSGZ1_0304 | 7/7 | 18/24 | 0.292832492 | Glycosyl transferase, group 2 family protein |  |
| SSGZ1_0324 | 7/7 | 15/24 | 0.076641282 | Deoxyguanosine triphosphate triphosphohydrolase-related protein |  |
| SSGZ1_0340 | 7/7 | 10/24 | 0.008701026 | Hypothetical protein |  |
| SSGZ1_0458 | 7/7 | 22/24 | 1 | Putative biotin synthase (BioY family protein) |  |
| SSGZ1_0520 | 7/7 | 17/24 | 0.16082789 | Hypothetical protein |  |
| SSGZ1_0536 | 7/7 | 19/24 | 0.562229638 | YSIRK Gram-positive signal peptide |  |
| SSGZ1_0676 | 7/7 | 21/24 | 1 | Predicted membrane protein |  |
| SSGZ1_0681 | 7/7 | 21/24 | 1 | FAD-dependent pyridine nucleotide-disulphide |  |
| SSGZ1_0743 | 7/7 | 18/24 | 0.292832492 | Muramidase-released protein | Mrp |
| SSGZ1_0748 | 7/7 | 14/24 | 0.066067711 | ABC-type phosphate transport system, ATPase |  |
| SSGZ1_0792 | 7/7 | 15/24 | 0.076641282 | Chloride channel protein, EriC |  |
| SSGZ1_0821 | 7/7 | 7/24 | 0.001305154 | Hypothetical protein |  |
| SSGZ1_0866 | 7/7 | 22/24 | 1 | Hypothetical protein |  |
| SSGZ1_0867 | 7/7 | 18/24 | 0.292832492 | Transcription activator |  |
| SSGZ1_0903 | 7/7 | 21/24 | 1 | Anchor region containing Surface protein |  |
| SSGZ1_0904 | 7/7 | 14/24 | 0.066067711 | Hypothetical protein |  |
| SSGZ1_0912 | 7/7 | 22/24 | 1 | Hypothetical protein |  |
| SSGZ1_0964 | 4/7 | 21/24 | 0.110027286 | Hypothetical protein |  |
| SSGZ1_0979 | 7/7 | 22/24 | 1 | Hypothetical protein |  |
| SSGZ1_0998 | 7/7 | 21/24 | 1 | Regulatory protein, MarR |  |
| SSGZ1_1000 | 7/7 | 21/24 | 1 | Hypothetical protein |  |
| SSGZ1_1002 | 7/7 | 22/24 | 1 | Putative permease |  |
| SSGZ1_1003 | 7/7 | 22/24 | 1 | Putative 2-amino-4- hydroxy-6-hydroxymethylpteridine pyrophosphokinase |  |
| SSGZ1_1005 | 7/7 | 21/24 | 1 | Dihydropteroate synthase |  |
| SSGZ1_1007 | 7/7 | 22/24 | 1 | NUDIX hydrolase |  |
| SSGZ1_1013 | 7/7 | 20/24 | 0.549721913 | GCN5-related N-acetyltransferase |  |
| SSGZ1_1045 | 7/7 | 20/24 | 0.549721913 | GCN5-related N-acetyltransferase |  |
| SSGZ1_1134 | 7/7 | 19/24 | 0.562229638 | Glycosyl transferase, family 2 |  |
| SSGZ1_1147 | 7/7 | 22/24 | 1 | Surface protein from Gram-positive cocci, anchor |  |
| SSGZ1_1162 | 7/7 | 21/24 | 1 | Hypothetical protein |  |
| SSGZ1_1217 | 7/7 | 19/24 | 0.562229638 | Surface antigen SP1 | Sao |
| SSGZ1_1246 | 7/7 | 13/24 | 0.03311942 | Suilysin | Suilysin |
| SSGZ1_1265 | 7/7 | 22/24 | 1 | Hypothetical protein |  |
| SSGZ1_1273 | 7/7 | 22/24 | 1 | Integral membrane protein |  |
| SSGZ1_1311 | 7/7 | 21/24 | 1 | Hypothetical protein |  |
| SSGZ1_1339 | 7/7 | 21/24 | 1 | Glycerate kinase |  |
| SSGZ1_1400 | 7/7 | 16/24 | 0.146011047 | Putative ion transport protein |  |
| SSGZ1_1408 | 7/7 | 20/24 | 0.549721913 | Internalin protein |  |
| SSGZ1_1417 | 7/7 | 22/24 | 1 | Hypothetical protein |  |
| SSGZ1_1420 | 7/7 | 7/24 | 0.001305154 | Membrane protein, putative |  |
| SSGZ1_1452 | 7/7 | 20/24 | 0.549721913 | Putative NAD(P)H oxidoreductaseNusG |  |
| SSGZ1_1544 | 7/7 | 21/24 | 1 | Acylphosphatase |  |
| SSGZ1_1545 | 0/7 | 19/24 | 0.000301189 | Putative inner membrane protein |  |
| SSGZ1_1552 | 7/7 | 22/24 | 1 | Hypothetical protein |  |
| SSGZ1_1553 | 0/7 | 16/24 | 0.002447164 | Permease of the major facilitator superfamily |  |
| SSGZ1_1589 | 7/7 | 15/24 | 0.076641282 | Hypothetical protein |  |
| SSGZ1_1636 | 7/7 | 19/24 | 0.562229638 | Ribonucleases G and E |  |
| SSGZ1_1638 | 7/7 | 11/24 | 0.024501298 | PTS system IIABC components |  |
| SSGZ1_1654 | 7/7 | 10/24 | 0.008701026 | Hypothetical protein |  |
| SSGZ1_1709 | 7/7 | 16/24 | 0.146011047 | Predicted metal-sulfur cluster biosynthetic |  |
| SSGZ1_1717 | 7/7 | 10/24 | 0.008701026 | Protein kinase |  |
| SSGZ1_1739 | 7/7 | 21/24 | 1 | Purine and other phosphorylases |  |
| SSGZ1_1740 | 7/7 | 11/24 | 0.024501298 | Serine O-acetyltransferase |  |
| SSGZ1_1760 | 7/7 | 21/24 | 1 | 3-isopropylmalate dehydratase large subunit |  |
| SSGZ1_1767 | 7/7 | 18/24 | 0.292832492 | LemA |  |
| SSGZ1_1774 | 7/7 | 20/24 | 0.549721913 | Major intrinsic protein |  |
| SSGZ1_1796 | 7/7 | 13/24 | 0.03311942 | tRNA-Cys |  |
| SSGZ1_1798 | 7/7 | 21/24 | 1 | Transcription antitermination protein NusG |  |
| SSGZ1_1812 | 7/7 | 15/24 | 0.076641282 | Metallo cofactor biosynthesis protein |  |
| SSGZ1_1813 | 7/7 | 13/24 | 0.03311942 | Transcriptional activator |  |
| SSGZ1_1816 | 7/7 | 21/24 | 1 | Permease of the major facilitator superfamily |  |
| SSGZ1_1822 | 7/7 | 11/24 | 0.024501298 | RelE/ParE family protein |  |
| SSGZ1_1823 | 7/7 | 20/24 | 0.549721913 | Hypothetical protein |  |
| SSGZ1_1859 | 7/7 | 18/24 | 0.292832492 | Hypothetical protein |  |
| SSGZ1_1860 | 7/7 | 11/24 | 0.024501298 | Hypothetical protein |  |
| SSGZ1_1861 | 7/7 | 17/24 | 0.16082789 | Serine/threonine protein phosphatase |  |
| SSGZ1_1896 | 7/7 | 7/24 | 0.000842912 | Hypothetical protein |  |
| SSGZ1_1897 | 7/7 | 14/24 | 0.066067711 | RevS (revS) | RevS |
| SSGZ1_1916 | 7/7 | 21/24 | 1 | Hypothetical protein |  |
| SSGZ1_1918 | 7/7 | 18/24 | 0.292832492 | Hypothetical protein |  |
| SSGZ1_1922 | 7/7 | 18/24 | 0.292832492 | Metallophosphoesterase |  |
| SSGZ1_1961 | 7/7 | 13/24 | 0.03311942 | Putative MutT/NudX family protein |  |
| SSGZ1_1962 | 7/7 | 19/24 | 0.562229638 | MutT/nudix family protein tetriphosphohydrolase related protein |  |

^#^HP: highly pathogenic. All other strains are treated as non-HP.

$: P value based on Fisher exact test.
